# Supplementary material for: Statistical guidelines for quality control of next-generation sequencing techniques
Source: Life Sci Alliance. 2021 Aug 30;4(11):e202101113. doi: 10.26508/lsa.202101113 (PMC8408346; doi:10.26508/lsa.202101113)
Supplement: Supplementary file 1 [file LSA-2021-01113_Supplemental_Data_1.pdf]

Decision trees for Quality Control of NGS files

Practical guidelines for decision making

Authors:

Maximilian Sprang

Matteo Krüger

Prof. Miguel A. Andrade-Navarro

Dr. Jean-Fred Fontaine

Johannes Gutenberg-University Mainz

Germany

9 March 2021

Table of Contents II

Abstract V

Quality Features VI

High-Level Decision Trees:

Group 1

human paired-ended ChIP-seq 1
human single-ended ChIP-seq 2
human paired-ended DNase-seq 3
human single-ended RNA-seq 4
mouse single-ended ChIP-seq 5
mouse paired-ended DNase-seq 6

Group 2

human paired-ended ControlChIP-seq 7
human single-ended ControlChIP-seq 8
human paired-ended DNase-seq 9
human paired-ended HistoneChIP-seq 10
human single-ended HistoneChIP-seq 11
human single-ended polyAplusRNA-seq 12
human single-ended smallRNA-seq 13
human paired-ended TFChIP-seq 14
human single-ended TFChIP-seq for 15
mouse single-ended ControlChIP-seq 16
mouse paired-ended DNase-seq 17
mouse single-ended HistoneChIP-seq 18
mouse single-ended TFChIP-seq 19

Low-Level Decision Trees:

Group A

human paired-ended ControlChIP-seq in PC-9 20
human single-ended ControlChIP-seq in GM12878 21
human single-ended ControlChIP-seq in HepG2 22
human paired-ended DNase-seq in body_of_pancreas 23
human paired-ended DNase-seq in limb 24
human paired-ended DNase-seq in neural_progenitor_cell 25
human paired-ended DNase-seq in thyroid_gland 26
human paired-ended DNase-seq in transverse_colon 27
human single-ended HistoneChIP-seq in gastrocnemius_medialis 28
human single-ended HistoneChIP-seq in MCF-7 29
human single-ended polyAplusRNA-seq in GM12878 30
human paired-ended TFChIP-seq in GM12878 31
human paired-ended TFChIP-seq in HEK293 32
human paired-ended TFChIP-seq in HepG2 33

human paired-ended TFChIP-seq in K562 34
human paired-ended TFChIP-seq in MCF-7 35
human single-ended TFChIP-seq in A549 36
human single-ended TFChIP-seq in GM12878 37
human single-ended TFChIP-seq in H1 38
human single-ended TFChIP-seq in HeLa-S3 39
human single-ended TFChIP-seq in HepG2 40
human single-ended TFChIP-seq in IMR-90 41
human single-ended TFChIP-seq in K562 42
human single-ended TFChIP-seq in MCF-7 43
human single-ended TFChIP-seq in MCF_10A 44
human single-ended TFChIP-seq in SK-N-SH 45
mouse single-ended ControlChIP-seq in MEL_cell_line 46
mouse paired-ended DNase-seq in hindbrain 47
mouse paired-ended DNase-seq in limb 48
mouse paired-ended DNase-seq in midbrain 49
mouse single-ended TFChIP-seq in CH12.LX 50
mouse single-ended TFChIP-seq in MEL_cell_line 51
Group B
human paired-ended ControlChIP-seq for Control 52
human single-ended ControlChIP-seq for Control 53
human single-ended ControlChIP-seq for rabbit-IgG-control 54
human single-ended HistoneChIP-seq for H3K27ac 55
human single-ended HistoneChIP-seq for H3K27me3 56
human single-ended HistoneChIP-seq for H3K36me3 57
human single-ended HistoneChIP-seq for H3K4me1 58
human single-ended HistoneChIP-seq for H3K4me2 59
human single-ended HistoneChIP-seq for H3K4me3 60
human single-ended HistoneChIP-seq for H3K9me3 61
human single-ended TFChIP-seq for BACH1 62
human single-ended TFChIP-seq for CHD1 63
human single-ended TFChIP-seq for CTCF 64
human single-ended TFChIP-seq for ELK1 65
human single-ended TFChIP-seq for EP300 66
human single-ended TFChIP-seq for FOS 67
human single-ended TFChIP-seq for JUND 68
human single-ended TFChIP-seq for MAX 69
human single-ended TFChIP-seq for MAZ 70
human single-ended TFChIP-seq for MXI1 71
human single-ended TFChIP-seq for MYC 72
human single-ended TFChIP-seq for NR3C1 73
human single-ended TFChIP-seq for POLR2A 74
human single-ended TFChIP-seq for RCOR1 75
human single-ended TFChIP-seq for RELA 76
human single-ended TFChIP-seq for STAT3 77
human single-ended TFChIP-seq for TCF7L2 78
mouse single-ended ControlChIP-seq for Control 79
mouse single-ended HistoneChIP-seq for H3K27ac 80
mouse single-ended HistoneChIP-seq for H3K27me3 81
mouse single-ended HistoneChIP-seq for H3K36me3 82

mouse single-ended HistoneChIP-seq for H3K4me1 83
mouse single-ended HistoneChIP-seq for H3K4me3 84
mouse single-ended HistoneChIP-seq for H3K9me3 85
mouse single-ended TFChIP-seq for CTCF 86
mouse single-ended TFChIP-seq for POLR2A 87

Group C

human single-ended ControlChIP-seq for rabbit-IgG-control (ENCAB000AOJ) 88
human single-ended HistoneChIP-seq for H3K27ac (ENCAB000AQN) 89
human single-ended HistoneChIP-seq for H3K27me3 (ENCAB000ANB) 90
human single-ended HistoneChIP-seq for H3K36me3 (ENCAB000ADU) 91
human single-ended HistoneChIP-seq for H3K4me1 (ENCAB000ADW) 92
human single-ended TFChIP-seq for BACH1 (ENCAB000AEA) 93
human single-ended TFChIP-seq for FOS (ENCAB000AEQ) 94
human single-ended TFChIP-seq for JUND (ENCAB000AID) 95
human single-ended TFChIP-seq for MAX (ENCAB000AIL) 96
human single-ended TFChIP-seq for MAZ (ENCAB000AIM) 97
human single-ended TFChIP-seq for MXI1 (ENCAB000AIT) 98
human single-ended TFChIP-seq for MYC (ENCAB000AET) 99
human single-ended TFChIP-seq for POLR2A (ENCAB000AOC) 100
human single-ended TFChIP-seq for RELA (ENCAB000AJG) 101
human single-ended TFChIP-seq for STAT3 (ENCAB000ALH) 102
human single-ended TFChIP-seq for TCF7L2 (ENCAB000AOA) 103
mouse single-ended TFChIP-seq for CTCF (ENCAB210NHK) 104
mouse single-ended TFChIP-seq for POLR2A (ENCAB281FBQ) 105

Abstract

As more and more next-generation sequencing (NGS) datasets are made available, the means to assure their quality increase in importance and it is imperative to combine tools and data features for understanding quality issues more efficiently. Our aim is to derive data-driven guidelines for the quality control of NGS data files.

We used multiple quality control or analysis tools on 2098 FastQ files from ENCODE marked as of low- or high-quality to derive a feature set of 47 quality features, that we investigated for their power in classifying the quality of NGS files in machine-learning-based decision trees (CART algorithm, scikit-learn).

We first studied the data files in high-level subsets defined by unique combinations of the following annotations:

- Group 1: subsets defined by organism, runtype and assay type (e.g. human single-ended ChIP-seq)
- Group 2: subsets defined by organism, runtype and assay title (e.g. mouse paired-ended histone ChIP-seq)

The most powerful features were the genome mapping statistics, but their power varied strongly from assay to assay. When relevant, we report for each tree the most frequent biological samples, ChIP protein targets or ChIP antibody that were used to build the decision tree model. A model mostly based on a very few types of biological samples or protein targets/antibodies is likely to be biased towards those types of files. We could observe that sometimes the first split in the decision tree was not made only due to quality differences but stemmed from particular files. An example can be found with the tree on page 16, mouse single-end control Chip-seq, for which most low-quality files are related to MEL cell lines.

To further investigate the classification power, we investigated the data in experiment-sized lower-level subsets, divided into three groups:

- Group A: subsets defined by assay title, organism, runtype and biological sample
- Group B: subsets defined by assay title, organism, runtype, and ChIP protein target
- Group C: subsets defined by assay title, organism, runtype, ChIP protein target, and ChIP antibody

A model built using files distributed equally (or not very biased) among various biological samples or ChIP proteins/antibodies will be more reliable. An example of probably unbiased 1-feature perfect classification can be seen with the tree on page 33, human paired-ended TF ChIP-seq in HepG2 cells. Yet, the classification performance must be moderated by the low number of files used to build the model (n=10).

This document contains decision trees with additional information about the counts of biological samples or ChIP proteins and antibodies in the observed data subset, as well as the classification metrics achieved with the same set. We did not use a cross validation approach here, since we had to deal with very small subsets at times and did not plan to use the trained trees as a classifier. Rather, these trees are used to emulate how a human user would look at the features we computed to assess the quality of NGS data.

Citations and Links:

- Interactive dashboard to see quality metrics in user-defined subsets (https://cbdm.uni-mainz.de/ngs-guidelines/)
- Software for automated classification of NGS files by quality including a script to generate the quality features: Albrecht et. al., 2021 (doi: https://doi.org/10.1186/s13059-021-02294-2)

Quality Features

feature set	feature	Definition
RAW	Basic_Statistics	Generates some simple composition statistics, like file name and type but also the overall GC content. Never raises a warning/failure and is only kept for completeness. Values: 0 if failure, 1 if warning, 2 if pass.
RAW	Per_base_sequence_quality	Based on the range of phred quality scores across all bases of a read. A warning/failure is returned if any base has a lower quantile below 10/5. Values: 0 if failure, 1 if warning, 2 if pass.
RAW	Per_tile_sequence_quality	Averaged base quality over flow tiles of the machine generating the files (only usable with Illumina machines). Values: 0 if failure, 1 if warning, 2 if pass.
RAW	Per_sequence_quality_scores	Based on mean phred score for all sequences, raises warning/error when the most frequent mean quality is below 27/20. Values: 0 if failure, 1 if warning, 2 if pass.
RAW	Per_base_sequence_content	Based on the content of each base in given sequence, warning/failure when difference between A and T or G and C is greater than 10%/20%. Values: 0 if failure, 1 if warning, 2 if pass.
RAW	Per_sequence_GC_content	Based on GC content in given sequence, warning/failure if more than 15%/30% of reads deviate from normal distribution over their mean GC content. Values: 0 if failure, 1 if warning, 2 if pass.
RAW	Per_base_N_content	Based on the content of non-callable bases in given position, warning/failure if any position shows more than 5%/10%. Values: 0 if failure, 1 if warning, 2 if pass.
RAW	Sequence_Length_Distribution	Based on the distribution of fragment sizes in the file, warning if any of the sequences differ in length, failure when any sequence has a length of zero. Values: 0 if failure, 1 if warning, 2 if pass.
RAW	Sequence_Duplication_Levels	Based on the degree of duplication for every sequence in a library, warning/failure issued if non-unique sequences make up more than 20%/50% in total. Values: 0 if failure, 1 if warning, 2 if pass.
RAW	Overrepresented_sequences	Based on the count of sequences that are overrepresented in distribution of a file, warning/failure is issued if any sequence is found to represent more than 0.1%/1% of total. Values: 0 if failure, 1 if warning, 2 if pass.
RAW	Adapter_Content	Based on specialized Kmer content, for adapter sequences. warning/failure is issued if any sequence is present in more than 5%/10% of all reads. Values: 0 if failure, 1 if warning, 2 if pass.
RAW	Kmer_Content	Based on the measure of the number of each 7-mer at each position in the library and uses binomial test to for significant deviations from even coverage at all positions. Warning/failure issued if any Kmer is imbalanced with a binomial p-value < 0.01/10e-5. Values: 0 if failure, 1 if warning, 2 if pass.
MAP_SE	no_mapping	Percentage of reads that could not be mapped to reference genome in a single-ended experiment
MAP_SE	uniquely	Percentage of reads that are mapped to a unique location in a single-ended experiment
MAP_SE	multiple	Percentage of reads that were mapped to multiple locations in a single-ended experiment
MAP_SE	Overall	Percentage of reads that could be mapped to reference genome in a single-ended experiment
MAP_PE	con_no_mapping	Percentage of read pairs that could not be mapped concordantly to reference genome in a paired-ended experiment
MAP_PE	con_uniquely	Percentage of read pairs that are mapped concordantly to a unique location in a paired-ended experiment
MAP_PE	con_multiple	Percentage of read pairs that were mapped concordantly to multiple locations in a paired-ended experiment
MAP_PE	dis_uniquely	Percentage of read pairs that are mapped discordantly to a unique location in a paired-ended experiment
MAP_PE	cod_no_mapping	Percentage of read pairs that could not be mapped concordantly or discordantly to reference genome in a paired-ended experiment

MAP_PE	cod_uniquely	Percentage of read pairs that are mapped concordantly or discordantly to a unique location in a paired-ended experiment
MAP_PE	cod_multiple	Percentage of read pairs that were mapped concordantly or discordantly to multiple locations in a paired-ended experiment
MAP_PE	overall	Percentage of reads that could be mapped to reference genome in a paired-ended experiment
MAP_MI	no_mapping	Equals either MAP_SE_no_mapping for single-ended experiments, or MAP_PE_con_no_mapping for paired-ended experiments
MAP_MI	uniquely	Equals either MAP_SE_uniquely for single-ended experiments, or MAP_PE_con_uniquely for paired-ended experiments
MAP_MI	multiple	Equals either MAP_SE_multiple for single-ended experiments, or MAP_PE_con_multiple for paired-ended experiments
MAP_MI	Overall	Equals either MAP_SE_overall for single-ended experiments, or MAP_PE_con_overall for paired-ended experiments
LOC	Promoter	Percentage of reads in promoter regions
LOC	5_UTR	Percentage of reads in 5' UTR regions
LOC	3_UTR	Percentage of reads in 3' UTR regions
LOC	1st_Exon	Percentage of reads in 1st exon regions
LOC	Other_Exon	Percentage of reads in non-first exon regions
LOC	1st_Intron	Percentage of reads in 1st intron regions
LOC	Other_Intron	Percentage of reads in non-first intron regions
LOC	Downstream	Percentage of reads in downstream gene regions
LOC	Distal_Intergenic	Percentage of reads in distal intergenic regions
TSS	-4500	Percentage of reads in -5k, -4k region relative to TSS
TSS	-3500	Percentage of reads in [-4k, -3k] bp region relative to transcription start sites
TSS	-2500	Percentage of reads in [-3k, -2k] bp region relative to transcription start sites
TSS	-1500	Percentage of reads in [-2k, -1k] bp region relative to transcription start sites
TSS	-500	Percentage of reads in [-1k, 0] bp region relative to transcription start sites
TSS	500	Percentage of reads in [0k, 1k] bp region relative to transcription start sites
TSS	1500	Percentage of reads in [1k, 2k] bp region relative to transcription start sites
TSS	2500	Percentage of reads in [2k, 3k] bp region relative to transcription start sites
TSS	3500	Percentage of reads in [3k, 4k] bp region relative to transcription start sites
TSS	4500	Percentage of reads in [4k, 5k] bp region relative to transcription start sites

Features derived by the following tools: fastqc (RAW), bowtie2 (MAP), ChIPseeker (LOC) and ChIPpeakAnno (TSS).

Group 1
human paired-ended ChIP-seq

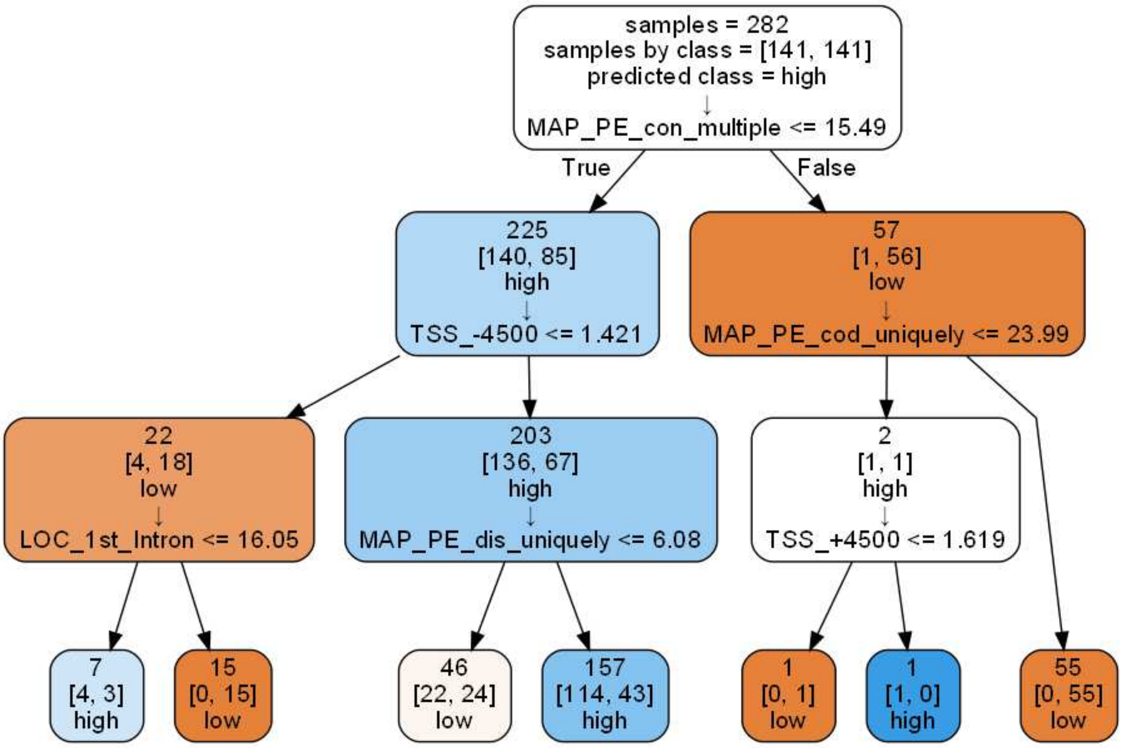

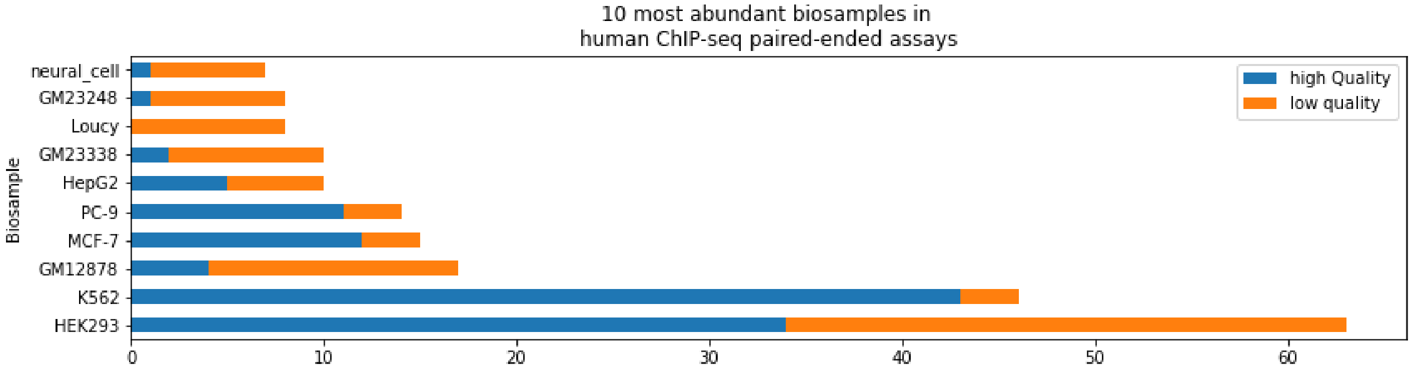

Metrics on training set:
- Accuracy: 0.76

High-quality files:
- Precision: 0.72
- Recall: 0.84
- F1-score: 0.78

Low-quality files
- Precision: 0.81
- Recall: 0.67
- F1-score: 0.74

Legend:
Nodes show data state and decision criteria to apply
Samples by class: [high-quality files, low-quality files]

Group 1
human single-ended ChIP-seq

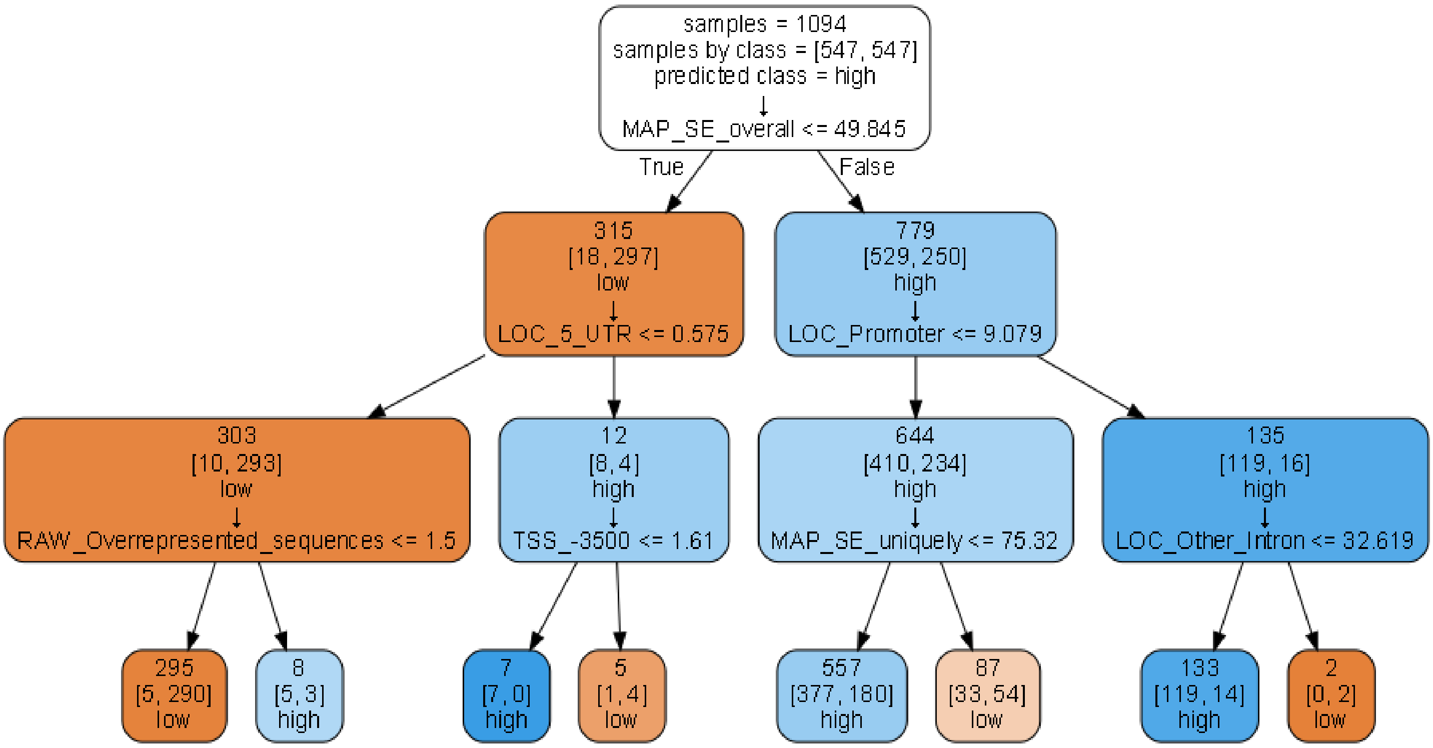

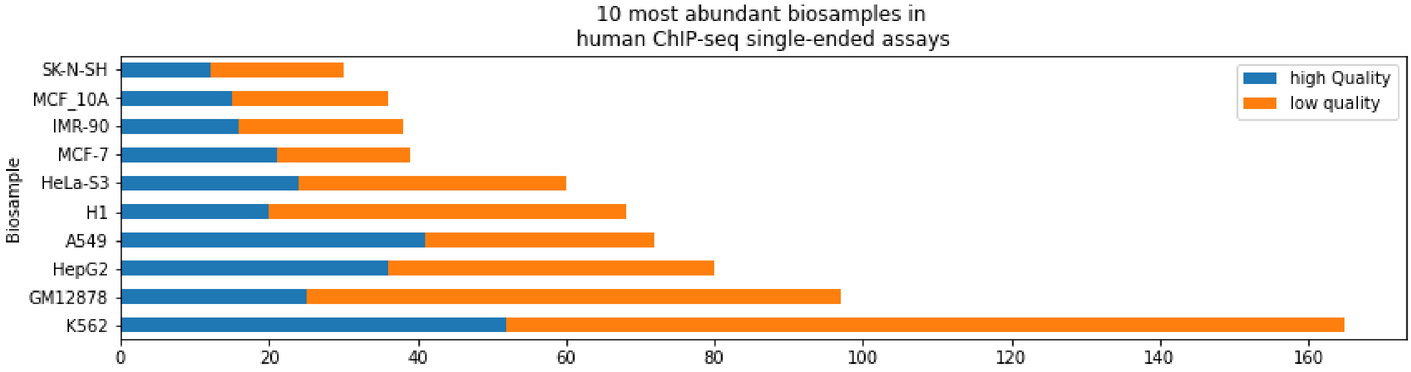

Metrics on training set:
- Accuracy: 0.78

High-quality files:
- Precision: 0.72
- Recall: 0.93
- F1-score: 0.81

Low-quality files
- Precision: 0.9
- Recall: 0.64
- F1-score: 0.75

Legend:
Nodes show data state and decision criteria to apply
Samples by class: [high-quality files, low-quality files]

Group 1

human paired-ended DNase-seq

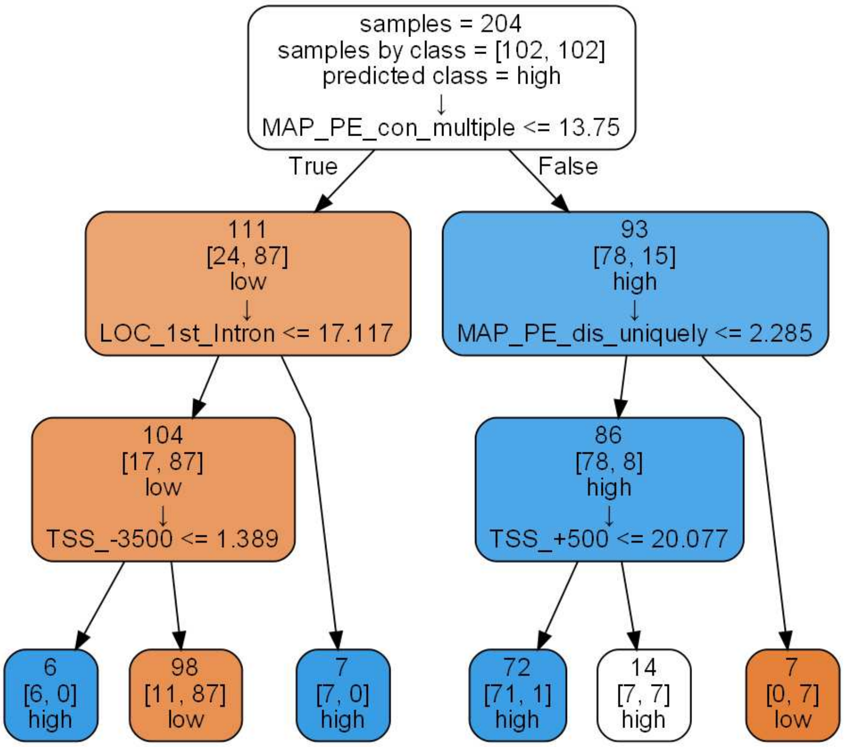

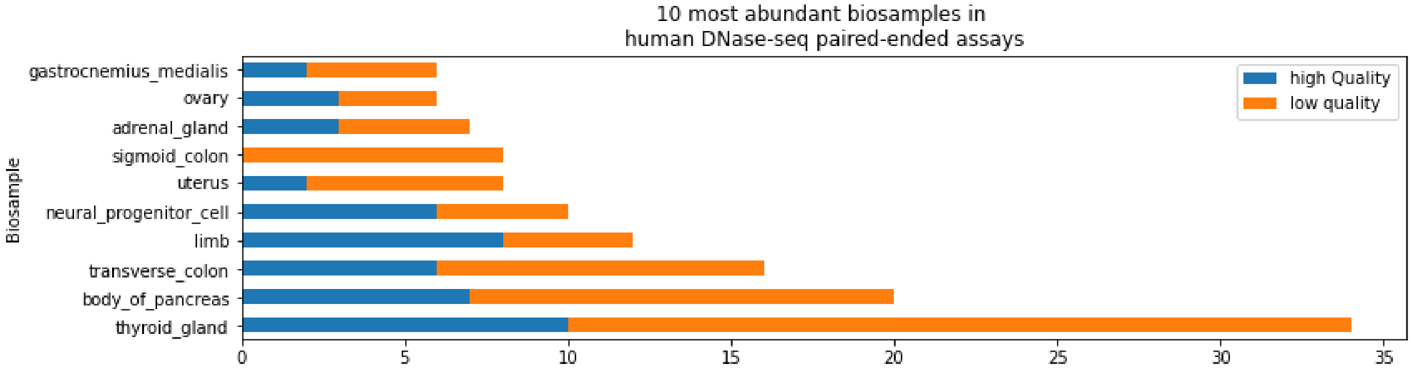

Metrics on training set:

- Accuracy: 0.91

High-quality files:

- Precision: 0.92
- Recall: 0.89
- F1-score: 0.91

Low-quality files

- Precision: 0.9
- Recall: 0.92
- F1-score: 0.91

Legend:

Nodes show data state and decision criteria to apply

Samples by class: [high-quality files, low-quality files]

Group 1

human single-ended RNA-seq

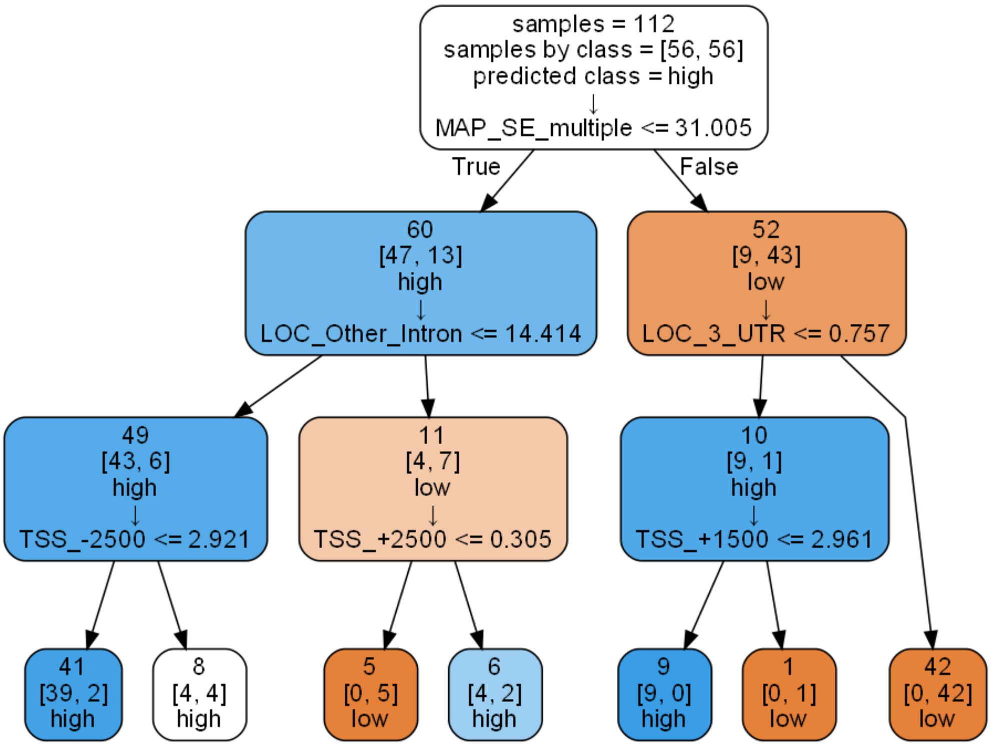

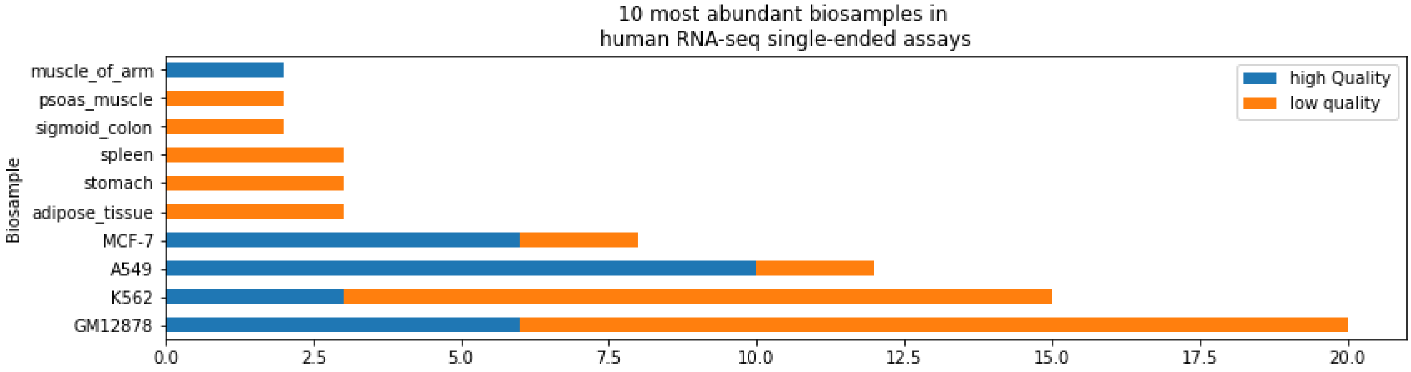

Metrics on training set:

- Accuracy: 0.93

High-quality files:

- Precision: 0.88
- Recall: 1.0
- F1-score: 0.93

Low-quality files

- Precision: 1.0
- Recall: 0.86
- F1-score: 0.92

Legend:

Nodes show data state and decision criteria to apply

Samples by class: [high-quality files, low-quality files]

Group 1

mouse single-ended ChIP-seq

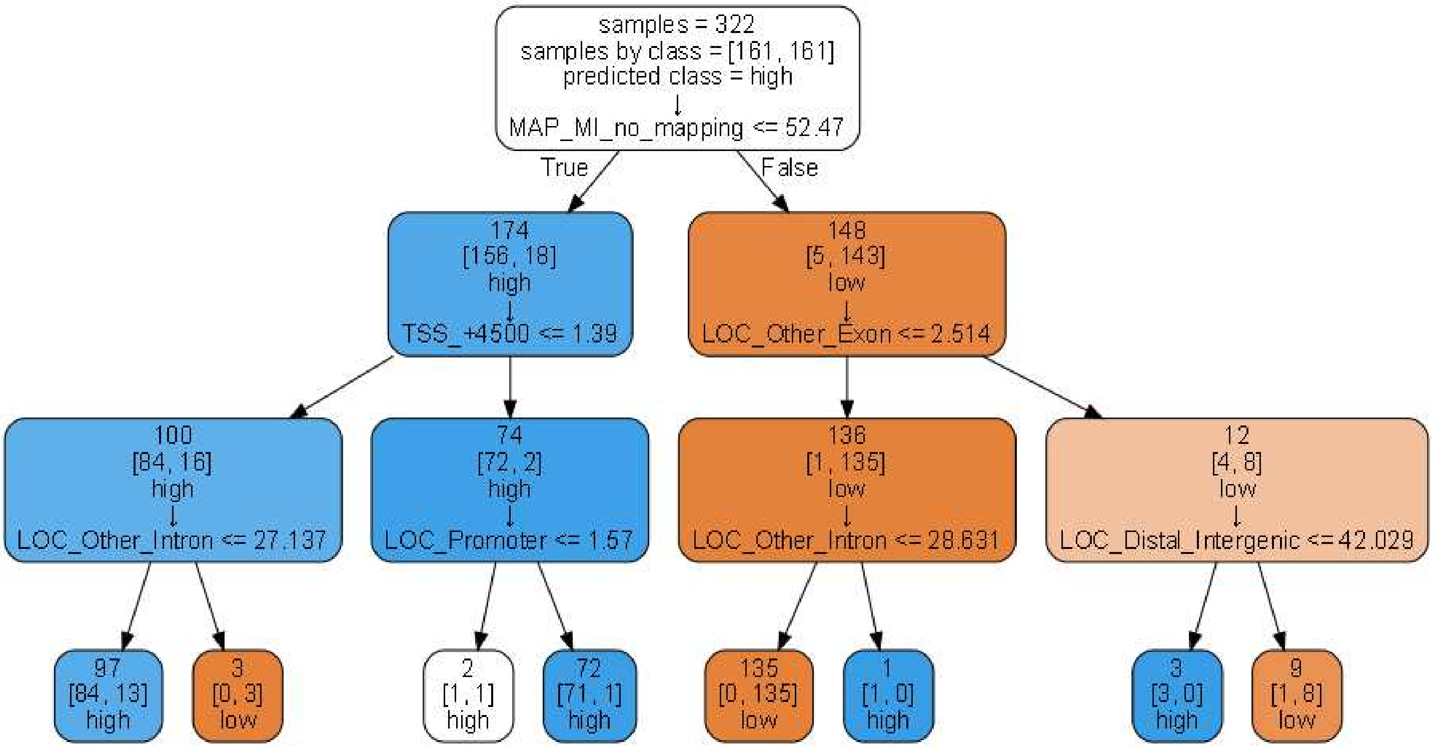

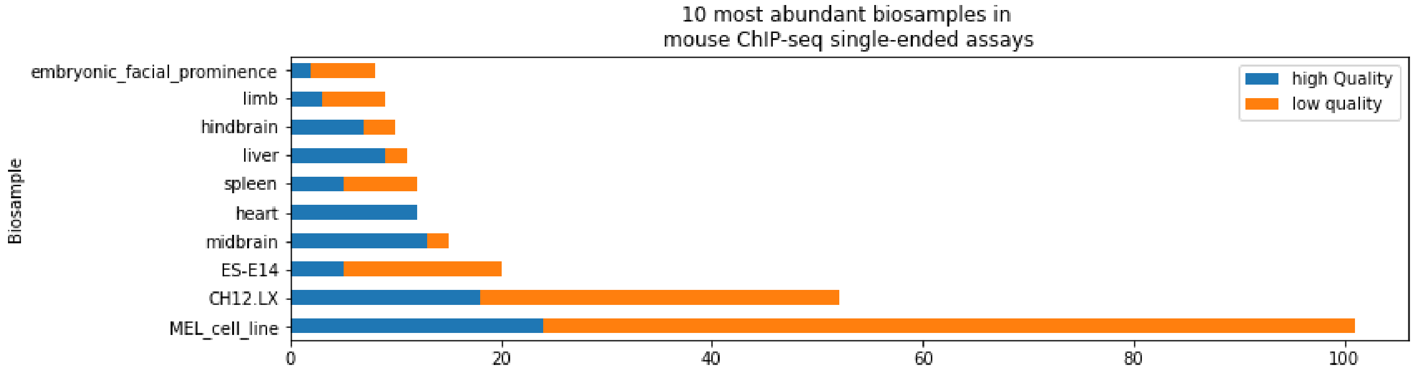

Metrics on training set:

- Accuracy:	0.95
High-quality files:	
- Precision:	0.91
- Recall:	0.99
- F1-score:	0.95
Low-quality files	
- Precision:	0.99
- Recall:	0.91
- F1-score:	0.95

Legend:
Nodes show data state and decision criteria to apply
Samples by class: [high-quality files, low-quality files]

Group 1

mouse paired-ended DNase-seq

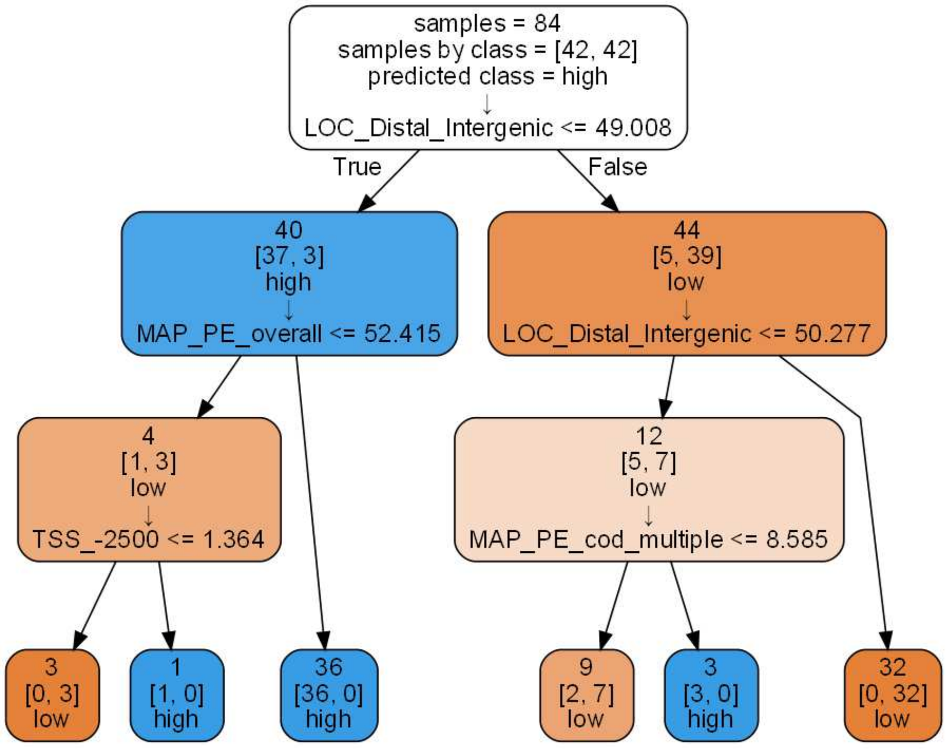

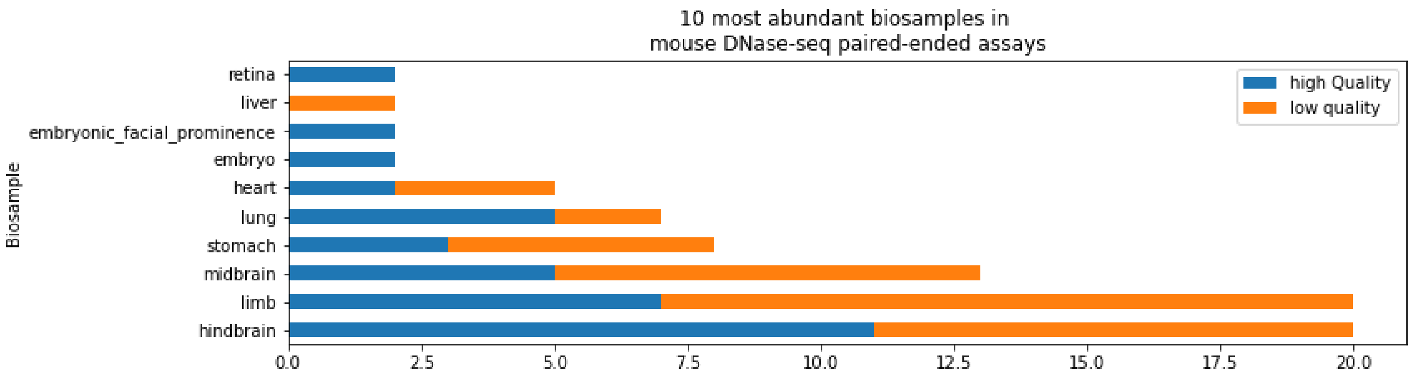

Metrics on training set:

- Accuracy: 0.98

High-quality files:

- Precision: 1.0
- Recall: 0.95
- F1-score: 0.98

Low-quality files

- Precision: 0.95
- Recall: 1.0
- F1-score: 0.98

Legend:

Nodes show data state and decision criteria to apply

Samples by class: [high-quality files, low-quality files]

Group 2

human paired-ended ControlChIP-seq

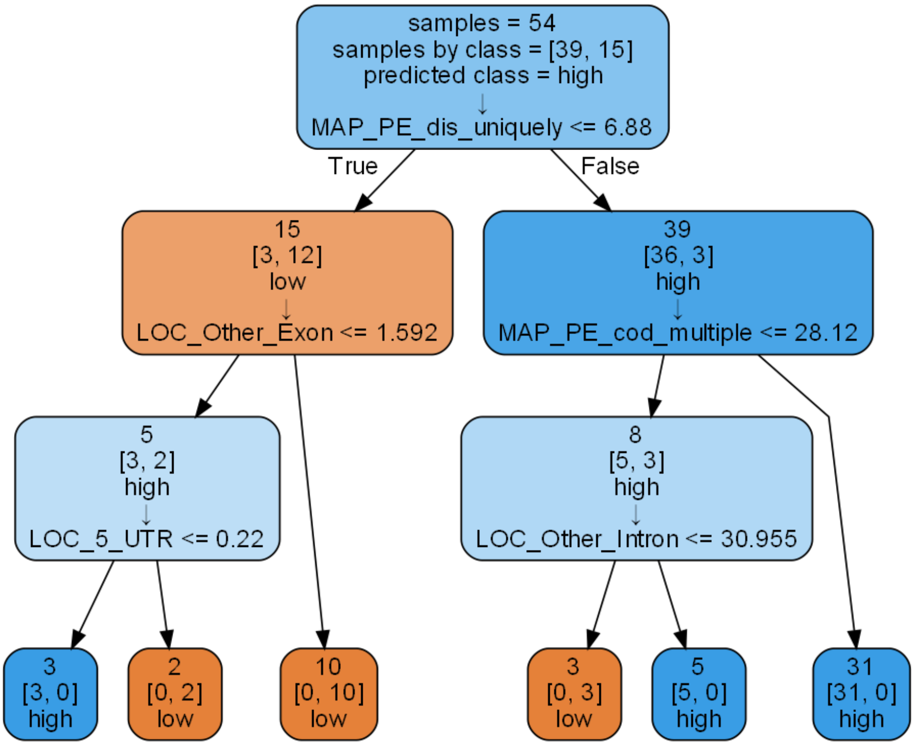

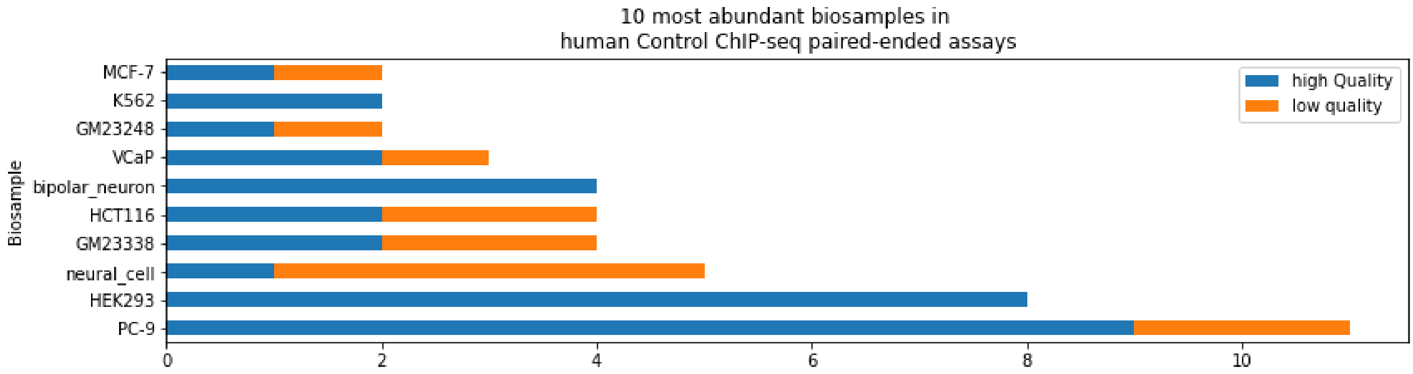

Metrics on training set:

- Accuracy:	1.0
High-quality files:	
- Precision:	1.0
- Recall:	1.0
- F1-score:	1.0
Low-quality files	
- Precision:	1.0
- Recall:	1.0
- F1-score:	1.0

Legend:

Nodes show data state and decision criteria to apply

Samples by class: [high-quality files, low-quality files]

Group 2

human single-ended ControlChIP-seq

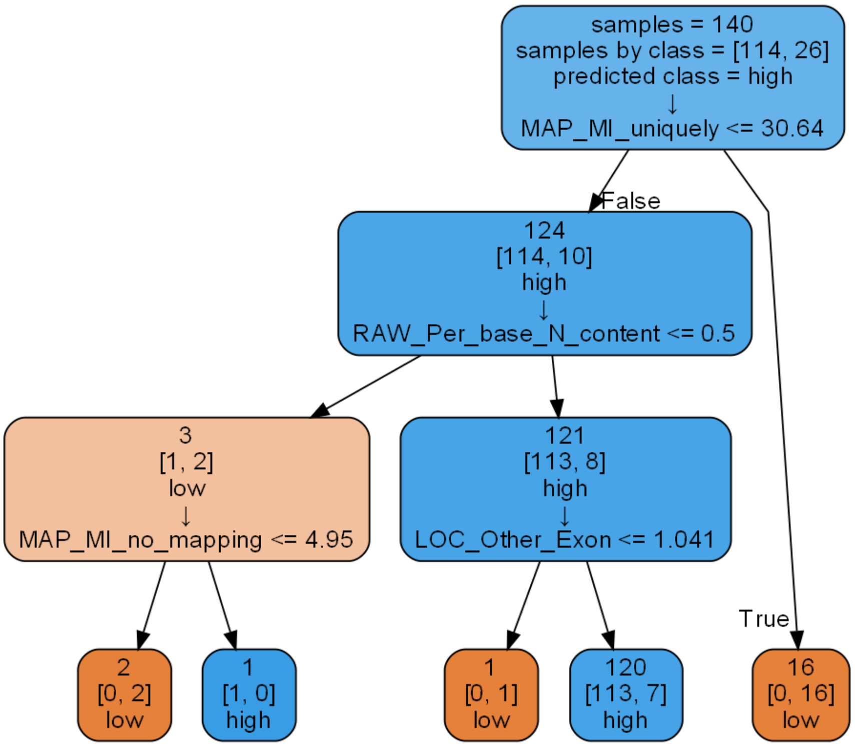

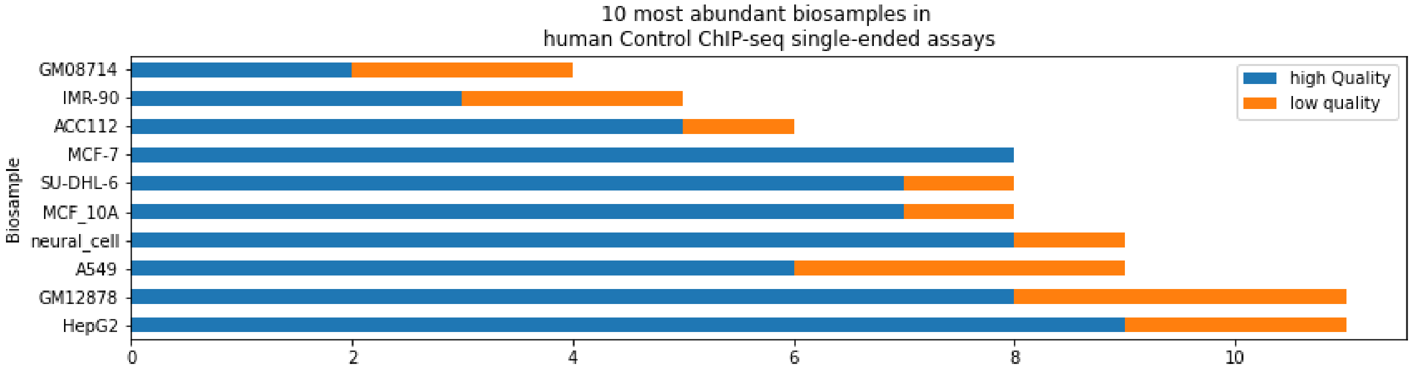

Metrics on training set:

- Accuracy: 0.95

High-quality files:

- Precision: 0.94
- Recall: 1.0
- F1-score: 0.97

Low-quality files

- Precision: 1.0
- Recall: 0.73
- F1-score: 0.84

Legend:

Nodes show data state and decision criteria to apply

Samples by class: [high-quality files, low-quality files]

Group 2

human paired-ended DNase-seq

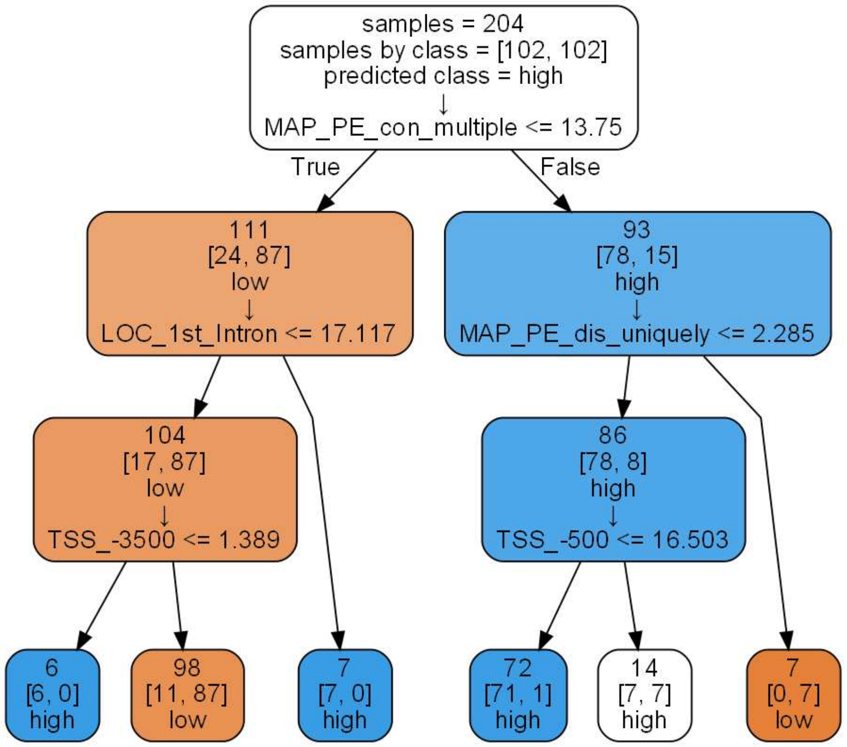

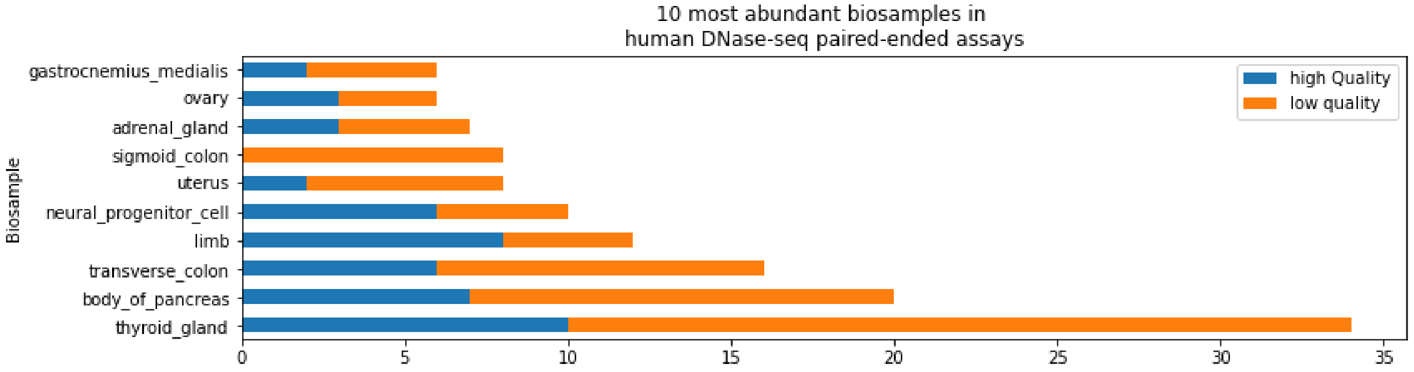

Metrics on training set:

- Accuracy: 0.91

High-quality files:

- Precision: 0.92
- Recall: 0.89
- F1-score: 0.91

Low-quality files

- Precision: 0.9
- Recall: 0.92
- F1-score: 0.91

Legend:

Nodes show data state and decision criteria to apply

Samples by class: [high-quality files, low-quality files]

Group 2

human paired-ended HistoneChIP-seq

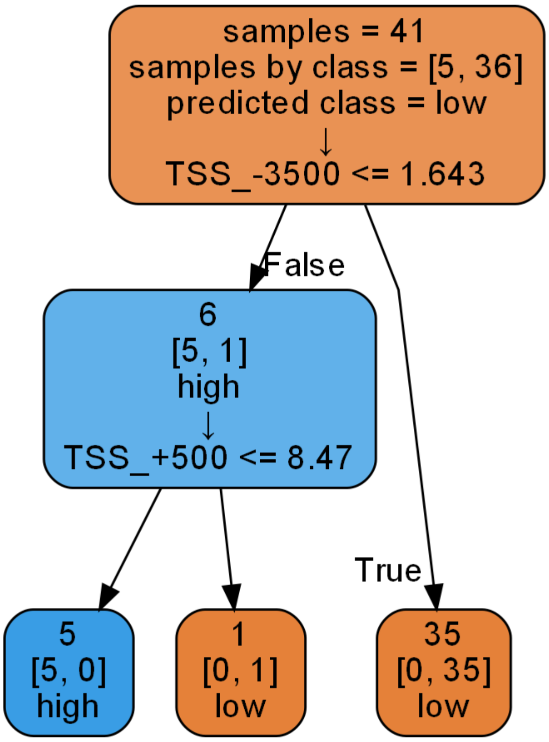

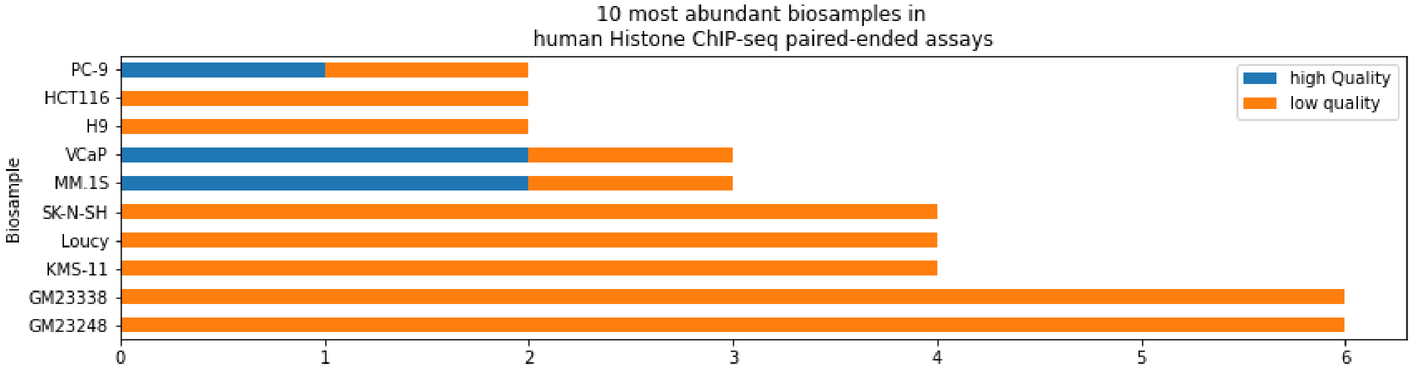

Metrics on training set:

- Accuracy: 1.0

High-quality files:

- Precision: 1.0
- Recall: 1.0
- F1-score: 1.0

Low-quality files

- Precision: 1.0
- Recall: 1.0
- F1-score: 1.0

Legend:

Nodes show data state and decision criteria to apply

Samples by class: [high-quality files, low-quality files]

Group 2

human single-ended HistoneChIP-seq

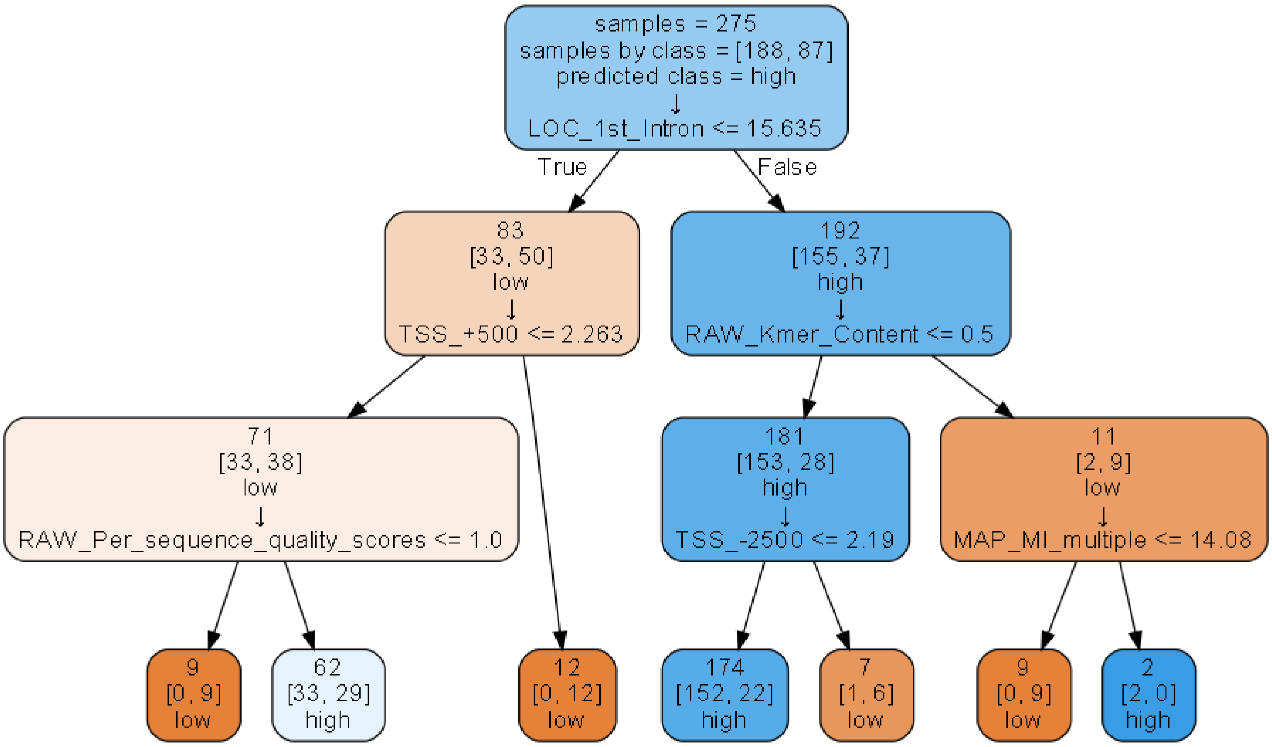

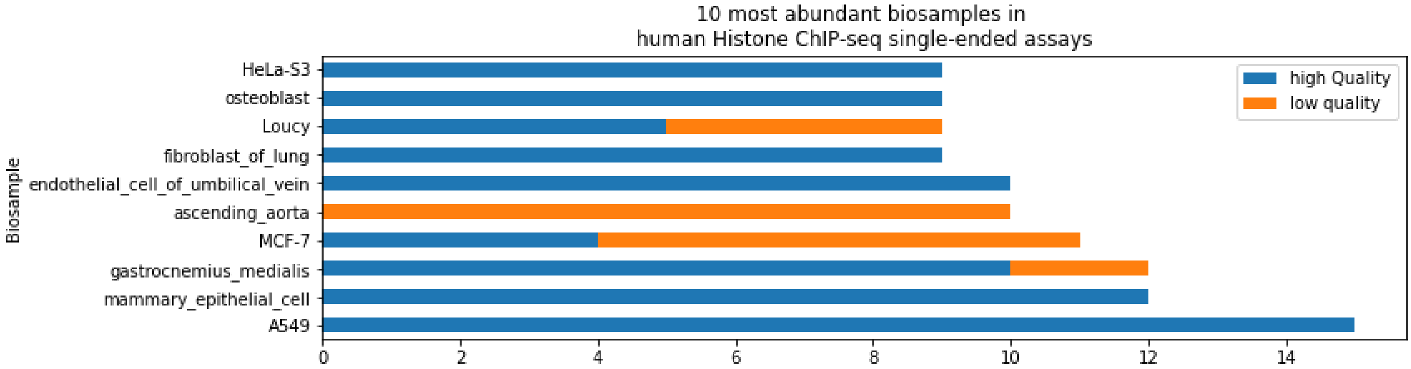

Metrics on training set:

- Accuracy: 0.81

High-quality files:

- Precision: 0.79
- Recall: 0.99
- F1-score: 0.88

Low-quality files

- Precision: 0.97
- Recall: 0.41
- F1-score: 0.58

Legend:

Nodes show data state and decision criteria to apply

Samples by class: [high-quality files, low-quality files]

Group 2

human single-ended polyAplusRNA-seq

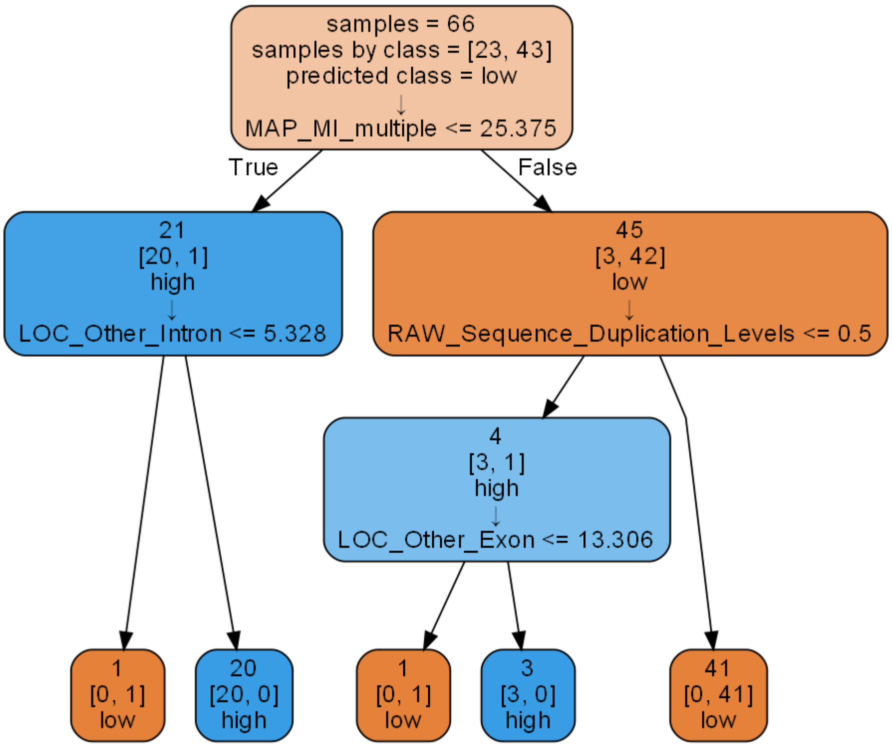

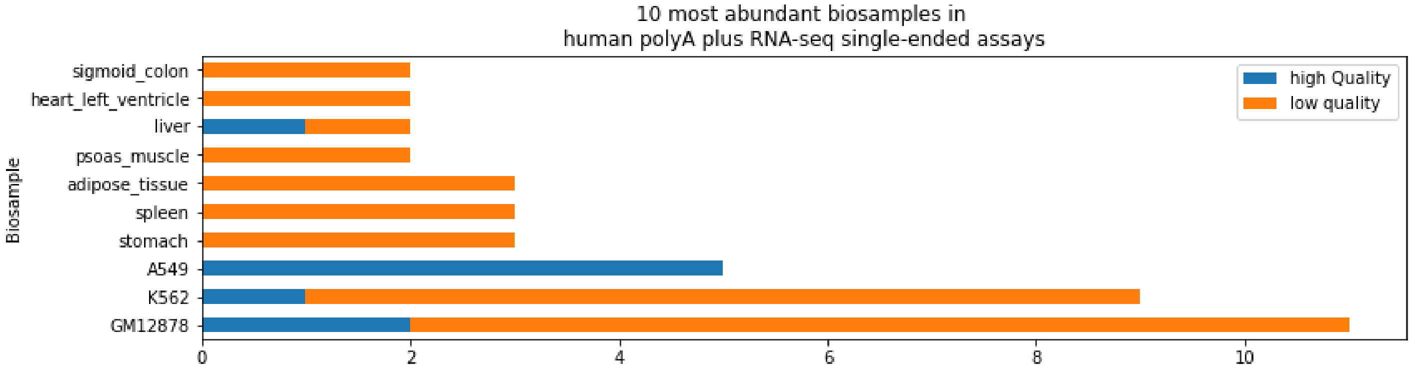

Metrics on training set:

- Accuracy: 1.0

High-quality files:

- Precision: 1.0
- Recall: 1.0
- F1-score: 1.0

Low-quality files

- Precision: 1.0
- Recall: 1.0
- F1-score: 1.0

Legend:

Nodes show data state and decision criteria to apply

Samples by class: [high-quality files, low-quality files]

Group 2

human single-ended smallRNA-seq

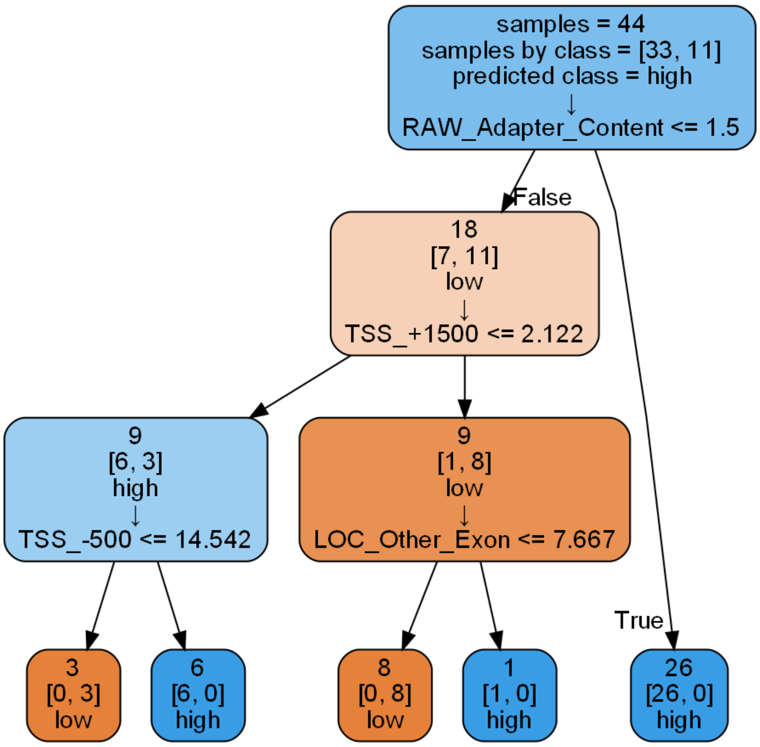

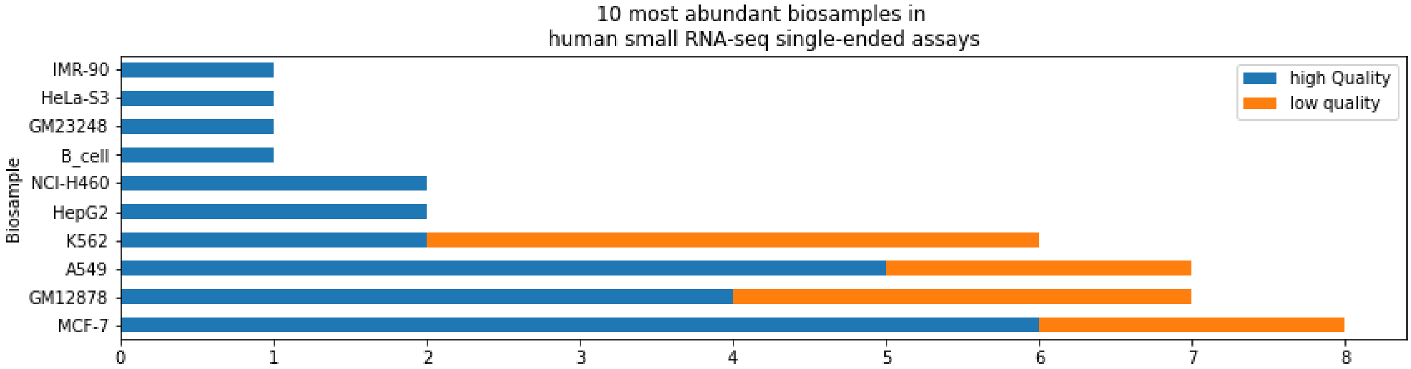

Metrics on training set:

- Accuracy: 1.0

High-quality files:

- Precision: 1.0
- Recall: 1.0
- F1-score: 1.0

Low-quality files

- Precision: 1.0
- Recall: 1.0
- F1-score: 1.0

Legend:

Nodes show data state and decision criteria to apply

Samples by class: [high-quality files, low-quality files]

Group 2

human paired-ended TFChIP-seq

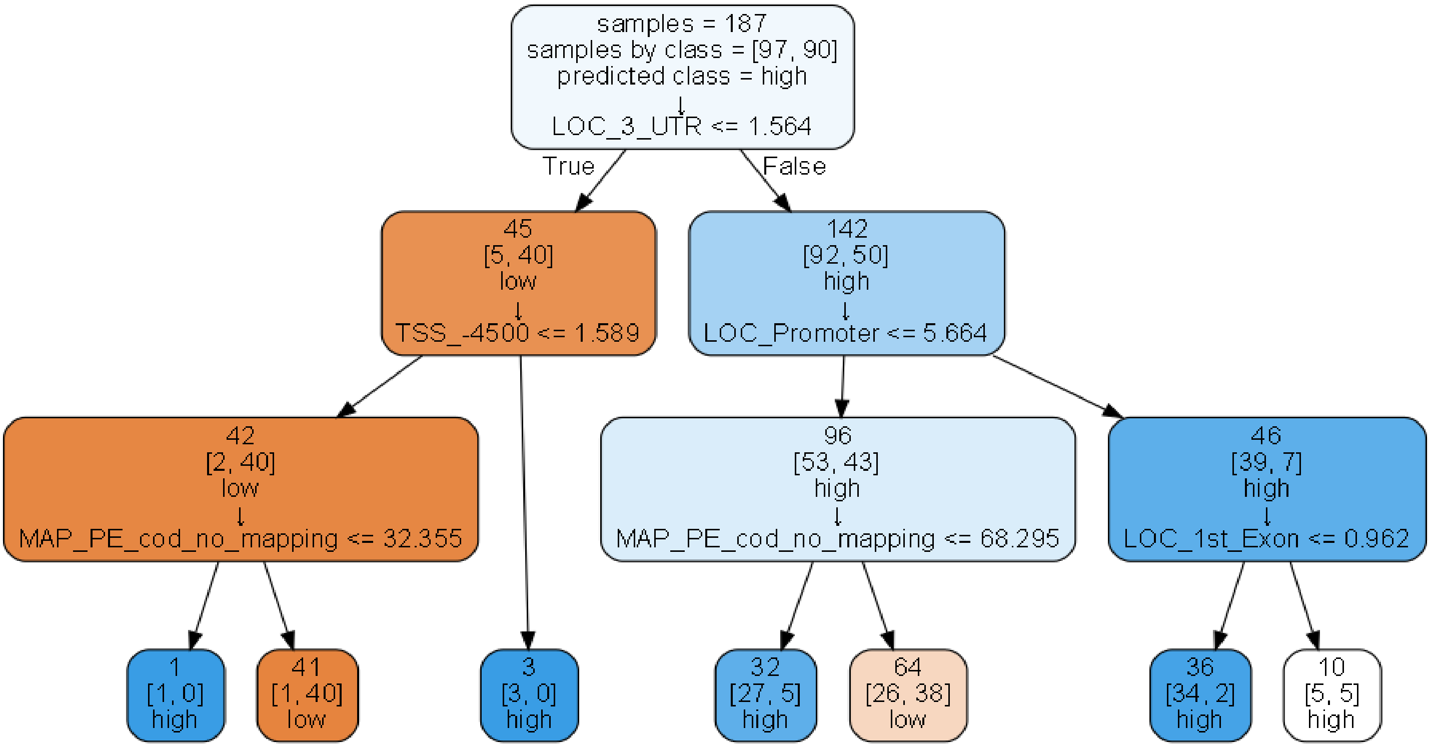

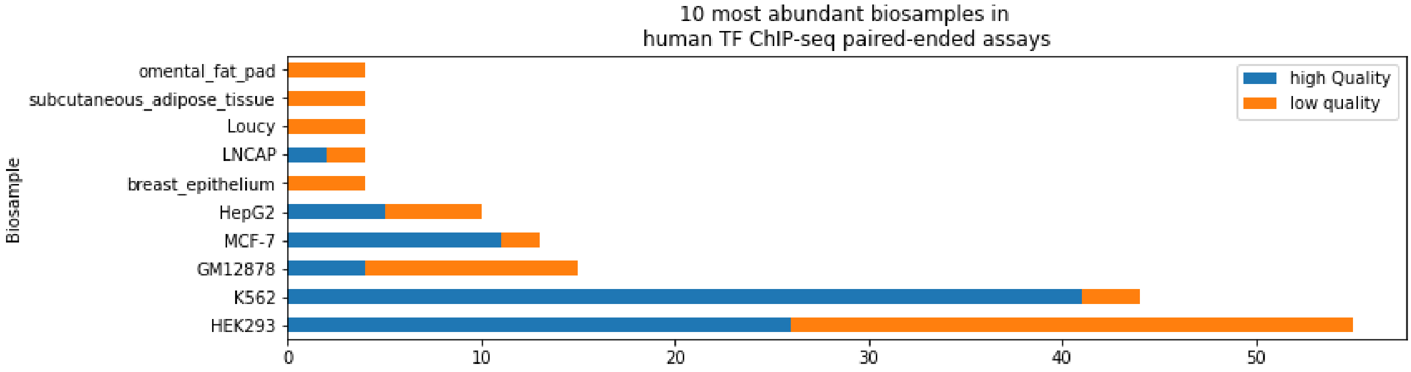

Metrics on training set:

- Accuracy: 0.79

High-quality files:

- Precision: 0.85
- Recall: 0.72
- F1-score: 0.78

Low-quality files

- Precision: 0.74
- Recall: 0.87
- F1-score: 0.8

Legend:

Nodes show data state and decision criteria to apply

Samples by class: [high-quality files, low-quality files]

Group 2
human single-ended TFChIP-seq

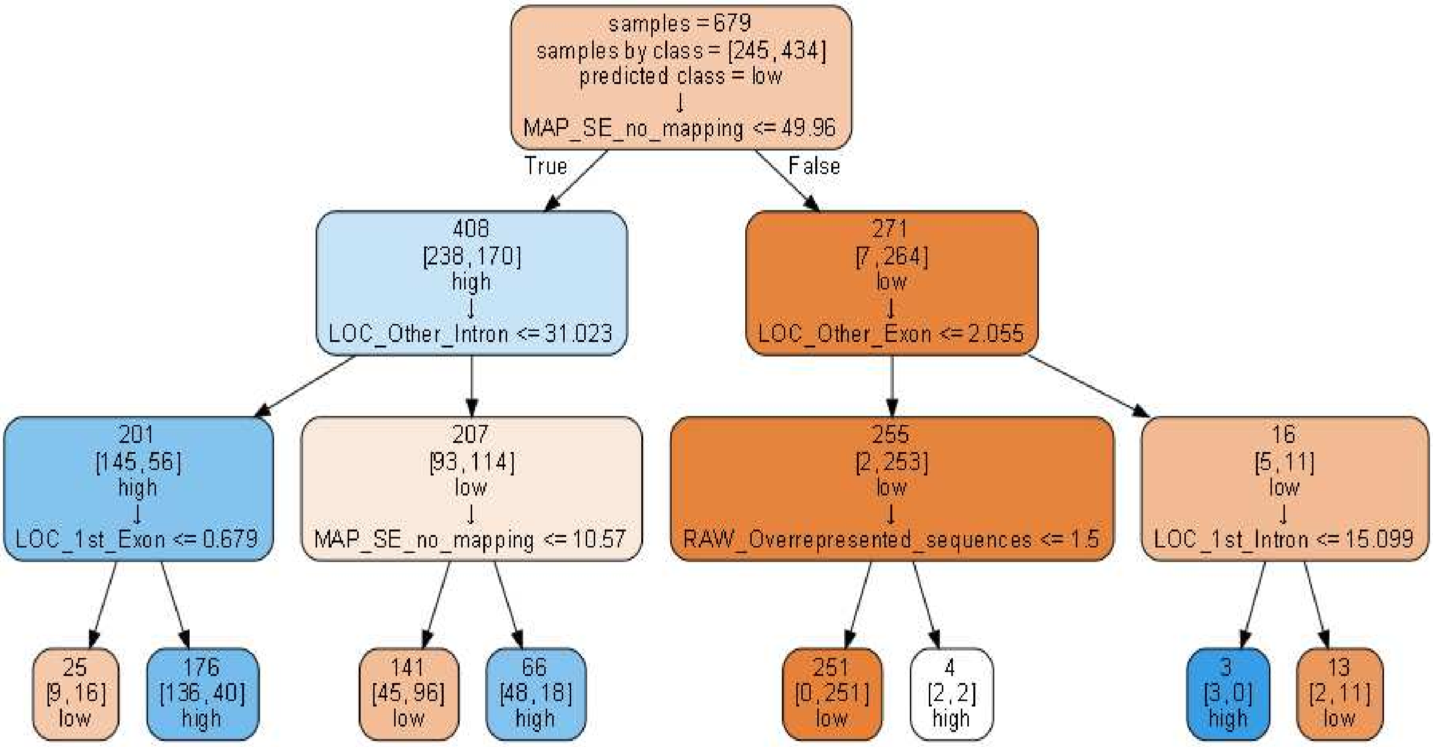

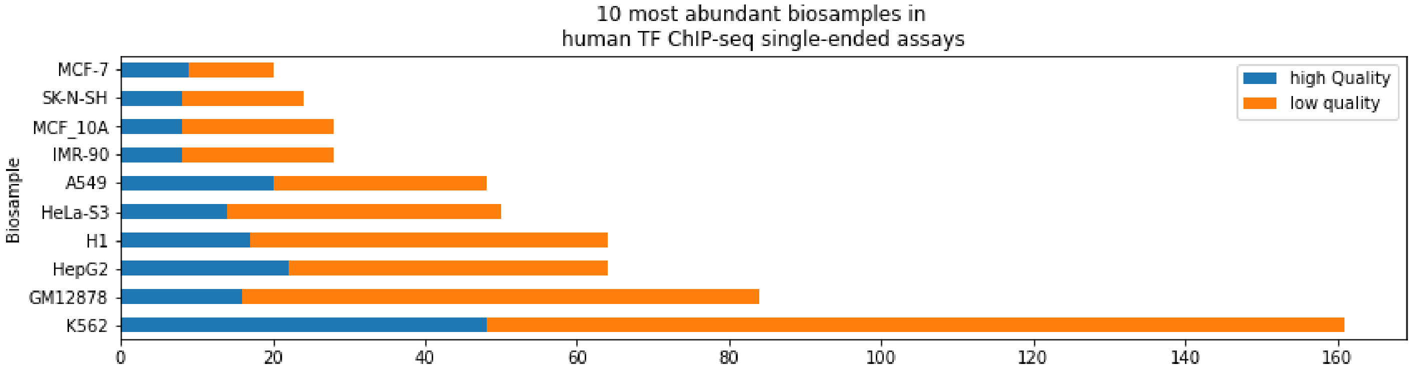

Metrics on training set:
- Accuracy: 0.83

High-quality files:
- Precision: 0.76
- Recall: 0.77
- F1-score: 0.77

Low-quality files
- Precision: 0.87
- Recall: 0.86
- F1-score: 0.87

Legend:
Nodes show data state and decision criteria to apply
Samples by class: [high-quality files, low-quality files]

Group 2

mouse single-ended ControlChIP-seq

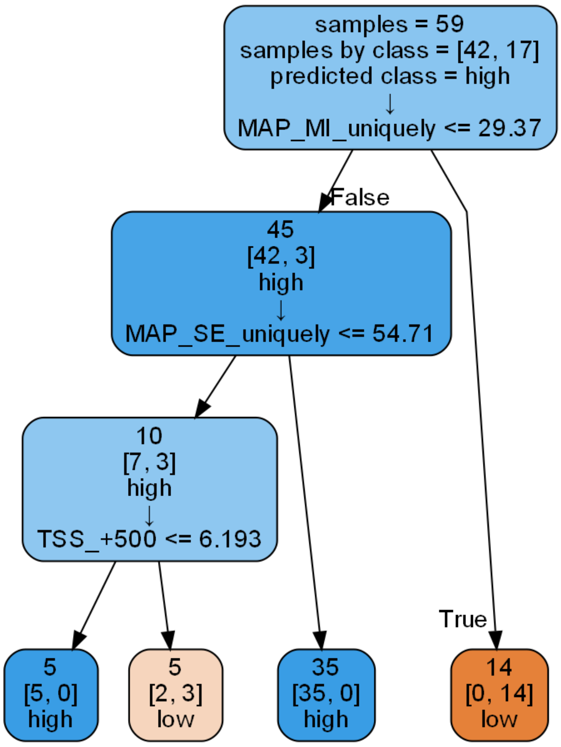

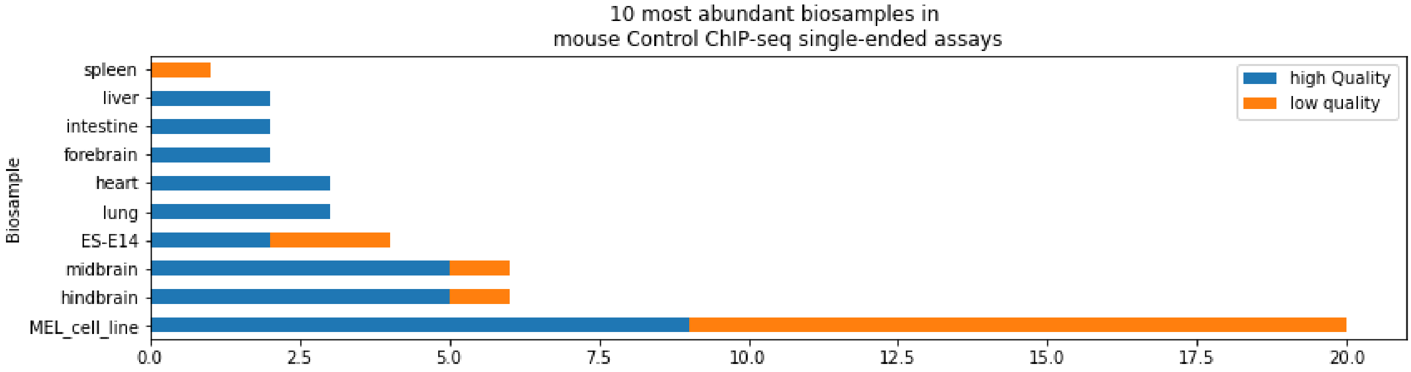

Metrics on training set:

- Accuracy:	0.97
High-quality files:	
- Precision:	1.0
- Recall:	0.95
- F1-score:	0.98
Low-quality files	
- Precision:	0.89
- Recall:	1.0
- F1-score:	0.94

Legend:
Nodes show data state and decision criteria to apply
Samples by class: [high-quality files, low-quality files]

Group 2

mouse paired-ended DNase-seq

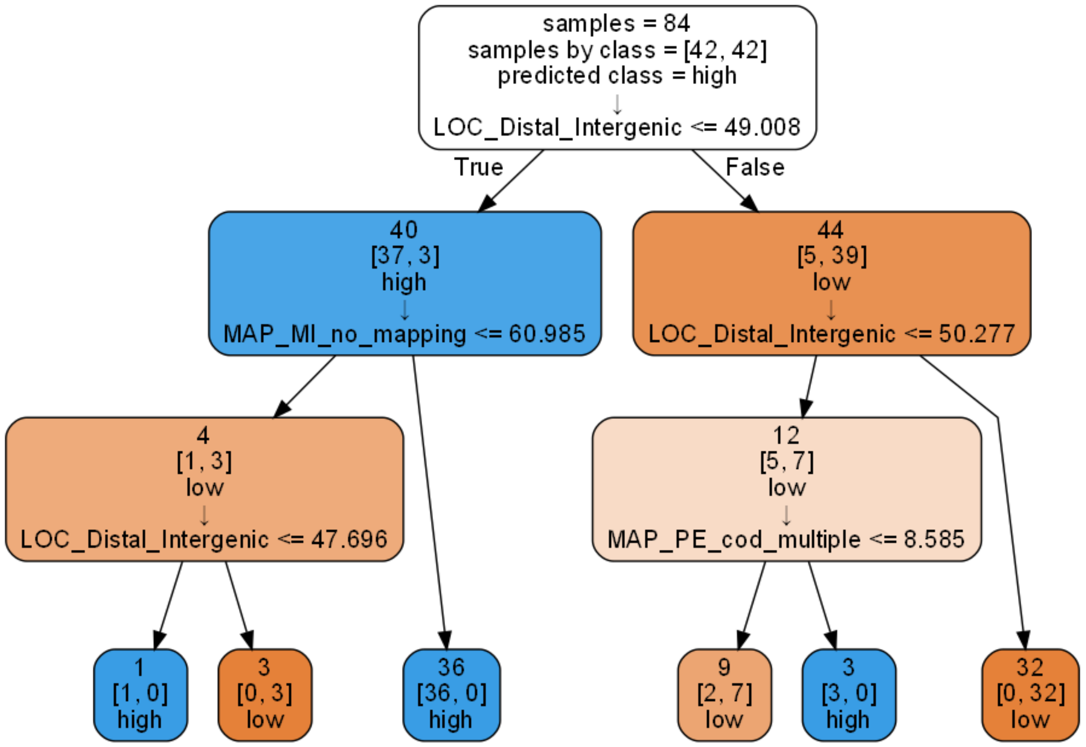

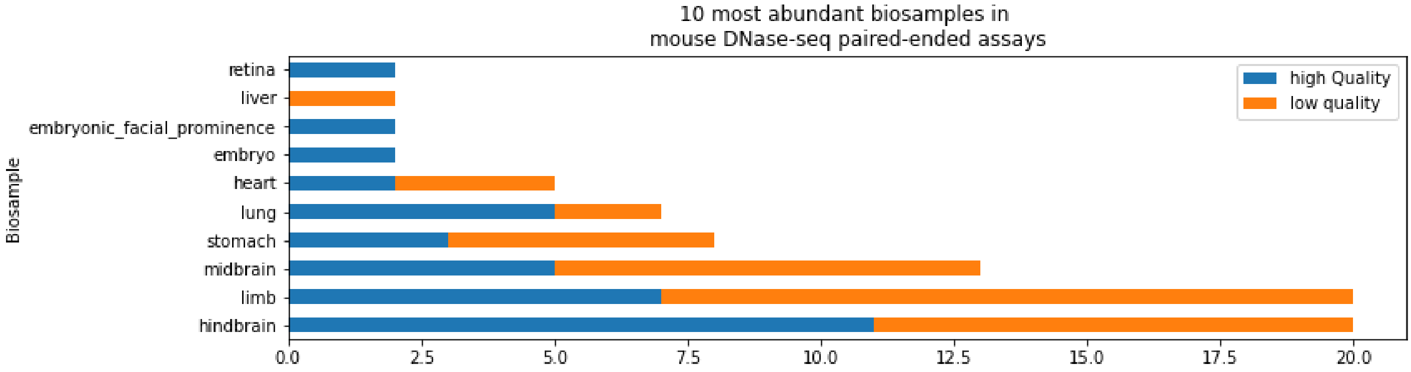

Metrics on training set:
- Accuracy: 0.98

High-quality files:
- Precision: 1.0
- Recall: 0.95
- F1-score: 0.98

Low-quality files
- Precision: 0.95
- Recall: 1.0
- F1-score: 0.98

Legend:
Nodes show data state and decision criteria to apply
Samples by class: [high-quality files, low-quality files]

Group 2

mouse single-ended HistoneChIP-seq

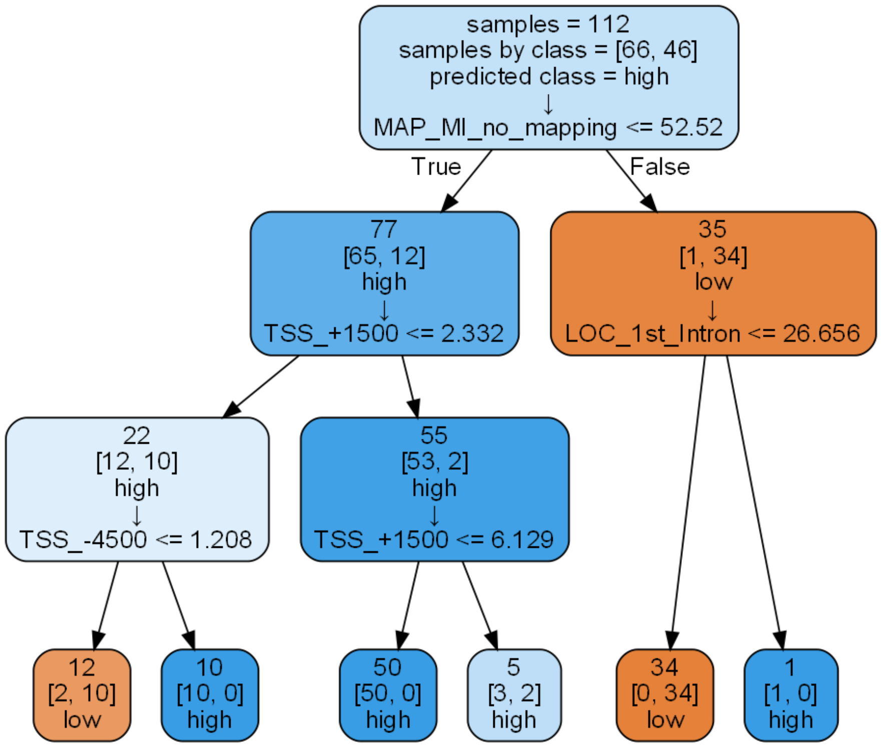

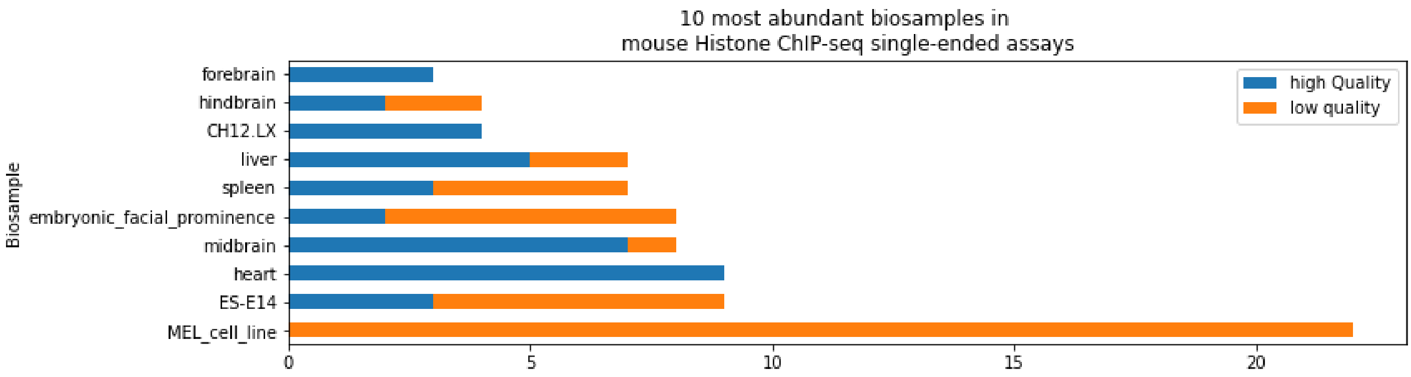

Metrics on training set:

- Accuracy: 0.96

High-quality files:

- Precision: 0.97
- Recall: 0.97
- F1-score: 0.97

Low-quality files

- Precision: 0.96
- Recall: 0.96
- F1-score: 0.96

Legend:

Nodes show data state and decision criteria to apply

Samples by class: [high-quality files, low-quality files]

Group 2

mouse single-ended TFChIP-seq

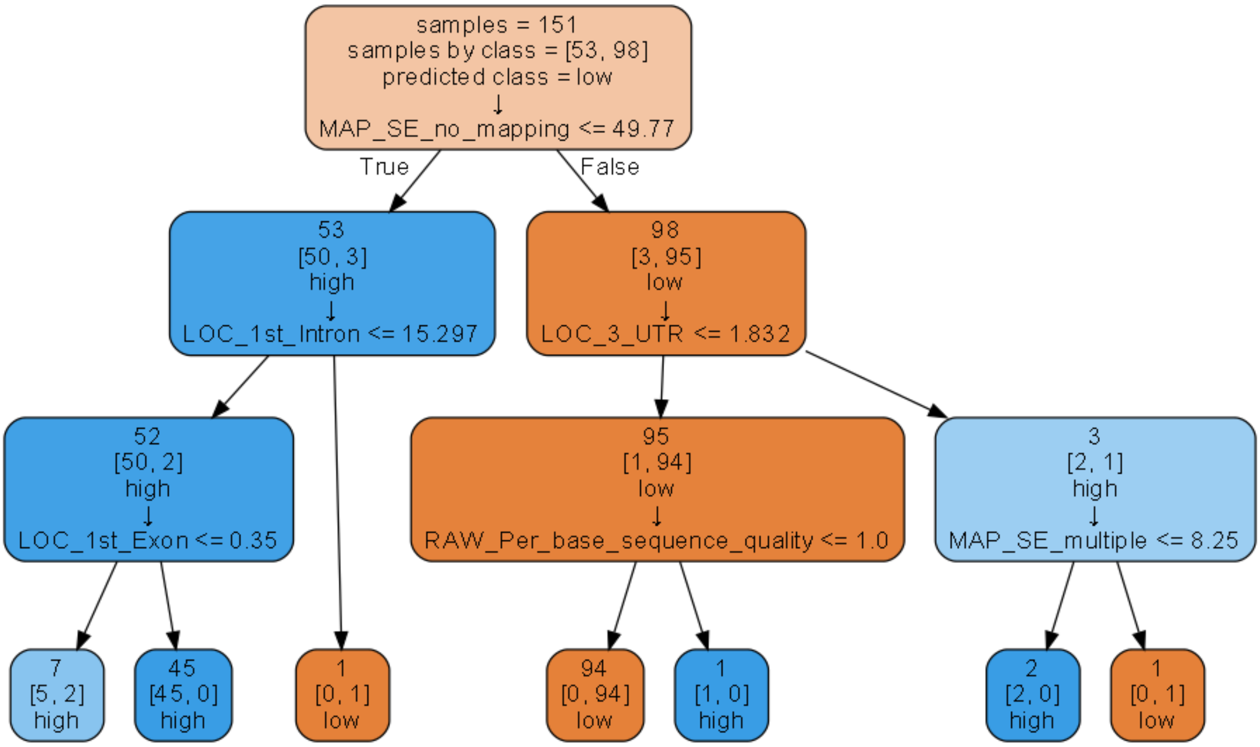

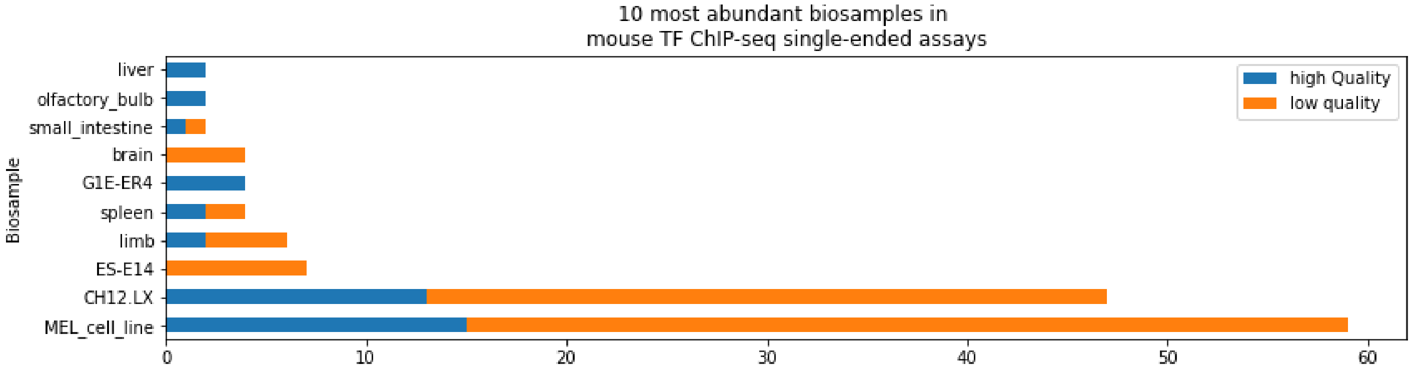

Metrics on training set:
- Accuracy: 0.99

High-quality files:
- Precision: 0.96
- Recall: 1.0
- F1-score: 0.98

Low-quality files
- Precision: 1.0
- Recall: 0.98
- F1-score: 0.99

Legend:
Nodes show data state and decision criteria to apply
Samples by class: [high-quality files, low-quality files]

Group A

human paired-ended ControlChIP-seq in PC-9

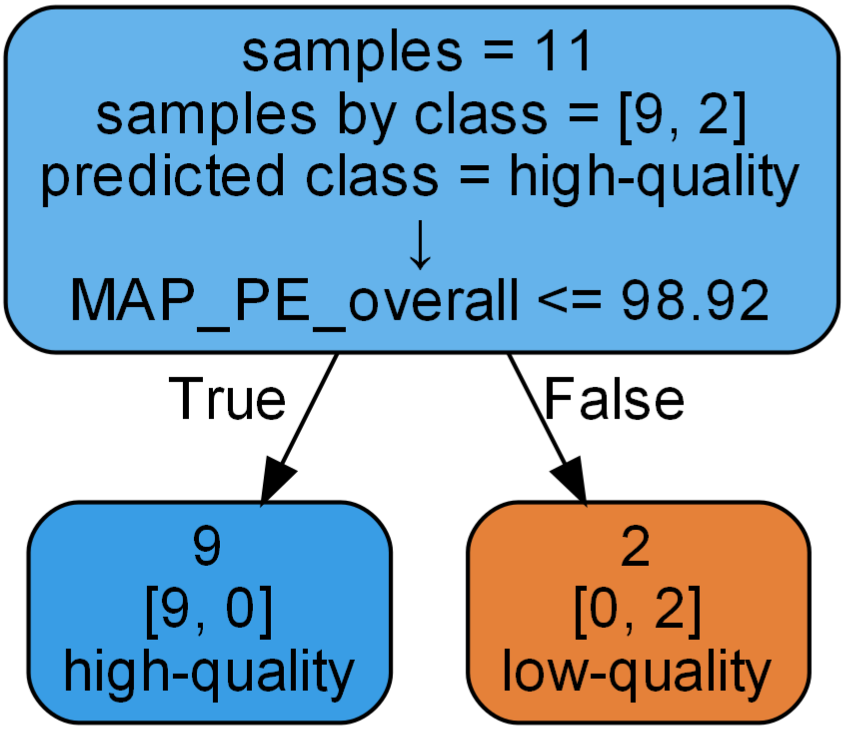

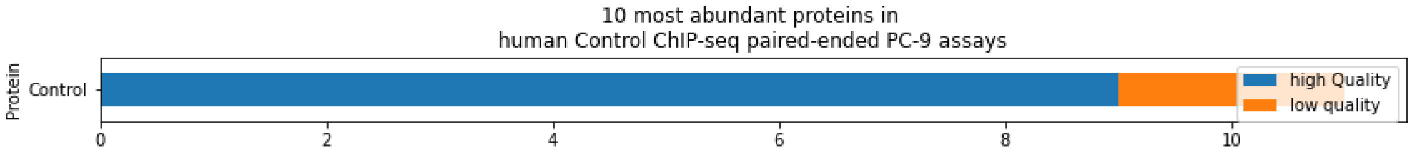

Metrics on training set:

- Accuracy: 1.0

High-quality files:

- Precision: 1.0
- Recall: 1.0
- F1-score: 1.0

Low-quality files

- Precision: 1.0
- Recall: 1.0
- F1-score: 1.0

Legend:

Nodes show data state and decision criteria to apply

Samples by class: [high-quality files, low-quality files]

Group A

human single-ended ControlChIP-seq in GM12878

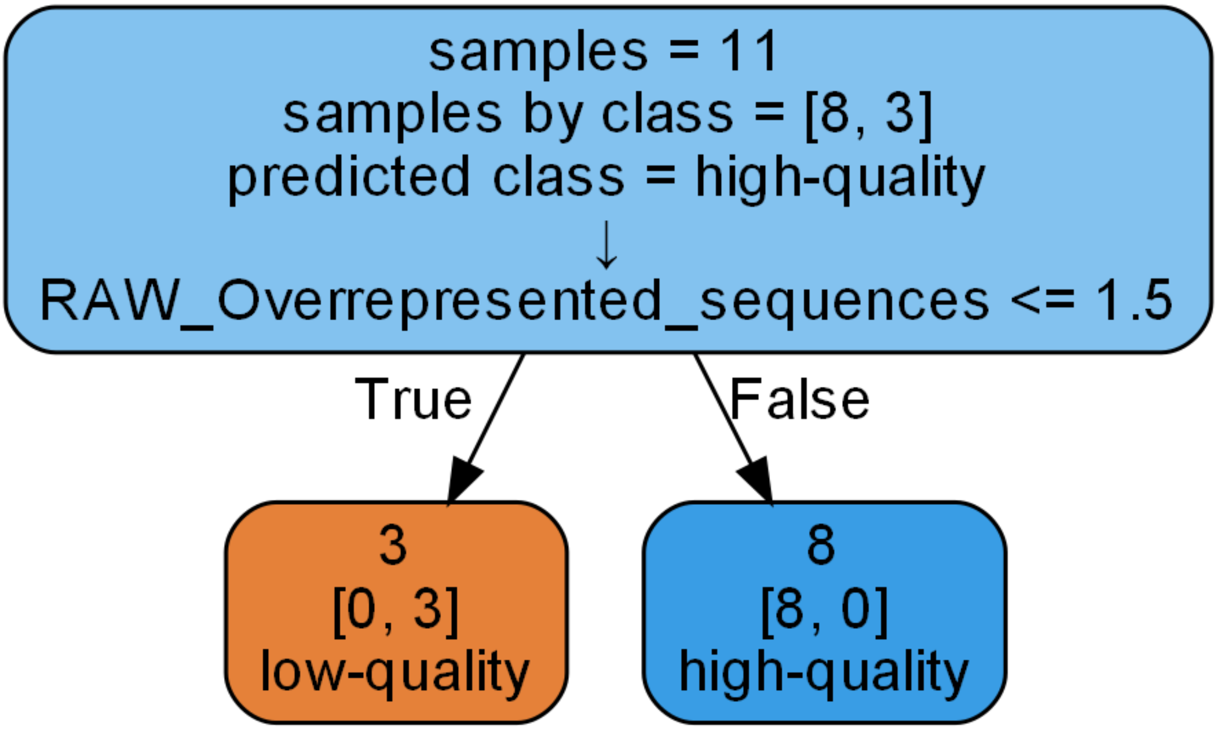

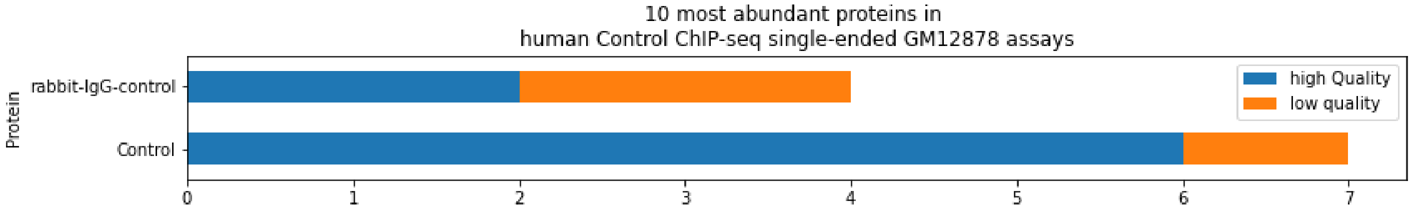

Metrics on training set:

- Accuracy: 1.0

High-quality files:

- Precision: 1.0
- Recall: 1.0
- F1-score: 1.0

Low-quality files

- Precision: 1.0
- Recall: 1.0
- F1-score: 1.0

Legend:

Nodes show data state and decision criteria to apply

Samples by class: [high-quality files, low-quality files]

Group A

human single-ended ControlChIP-seq in HepG2

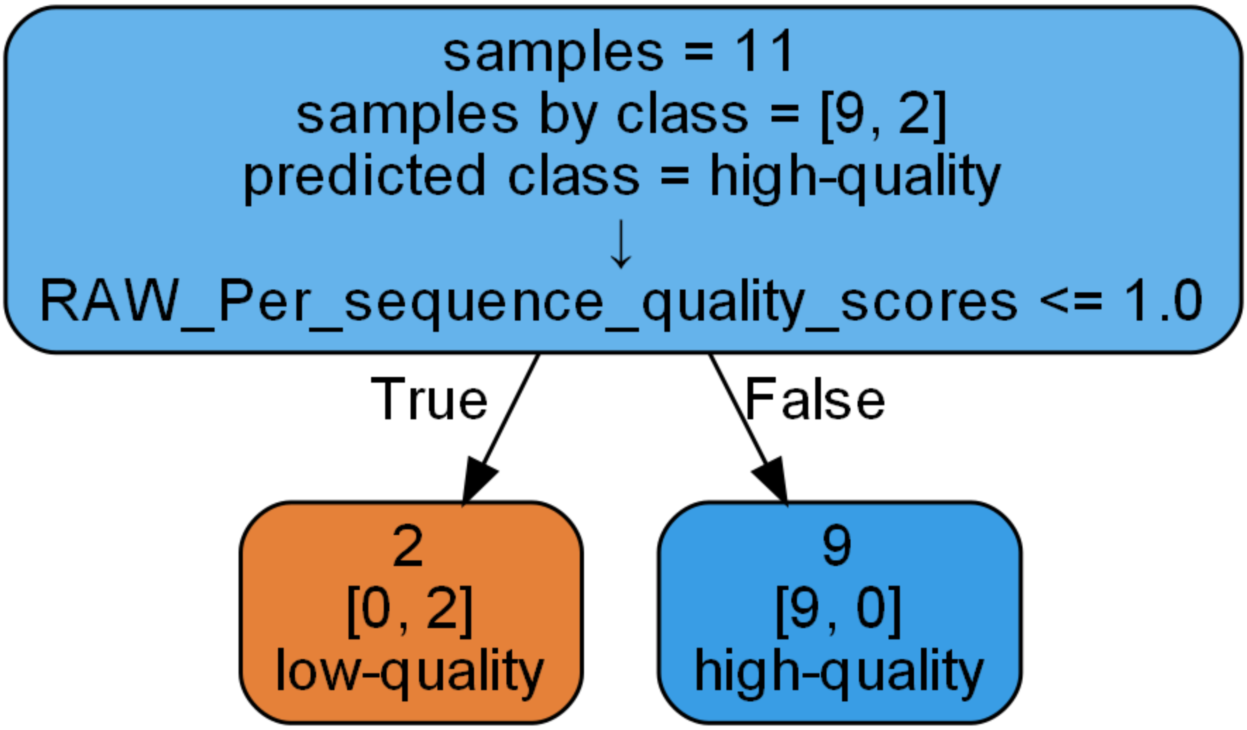

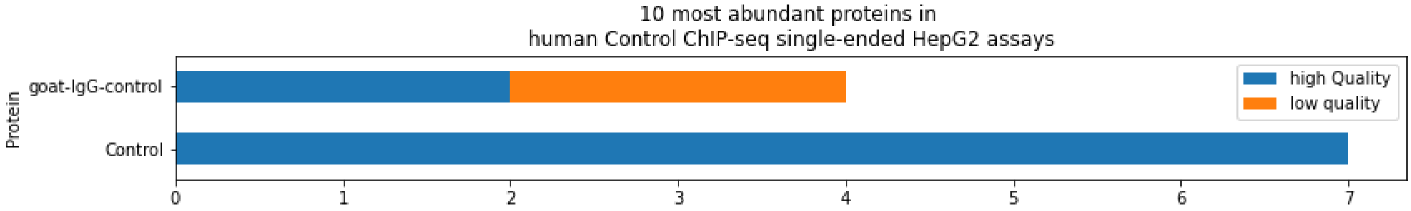

Metrics on training set:

- Accuracy: 1.0

High-quality files:

- Precision: 1.0
- Recall: 1.0
- F1-score: 1.0

Low-quality files

- Precision: 1.0
- Recall: 1.0
- F1-score: 1.0

Legend:

Nodes show data state and decision criteria to apply

Samples by class: [high-quality files, low-quality files]

Group A

human paired-ended DNase-seq in body_of_pancreas

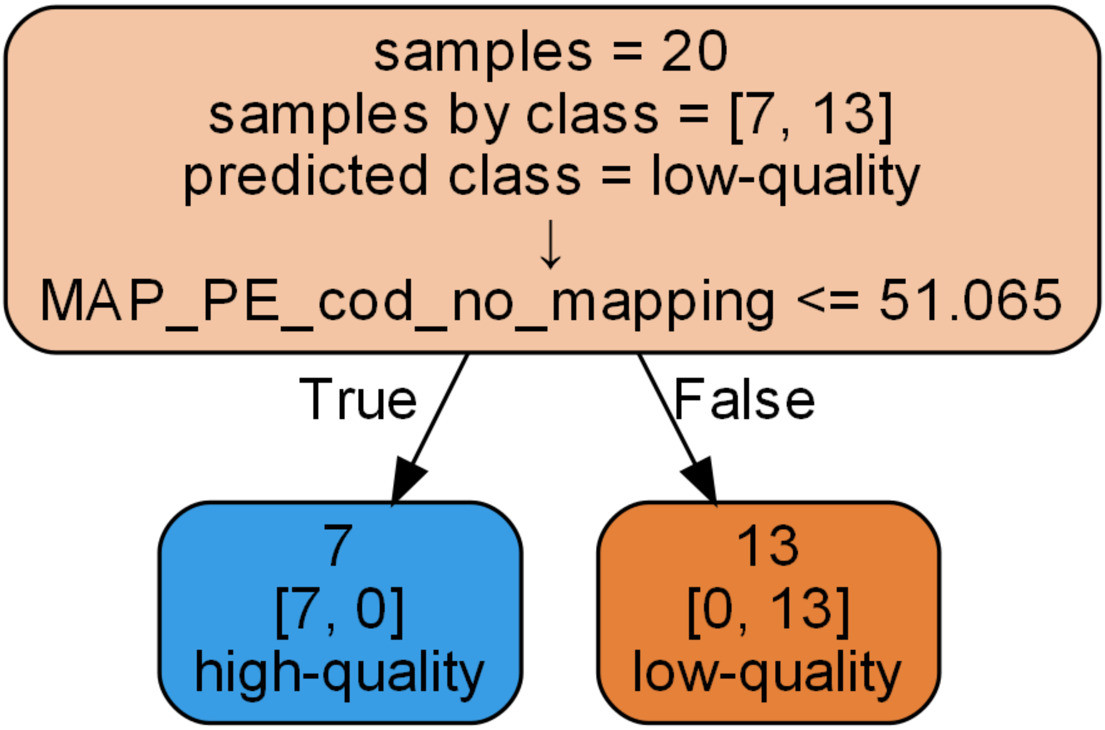

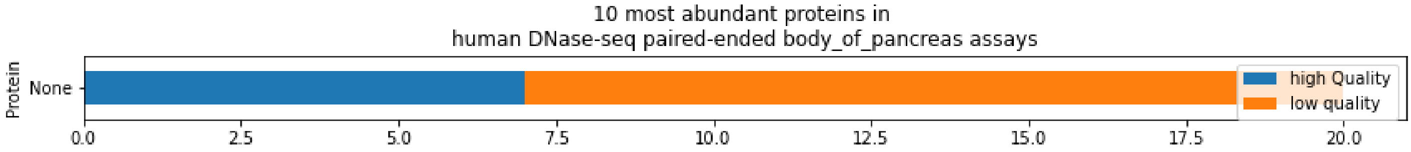

Metrics on training set:

- Accuracy: 1.0

High-quality files:

- Precision: 1.0
- Recall: 1.0
- F1-score: 1.0

Low-quality files

- Precision: 1.0
- Recall: 1.0
- F1-score: 1.0

Legend:

Nodes show data state and decision criteria to apply

Samples by class: [high-quality files, low-quality files]

Group A

human paired-ended DNase-seq in limb

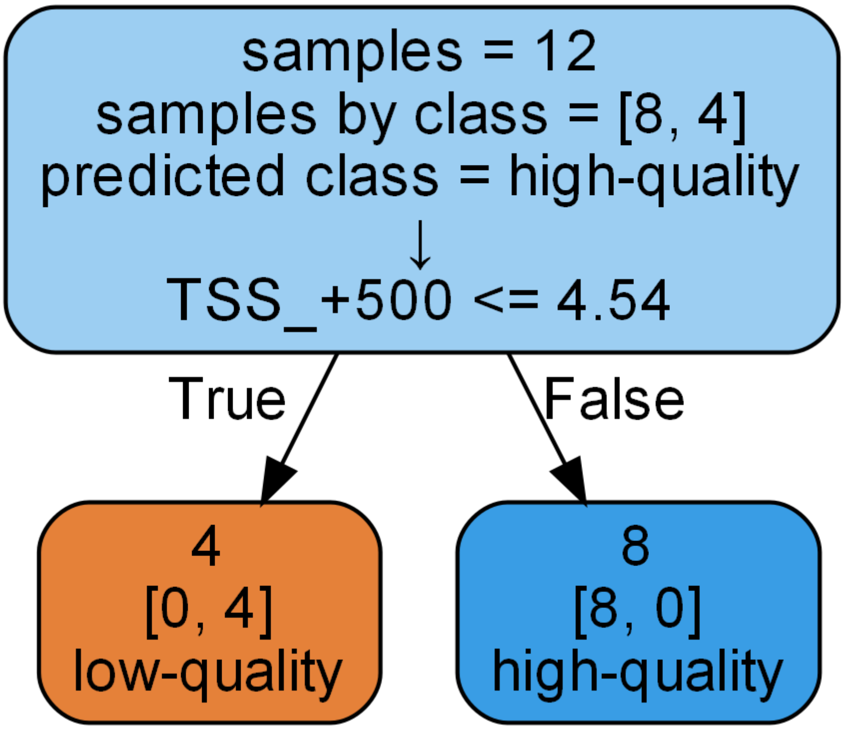

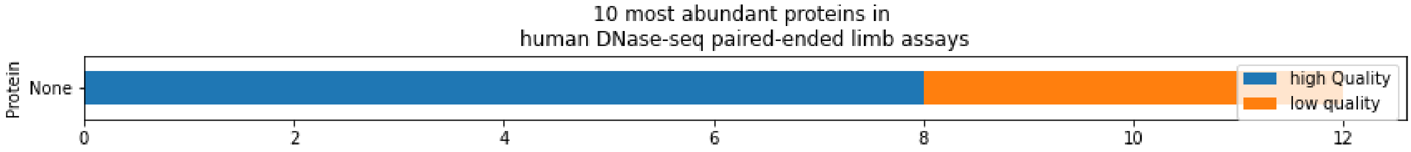

Metrics on training set:

- Accuracy: 1.0

High-quality files:

- Precision: 1.0
- Recall: 1.0
- F1-score: 1.0

Low-quality files

- Precision: 1.0
- Recall: 1.0
- F1-score: 1.0

Legend:

Nodes show data state and decision criteria to apply

Samples by class: [high-quality files, low-quality files]

Group A
human paired-ended DNase-seq in neural_progenitor_cell

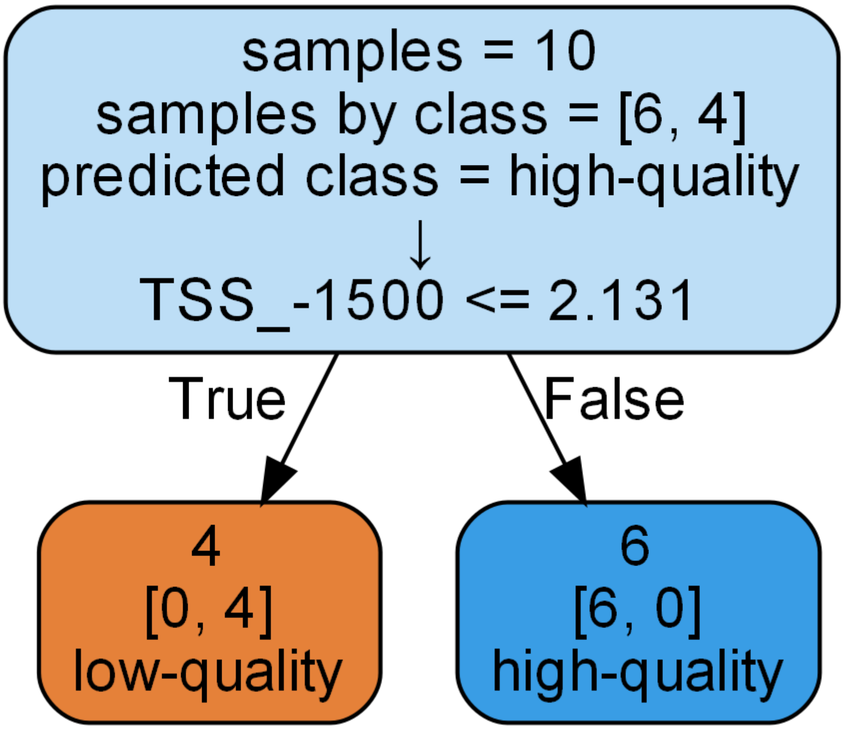

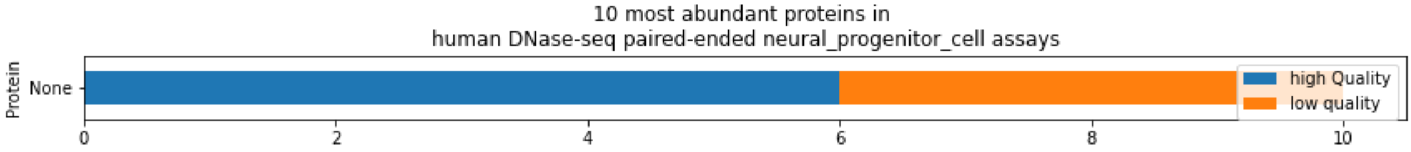

Metrics on training set:
- Accuracy: 1.0

High-quality files:
- Precision: 1.0
- Recall: 1.0
- F1-score: 1.0

Low-quality files
- Precision: 1.0
- Recall: 1.0
- F1-score: 1.0

Legend:
Nodes show data state and decision criteria to apply
Samples by class: [high-quality files, low-quality files]

Group A

human paired-ended DNase-seq in thyroid_gland

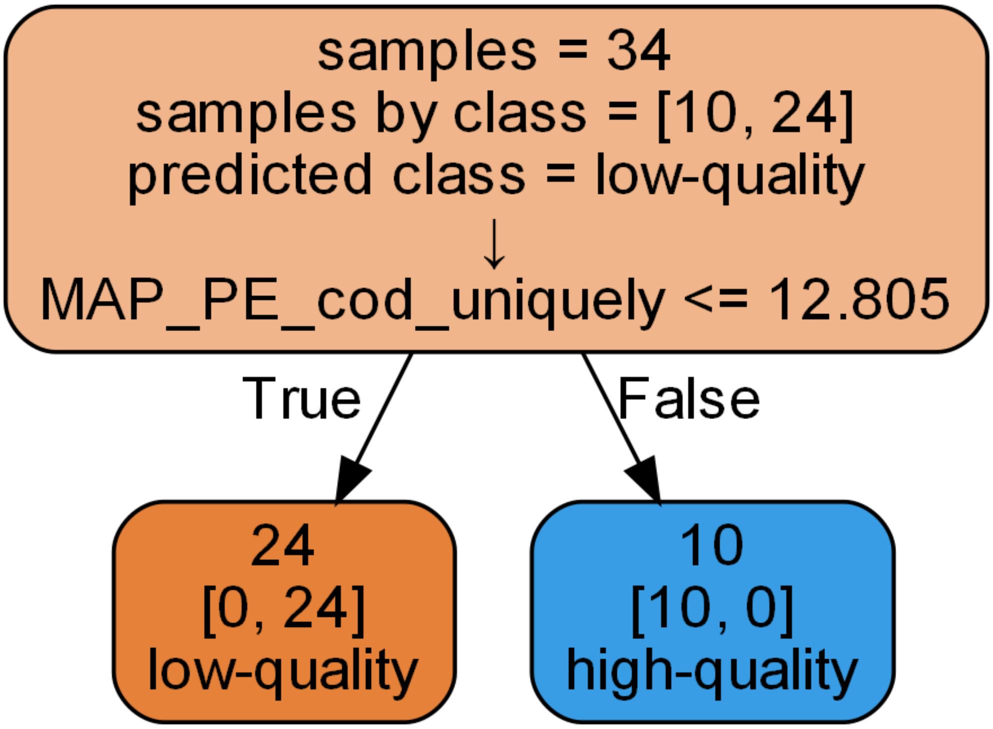

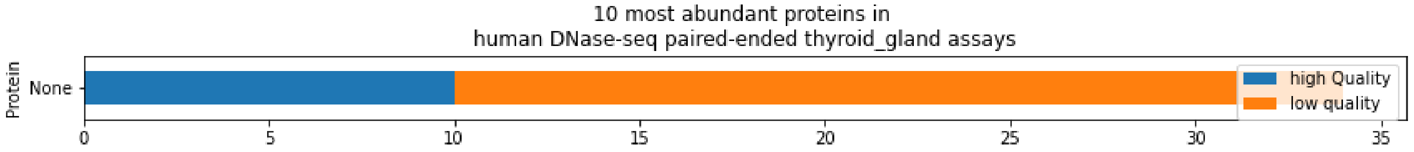

Metrics on training set:

- Accuracy:	1.0
High-quality files:	
- Precision:	1.0
- Recall:	1.0
- F1-score:	1.0
Low-quality files	
- Precision:	1.0
- Recall:	1.0
- F1-score:	1.0

Legend:
Nodes show data state and decision criteria to apply
Samples by class: [high-quality files, low-quality files]

Group A

human paired-ended DNase-seq in transverse_colon

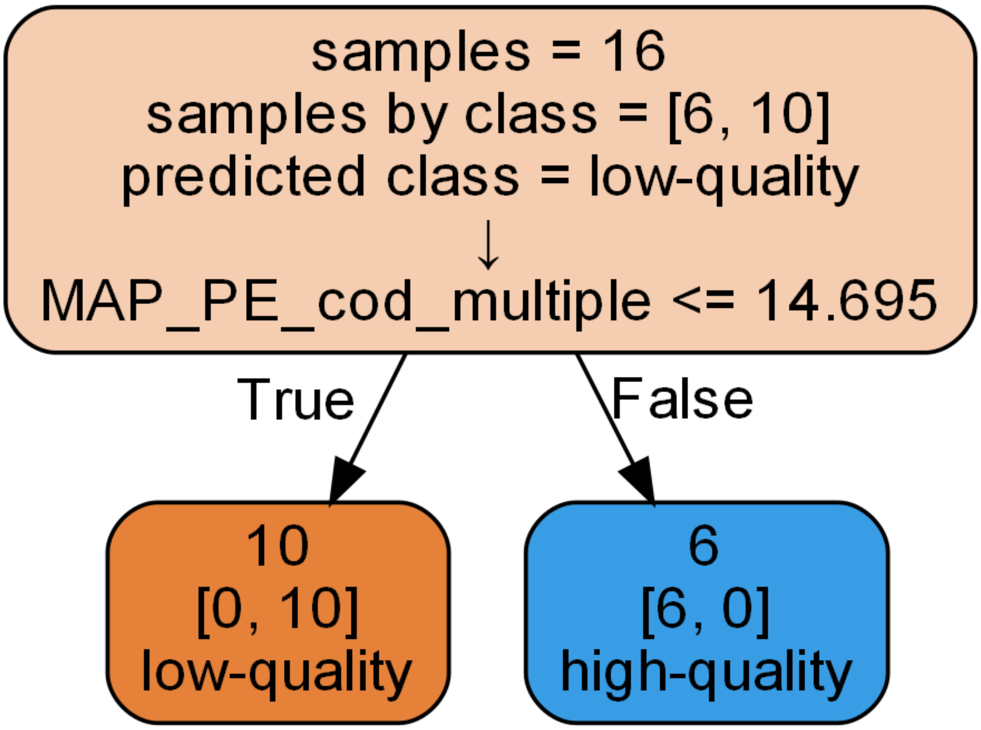

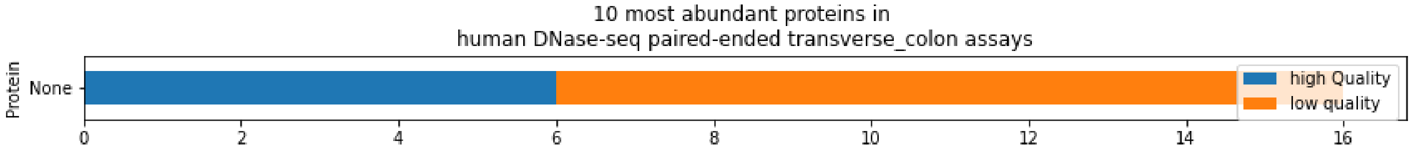

Metrics on training set:
- Accuracy: 1.0

High-quality files:
- Precision: 1.0
- Recall: 1.0
- F1-score: 1.0

Low-quality files
- Precision: 1.0
- Recall: 1.0
- F1-score: 1.0

Legend:
Nodes show data state and decision criteria to apply
Samples by class: [high-quality files, low-quality files]

Group A

human single-ended HistoneChIP-seq in gastrocnemius_medialis

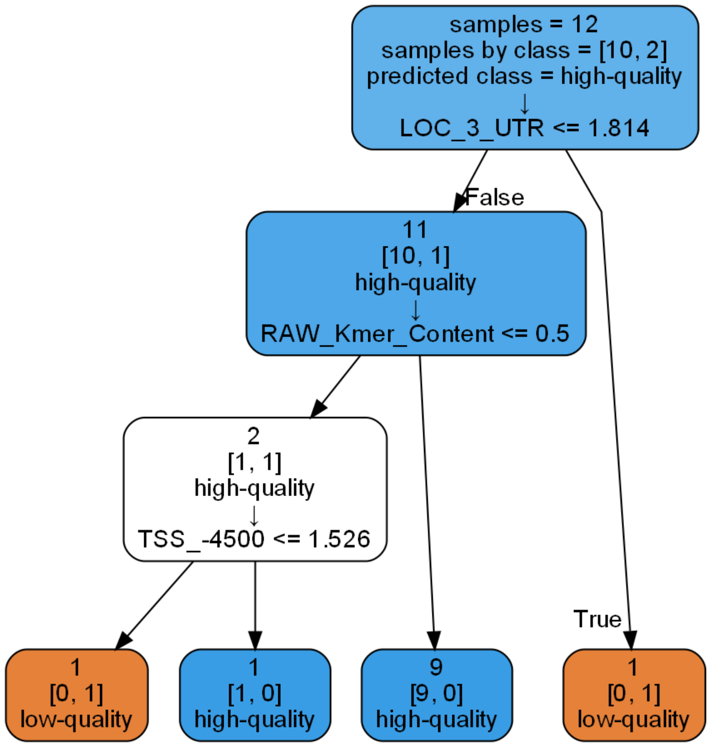

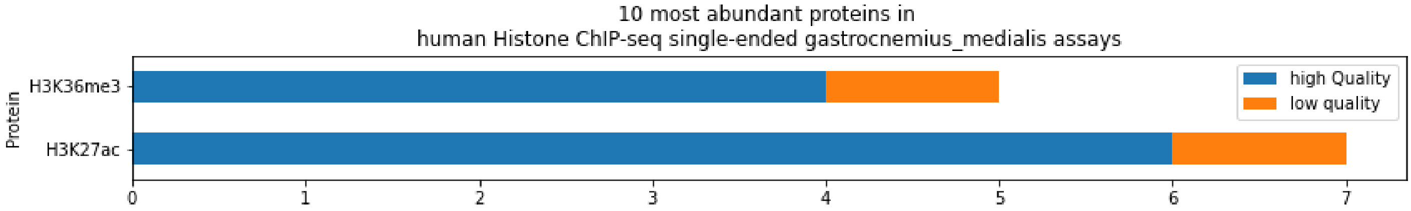

Metrics on training set:
- Accuracy: 1.0

High-quality files:
- Precision: 1.0
- Recall: 1.0
- F1-score: 1.0

Low-quality files
- Precision: 1.0
- Recall: 1.0
- F1-score: 1.0

Legend:
Nodes show data state and decision criteria to apply
Samples by class: [high-quality files, low-quality files]

Group A

human single-ended HistoneChIP-seq in MCF-7

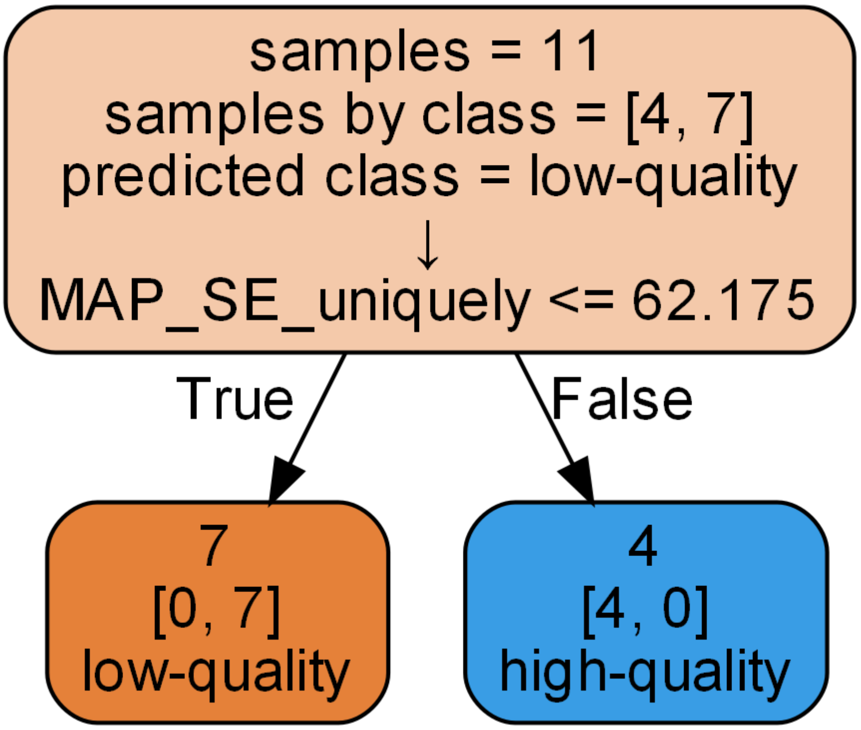

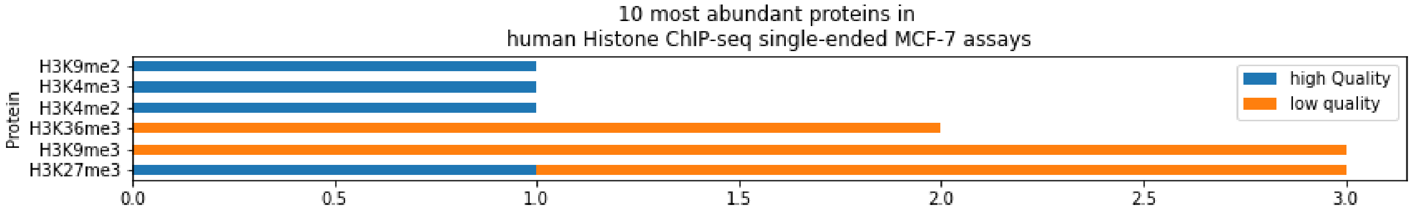

Metrics on training set:
- Accuracy: 1.0

High-quality files:
- Precision: 1.0
- Recall: 1.0
- F1-score: 1.0

Low-quality files
- Precision: 1.0
- Recall: 1.0
- F1-score: 1.0

Legend:
Nodes show data state and decision criteria to apply
Samples by class: [high-quality files, low-quality files]

Group A

human single-ended polyAplusRNA-seq in GM12878

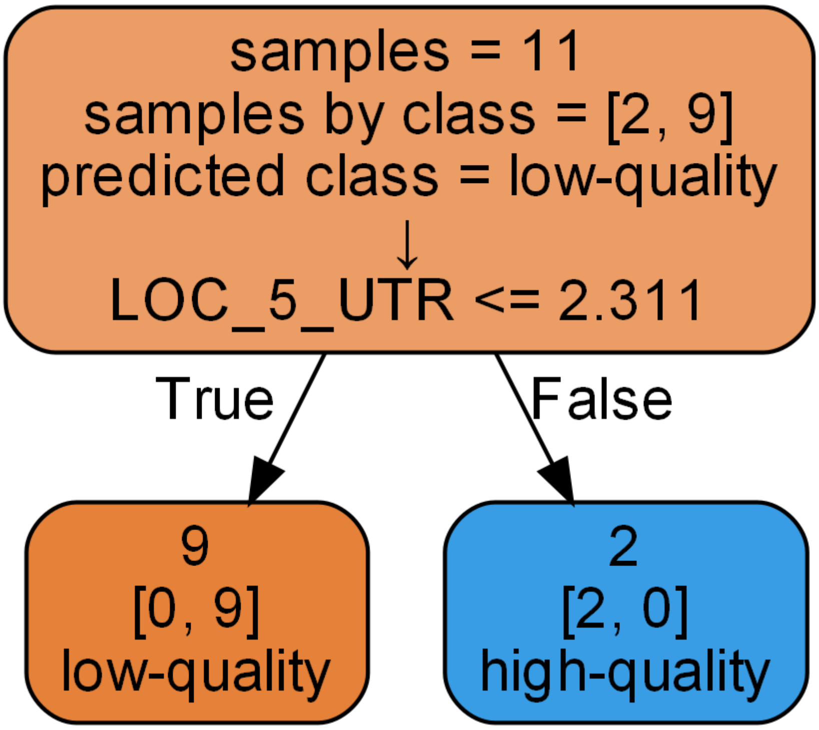

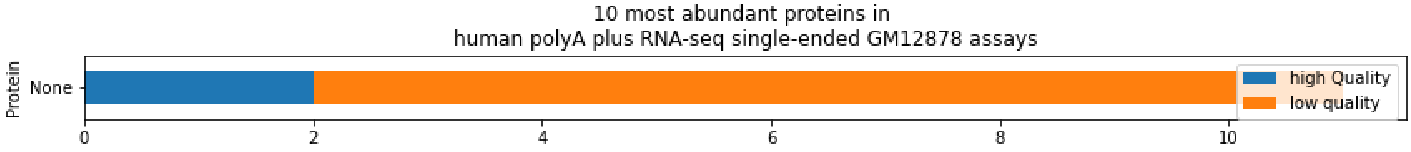

Metrics on training set:
- Accuracy: 1.0

High-quality files:
- Precision: 1.0
- Recall: 1.0
- F1-score: 1.0

Low-quality files
- Precision: 1.0
- Recall: 1.0
- F1-score: 1.0

Legend:
Nodes show data state and decision criteria to apply
Samples by class: [high-quality files, low-quality files]

Group A

human paired-ended TFChIP-seq in GM12878

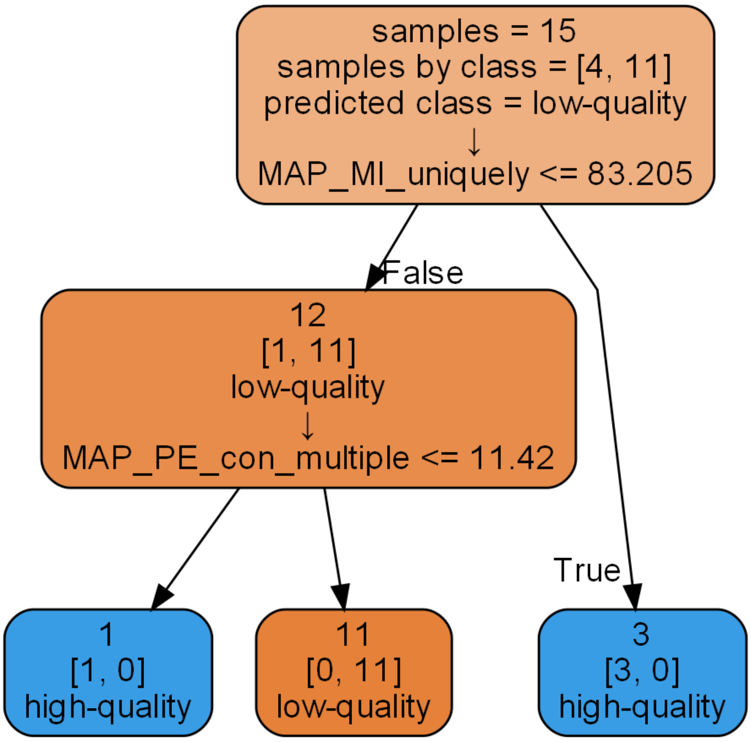

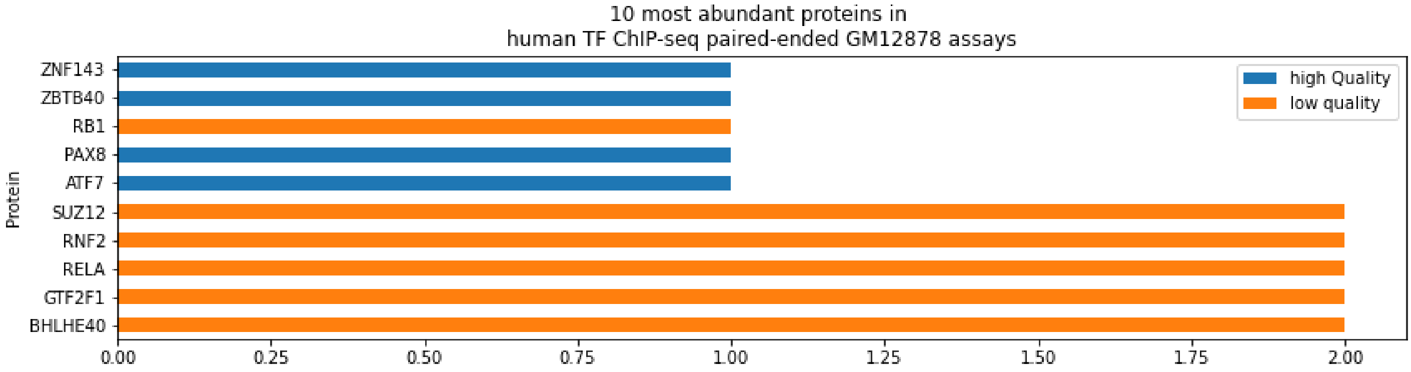

Metrics on training set:
- Accuracy: 1.0

High-quality files:
- Precision: 1.0
- Recall: 1.0
- F1-score: 1.0

Low-quality files
- Precision: 1.0
- Recall: 1.0
- F1-score: 1.0

Legend:
Nodes show data state and decision criteria to apply
Samples by class: [high-quality files, low-quality files]

Group A

human paired-ended TFChIP-seq in HEK293

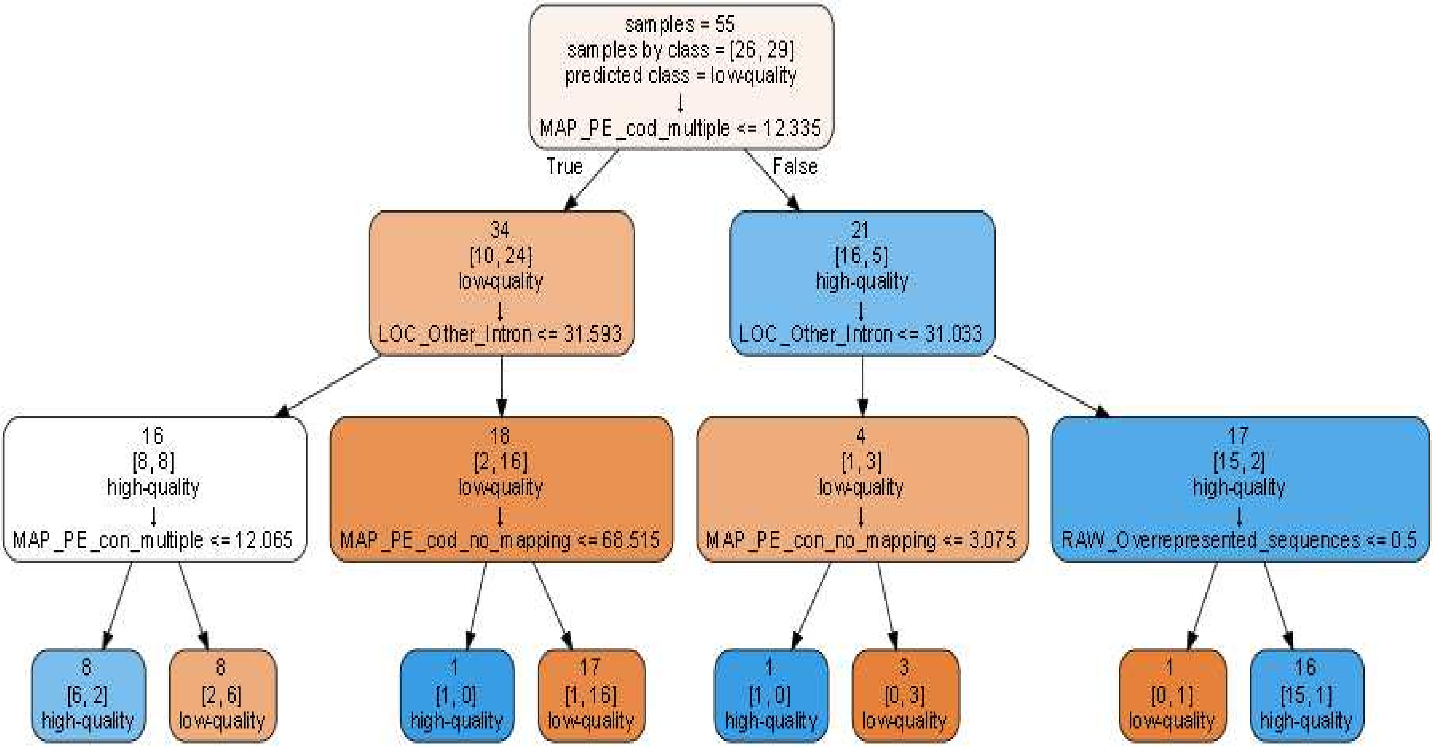

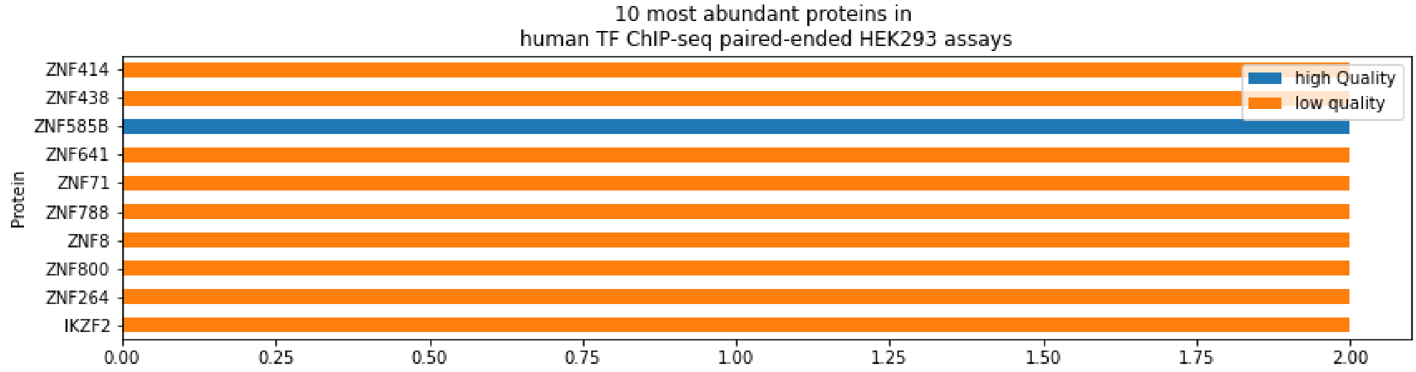

Metrics on training set:
- Accuracy: 0.89

High-quality files:
- Precision: 0.88
- Recall: 0.88
- F1-score: 0.88

Low-quality files
- Precision: 0.9
- Recall: 0.9
- F1-score: 0.9

Legend:
Nodes show data state and decision criteria to apply
Samples by class: [high-quality files, low-quality files]

Group A

human paired-ended TFChIP-seq in HepG2

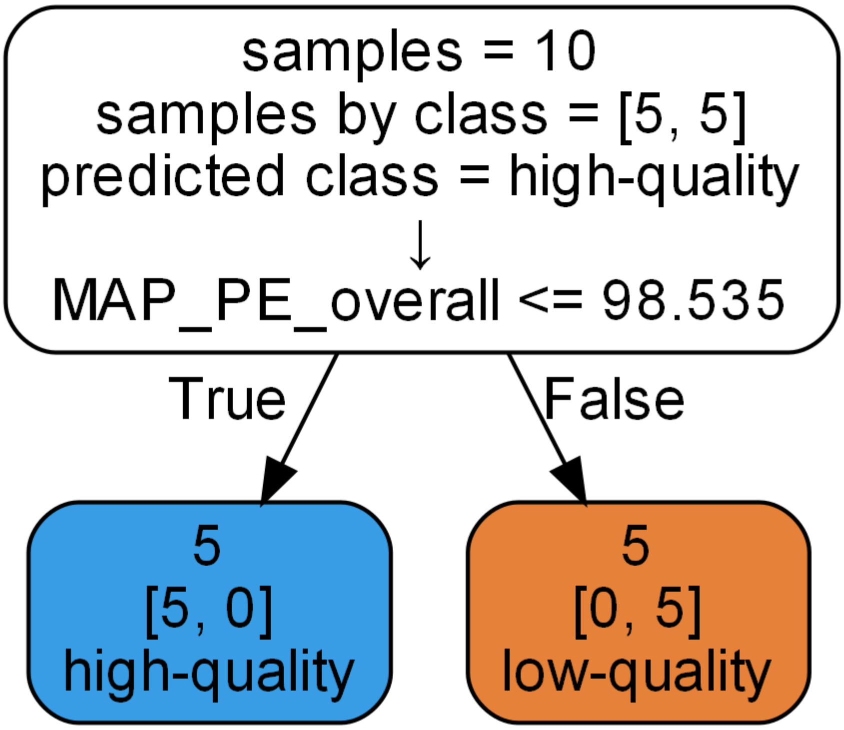

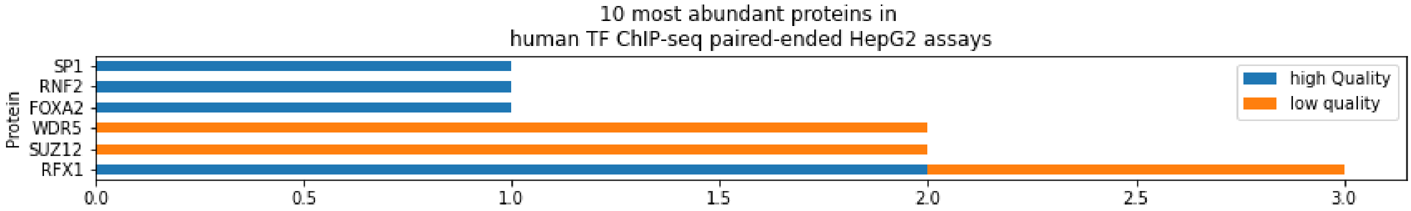

Metrics on training set:
- Accuracy: 1.0

High-quality files:
- Precision: 1.0
- Recall: 1.0
- F1-score: 1.0

Low-quality files
- Precision: 1.0
- Recall: 1.0
- F1-score: 1.0

Legend:
Nodes show data state and decision criteria to apply
Samples by class: [high-quality files, low-quality files]

Group A

human paired-ended TFChIP-seq in K562

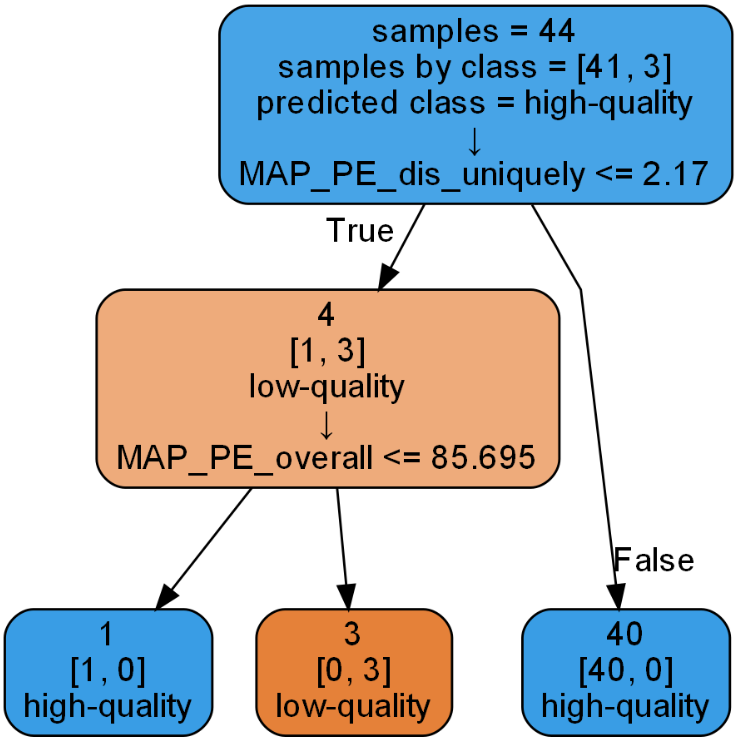

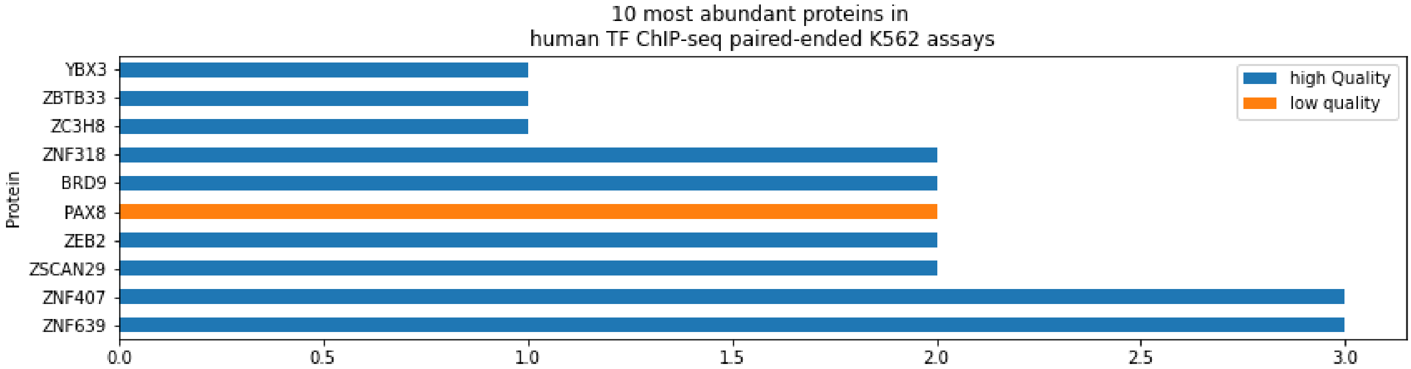

Metrics on training set:

- Accuracy: 1.0

High-quality files:

- Precision: 1.0
- Recall: 1.0
- F1-score: 1.0

Low-quality files

- Precision: 1.0
- Recall: 1.0
- F1-score: 1.0

Legend:

Nodes show data state and decision criteria to apply

Samples by class: [high-quality files, low-quality files]

Group A

human paired-ended TFChIP-seq in MCF-7

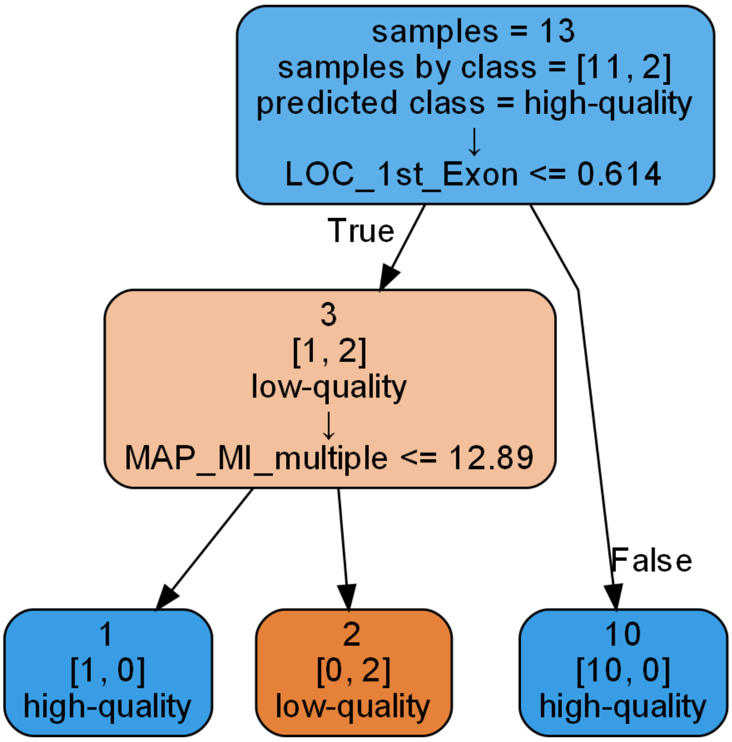

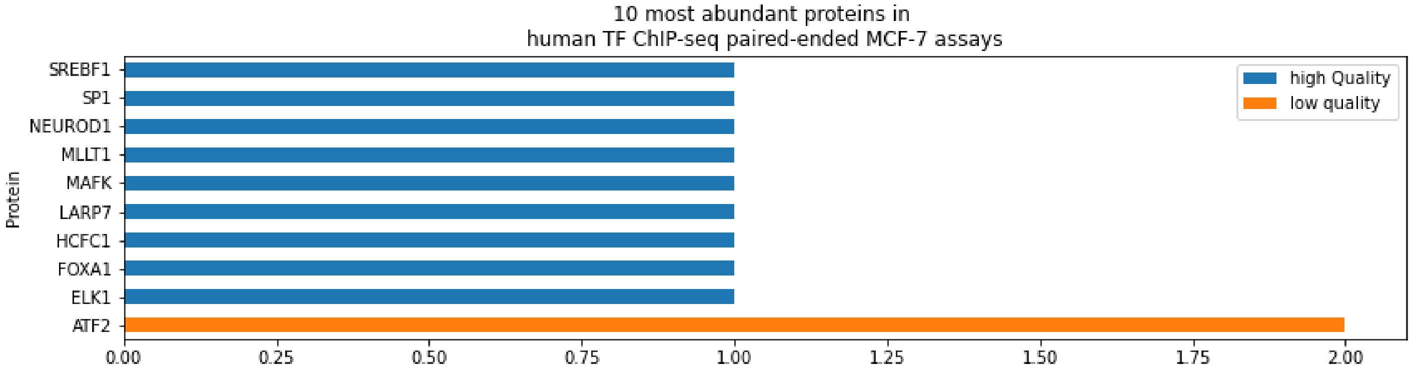

Metrics on training set:

- Accuracy: 1.0

High-quality files:

- Precision: 1.0
- Recall: 1.0
- F1-score: 1.0

Low-quality files

- Precision: 1.0
- Recall: 1.0
- F1-score: 1.0

Legend:

Nodes show data state and decision criteria to apply

Samples by class: [high-quality files, low-quality files]

Group A
human single-ended TFChIP-seq in A549

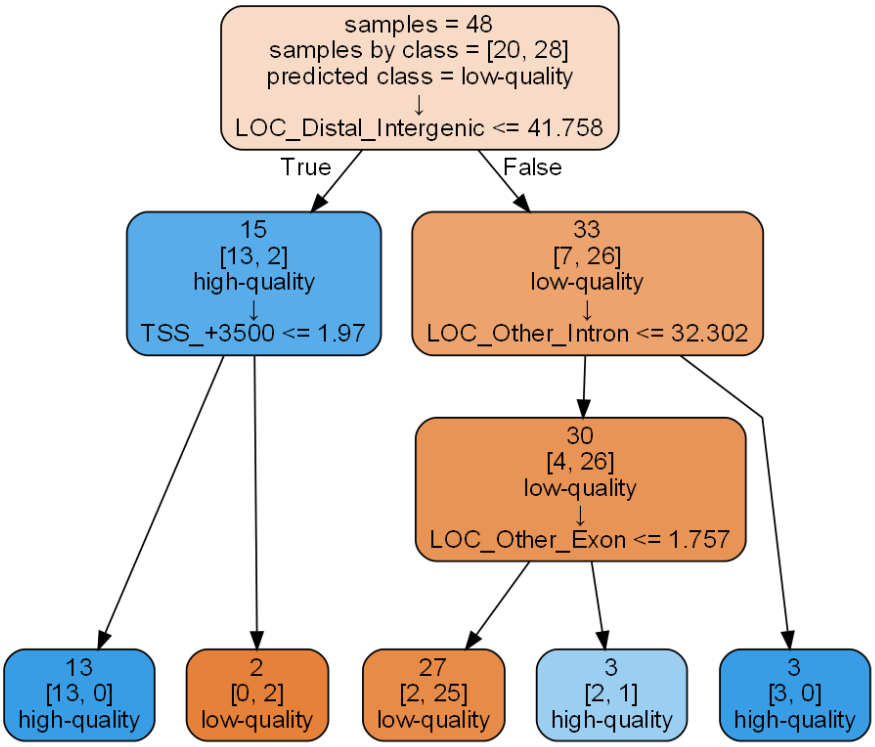

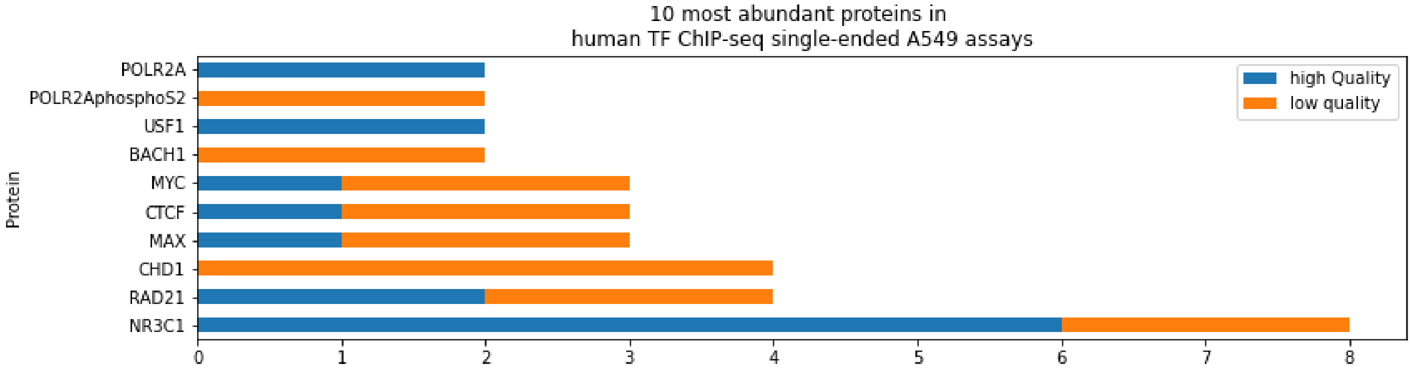

Metrics on training set:
- Accuracy: 0.94

High-quality files:
- Precision: 0.95
- Recall: 0.9
- F1-score: 0.92

Low-quality files
- Precision: 0.93
- Recall: 0.96
- F1-score: 0.95

Legend:
Nodes show data state and decision criteria to apply
Samples by class: [high-quality files, low-quality files]

Group A

human single-ended TFChIP-seq in GM12878

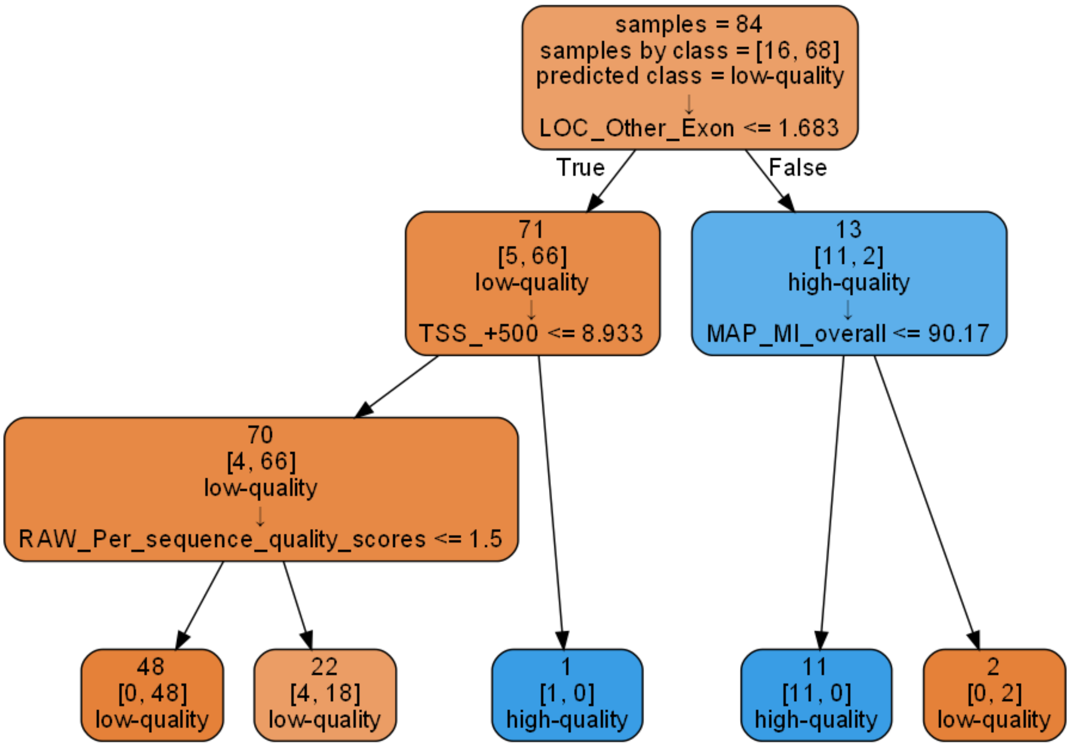

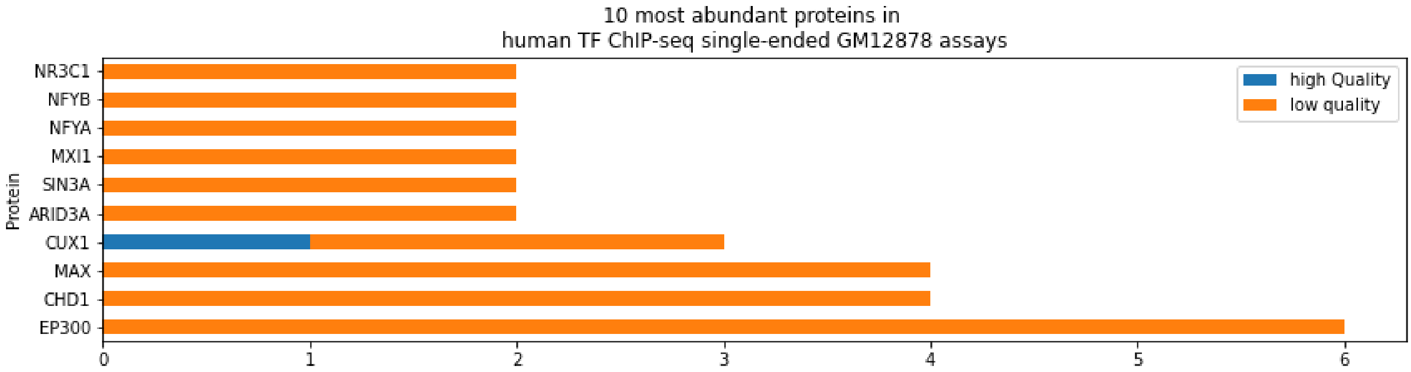

Metrics on training set:

- Accuracy: 0.95

High-quality files:

- Precision: 1.0
- Recall: 0.75
- F1-score: 0.86

Low-quality files

- Precision: 0.94
- Recall: 1.0
- F1-score: 0.97

Legend:

Nodes show data state and decision criteria to apply

Samples by class: [high-quality files, low-quality files]

Group A

human single-ended TFChIP-seq in H1

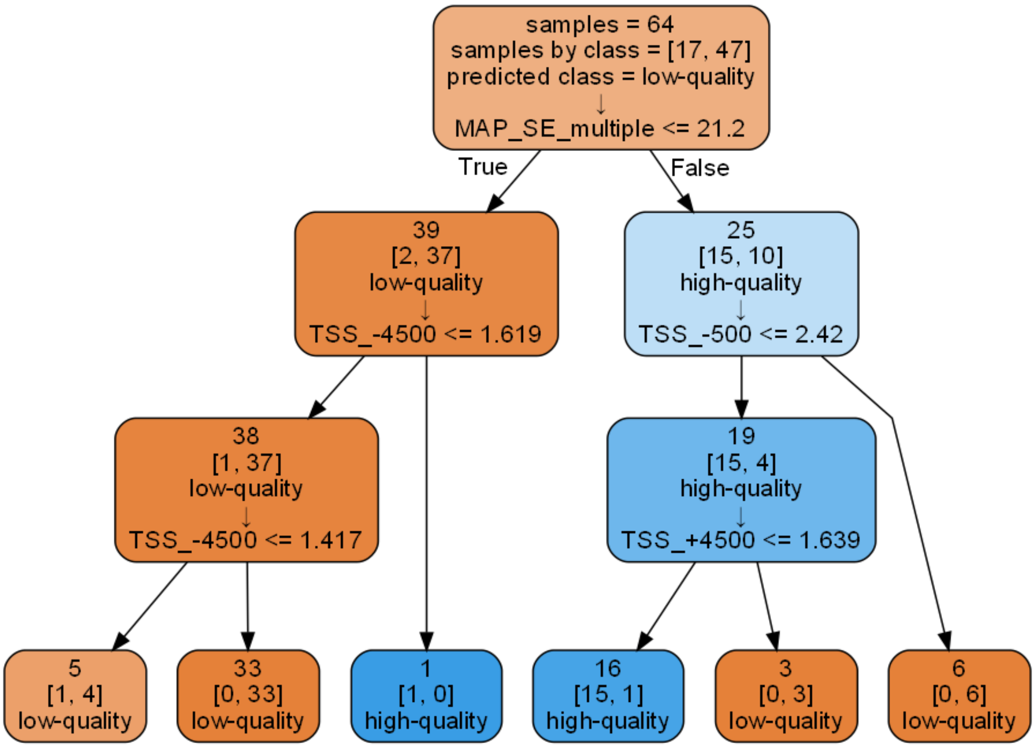

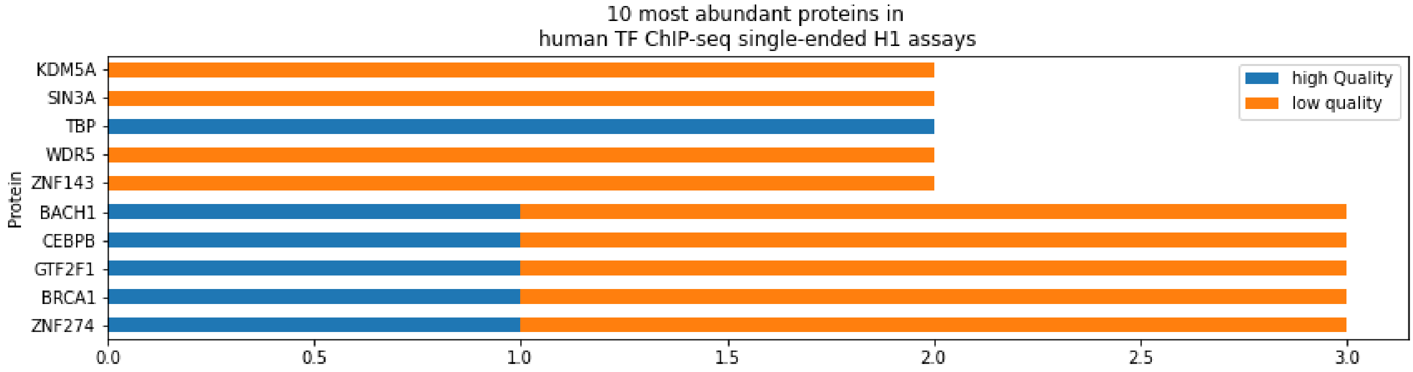

Metrics on training set:
- Accuracy: 0.97

High-quality files:
- Precision: 0.94
- Recall: 0.94
- F1-score: 0.94

Low-quality files
- Precision: 0.98
- Recall: 0.98
- F1-score: 0.98

Legend:
Nodes show data state and decision criteria to apply
Samples by class: [high-quality files, low-quality files]

Group A

human single-ended TFChIP-seq in HeLa-S3

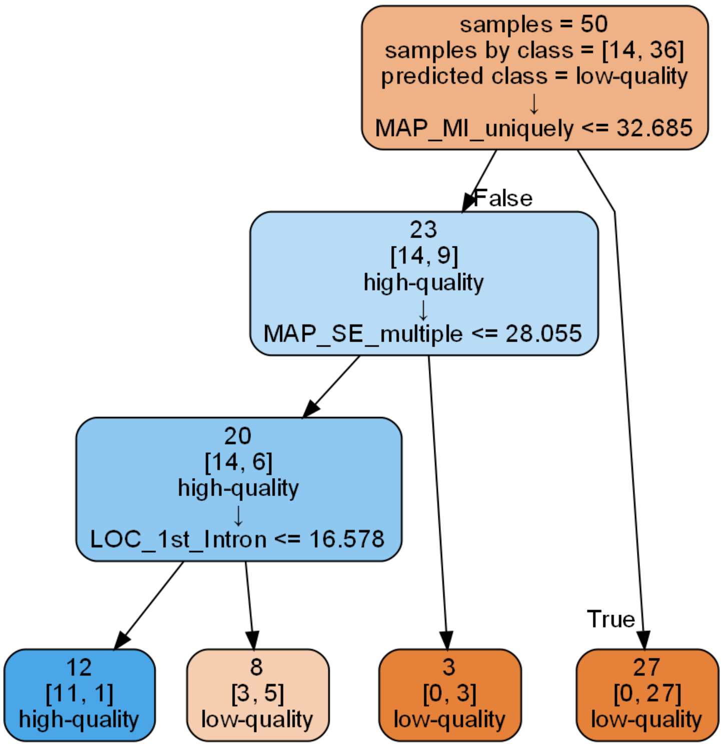

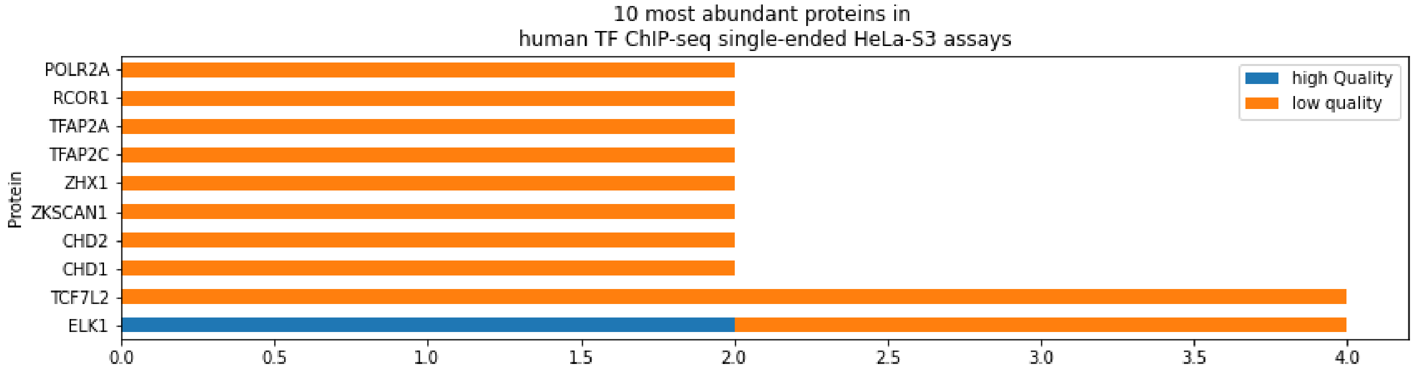

Metrics on training set:

- Accuracy: 0.92

High-quality files:

- Precision: 0.92
- Recall: 0.79
- F1-score: 0.85

Low-quality files

- Precision: 0.92
- Recall: 0.97
- F1-score: 0.95

Legend:

Nodes show data state and decision criteria to apply

Samples by class: [high-quality files, low-quality files]

Group A

human single-ended TFChIP-seq in HepG2

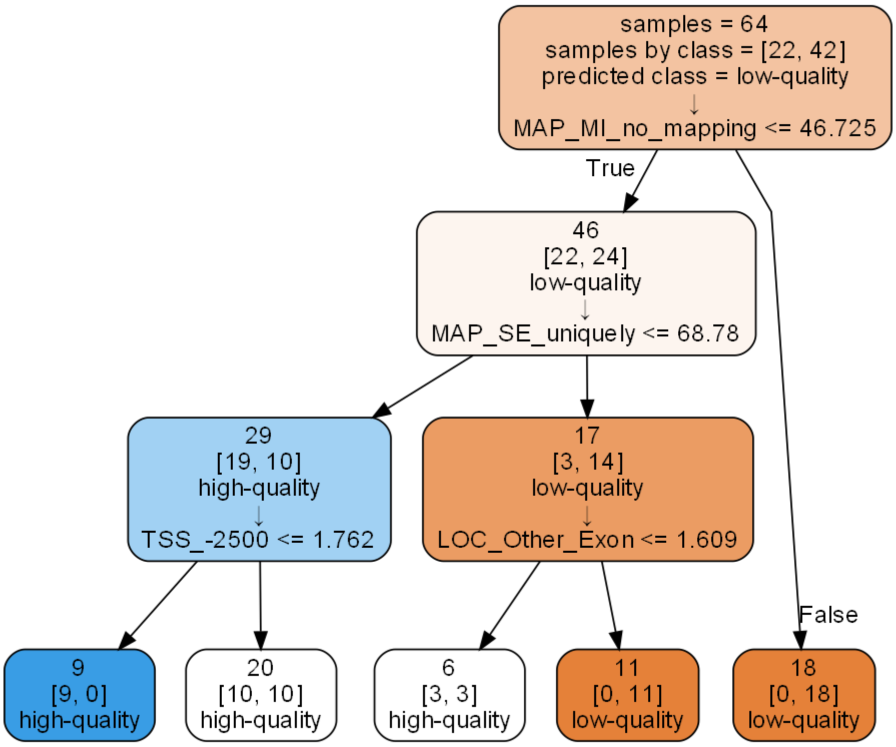

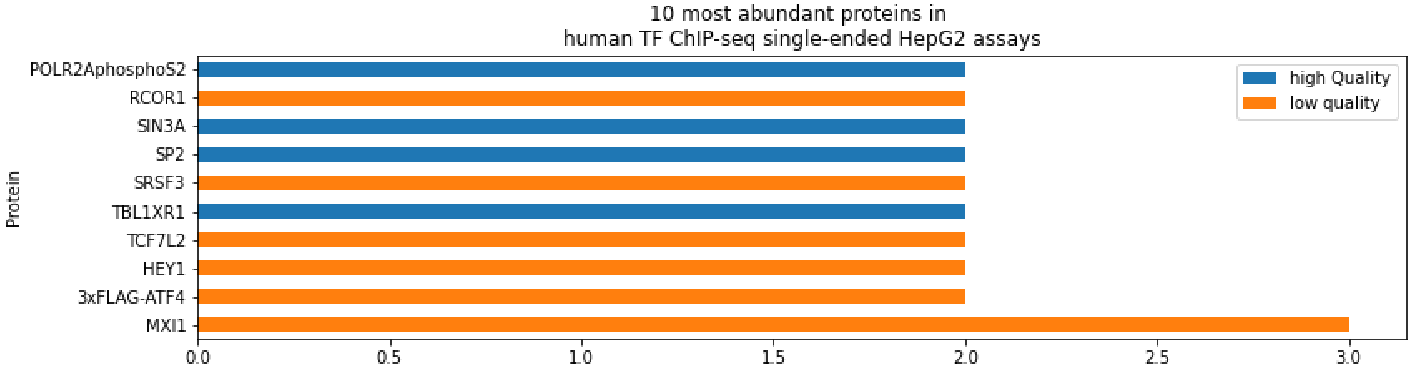

Metrics on training set:

- Accuracy: 0.8

High-quality files:

- Precision: 0.63
- Recall: 1.0
- F1-score: 0.77

Low-quality files

- Precision: 1.0
- Recall: 0.69
- F1-score: 0.82

Legend:
Nodes show data state and decision criteria to apply
Samples by class: [high-quality files, low-quality files]

Group A
human single-ended TFChIP-seq in IMR-90

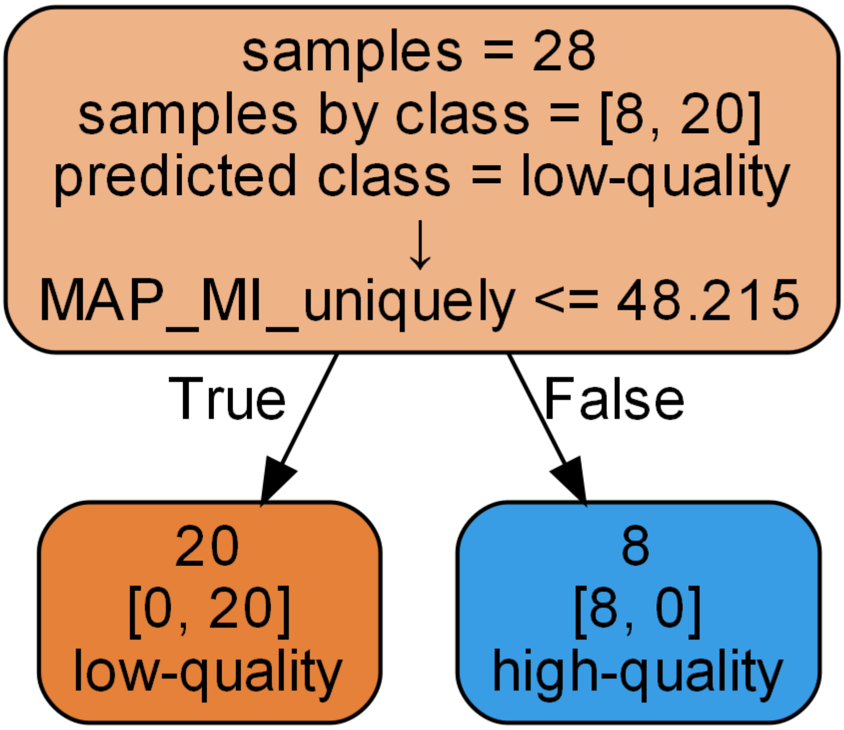

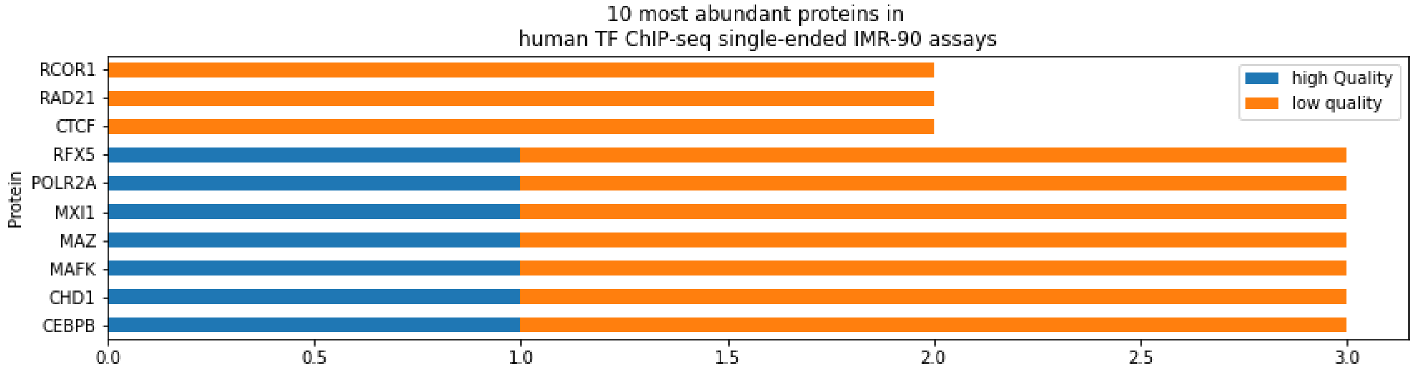

Metrics on training set:
- Accuracy: 1.0

High-quality files:
- Precision: 1.0
- Recall: 1.0
- F1-score: 1.0

Low-quality files
- Precision: 1.0
- Recall: 1.0
- F1-score: 1.0

Legend:
Nodes show data state and decision criteria to apply
Samples by class: [high-quality files, low-quality files]

Group A

human single-ended TFChIP-seq in K562

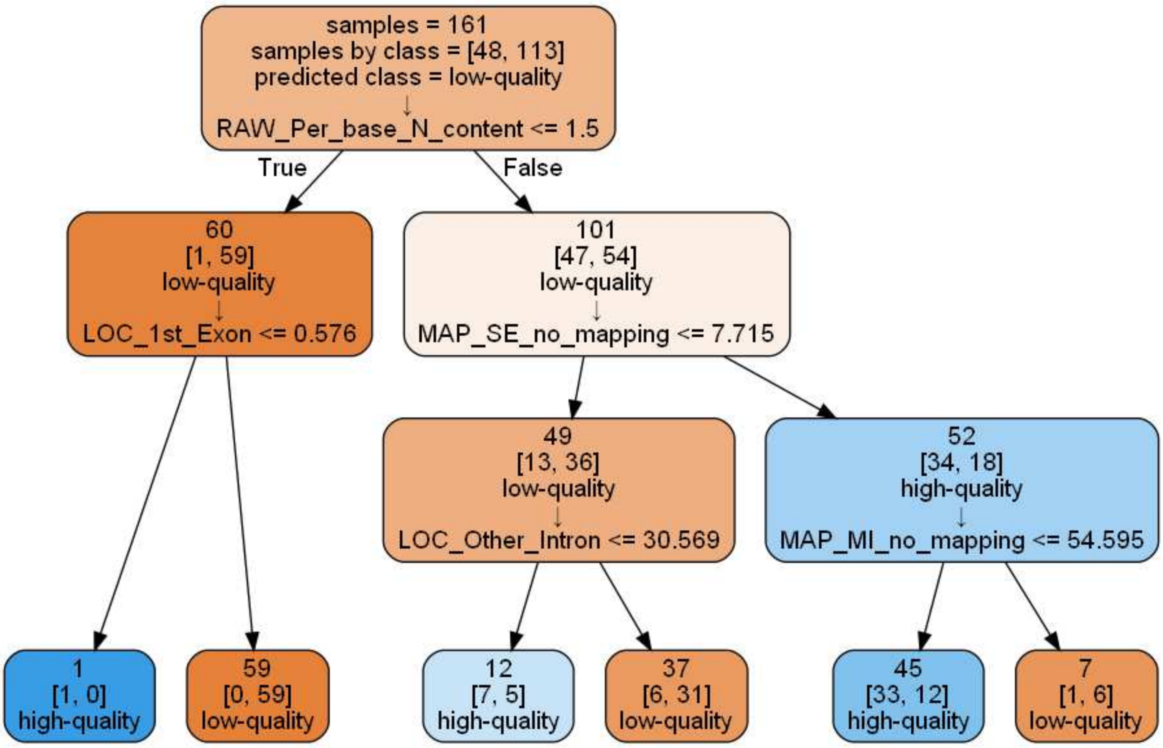

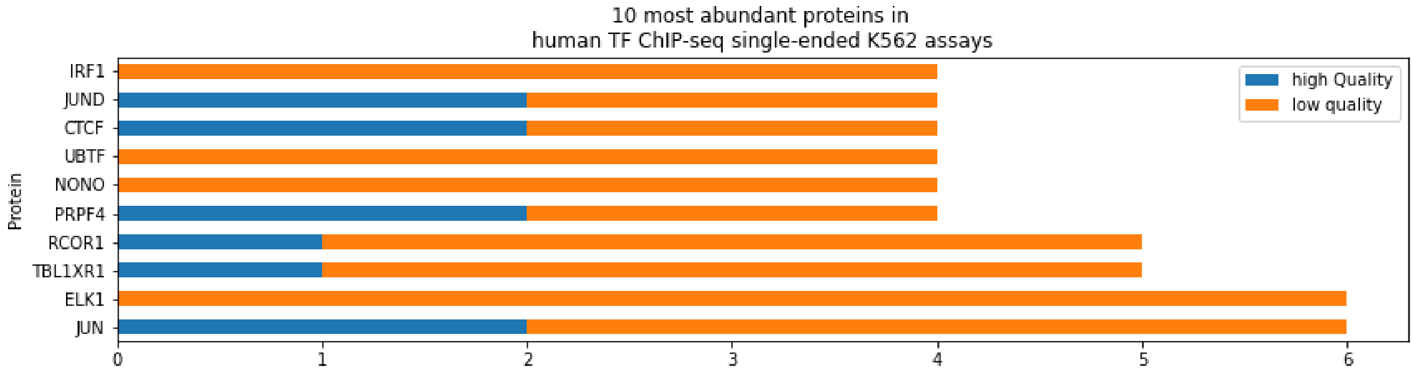

Metrics on training set:
- Accuracy: 0.85

High-quality files:
- Precision: 0.71
- Recall: 0.85
- F1-score: 0.77

Low-quality files
- Precision: 0.93
- Recall: 0.85
- F1-score: 0.89

Legend:
Nodes show data state and decision criteria to apply
Samples by class: [high-quality files, low-quality files]

Group A

human single-ended TFChIP-seq in MCF-7

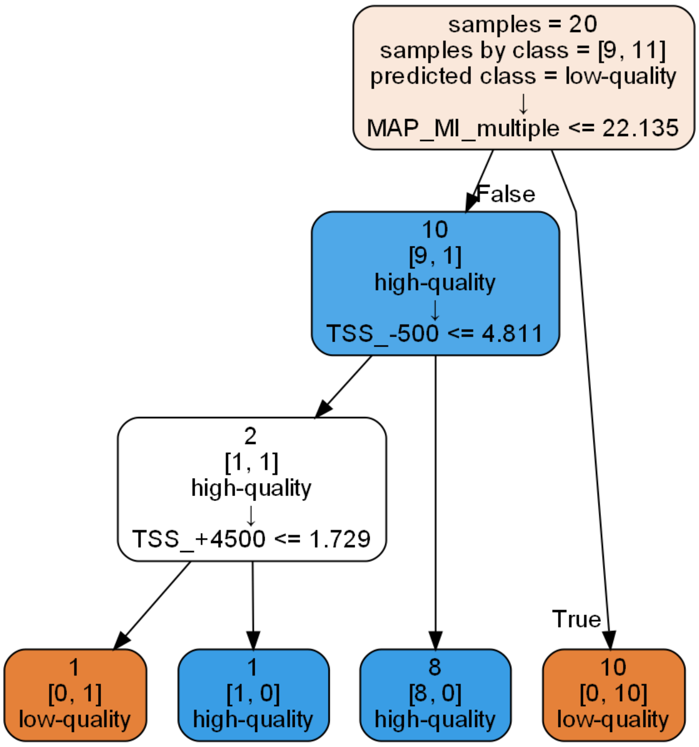

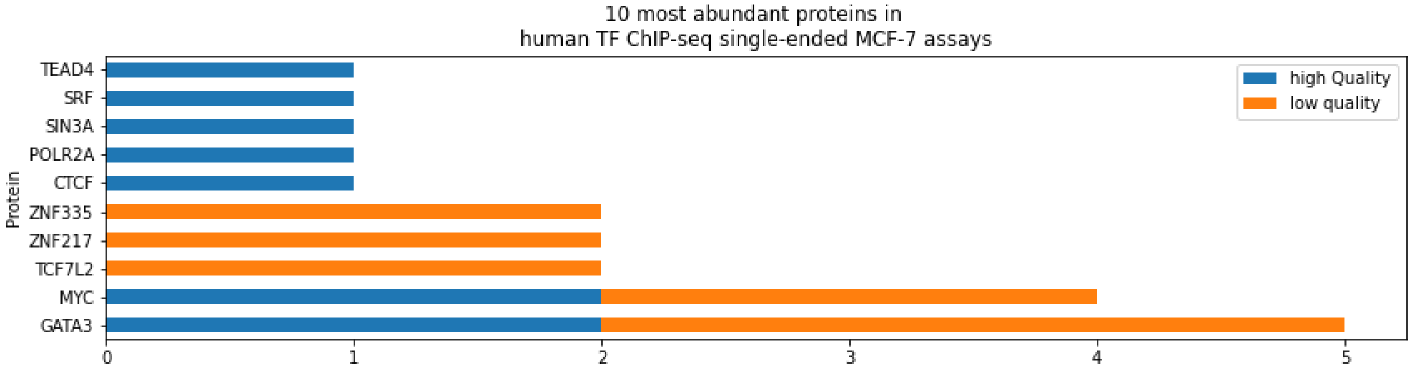

Metrics on training set:

- Accuracy: 1.0

High-quality files:

- Precision: 1.0
- Recall: 1.0
- F1-score: 1.0

Low-quality files

- Precision: 1.0
- Recall: 1.0
- F1-score: 1.0

Legend:

Nodes show data state and decision criteria to apply

Samples by class: [high-quality files, low-quality files]

Group A

human single-ended TFChIP-seq in MCF_10A

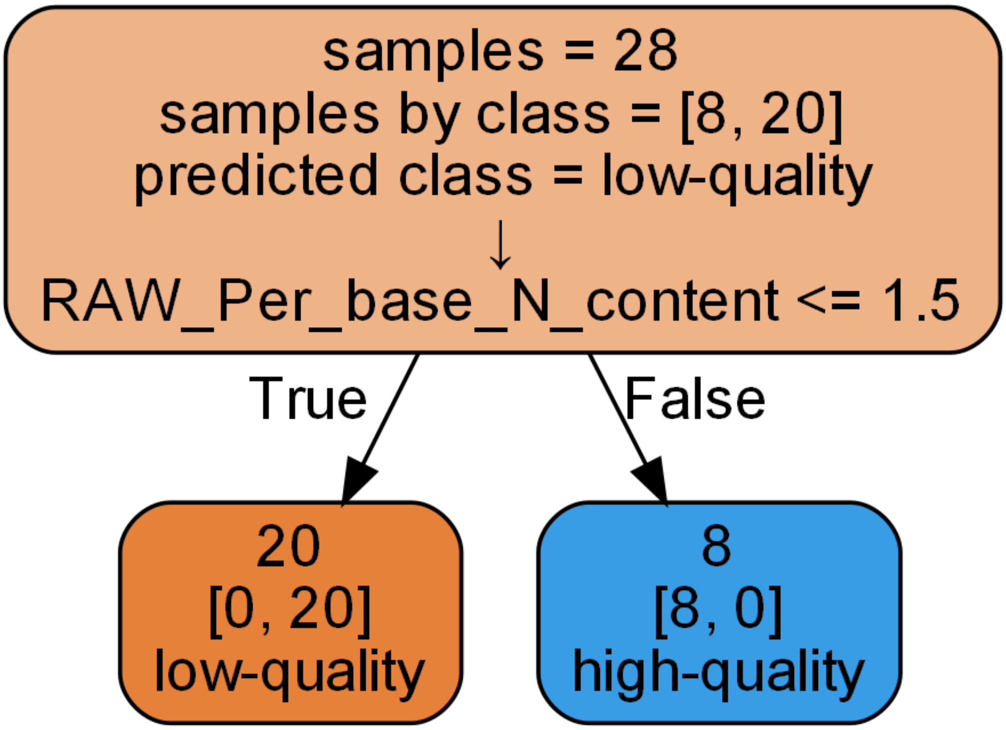

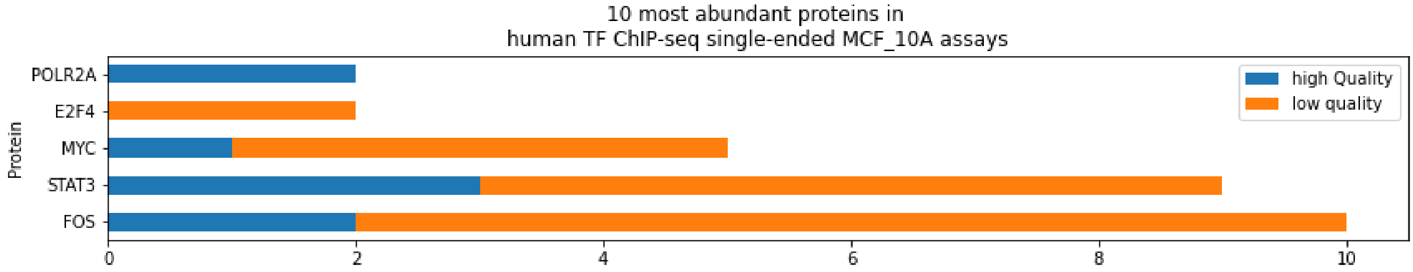

Metrics on training set:		
- Accuracy:	1.0	
High-quality files:		
- Precision:	1.0	
- Recall:	1.0	
- F1-score:	1.0	
Low-quality files		
- Precision:	1.0	
- Recall:	1.0	
- F1-score:	1.0	

Legend:
Nodes show data state and decision criteria to apply
Samples by class: [high-quality files, low-quality files]

Group A

human single-ended TFChIP-seq in SK-N-SH

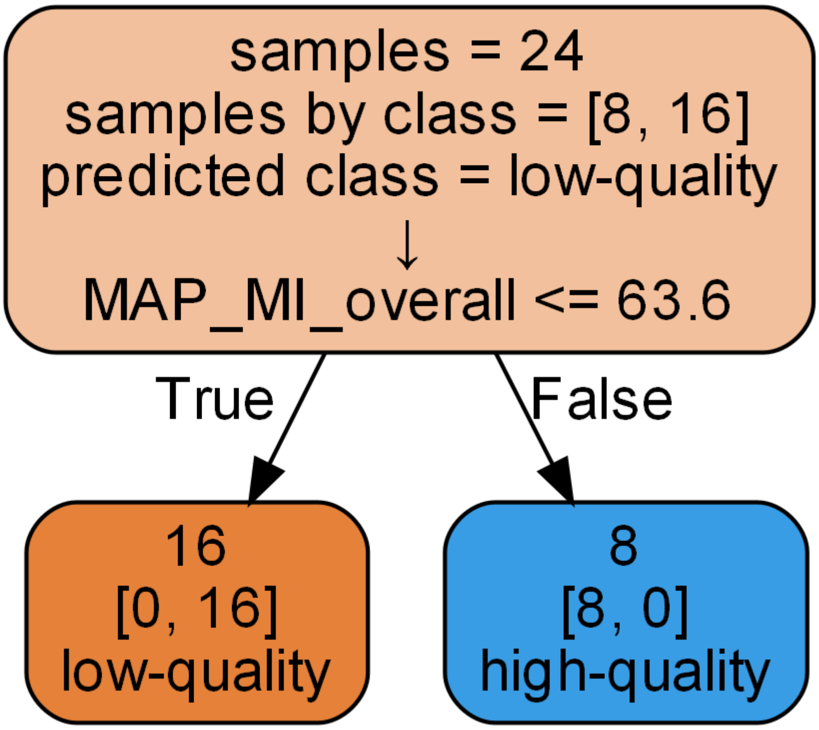

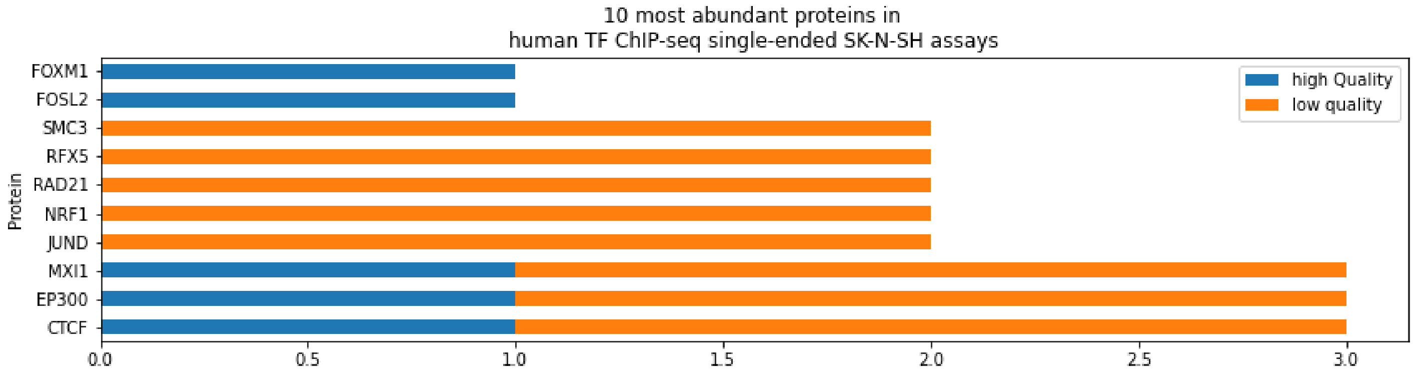

Metrics on training set:
- Accuracy: 1.0

High-quality files:
- Precision: 1.0
- Recall: 1.0
- F1-score: 1.0

Low-quality files
- Precision: 1.0
- Recall: 1.0
- F1-score: 1.0

Legend:
Nodes show data state and decision criteria to apply
Samples by class: [high-quality files, low-quality files]

Group A
mouse single-ended ControlChIP-seq in MEL_cell_line

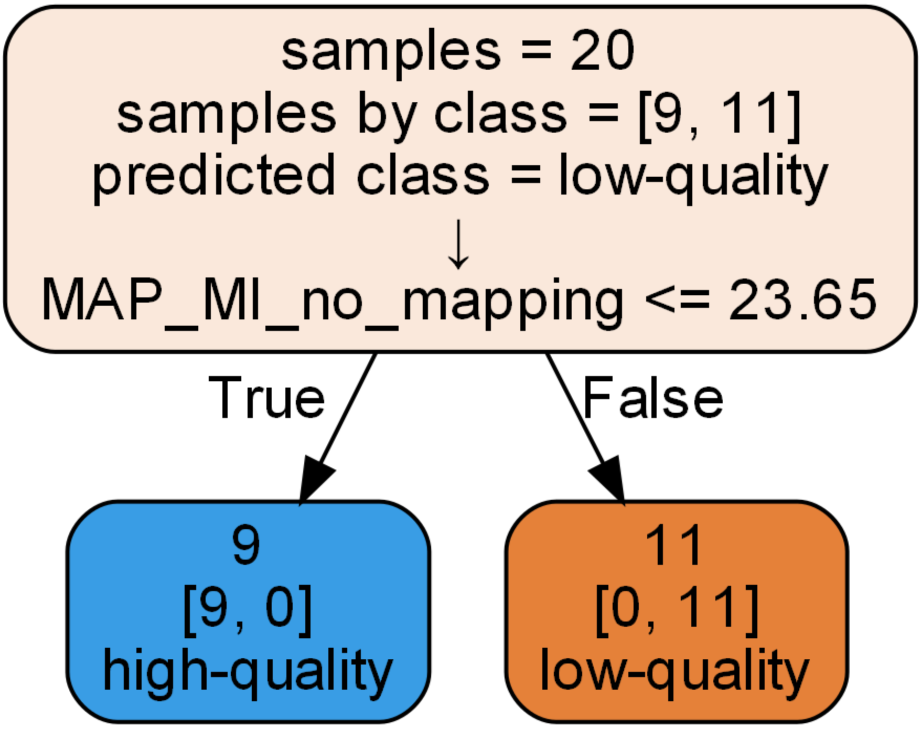

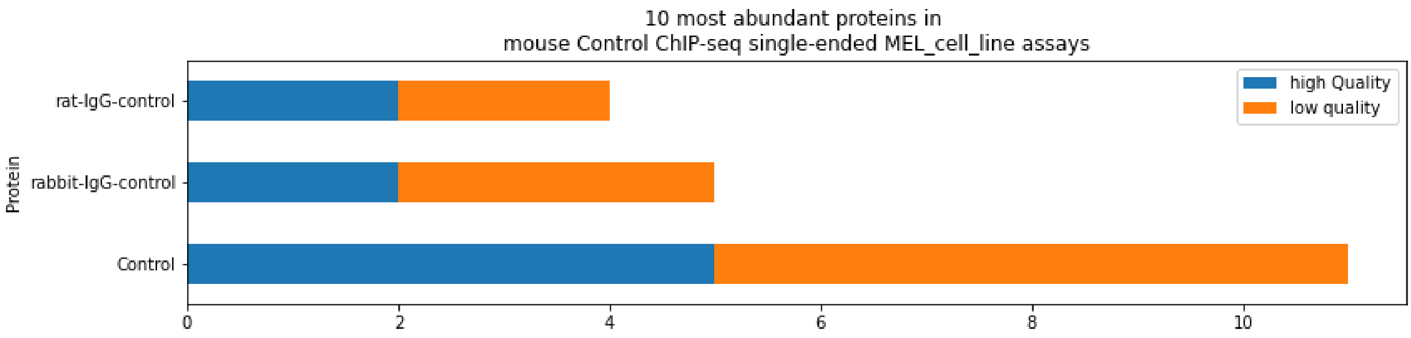

Metrics on training set:
- Accuracy: 1.0

High-quality files:
- Precision: 1.0
- Recall: 1.0
- F1-score: 1.0

Low-quality files
- Precision: 1.0
- Recall: 1.0
- F1-score: 1.0

Legend:
Nodes show data state and decision criteria to apply
Samples by class: [high-quality files, low-quality files]

Group A

mouse paired-ended DNase-seq in hindbrain

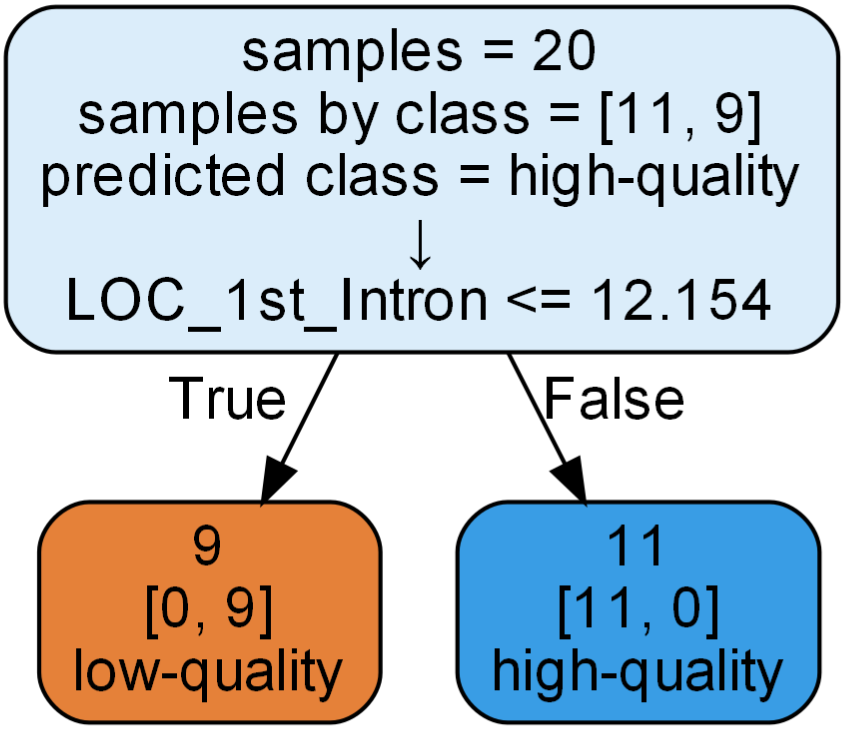

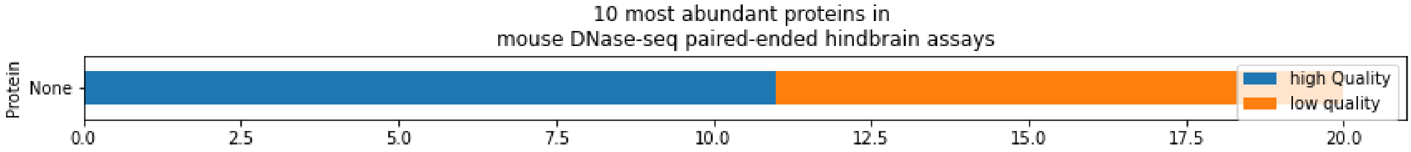

Metrics on training set:

- Accuracy:	1.0
High-quality files:	
- Precision:	1.0
- Recall:	1.0
- F1-score:	1.0
Low-quality files	
- Precision:	1.0
- Recall:	1.0
- F1-score:	1.0

Legend:
Nodes show data state and decision criteria to apply
Samples by class: [high-quality files, low-quality files]

Group A

mouse paired-ended DNase-seq in limb

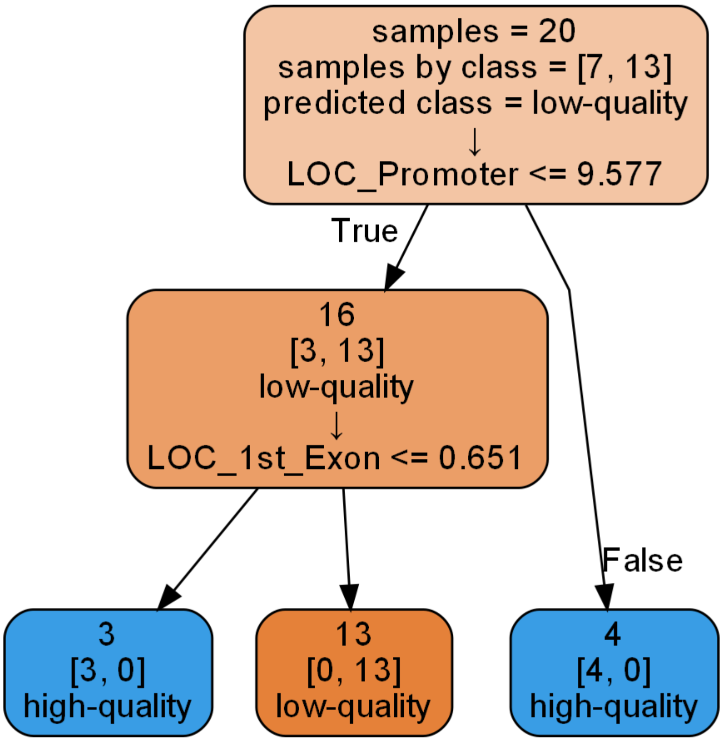

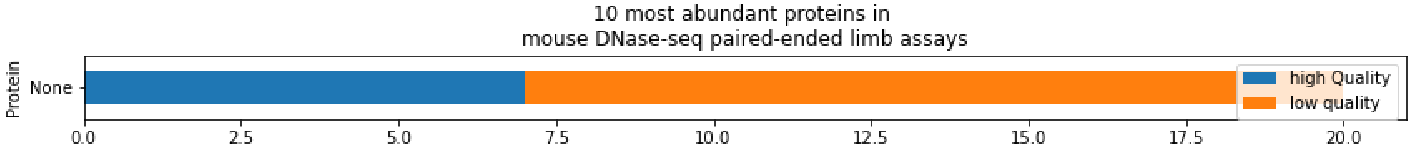

Metrics on training set:

- Accuracy:	1.0
High-quality files:	
- Precision:	1.0
- Recall:	1.0
- F1-score:	1.0
Low-quality files	
- Precision:	1.0
- Recall:	1.0
- F1-score:	1.0

Legend:

Nodes show data state and decision criteria to apply

Samples by class: [high-quality files, low-quality files]

Group A

mouse paired-ended DNase-seq in midbrain

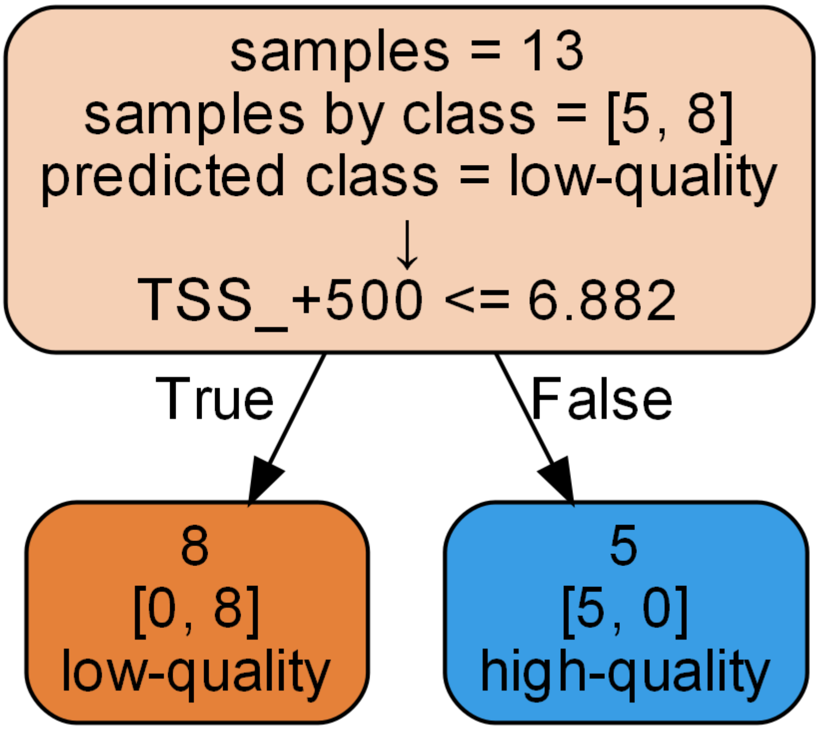

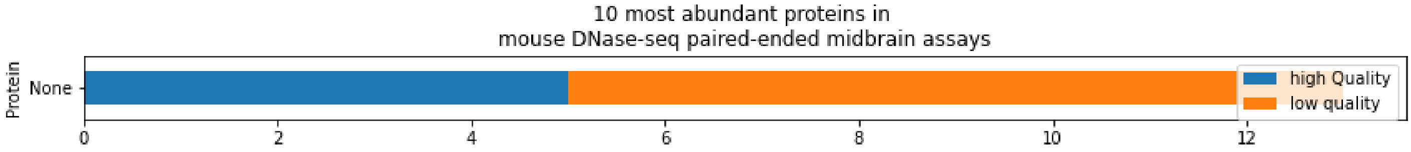

Metrics on training set:
- Accuracy: 1.0

High-quality files:
- Precision: 1.0
- Recall: 1.0
- F1-score: 1.0

Low-quality files
- Precision: 1.0
- Recall: 1.0
- F1-score: 1.0

Legend:
Nodes show data state and decision criteria to apply
Samples by class: [high-quality files, low-quality files]

Group A

mouse single-ended TFChIP-seq in CH12.LX

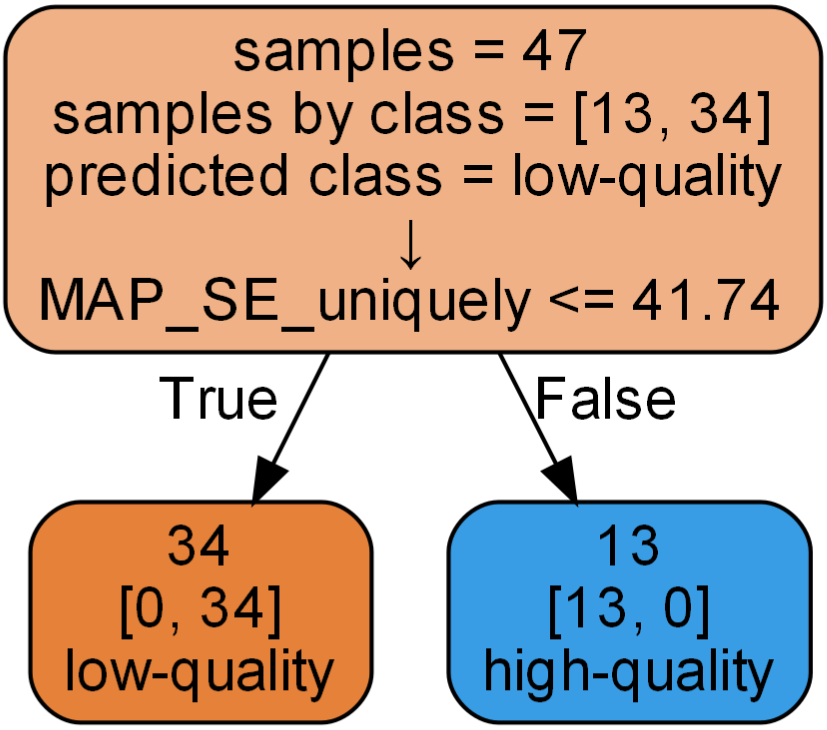

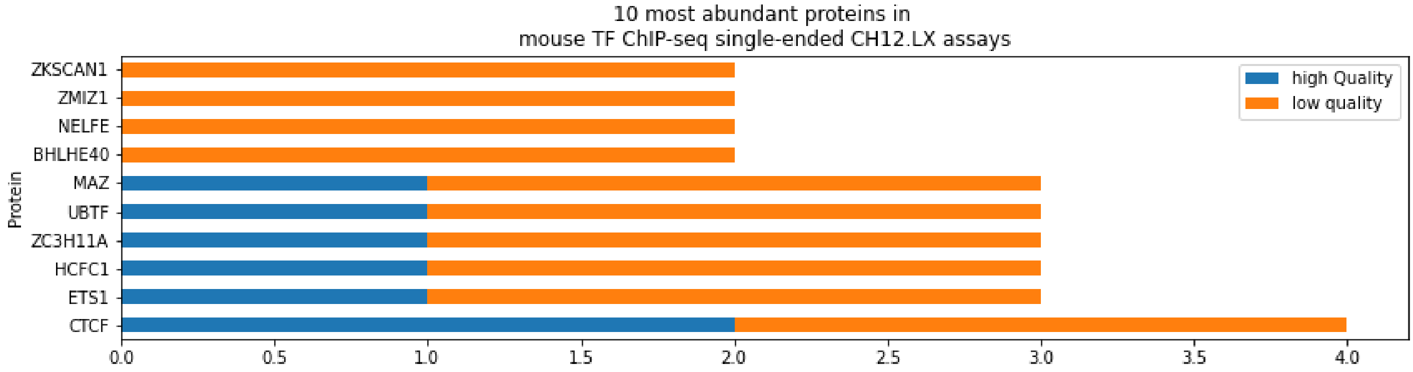

Metrics on training set:

- Accuracy: 1.0

High-quality files:

- Precision: 1.0
- Recall: 1.0
- F1-score: 1.0

Low-quality files

- Precision: 1.0
- Recall: 1.0
- F1-score: 1.0

Legend:

Nodes show data state and decision criteria to apply

Samples by class: [high-quality files, low-quality files]

Group A

mouse single-ended TFChIP-seq in MEL_cell_line

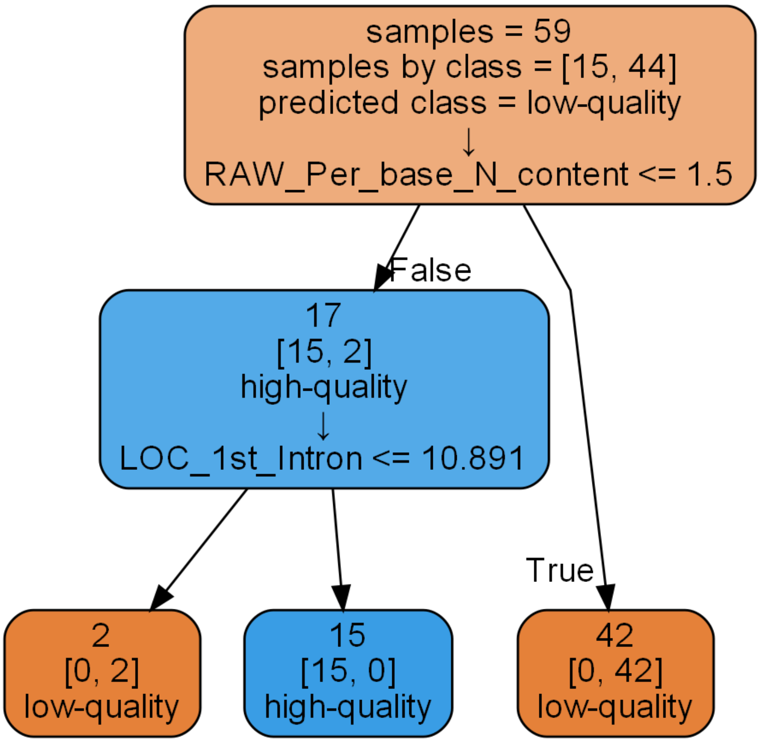

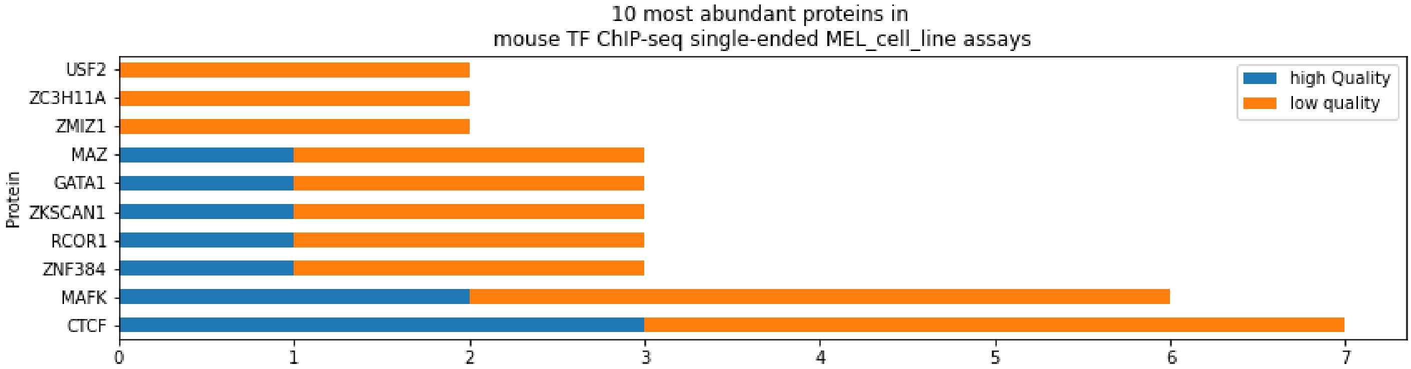

Metrics on training set:
- Accuracy: 1.0

High-quality files:
- Precision: 1.0
- Recall: 1.0
- F1-score: 1.0

Low-quality files
- Precision: 1.0
- Recall: 1.0
- F1-score: 1.0

Legend:
Nodes show data state and decision criteria to apply
Samples by class: [high-quality files, low-quality files]

Group B

human paired-ended ControlChIP-seq for Control

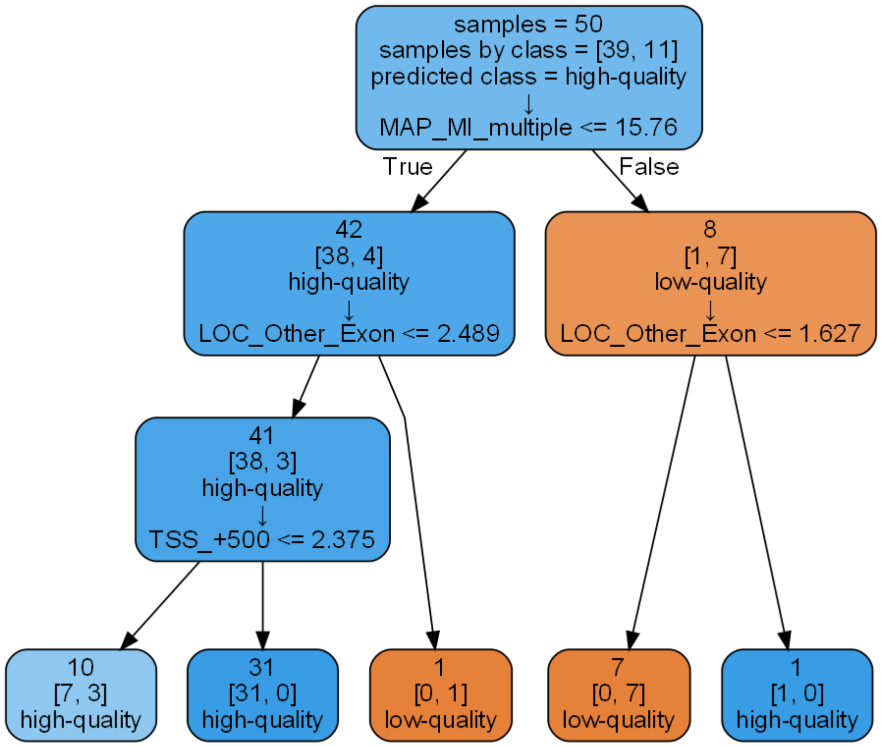

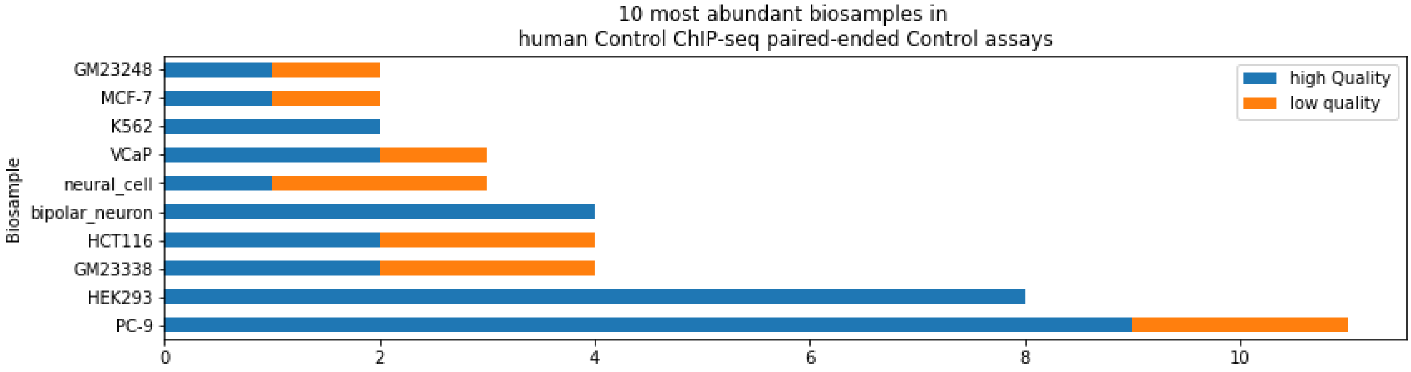

Metrics on training set:

- Accuracy: 0.94

High-quality files:

- Precision: 0.93
- Recall: 1.0
- F1-score: 0.96

Low-quality files

- Precision: 1.0
- Recall: 0.73
- F1-score: 0.84

Legend:

Nodes show data state and decision criteria to apply

Samples by class: [high-quality files, low-quality files]

Group B

human single-ended ControlChIP-seq for Control

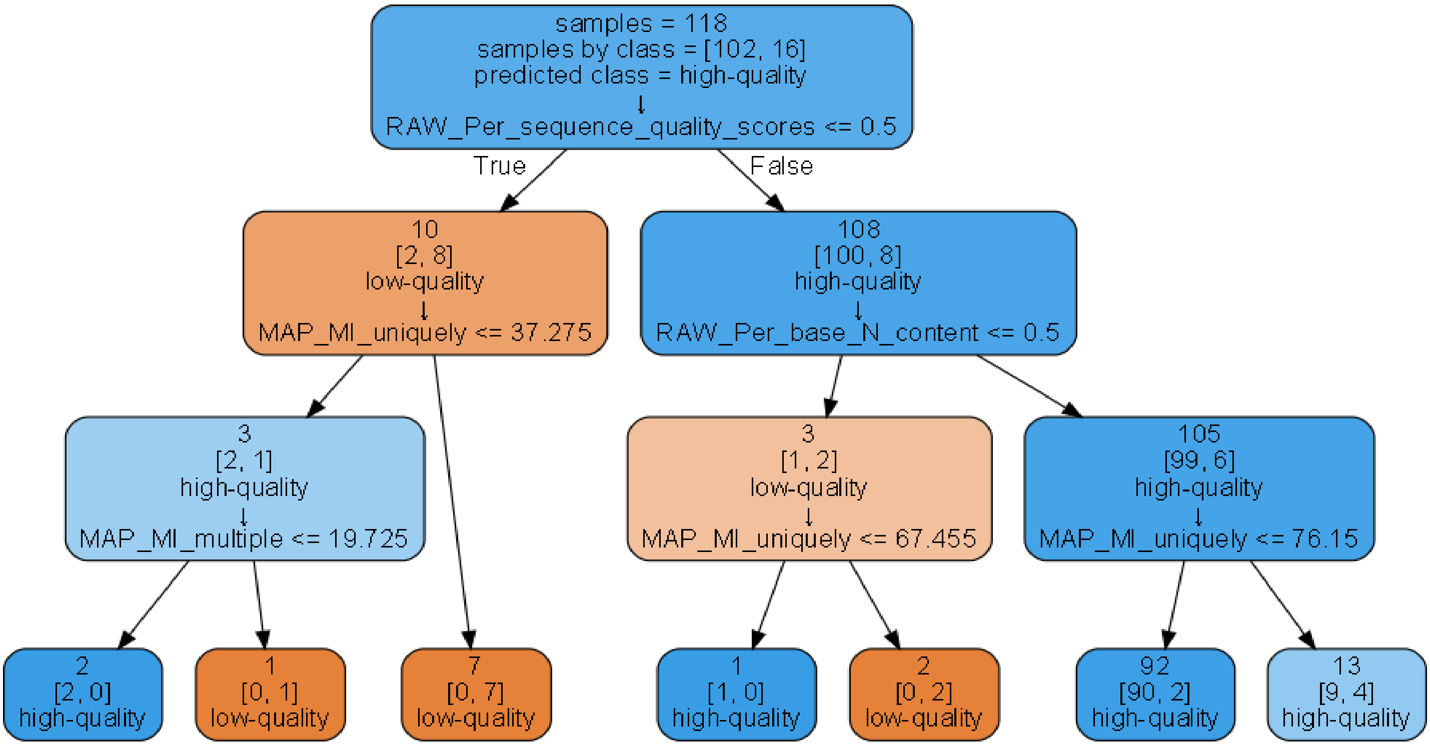

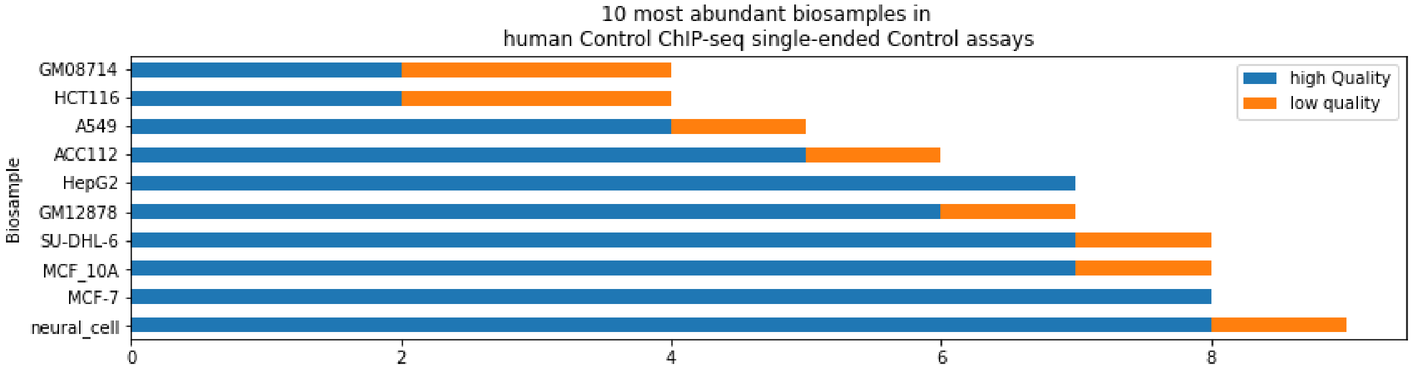

Metrics on training set:
- Accuracy: 0.95

High-quality files:
- Precision: 0.94
- Recall: 1.0
- F1-score: 0.97

Low-quality files
- Precision: 1.0
- Recall: 0.62
- F1-score: 0.77

Legend:
Nodes show data state and decision criteria to apply
Samples by class: [high-quality files, low-quality files]

Group B

human single-ended ControlChIP-seq for rabbit-IgG-control

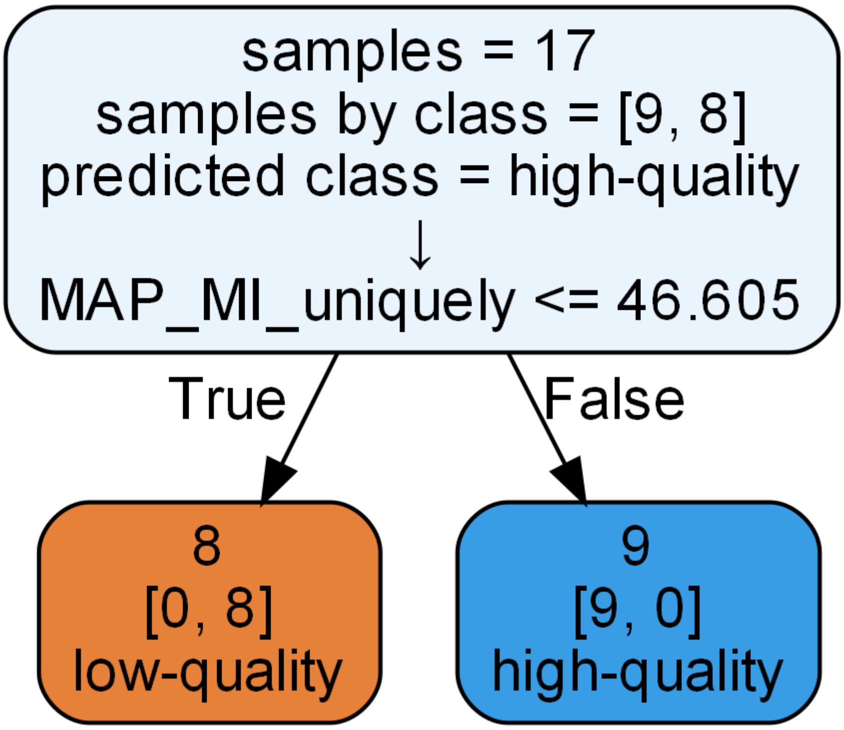

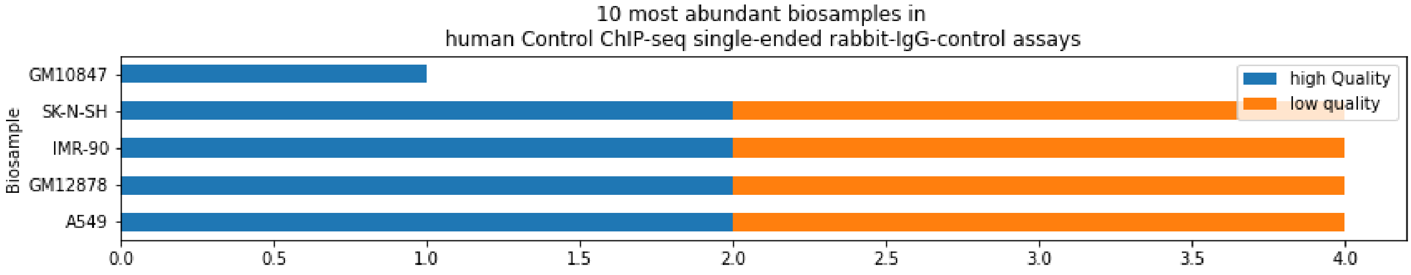

Metrics on training set:
- Accuracy: 1.0

High-quality files:
- Precision: 1.0
- Recall: 1.0
- F1-score: 1.0

Low-quality files
- Precision: 1.0
- Recall: 1.0
- F1-score: 1.0

Legend:
Nodes show data state and decision criteria to apply
Samples by class: [high-quality files, low-quality files]

Group B

human single-ended HistoneChIP-seq for H3K27ac

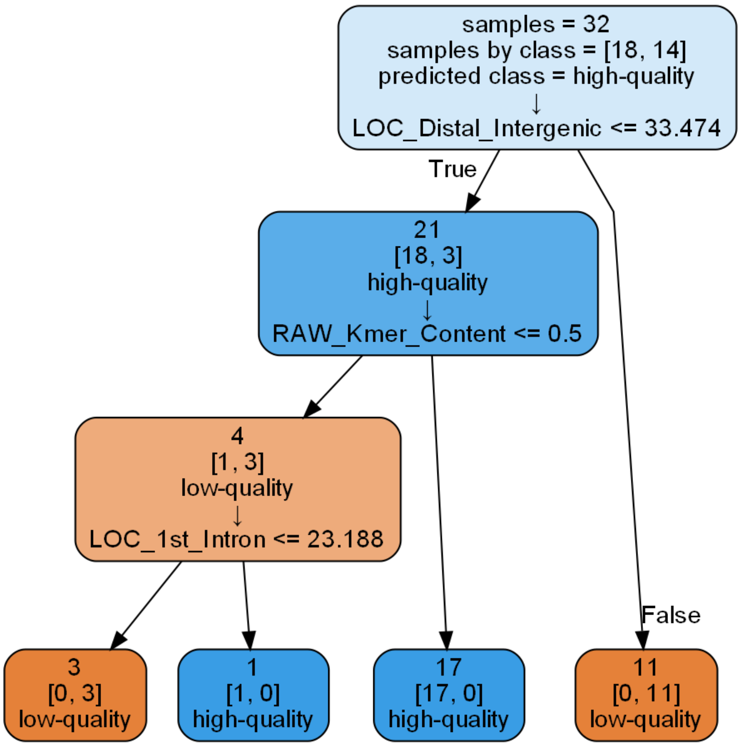

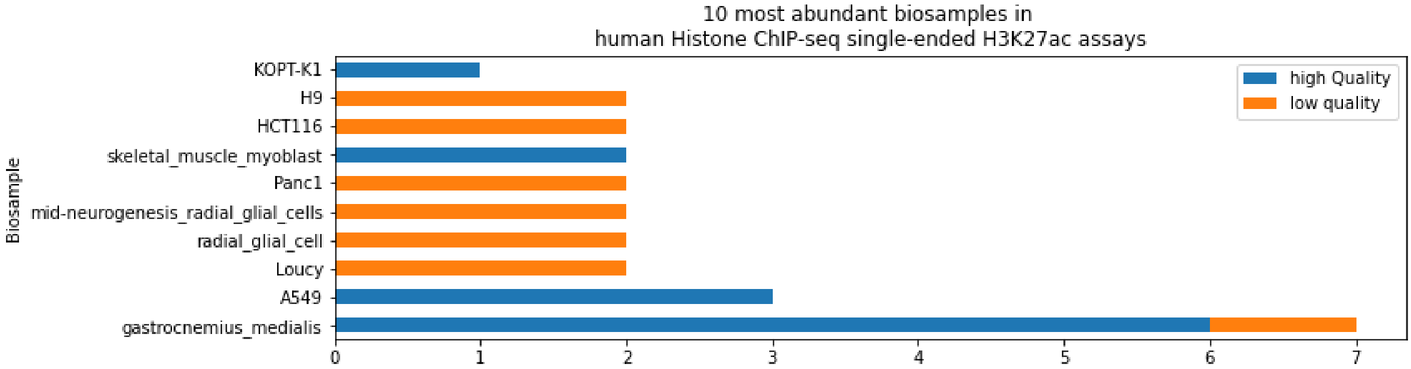

Metrics on training set:

- Accuracy: 1.0

High-quality files:

- Precision: 1.0
- Recall: 1.0
- F1-score: 1.0

Low-quality files

- Precision: 1.0
- Recall: 1.0
- F1-score: 1.0

Legend:

Nodes show data state and decision criteria to apply

Samples by class: [high-quality files, low-quality files]

Group B

human single-ended HistoneChIP-seq for H3K27me3

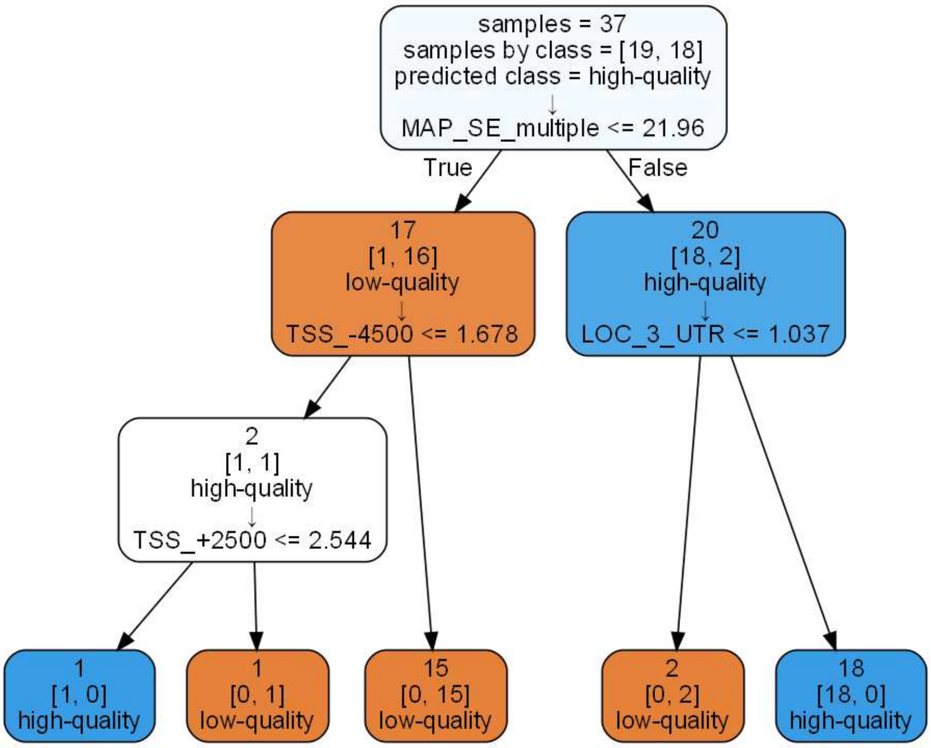

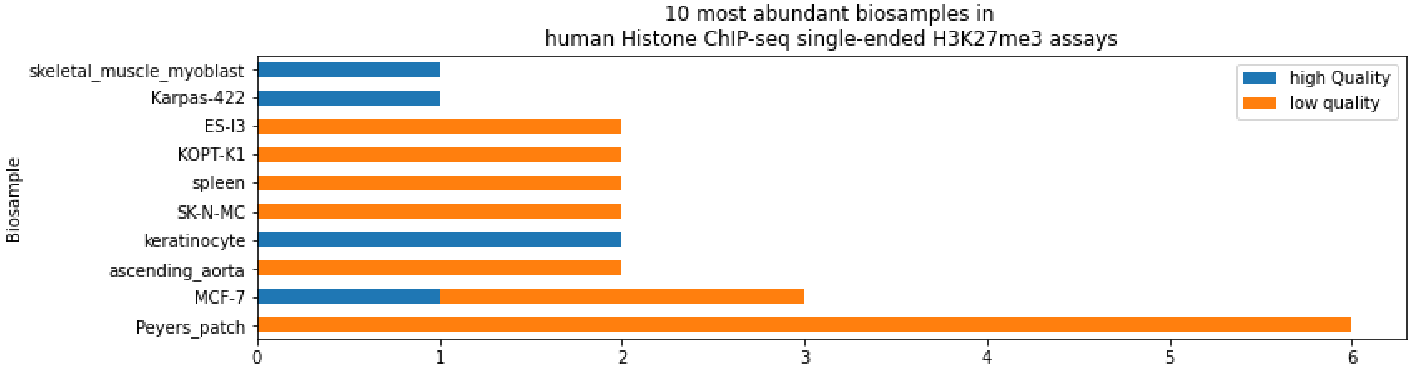

Metrics on training set:
- Accuracy: 1.0

High-quality files:
- Precision: 1.0
- Recall: 1.0
- F1-score: 1.0

Low-quality files
- Precision: 1.0
- Recall: 1.0
- F1-score: 1.0

Legend:
Nodes show data state and decision criteria to apply
Samples by class: [high-quality files, low-quality files]

Group B

human single-ended HistoneChIP-seq for H3K36me3

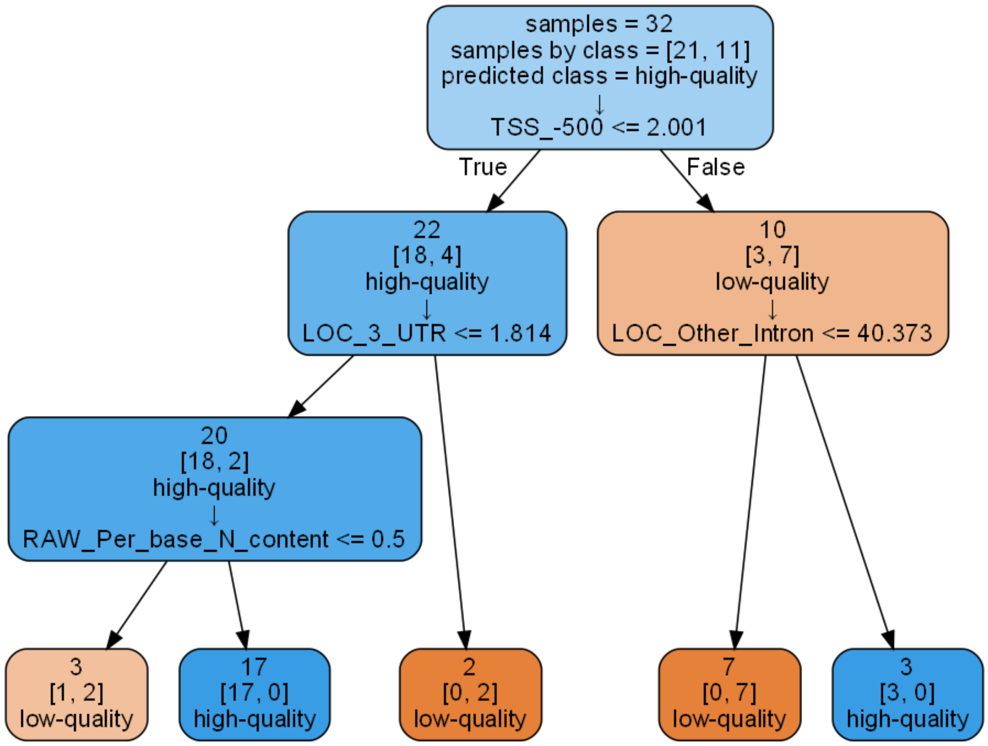

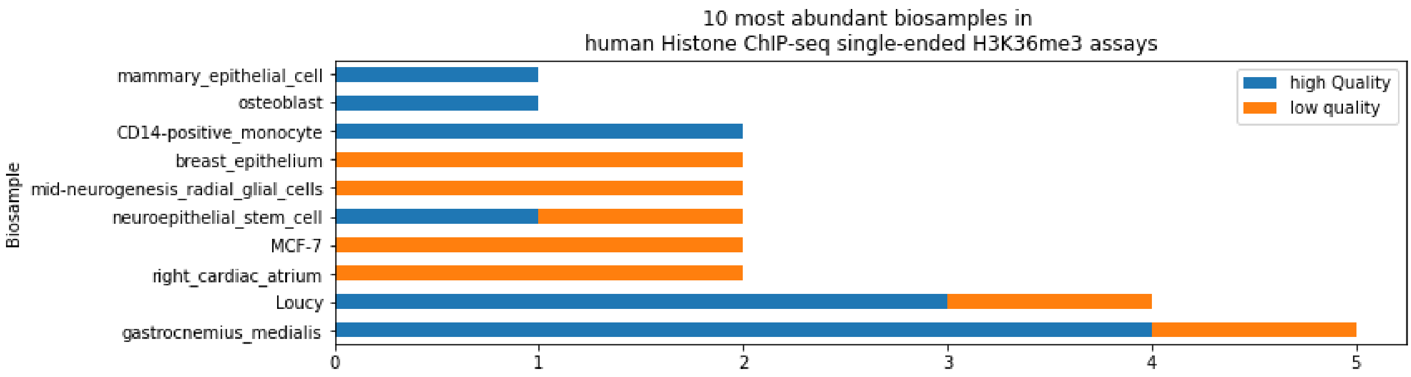

Metrics on training set:

- Accuracy:	0.97
High-quality files:	
- Precision:	1.0
- Recall:	0.95
- F1-score:	0.98
Low-quality files	
- Precision:	0.92
- Recall:	1.0
- F1-score:	0.96

Legend:
Nodes show data state and decision criteria to apply
Samples by class: [high-quality files, low-quality files]

Group B

human single-ended HistoneChIP-seq for H3K4me1

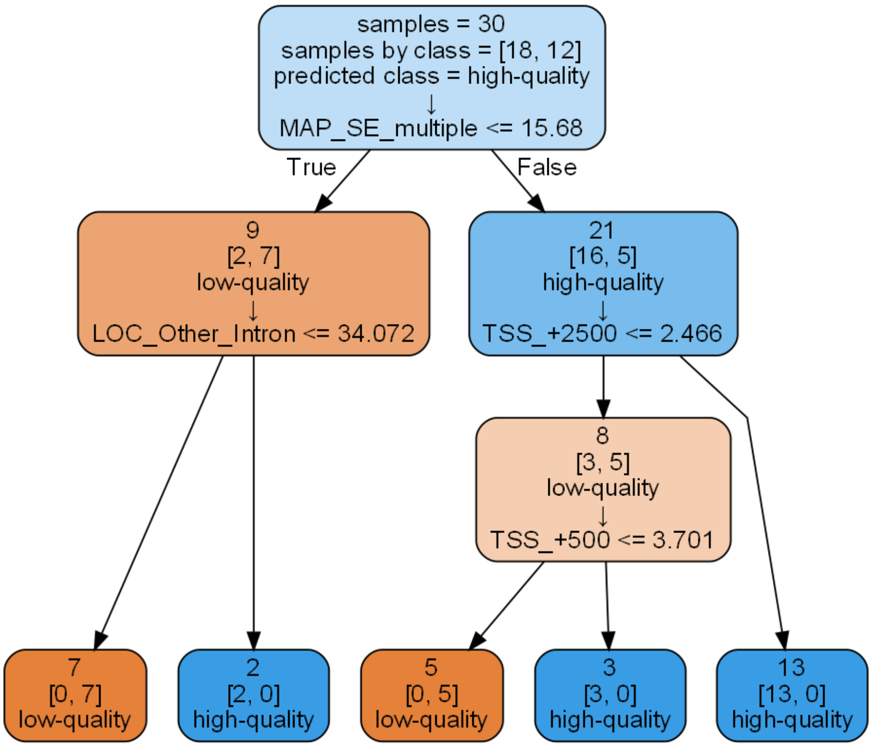

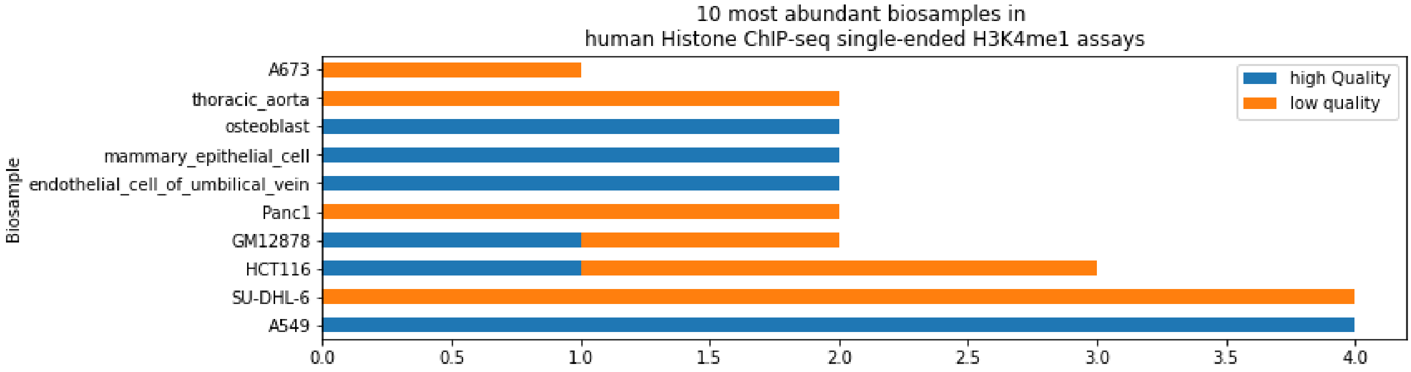

Metrics on training set:

- Accuracy: 1.0

High-quality files:

- Precision: 1.0
- Recall: 1.0
- F1-score: 1.0

Low-quality files

- Precision: 1.0
- Recall: 1.0
- F1-score: 1.0

Legend:

Nodes show data state and decision criteria to apply

Samples by class: [high-quality files, low-quality files]

Group B

human single-ended HistoneChIP-seq for H3K4me2

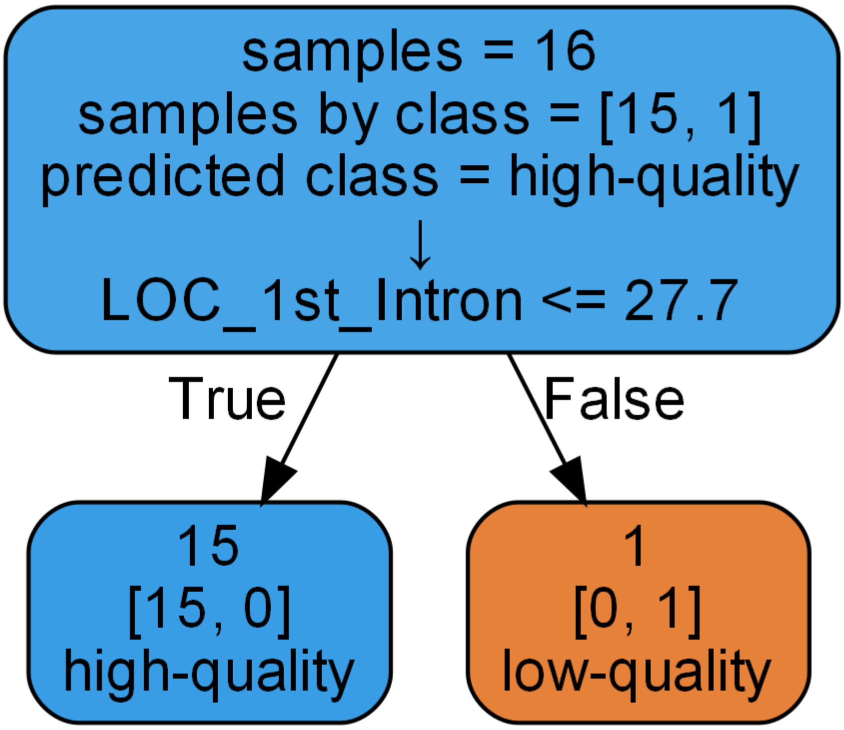

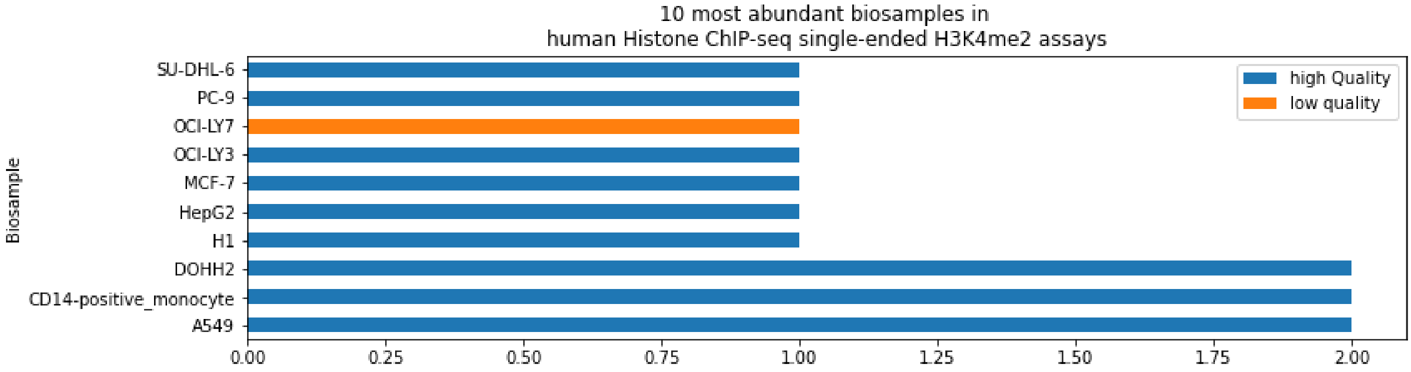

Metrics on training set:

- Accuracy:	1.0
High-quality files:	
- Precision:	1.0
- Recall:	1.0
- F1-score:	1.0
Low-quality files	
- Precision:	1.0
- Recall:	1.0
- F1-score:	1.0

Legend:
Nodes show data state and decision criteria to apply
Samples by class: [high-quality files, low-quality files]

Group B

human single-ended HistoneChIP-seq for H3K4me3

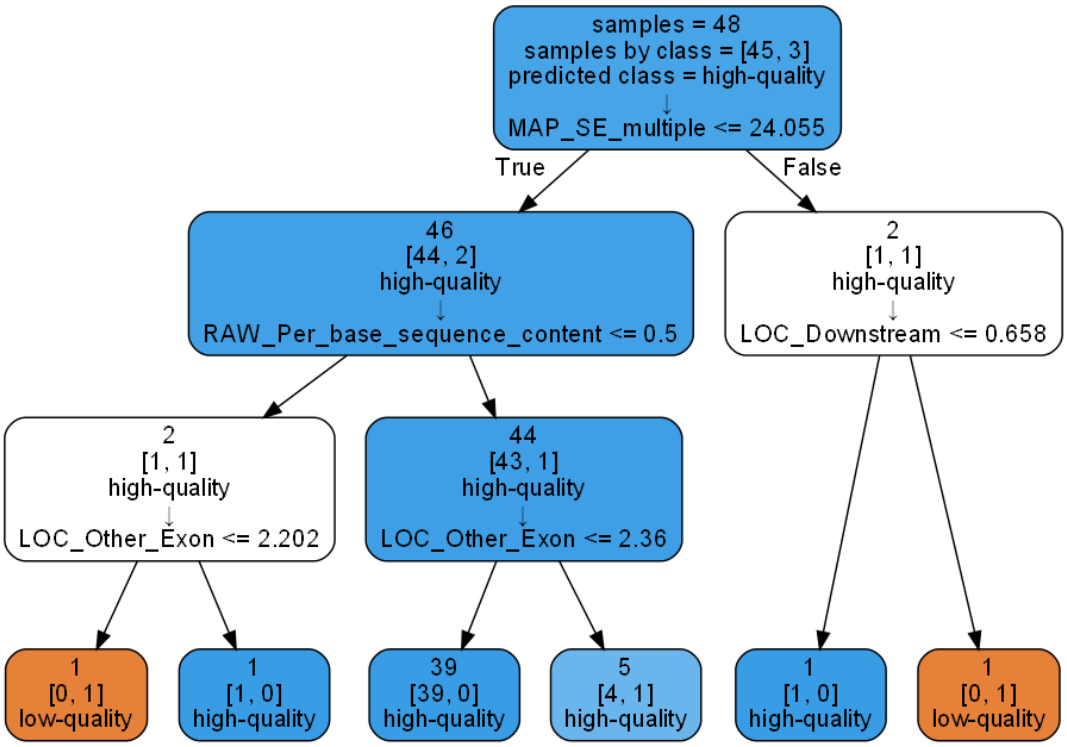

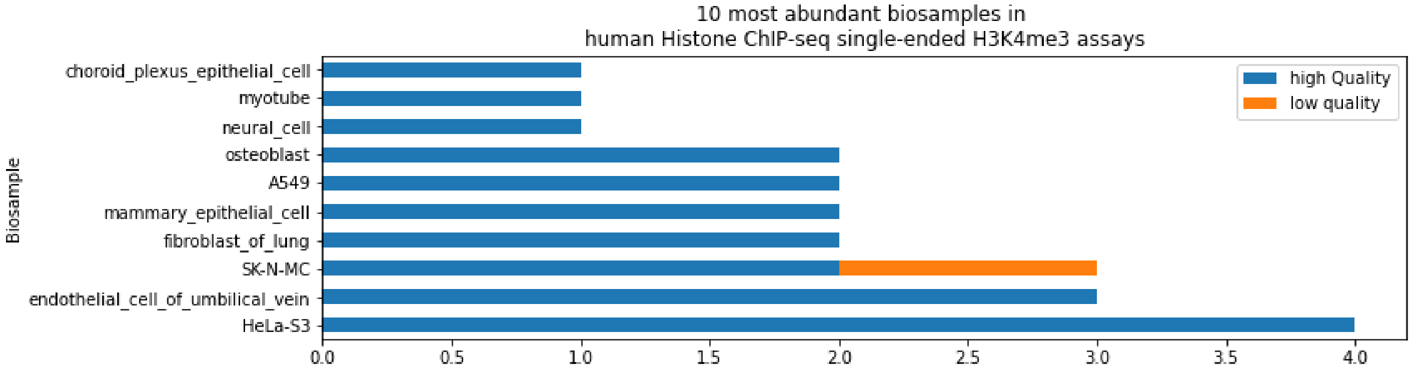

Metrics on training set:

- Accuracy:	0.98
High-quality files:	
- Precision:	0.98
- Recall:	1.0
- F1-score:	0.99
Low-quality files	
- Precision:	1.0
- Recall:	0.67
- F1-score:	0.8

Legend:
Nodes show data state and decision criteria to apply
Samples by class: [high-quality files, low-quality files]

Group B

human single-ended HistoneChIP-seq for H3K9me3

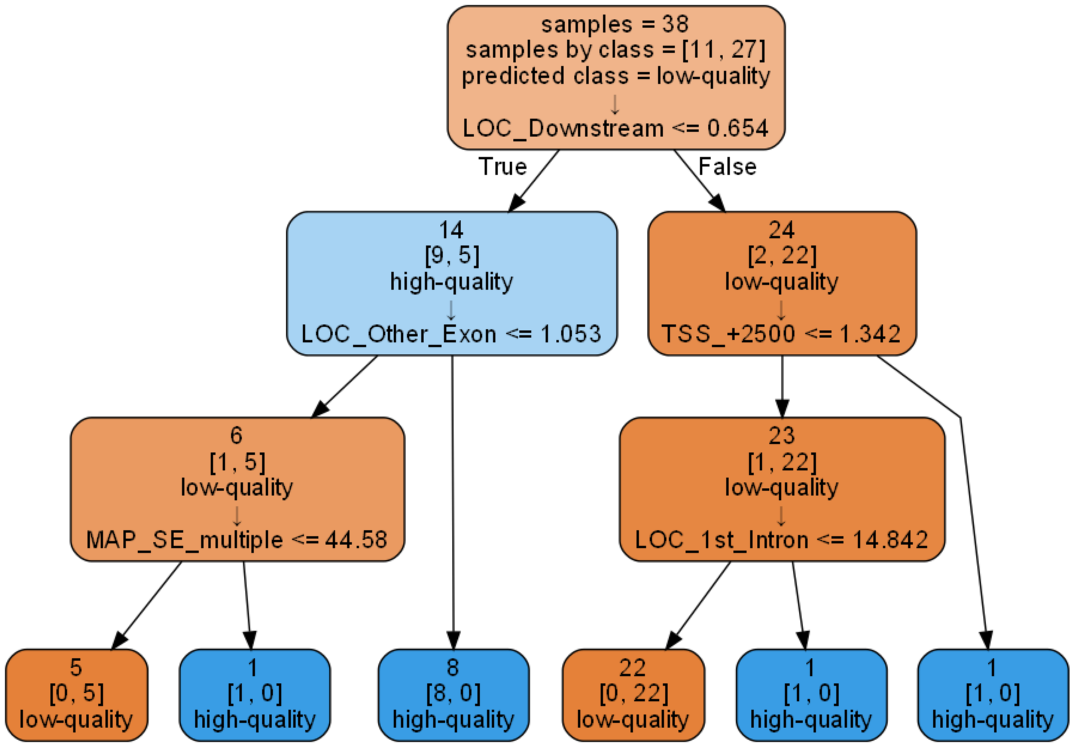

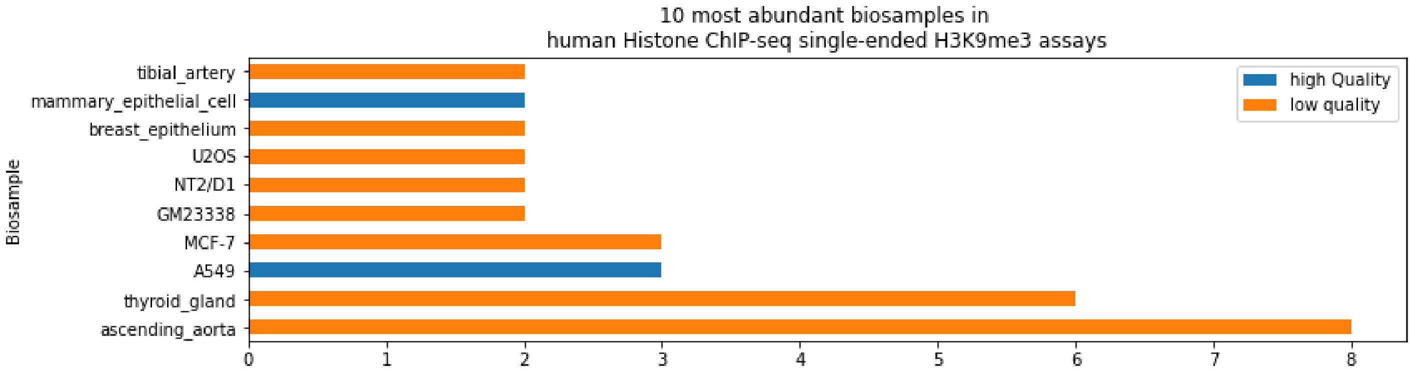

Metrics on training set:
- Accuracy: 1.0

High-quality files:
- Precision: 1.0
- Recall: 1.0
- F1-score: 1.0

Low-quality files
- Precision: 1.0
- Recall: 1.0
- F1-score: 1.0

Legend:
Nodes show data state and decision criteria to apply
Samples by class: [high-quality files, low-quality files]

Group B

human single-ended TFChIP-seq for BACH1

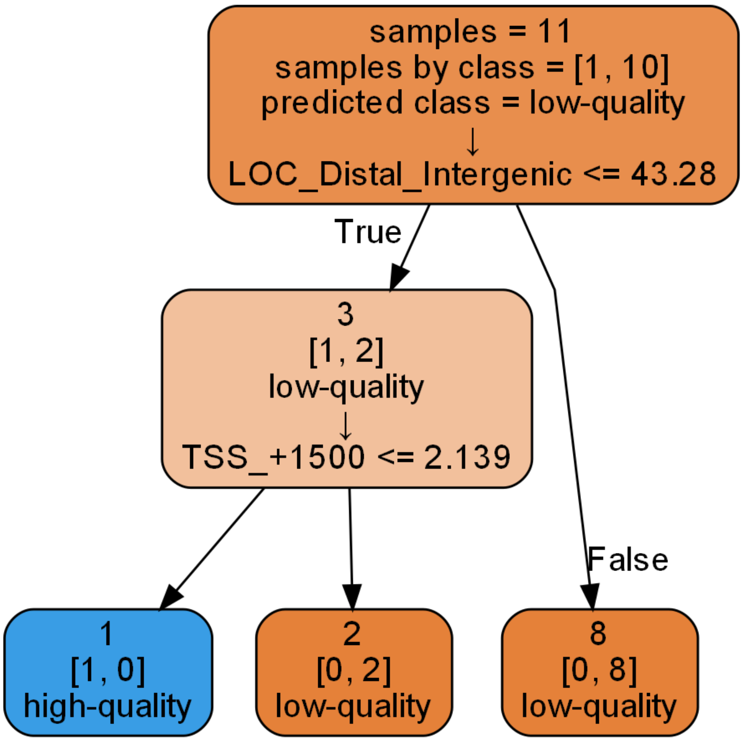

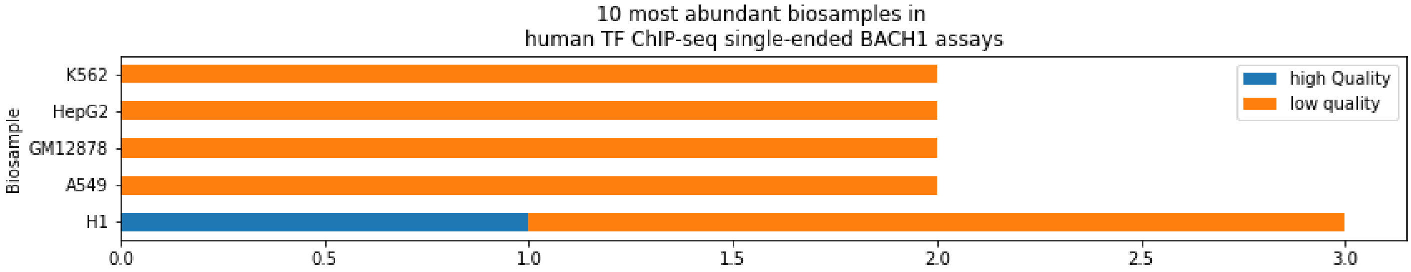

Metrics on training set:
- Accuracy: 1.0

High-quality files:
- Precision: 1.0
- Recall: 1.0
- F1-score: 1.0

Low-quality files
- Precision: 1.0
- Recall: 1.0
- F1-score: 1.0

Legend:

Nodes show data state and decision criteria to apply

Samples by class: [high-quality files, low-quality files]

Group B

human single-ended TFChIP-seq for CHD1

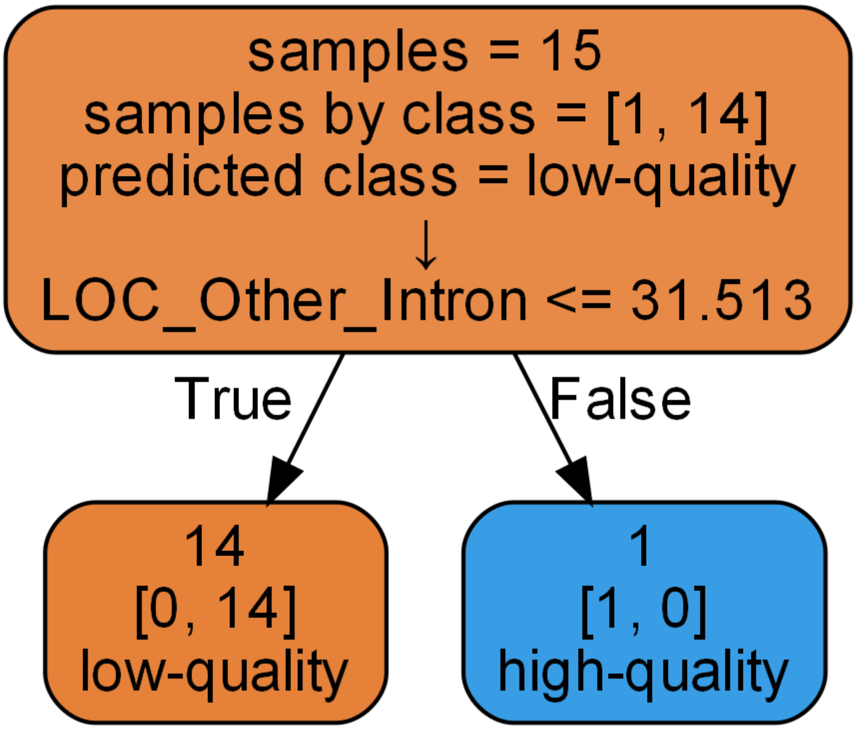

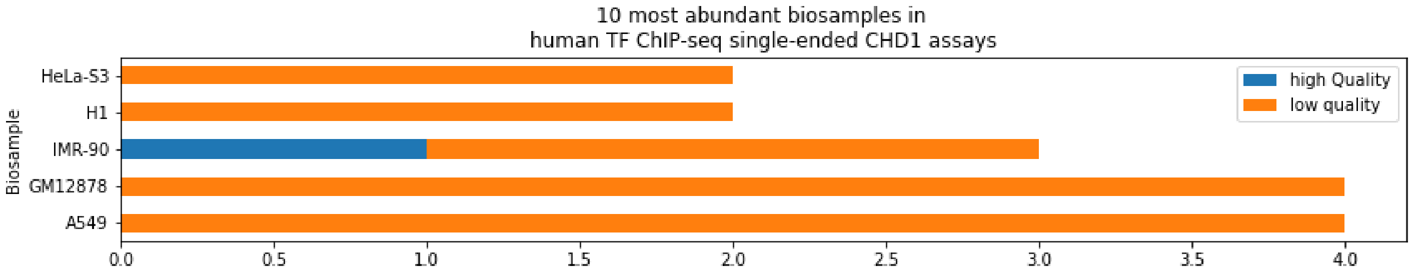

Metrics on training set:

- Accuracy: 1.0

High-quality files:

- Precision: 1.0
- Recall: 1.0
- F1-score: 1.0

Low-quality files

- Precision: 1.0
- Recall: 1.0
- F1-score: 1.0

Legend:

Nodes show data state and decision criteria to apply

Samples by class: [high-quality files, low-quality files]

Group B
human single-ended TFChIP-seq for CTCF

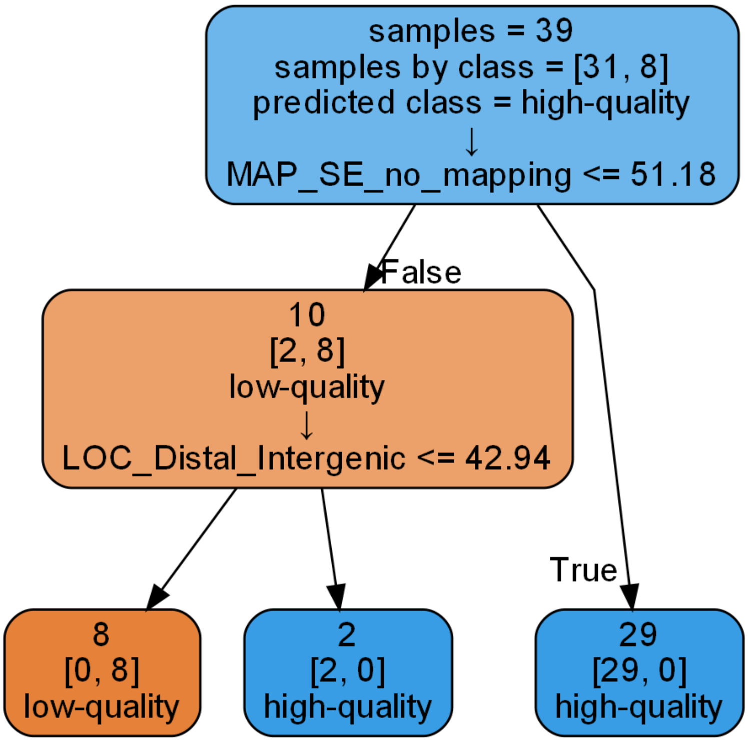

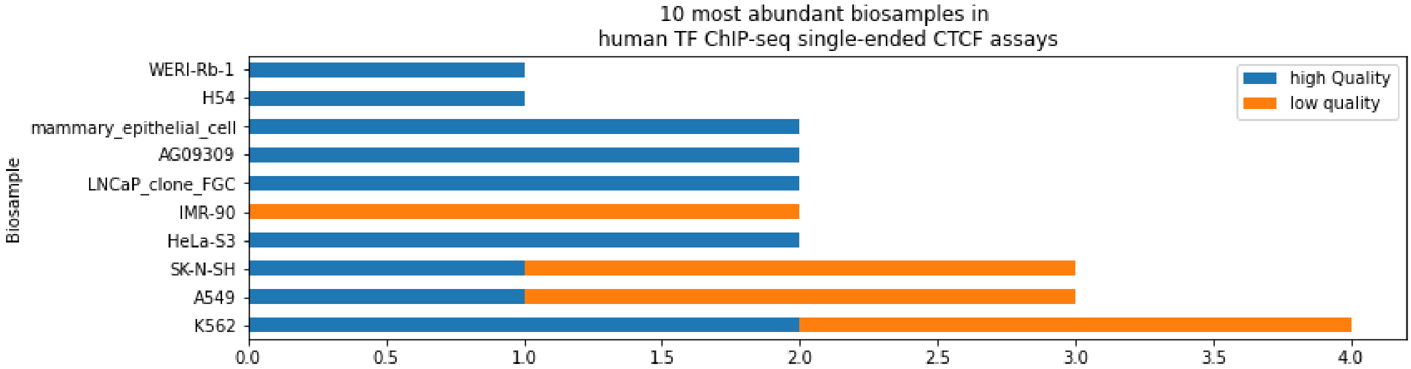

Metrics on training set:
- Accuracy: 1.0

High-quality files:
- Precision: 1.0
- Recall: 1.0
- F1-score: 1.0

Low-quality files
- Precision: 1.0
- Recall: 1.0
- F1-score: 1.0

Legend:
Nodes show data state and decision criteria to apply
Samples by class: [high-quality files, low-quality files]

Group B

human single-ended TFChIP-seq for ELK1

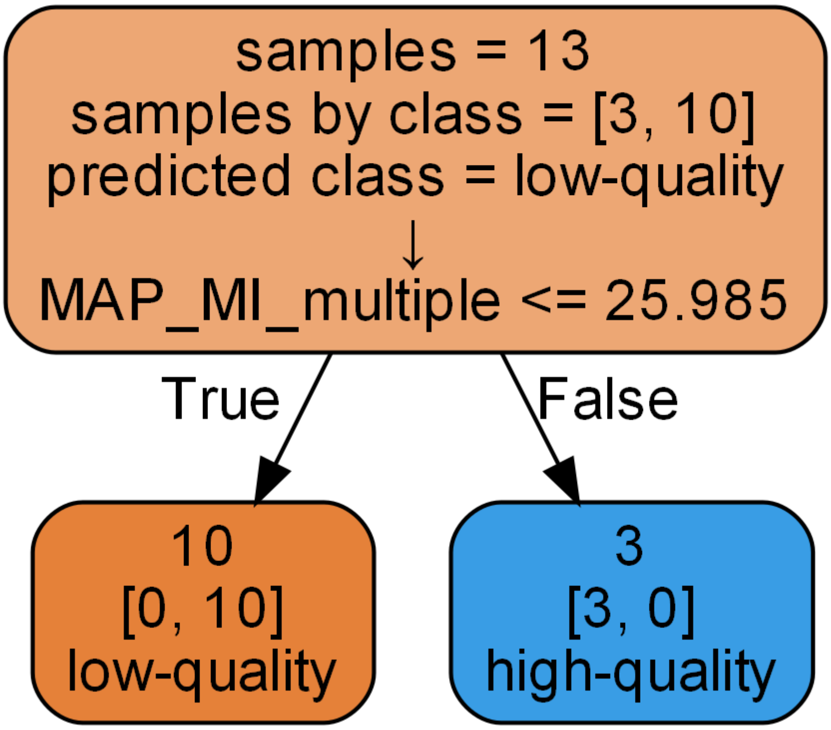

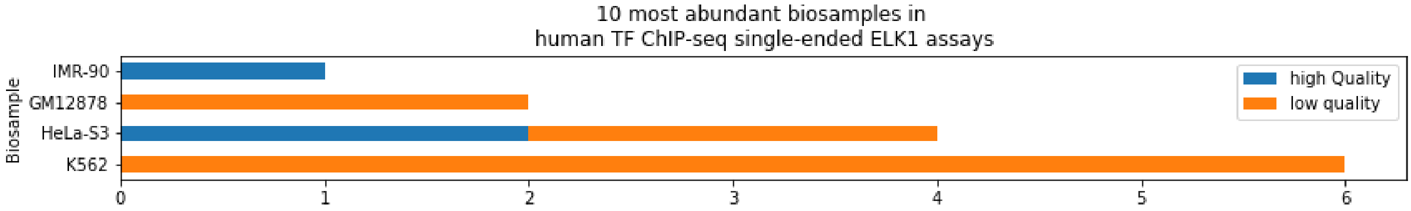

Metrics on training set:

- Accuracy: 1.0

High-quality files:

- Precision: 1.0
- Recall: 1.0
- F1-score: 1.0

Low-quality files

- Precision: 1.0
- Recall: 1.0
- F1-score: 1.0

Legend:

Nodes show data state and decision criteria to apply

Samples by class: [high-quality files, low-quality files]

Group B

human single-ended TFChIP-seq for EP300

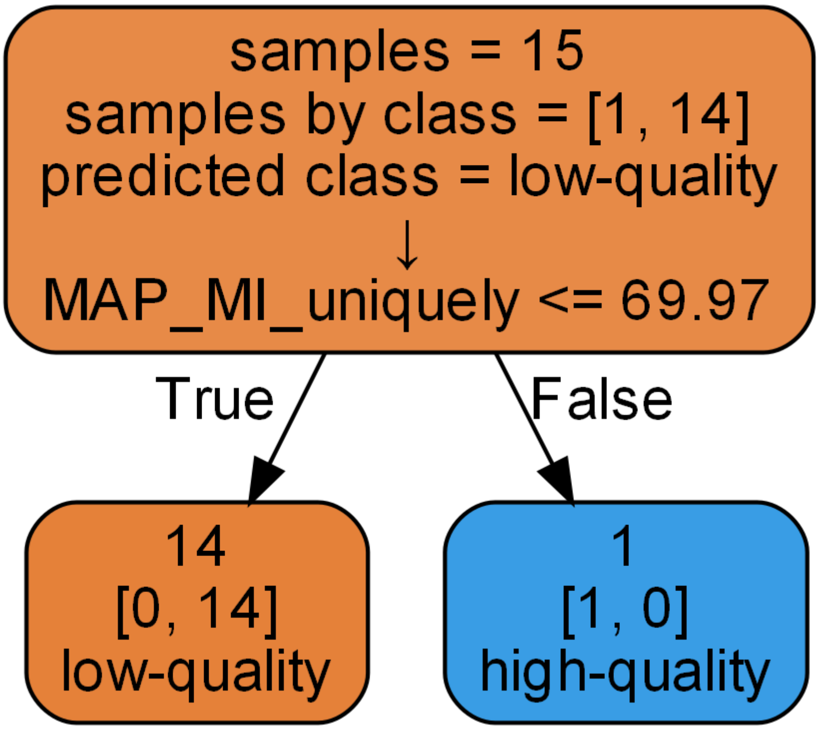

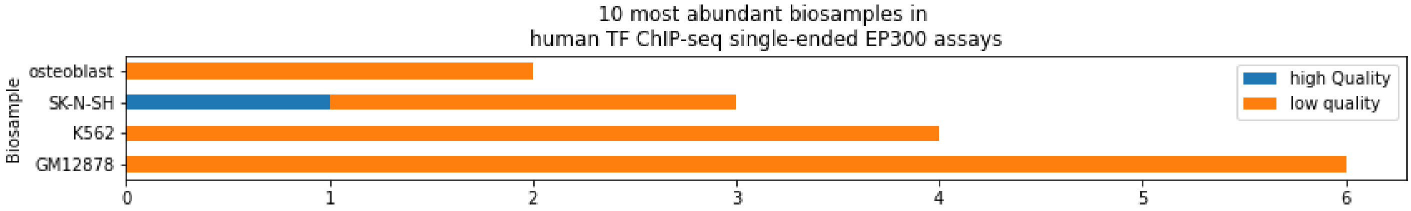

Metrics on training set:

- Accuracy:	1.0
High-quality files:	
- Precision:	1.0
- Recall:	1.0
- F1-score:	1.0
Low-quality files	
- Precision:	1.0
- Recall:	1.0
- F1-score:	1.0

Legend:
Nodes show data state and decision criteria to apply
Samples by class: [high-quality files, low-quality files]

Group B

human single-ended TFChIP-seq for FOS

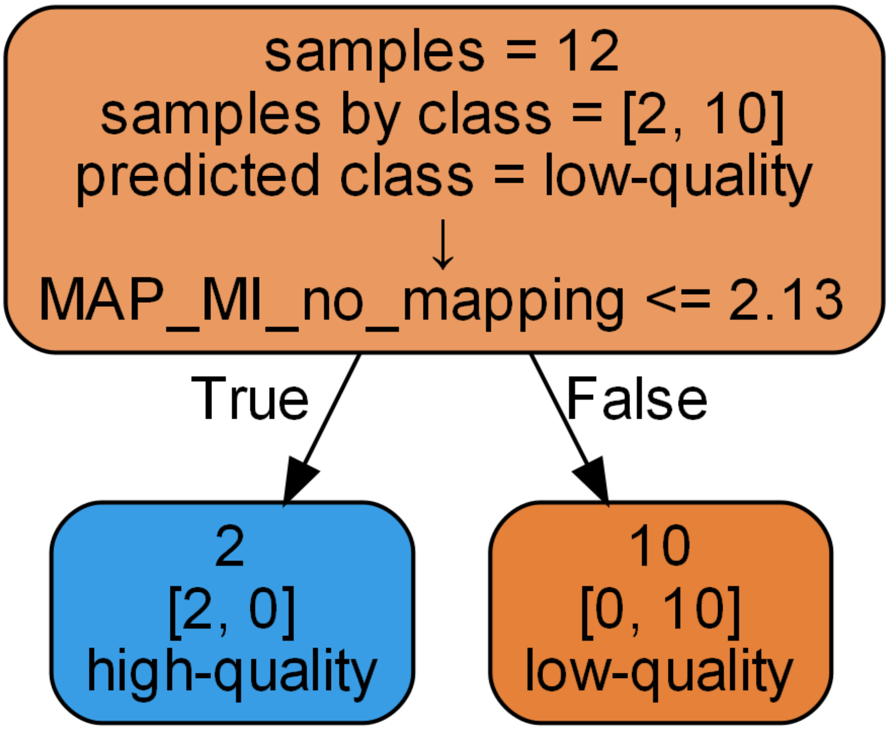

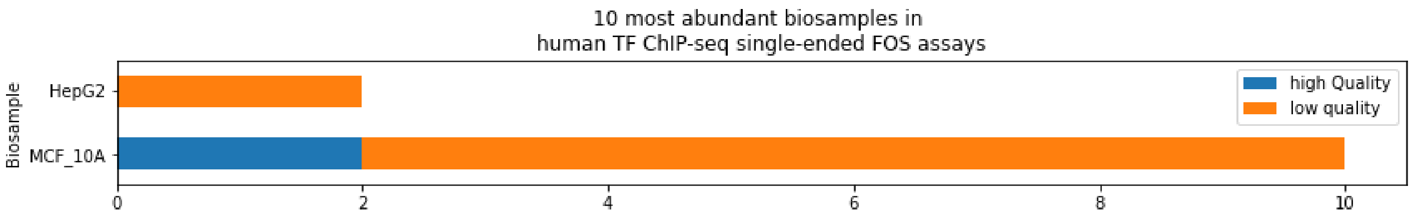

Metrics on training set:
- Accuracy: 1.0

High-quality files:
- Precision: 1.0
- Recall: 1.0
- F1-score: 1.0

Low-quality files
- Precision: 1.0
- Recall: 1.0
- F1-score: 1.0

Legend:
Nodes show data state and decision criteria to apply
Samples by class: [high-quality files, low-quality files]

Group B

human single-ended TFChIP-seq for JUND

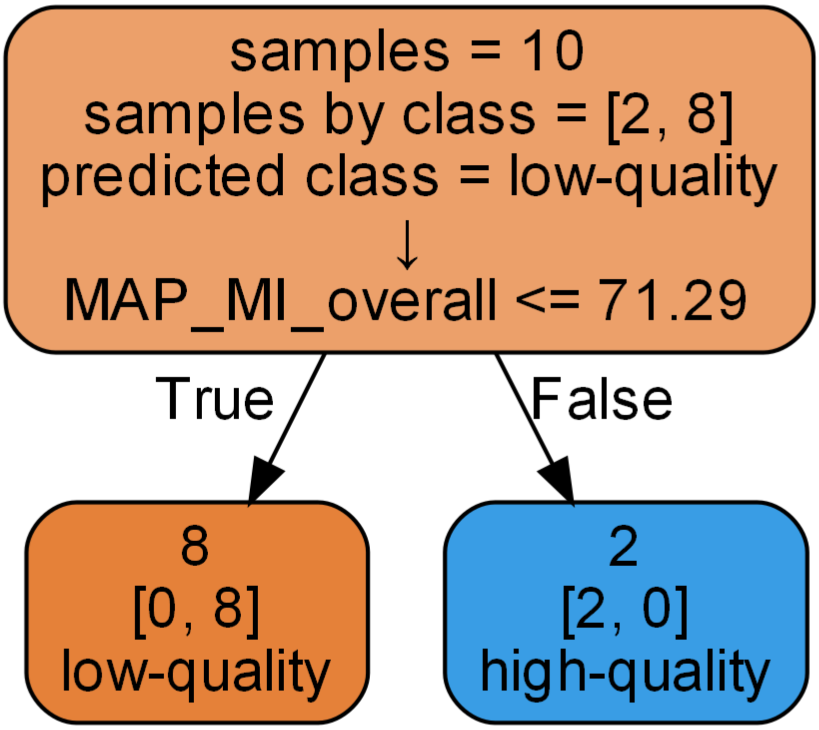

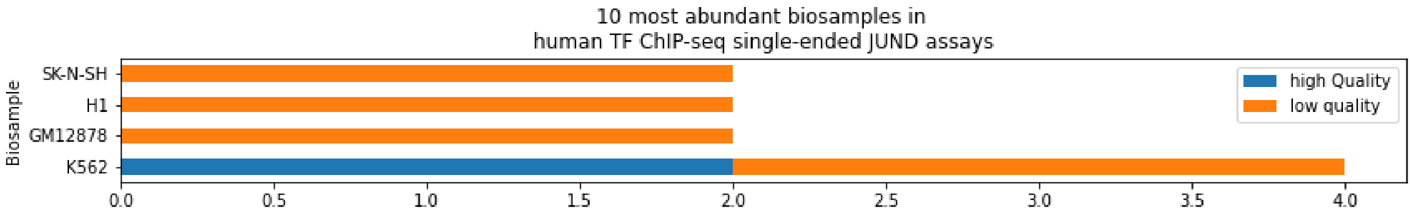

Metrics on training set:
- Accuracy: 1.0

High-quality files:
- Precision: 1.0
- Recall: 1.0
- F1-score: 1.0

Low-quality files
- Precision: 1.0
- Recall: 1.0
- F1-score: 1.0

Legend:
Nodes show data state and decision criteria to apply
Samples by class: [high-quality files, low-quality files]

Group B

human single-ended TFChIP-seq for MAX

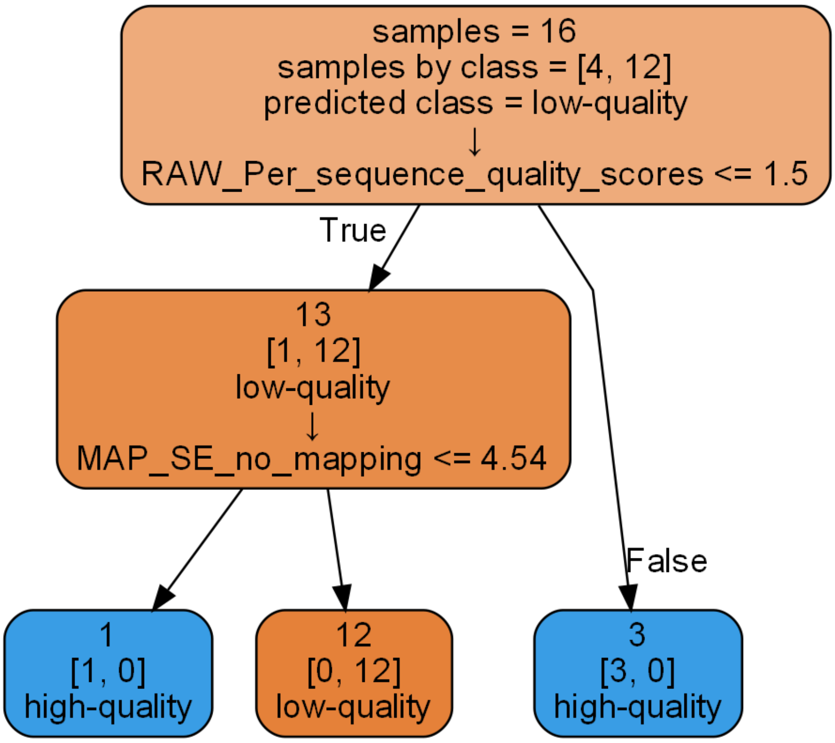

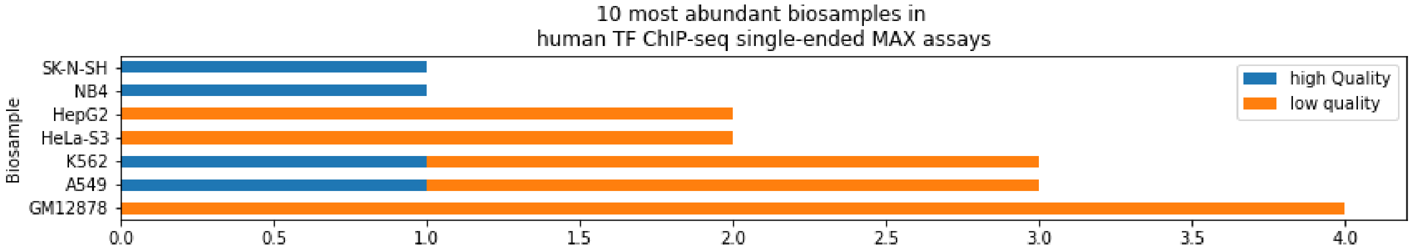

Metrics on training set:

- Accuracy: 1.0

High-quality files:

- Precision: 1.0
- Recall: 1.0
- F1-score: 1.0

Low-quality files

- Precision: 1.0
- Recall: 1.0
- F1-score: 1.0

Legend:

Nodes show data state and decision criteria to apply

Samples by class: [high-quality files, low-quality files]

Group B

human single-ended TFChIP-seq for MAZ

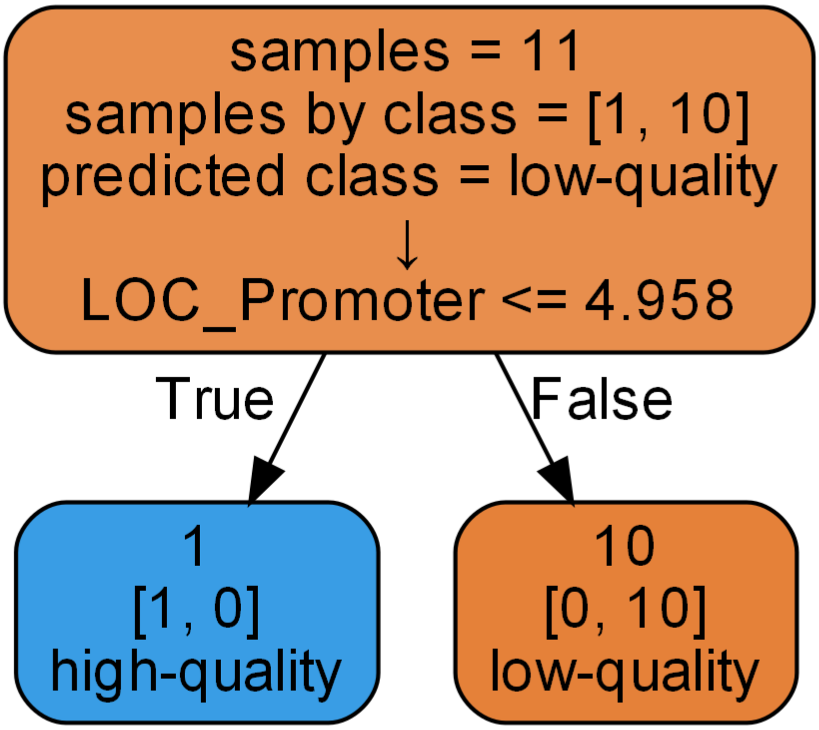

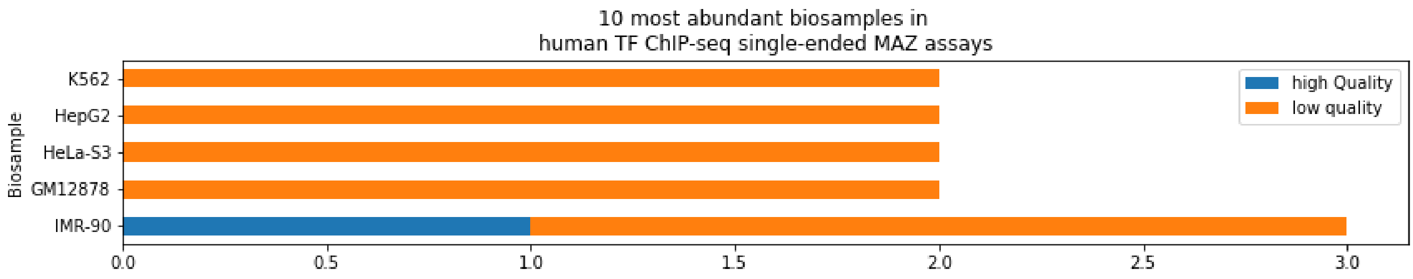

Metrics on training set:

- Accuracy: 1.0

High-quality files:

- Precision: 1.0
- Recall: 1.0
- F1-score: 1.0

Low-quality files

- Precision: 1.0
- Recall: 1.0
- F1-score: 1.0

Legend:

Nodes show data state and decision criteria to apply

Samples by class: [high-quality files, low-quality files]

Group B

human single-ended TFChIP-seq for MXI1

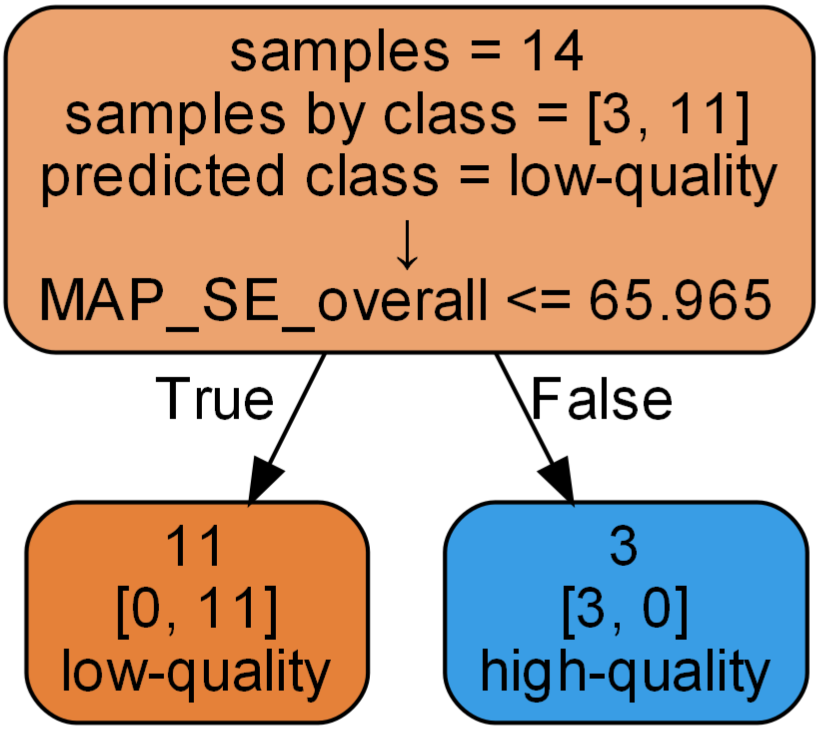

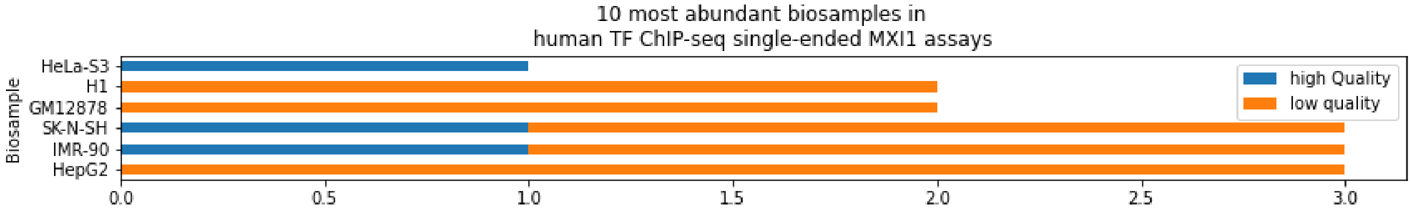

Metrics on training set:

- Accuracy:	1.0
High-quality files:	
- Precision:	1.0
- Recall:	1.0
- F1-score:	1.0
Low-quality files	
- Precision:	1.0
- Recall:	1.0
- F1-score:	1.0

Legend:
Nodes show data state and decision criteria to apply
Samples by class: [high-quality files, low-quality files]

Group B

human single-ended TFChIP-seq for MYC

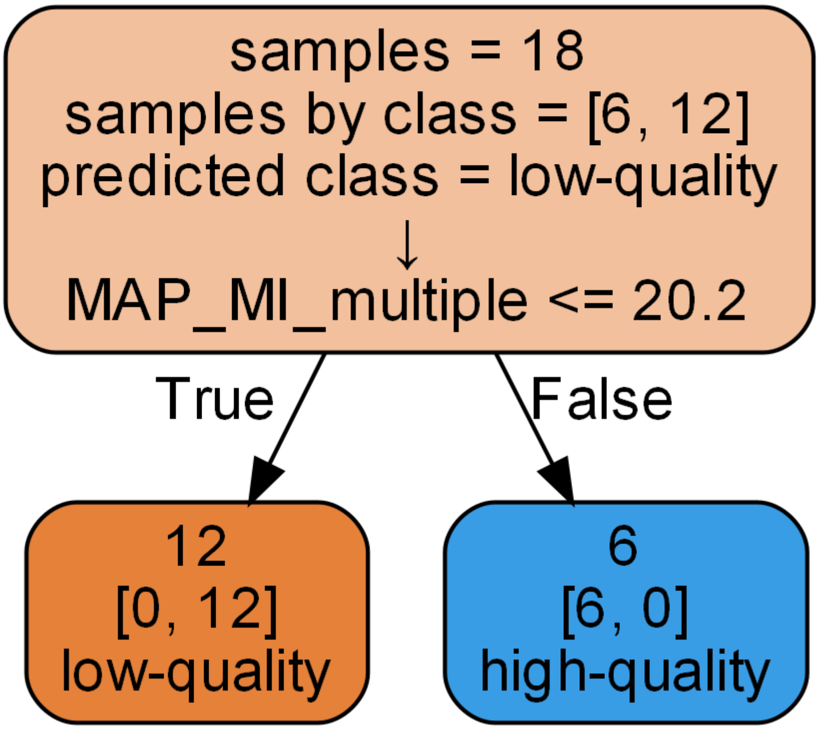

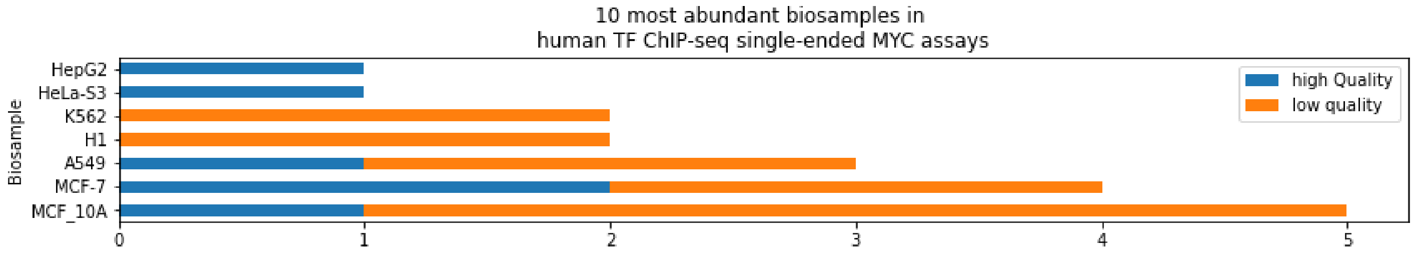

Metrics on training set:
- Accuracy: 1.0

High-quality files:
- Precision: 1.0
- Recall: 1.0
- F1-score: 1.0

Low-quality files
- Precision: 1.0
- Recall: 1.0
- F1-score: 1.0

Legend:
Nodes show data state and decision criteria to apply
Samples by class: [high-quality files, low-quality files]

Group B

human single-ended TFChIP-seq for NR3C1

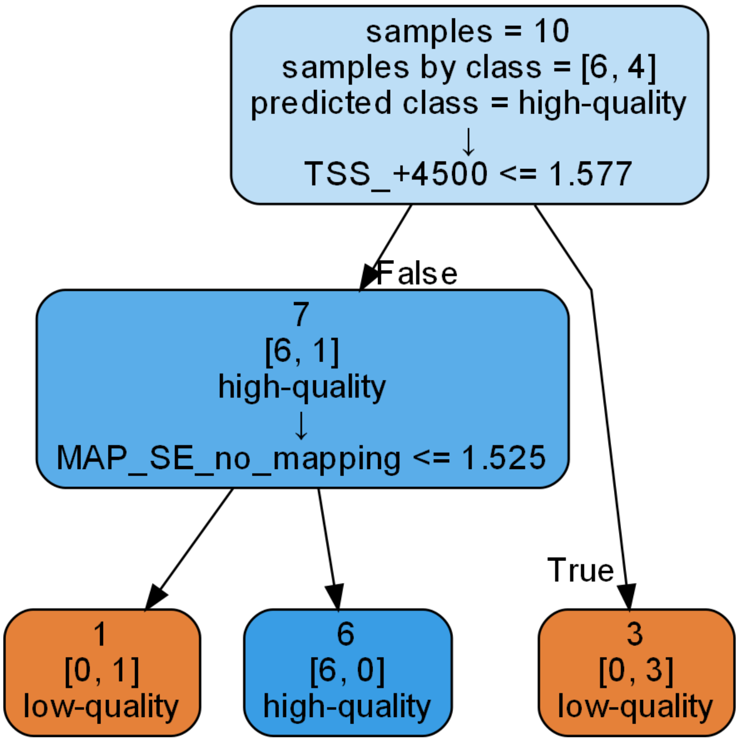

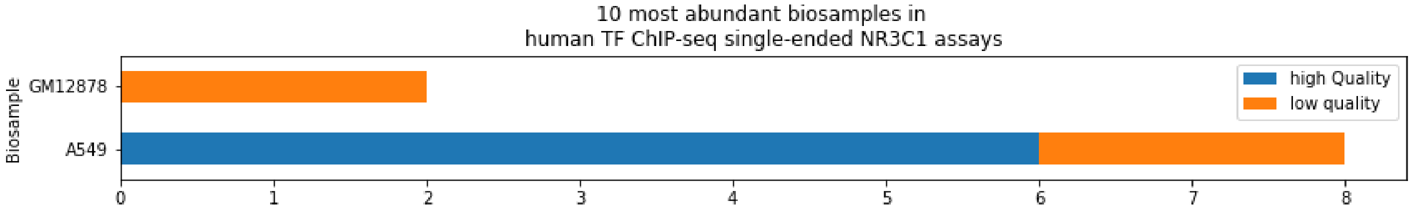

Metrics on training set:
- Accuracy: 1.0

High-quality files:
- Precision: 1.0
- Recall: 1.0
- F1-score: 1.0

Low-quality files
- Precision: 1.0
- Recall: 1.0
- F1-score: 1.0

Legend:
Nodes show data state and decision criteria to apply
Samples by class: [high-quality files, low-quality files]

Group B

human single-ended TFChIP-seq for POLR2A

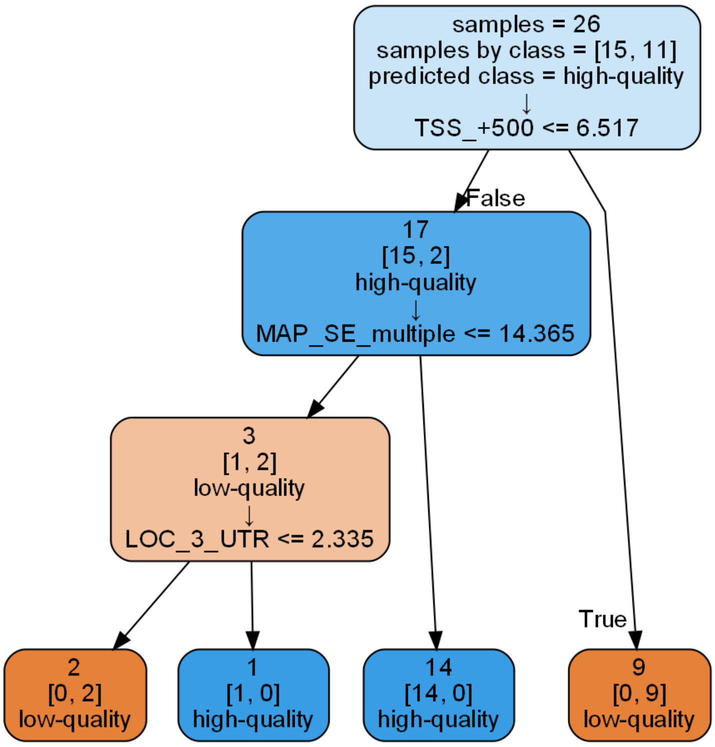

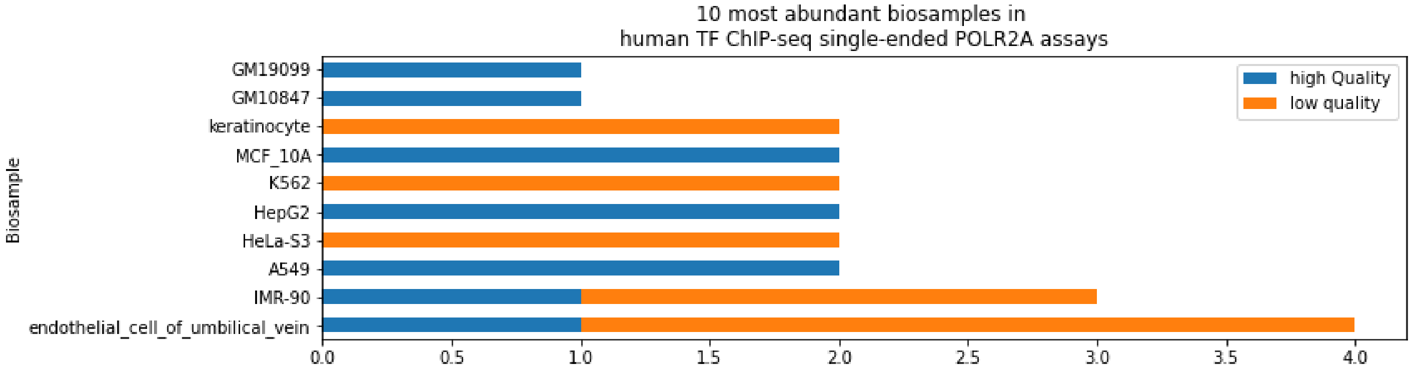

Metrics on training set:

- Accuracy: 1.0

High-quality files:

- Precision: 1.0
- Recall: 1.0
- F1-score: 1.0

Low-quality files

- Precision: 1.0
- Recall: 1.0
- F1-score: 1.0

Legend:

Nodes show data state and decision criteria to apply

Samples by class: [high-quality files, low-quality files]

Group B

human single-ended TFChIP-seq for RCOR1

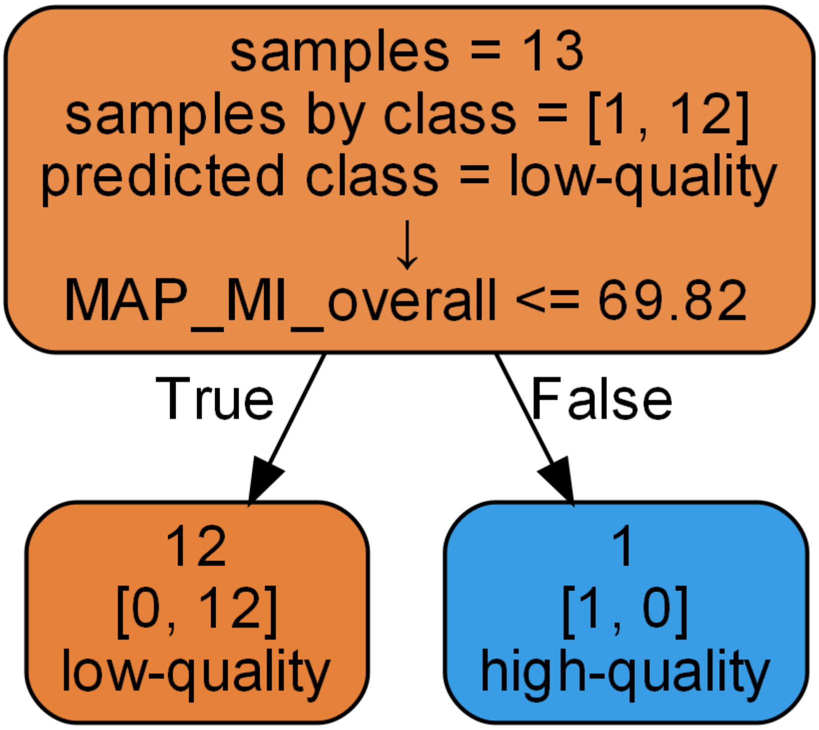

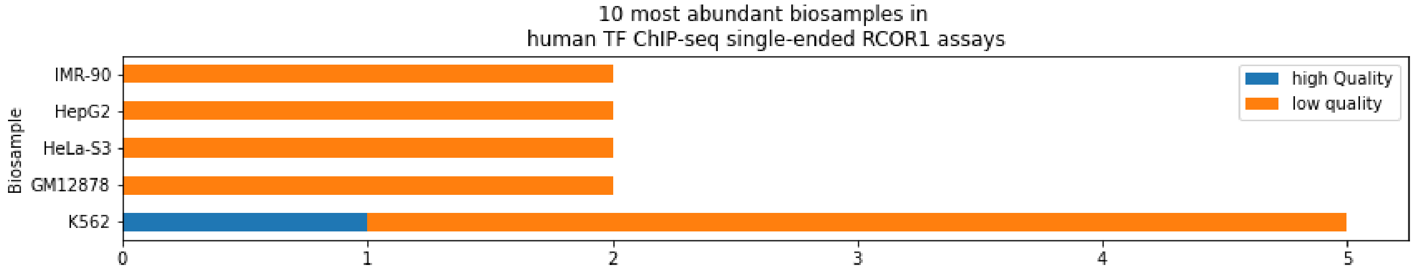

Metrics on training set:

- Accuracy: 1.0

High-quality files:

- Precision: 1.0
- Recall: 1.0
- F1-score: 1.0

Low-quality files

- Precision: 1.0
- Recall: 1.0
- F1-score: 1.0

Legend:

Nodes show data state and decision criteria to apply

Samples by class: [high-quality files, low-quality files]

Group B

human single-ended TFChIP-seq for RELA

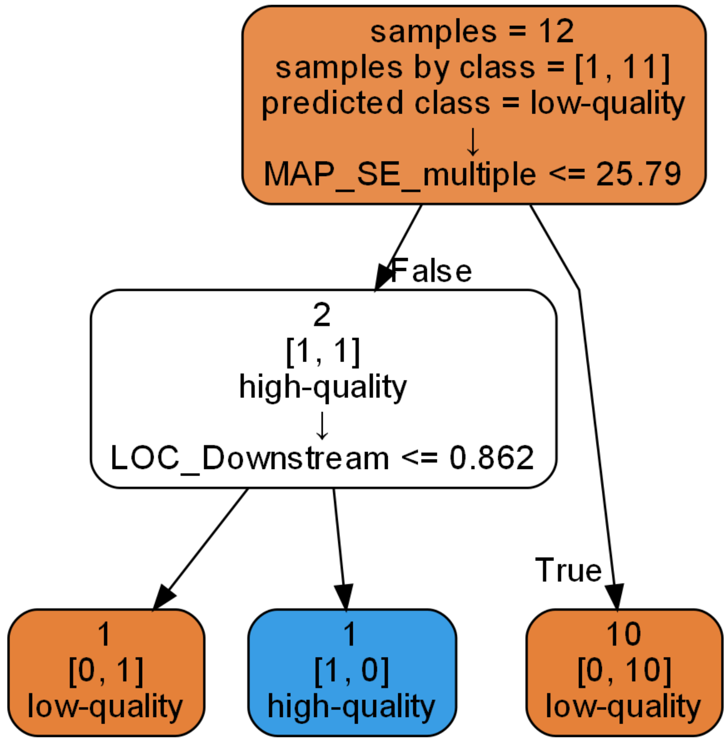

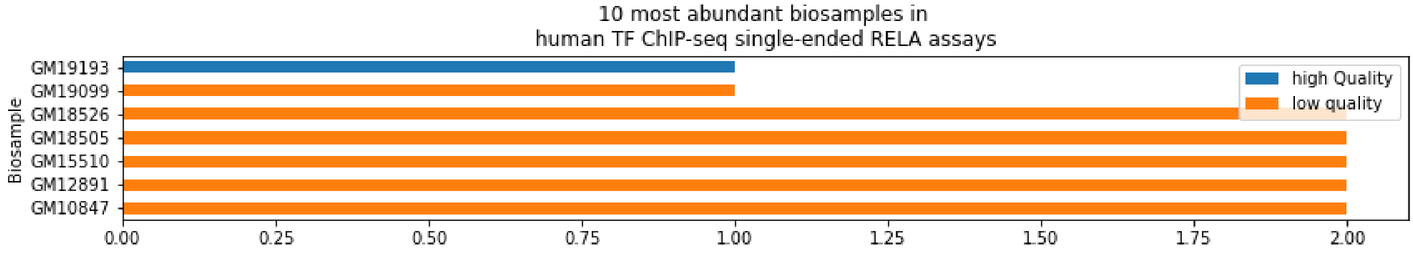

Metrics on training set:
- Accuracy: 1.0

High-quality files:
- Precision: 1.0
- Recall: 1.0
- F1-score: 1.0

Low-quality files
- Precision: 1.0
- Recall: 1.0
- F1-score: 1.0

Legend:
Nodes show data state and decision criteria to apply
Samples by class: [high-quality files, low-quality files]

Group B

human single-ended TFChIP-seq for STAT3

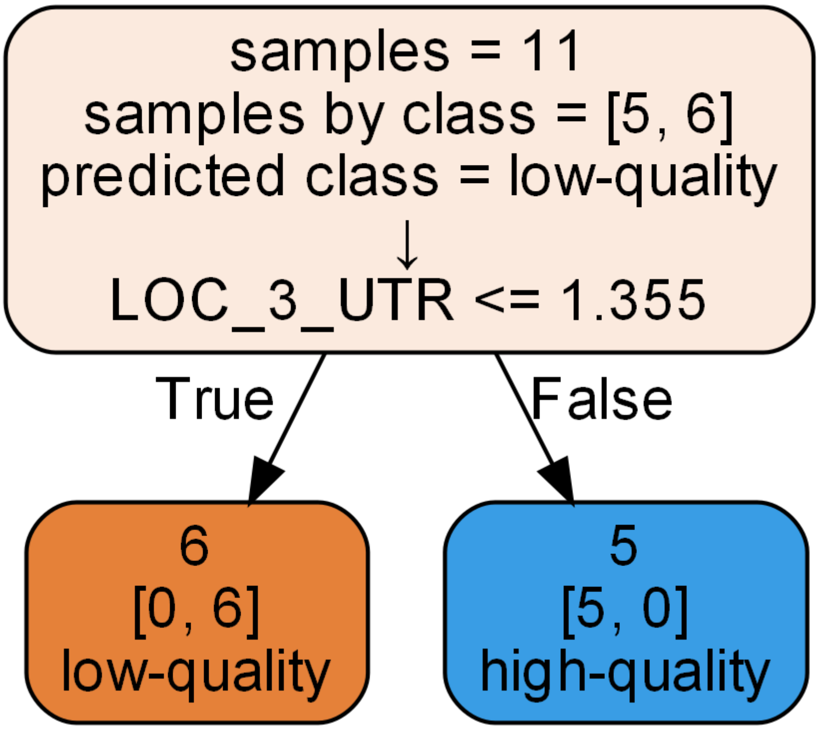

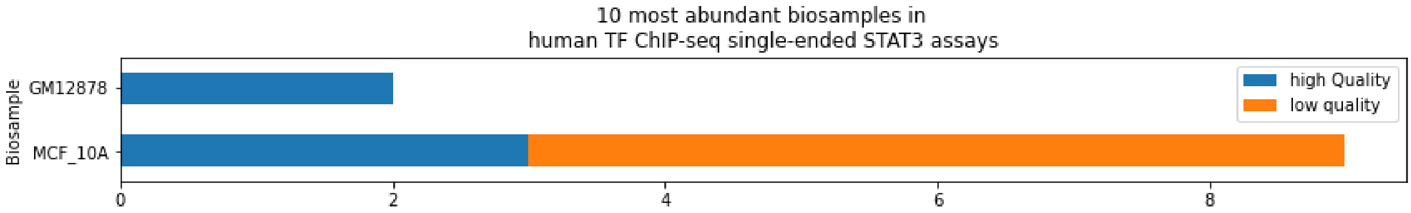

Metrics on training set:

- Accuracy: 1.0

High-quality files:

- Precision: 1.0
- Recall: 1.0
- F1-score: 1.0

Low-quality files

- Precision: 1.0
- Recall: 1.0
- F1-score: 1.0

Legend:

Nodes show data state and decision criteria to apply

Samples by class: [high-quality files, low-quality files]

Group B

human single-ended TFChIP-seq for TCF7L2

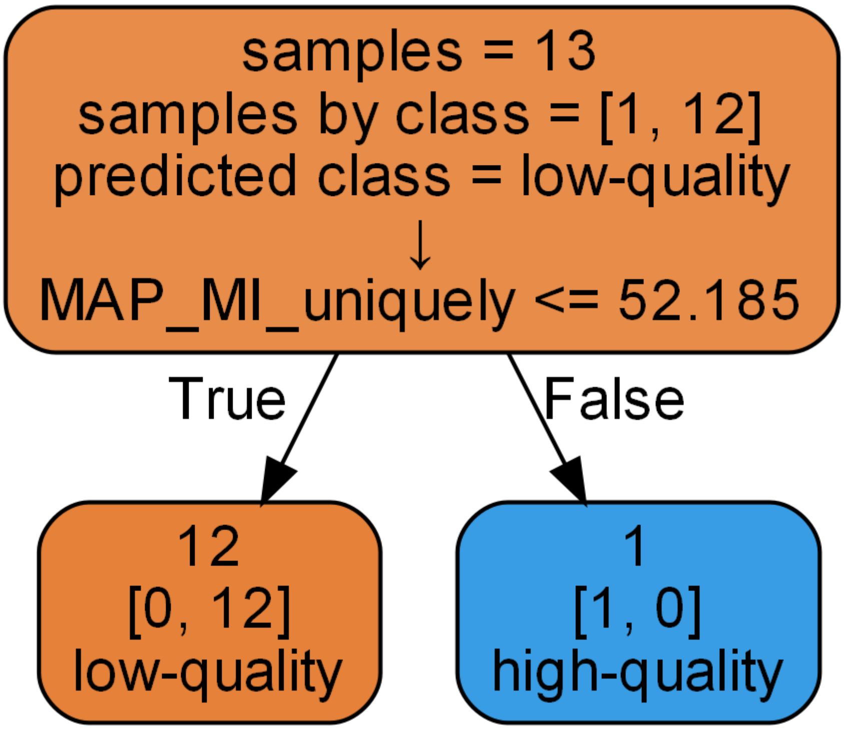

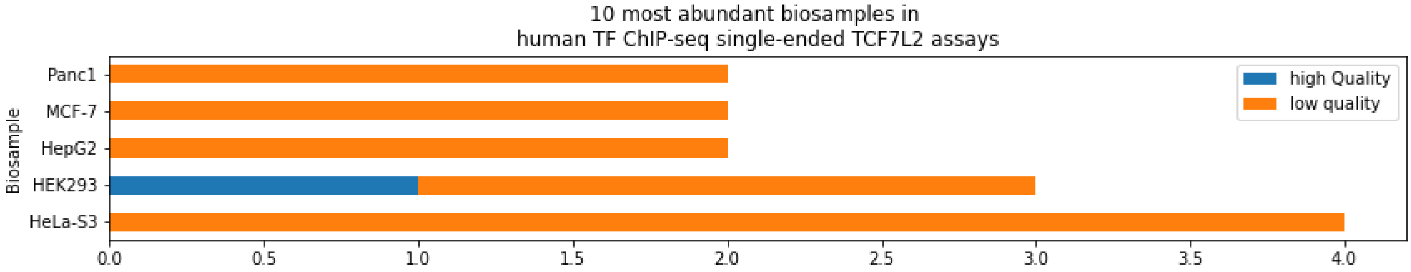

Metrics on training set:
- Accuracy: 1.0

High-quality files:
- Precision: 1.0
- Recall: 1.0
- F1-score: 1.0

Low-quality files
- Precision: 1.0
- Recall: 1.0
- F1-score: 1.0

Legend:
Nodes show data state and decision criteria to apply
Samples by class: [high-quality files, low-quality files]

Group B

mouse single-ended ControlChIP-seq for Control

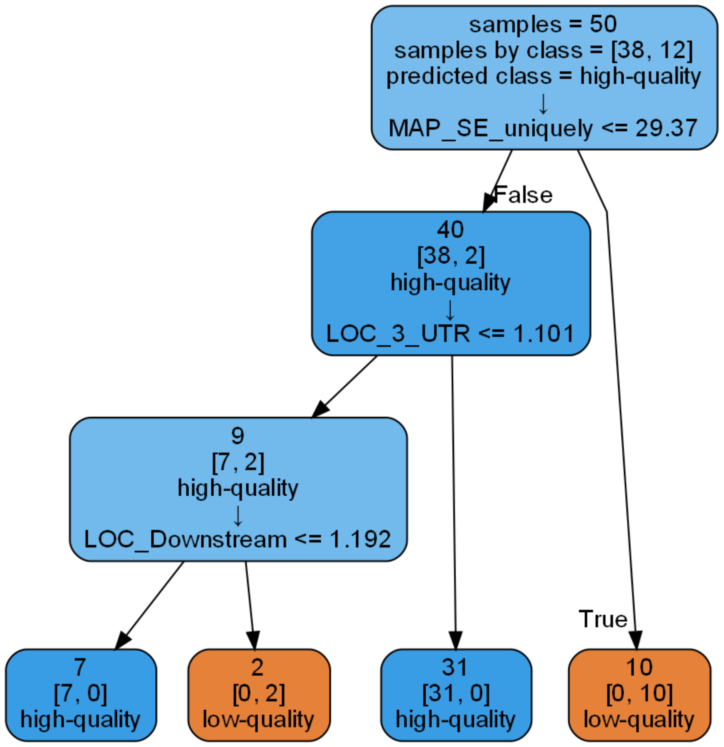

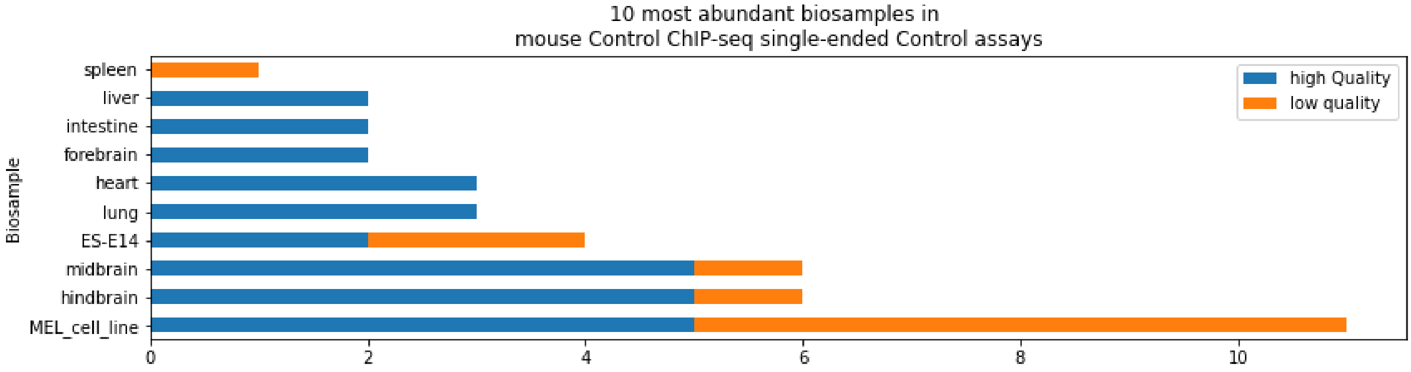

Metrics on training set:

- Accuracy: 1.0

High-quality files:

- Precision: 1.0
- Recall: 1.0
- F1-score: 1.0

Low-quality files

- Precision: 1.0
- Recall: 1.0
- F1-score: 1.0

Legend:

Nodes show data state and decision criteria to apply

Samples by class: [high-quality files, low-quality files]

Group B

mouse single-ended HistoneChIP-seq for H3K27ac

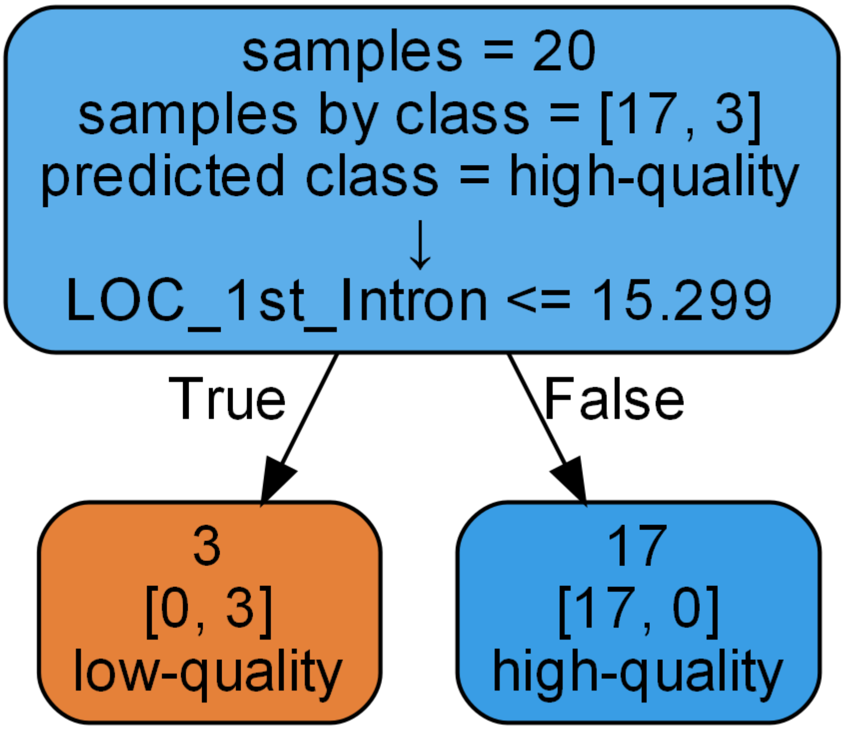

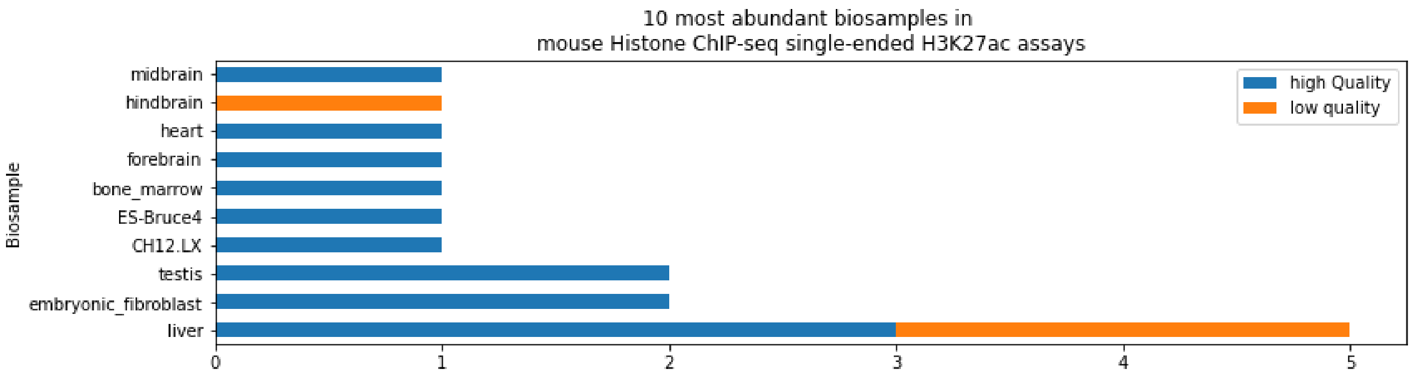

Metrics on training set:

- Accuracy: 1.0

High-quality files:

- Precision: 1.0
- Recall: 1.0
- F1-score: 1.0

Low-quality files

- Precision: 1.0
- Recall: 1.0
- F1-score: 1.0

Legend:

Nodes show data state and decision criteria to apply

Samples by class: [high-quality files, low-quality files]

Group B

mouse single-ended HistoneChIP-seq for H3K27me3

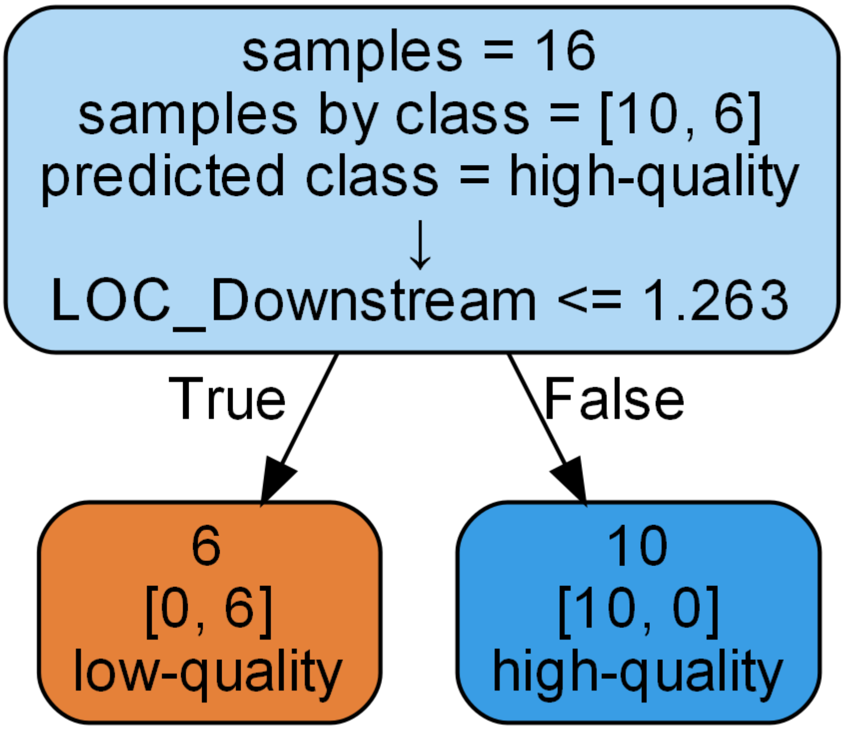

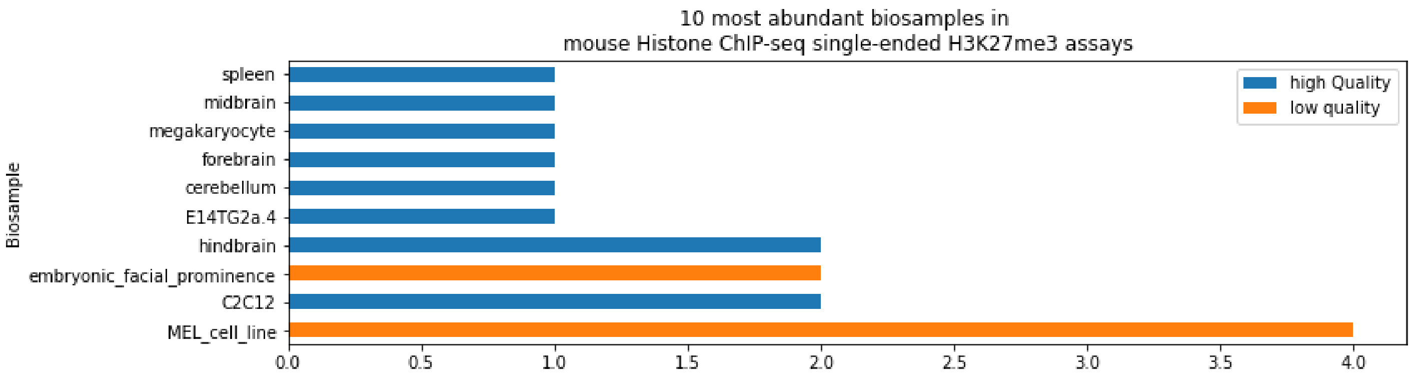

Metrics on training set:

- Accuracy: 1.0

High-quality files:

- Precision: 1.0
- Recall: 1.0
- F1-score: 1.0

Low-quality files

- Precision: 1.0
- Recall: 1.0
- F1-score: 1.0

Legend:

Nodes show data state and decision criteria to apply

Samples by class: [high-quality files, low-quality files]

Group B

mouse single-ended HistoneChIP-seq for H3K36me3

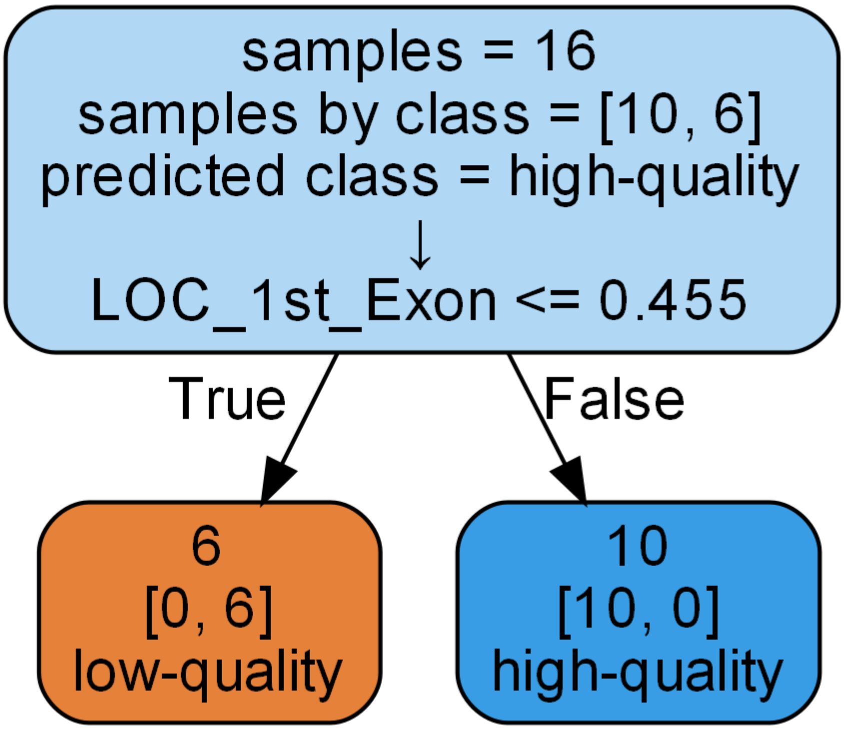

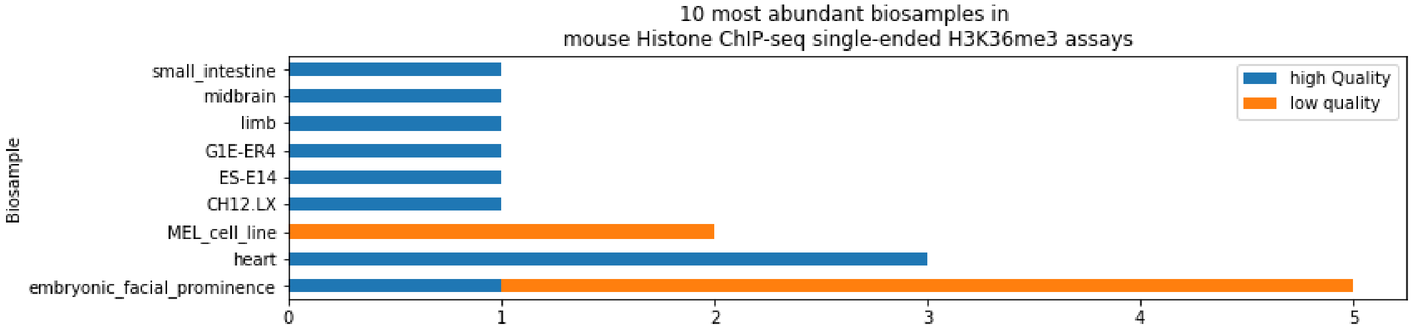

Metrics on training set:
- Accuracy: 1.0

High-quality files:
- Precision: 1.0
- Recall: 1.0
- F1-score: 1.0

Low-quality files
- Precision: 1.0
- Recall: 1.0
- F1-score: 1.0

Legend:
Nodes show data state and decision criteria to apply
Samples by class: [high-quality files, low-quality files]

Group B

mouse single-ended HistoneChIP-seq for H3K4me1

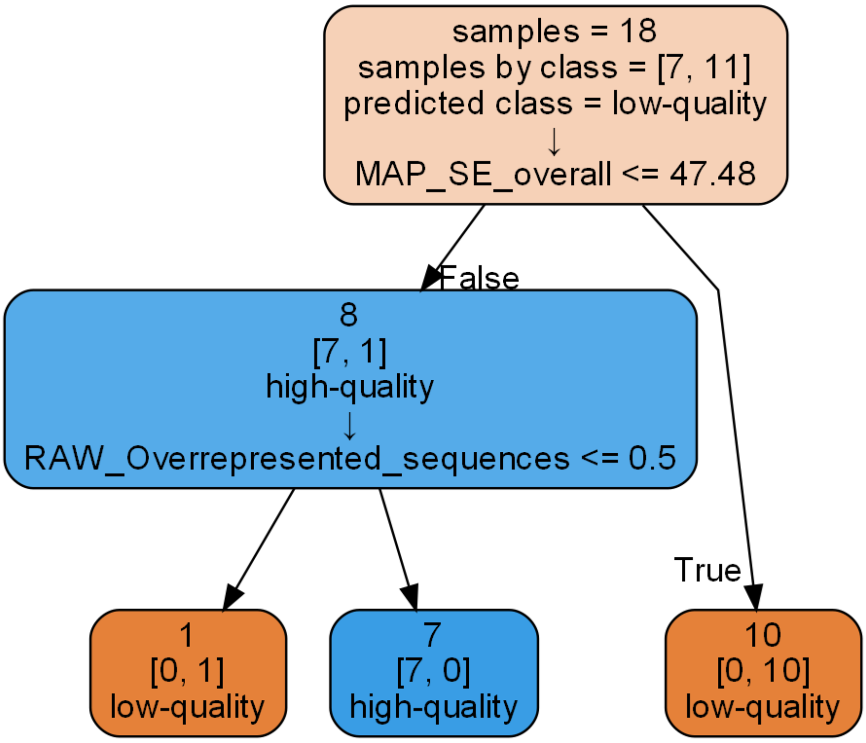

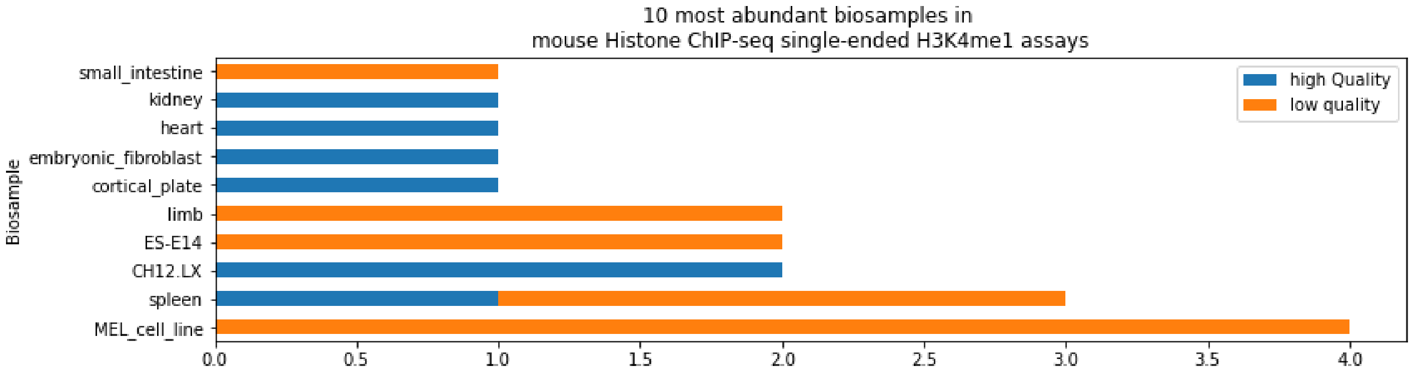

Metrics on training set:
- Accuracy: 1.0

High-quality files:
- Precision: 1.0
- Recall: 1.0
- F1-score: 1.0

Low-quality files
- Precision: 1.0
- Recall: 1.0
- F1-score: 1.0

Legend:
Nodes show data state and decision criteria to apply
Samples by class: [high-quality files, low-quality files]

Group B

mouse single-ended HistoneChIP-seq for H3K4me3

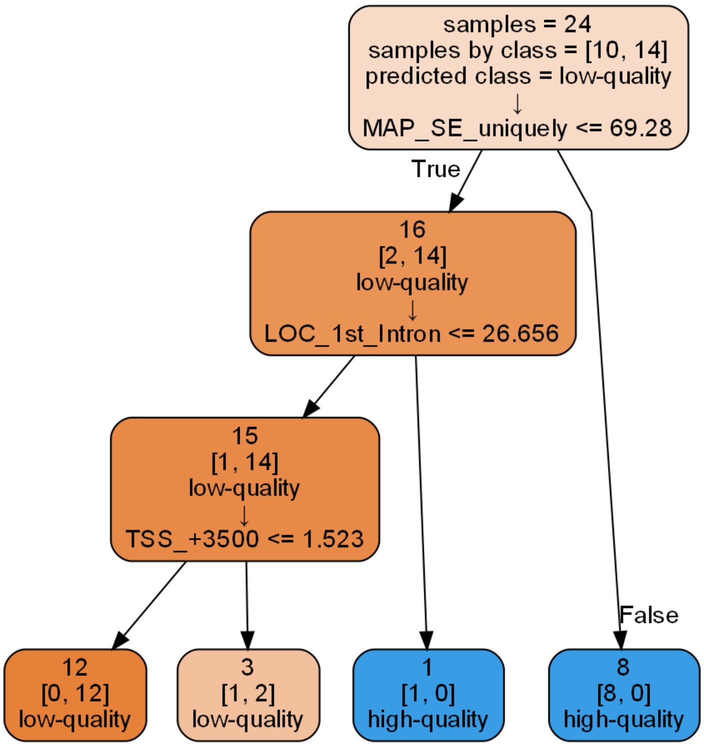

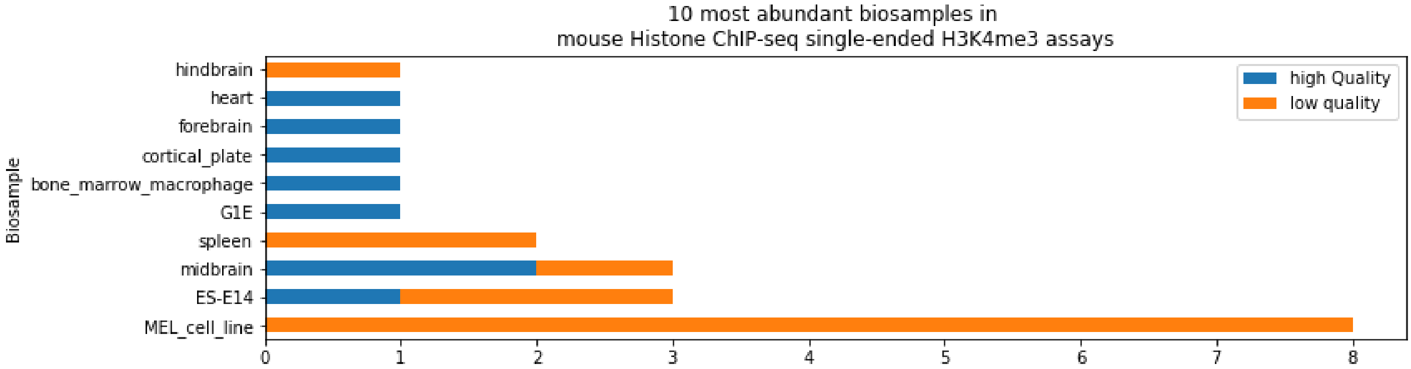

Metrics on training set:

- Accuracy: 0.96

High-quality files:

- Precision: 1.0
- Recall: 0.9
- F1-score: 0.95

Low-quality files

- Precision: 0.93
- Recall: 1.0
- F1-score: 0.97

Legend:

Nodes show data state and decision criteria to apply

Samples by class: [high-quality files, low-quality files]

Group B

mouse single-ended HistoneChIP-seq for H3K9me3

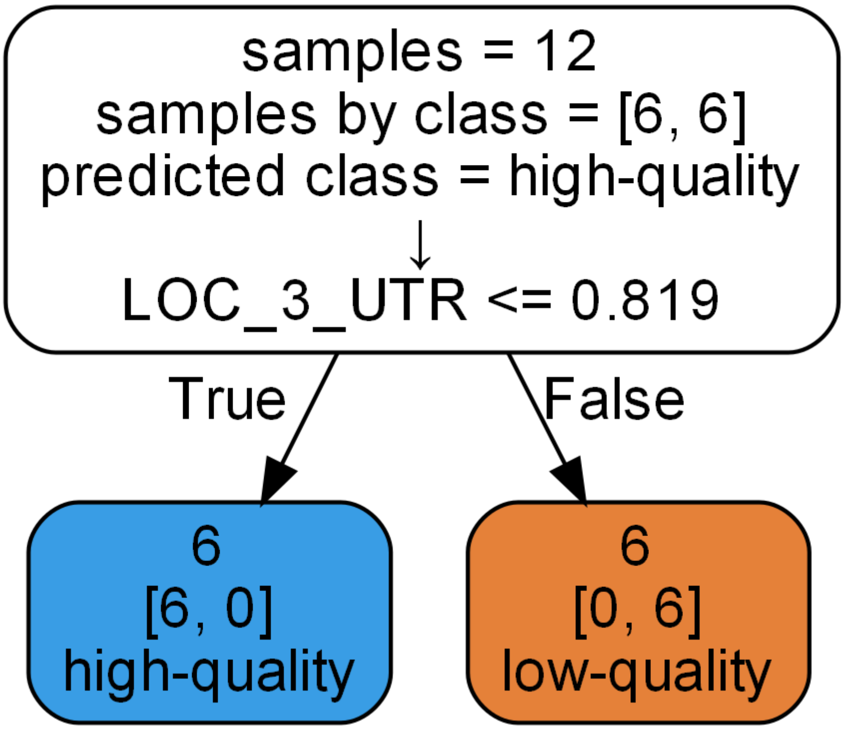

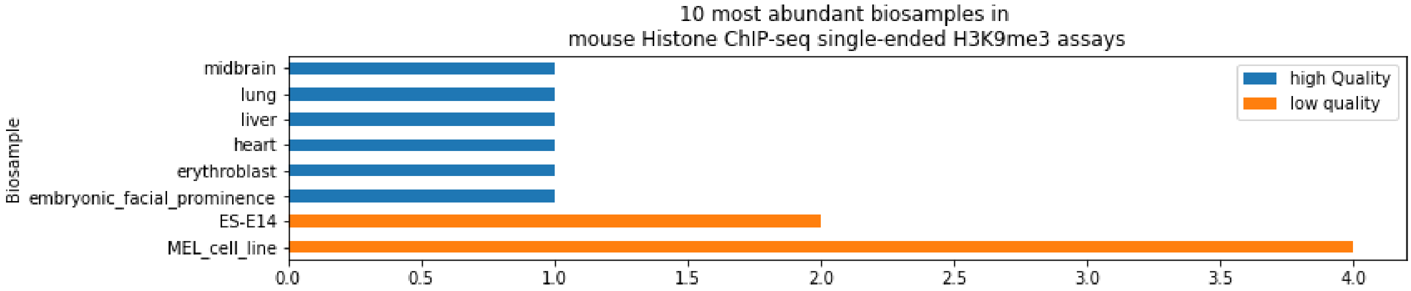

Metrics on training set:

- Accuracy: 1.0

High-quality files:

- Precision: 1.0
- Recall: 1.0
- F1-score: 1.0

Low-quality files

- Precision: 1.0
- Recall: 1.0
- F1-score: 1.0

Legend:

Nodes show data state and decision criteria to apply

Samples by class: [high-quality files, low-quality files]

Group B

mouse single-ended TFChIP-seq for CTCF

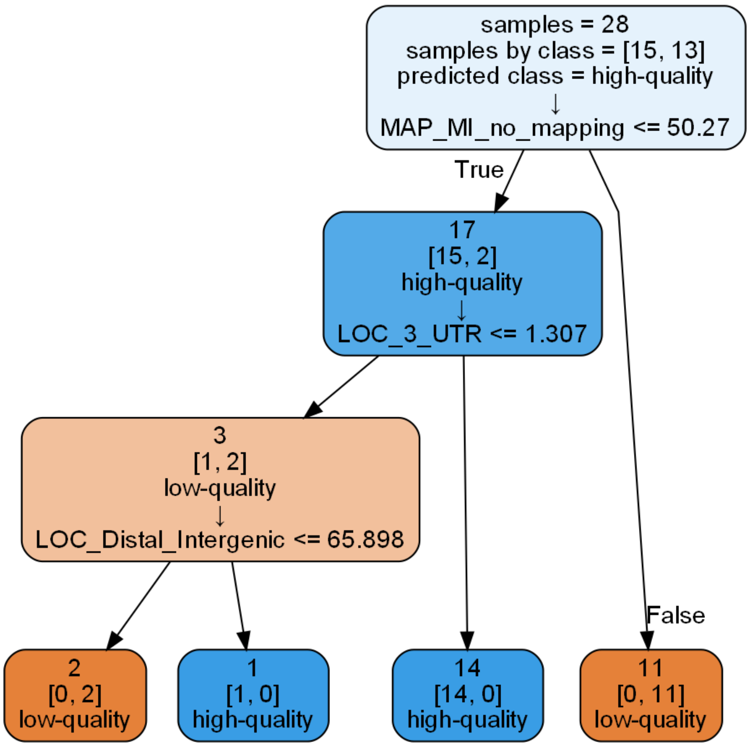

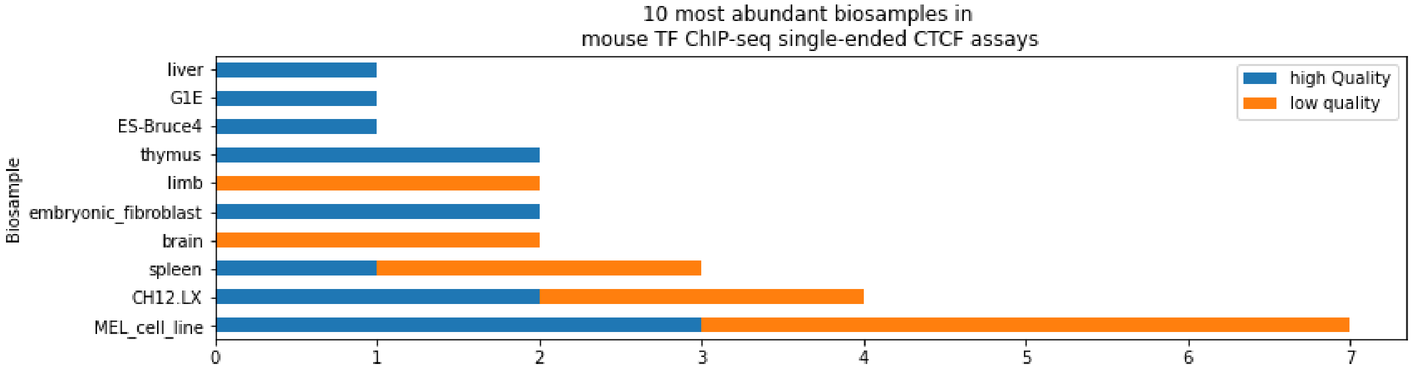

Metrics on training set:

- Accuracy: 1.0

High-quality files:

- Precision: 1.0
- Recall: 1.0
- F1-score: 1.0

Low-quality files

- Precision: 1.0
- Recall: 1.0
- F1-score: 1.0

Legend:

Nodes show data state and decision criteria to apply

Samples by class: [high-quality files, low-quality files]

Group B

mouse single-ended TFChIP-seq for POLR2A

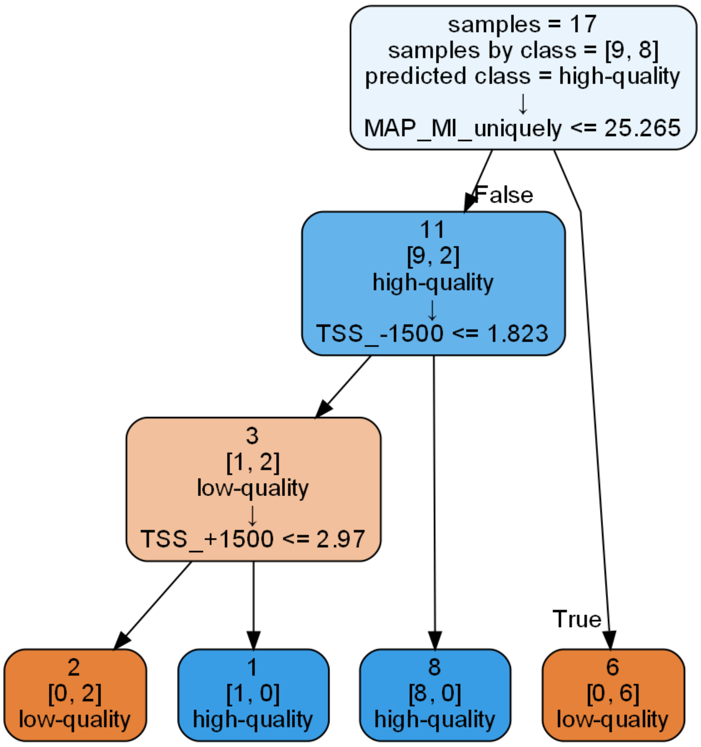

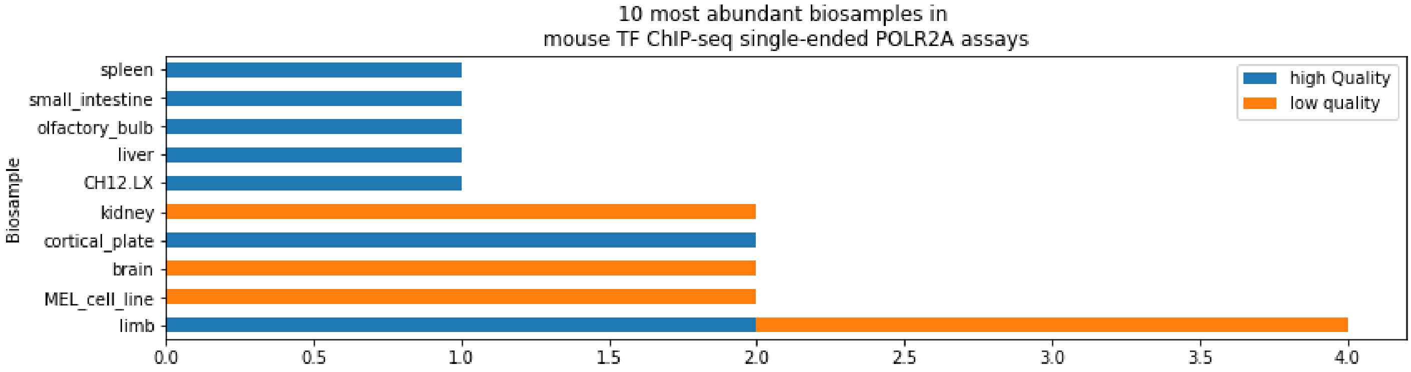

Metrics on training set:
- Accuracy: 1.0

High-quality files:
- Precision: 1.0
- Recall: 1.0
- F1-score: 1.0

Low-quality files
- Precision: 1.0
- Recall: 1.0
- F1-score: 1.0

Legend:
Nodes show data state and decision criteria to apply
Samples by class: [high-quality files, low-quality files]

Group C

human single-ended ControlChIP-seq for rabbit-IgG-control (ENCAB000AOJ)

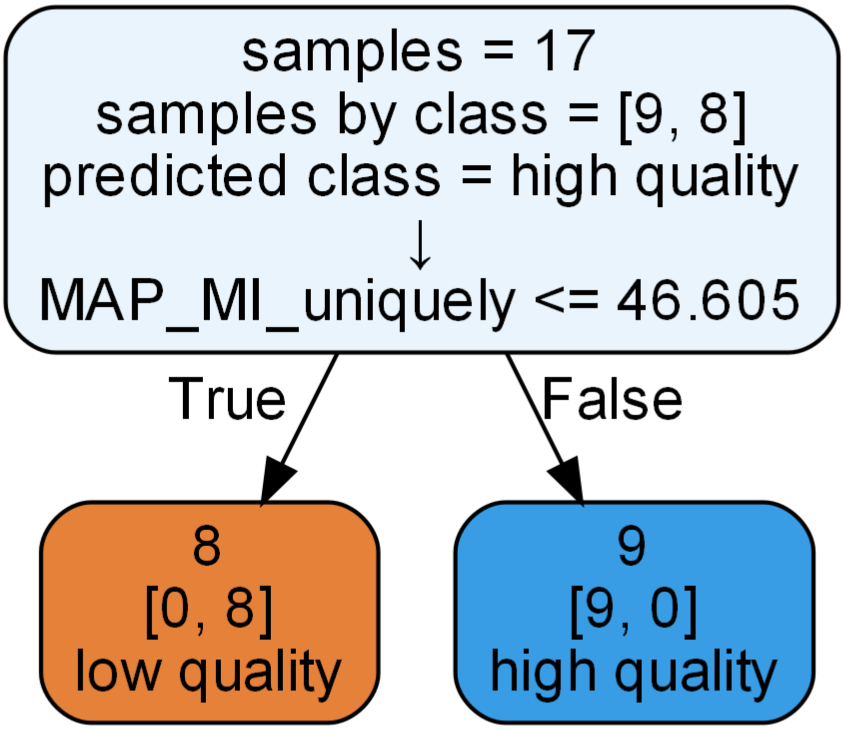

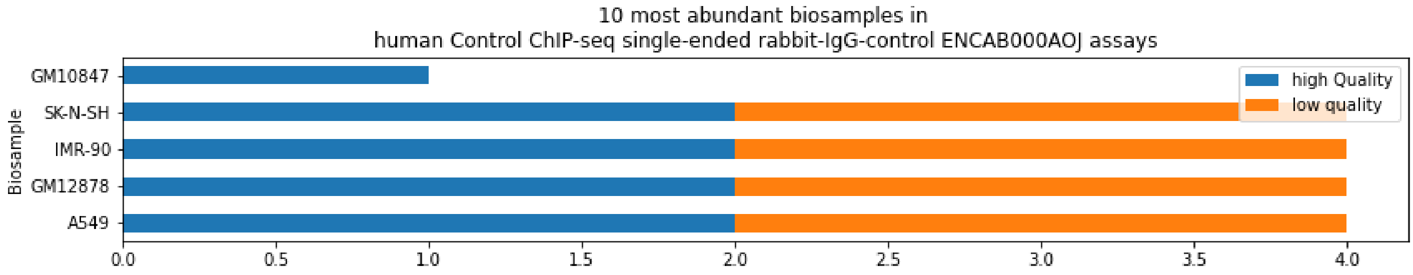

Metrics on training set:

- Accuracy:	1.0
High-quality files:	
- Precision:	1.0
- Recall:	1.0
- F1-score:	1.0
Low-quality files	
- Precision:	1.0
- Recall:	1.0
- F1-score:	1.0

Legend:
Nodes show data state and decision criteria to apply
Samples by class: [high-quality files, low-quality files]

Group C

human single-ended HistoneChIP-seq for H3K27ac (ENCAB000AQN)

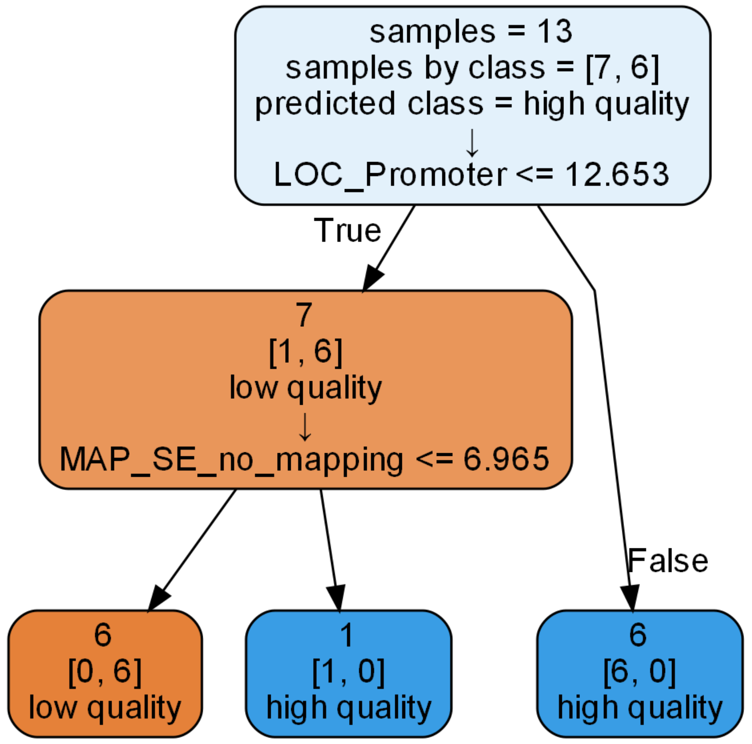

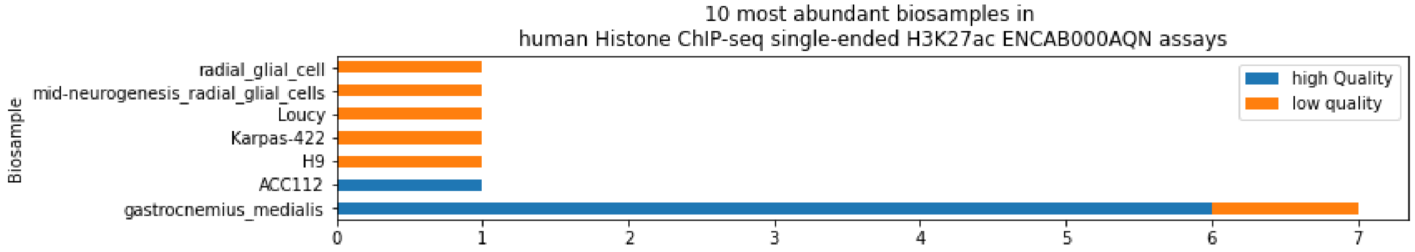

Metrics on training set:

- Accuracy: 1.0

High-quality files:

- Precision: 1.0
- Recall: 1.0
- F1-score: 1.0

Low-quality files

- Precision: 1.0
- Recall: 1.0
- F1-score: 1.0

Legend:

Nodes show data state and decision criteria to apply

Samples by class: [high-quality files, low-quality files]

Group C

human single-ended HistoneChIP-seq for H3K27me3 (ENCAB000ANB)

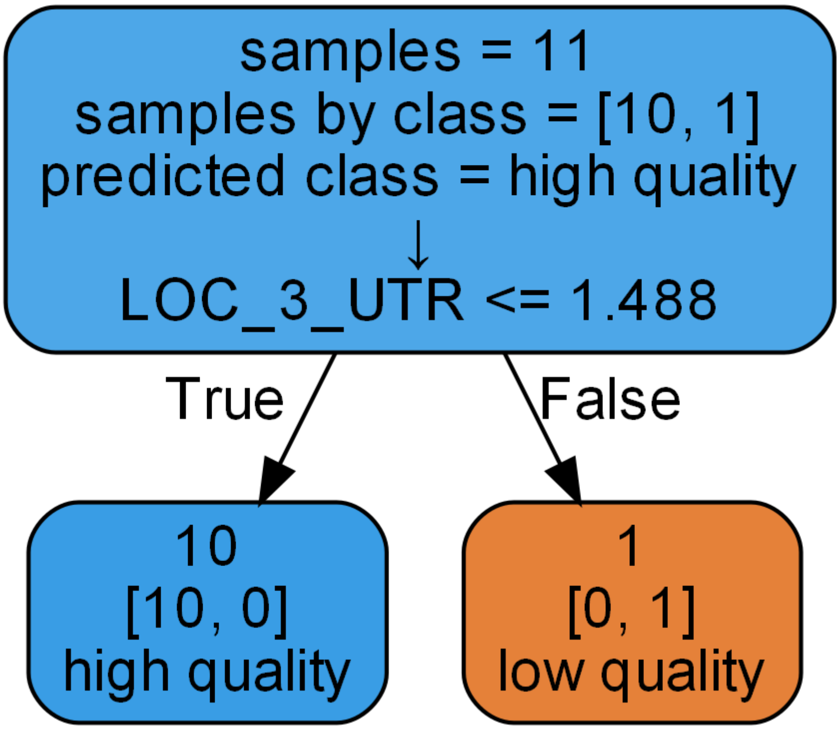

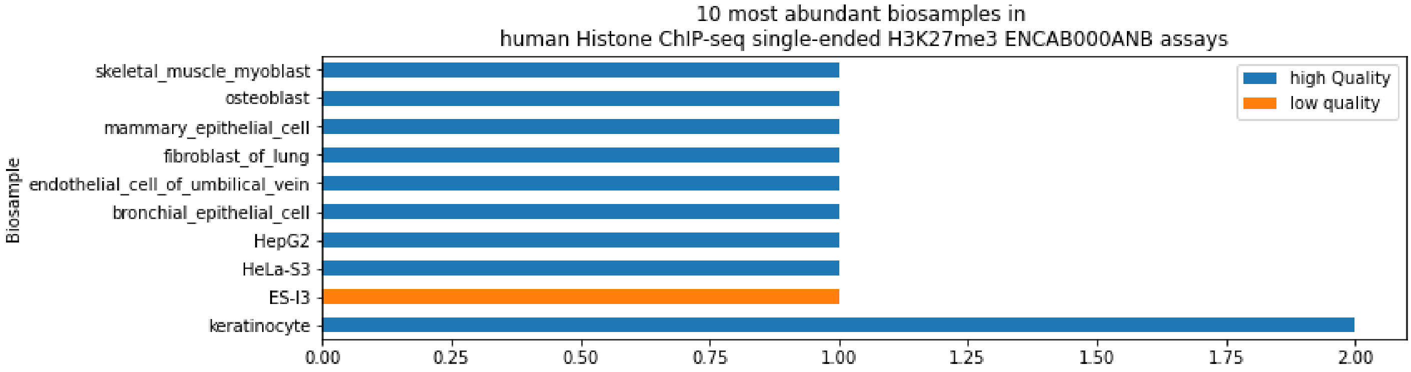

Metrics on training set:

- Accuracy:	1.0
High-quality files:	
- Precision:	1.0
- Recall:	1.0
- F1-score:	1.0
Low-quality files	
- Precision:	1.0
- Recall:	1.0
- F1-score:	1.0

Legend:
Nodes show data state and decision criteria to apply
Samples by class: [high-quality files, low-quality files]

Group C

human single-ended HistoneChIP-seq for H3K36me3 (ENCAB000ADU)

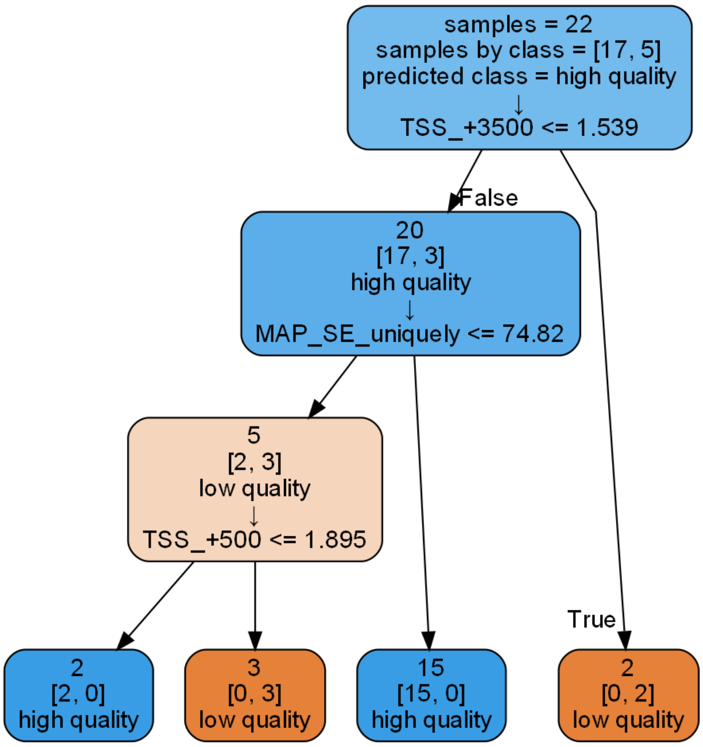

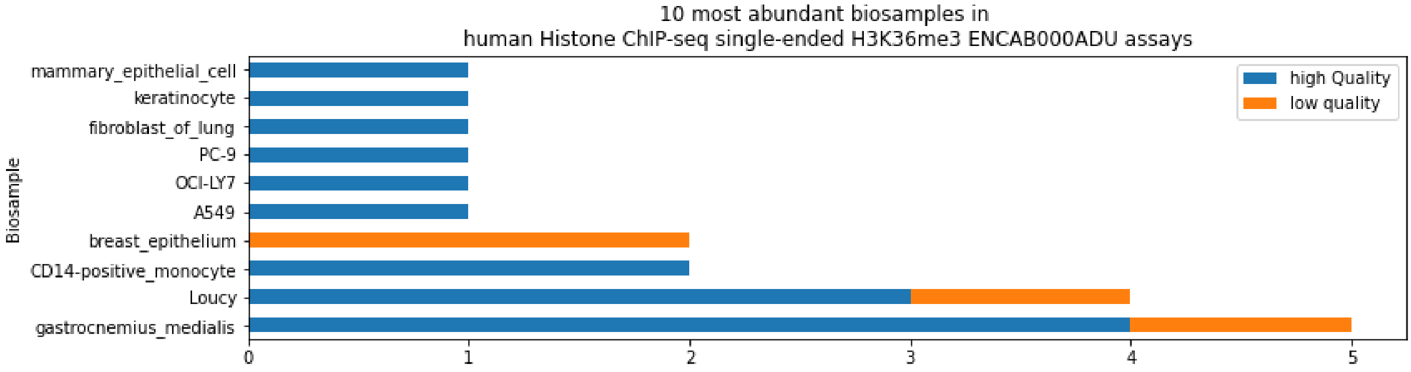

Metrics on training set:

- Accuracy: 1.0

High-quality files:

- Precision: 1.0
- Recall: 1.0
- F1-score: 1.0

Low-quality files

- Precision: 1.0
- Recall: 1.0
- F1-score: 1.0

Legend:

Nodes show data state and decision criteria to apply

Samples by class: [high-quality files, low-quality files]

Group C
human single-ended HistoneChIP-seq for H3K4me1 (ENCAB000ADW)

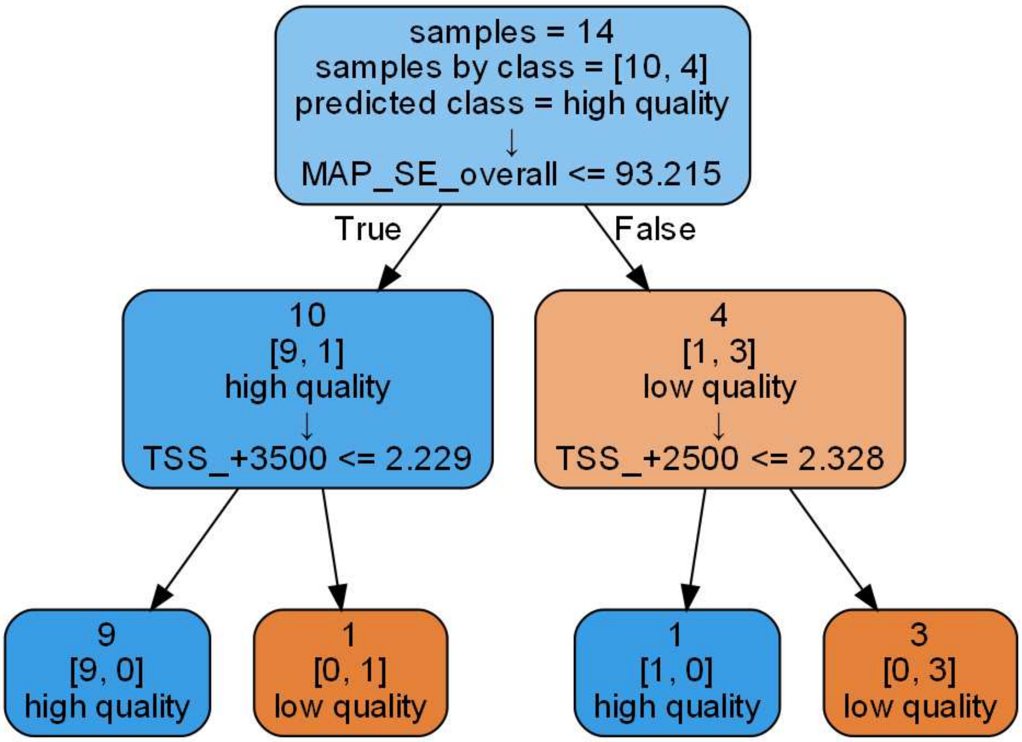

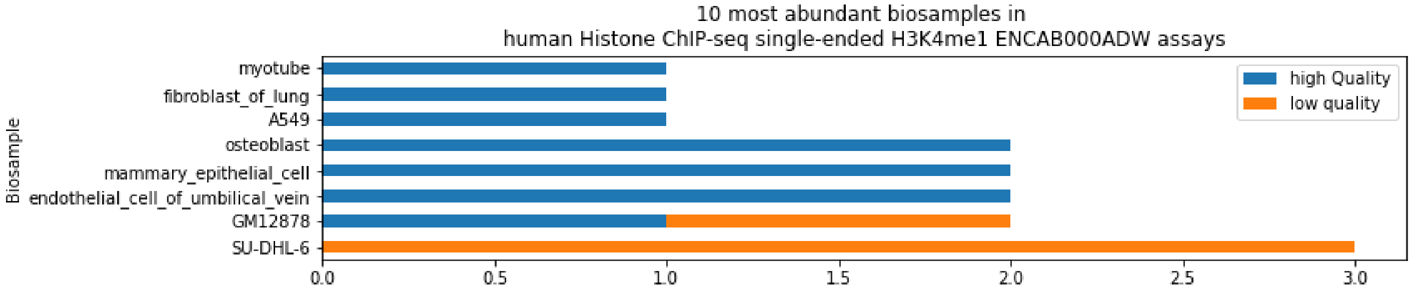

Metrics on training set:
- Accuracy: 1.0

High-quality files:
- Precision: 1.0
- Recall: 1.0
- F1-score: 1.0

Low-quality files
- Precision: 1.0
- Recall: 1.0
- F1-score: 1.0

Legend:
Nodes show data state and decision criteria to apply
Samples by class: [high-quality files, low-quality files]

Group C

human single-ended TFChIP-seq for BACH1 (ENCAB000AEA)

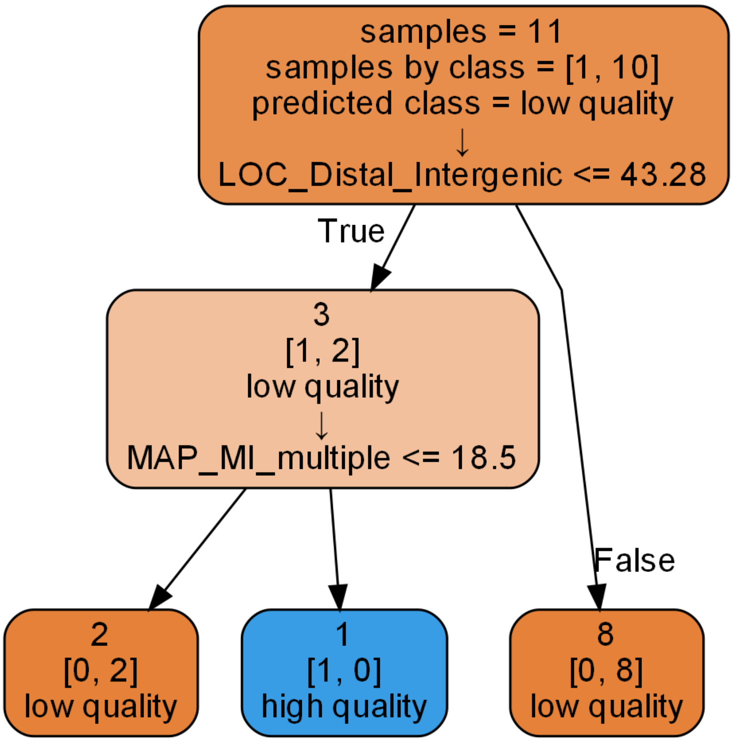

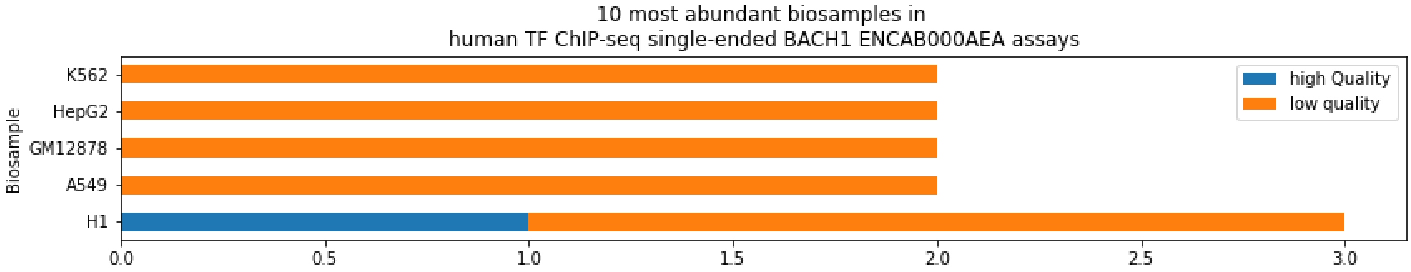

Metrics on training set:
- Accuracy: 1.0

High-quality files:
- Precision: 1.0
- Recall: 1.0
- F1-score: 1.0

Low-quality files
- Precision: 1.0
- Recall: 1.0
- F1-score: 1.0

Legend:
Nodes show data state and decision criteria to apply
Samples by class: [high-quality files, low-quality files]

Group C

human single-ended TFChIP-seq for FOS (ENCAB000AEQ)

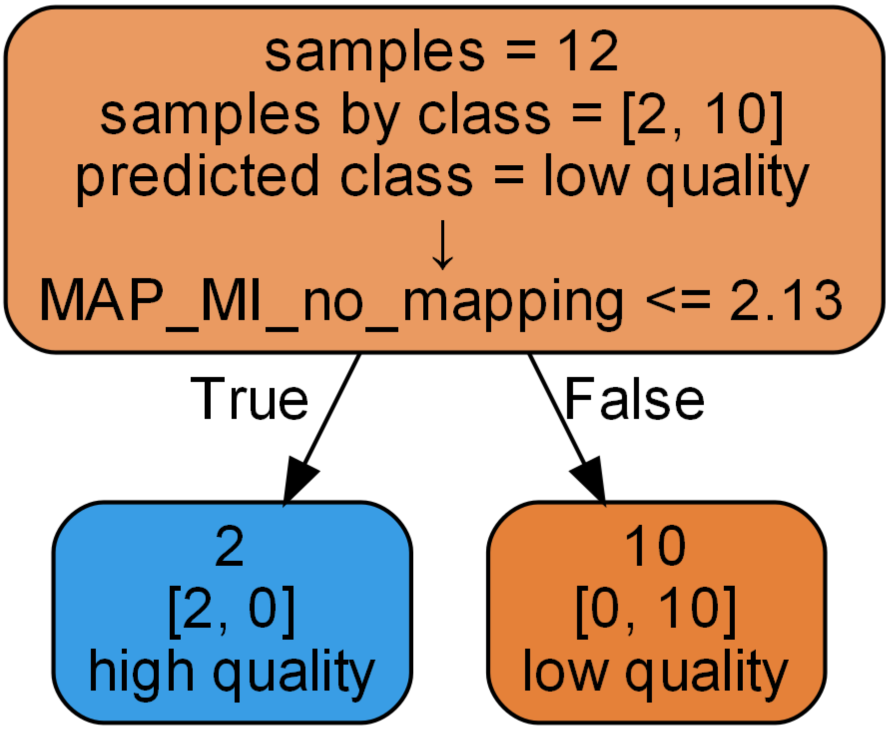

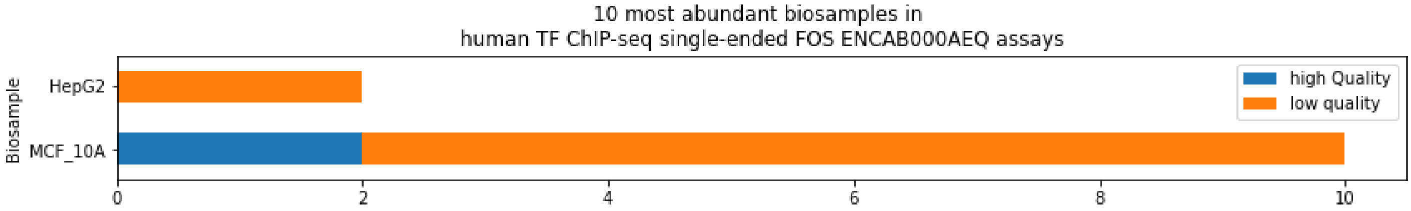

Metrics on training set:

- Accuracy: 1.0

High-quality files:

- Precision: 1.0
- Recall: 1.0
- F1-score: 1.0

Low-quality files

- Precision: 1.0
- Recall: 1.0
- F1-score: 1.0

Legend:

Nodes show data state and decision criteria to apply

Samples by class: [high-quality files, low-quality files]

Group C

human single-ended TFChIP-seq for JUND (ENCAB000AID)

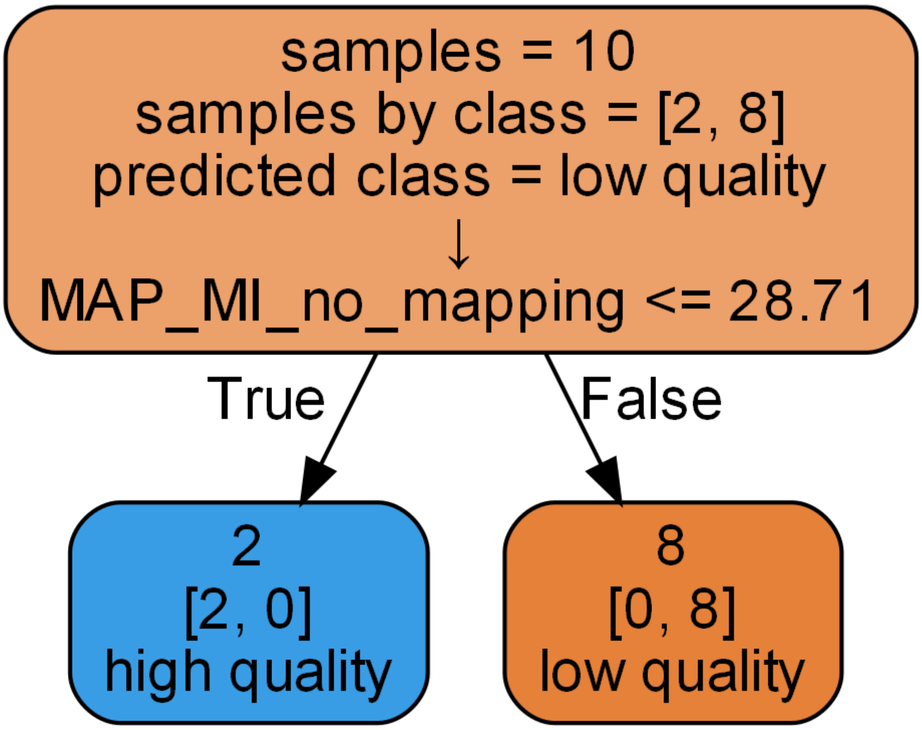

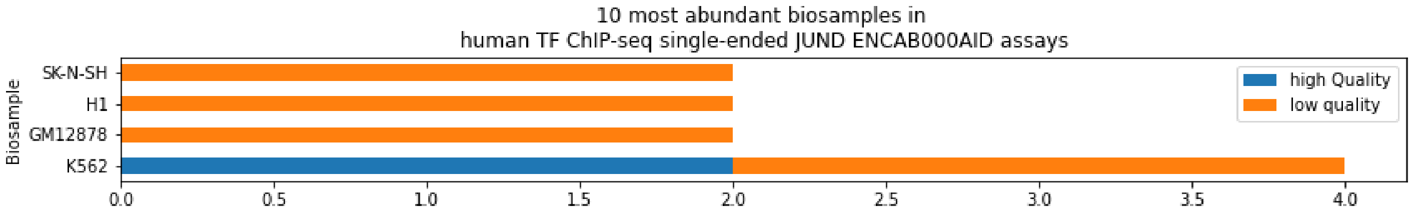

Metrics on training set:
- Accuracy: 1.0

High-quality files:
- Precision: 1.0
- Recall: 1.0
- F1-score: 1.0

Low-quality files
- Precision: 1.0
- Recall: 1.0
- F1-score: 1.0

Legend:

Nodes show data state and decision criteria to apply

Samples by class: [high-quality files, low-quality files]

Group C

human single-ended TFChIP-seq for MAX (ENCAB000AIL)

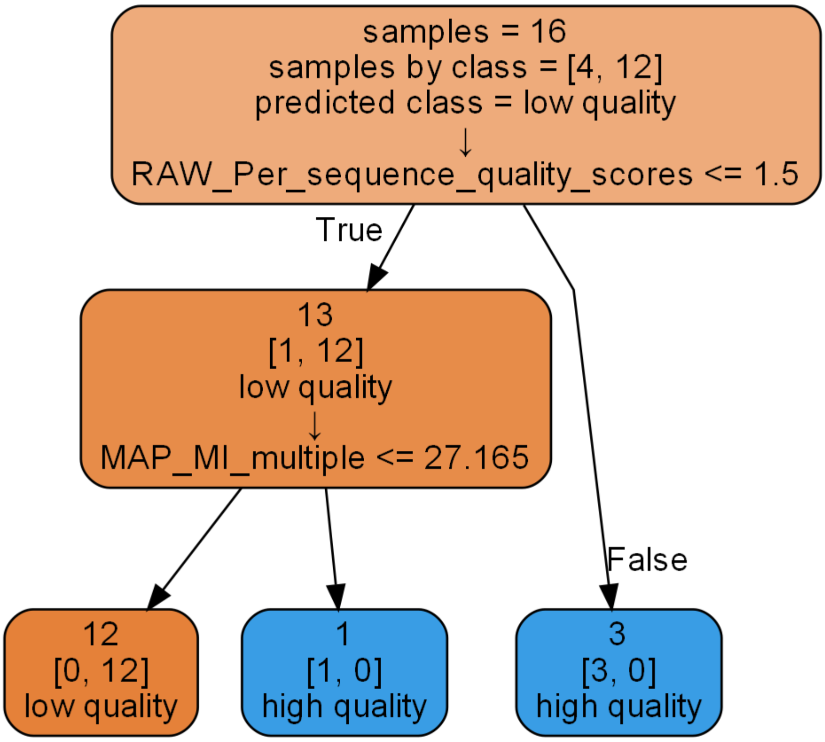

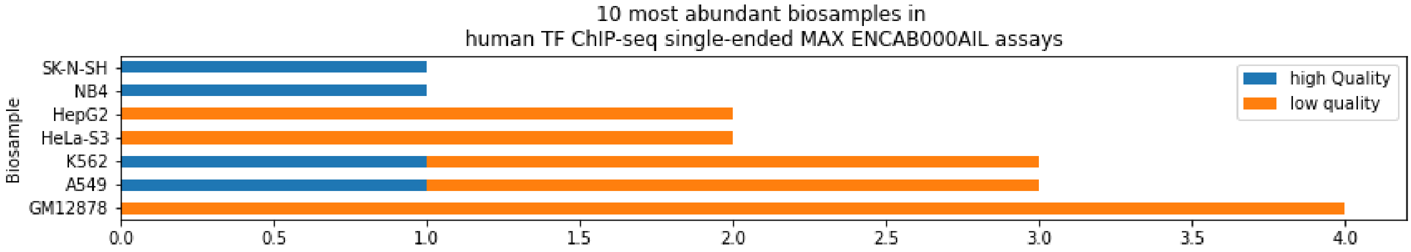

Metrics on training set:

- Accuracy: 1.0

High-quality files:

- Precision: 1.0
- Recall: 1.0
- F1-score: 1.0

Low-quality files

- Precision: 1.0
- Recall: 1.0
- F1-score: 1.0

Legend:

Nodes show data state and decision criteria to apply

Samples by class: [high-quality files, low-quality files]

Group C

human single-ended TFChIP-seq for MAZ (ENCAB000AIM)

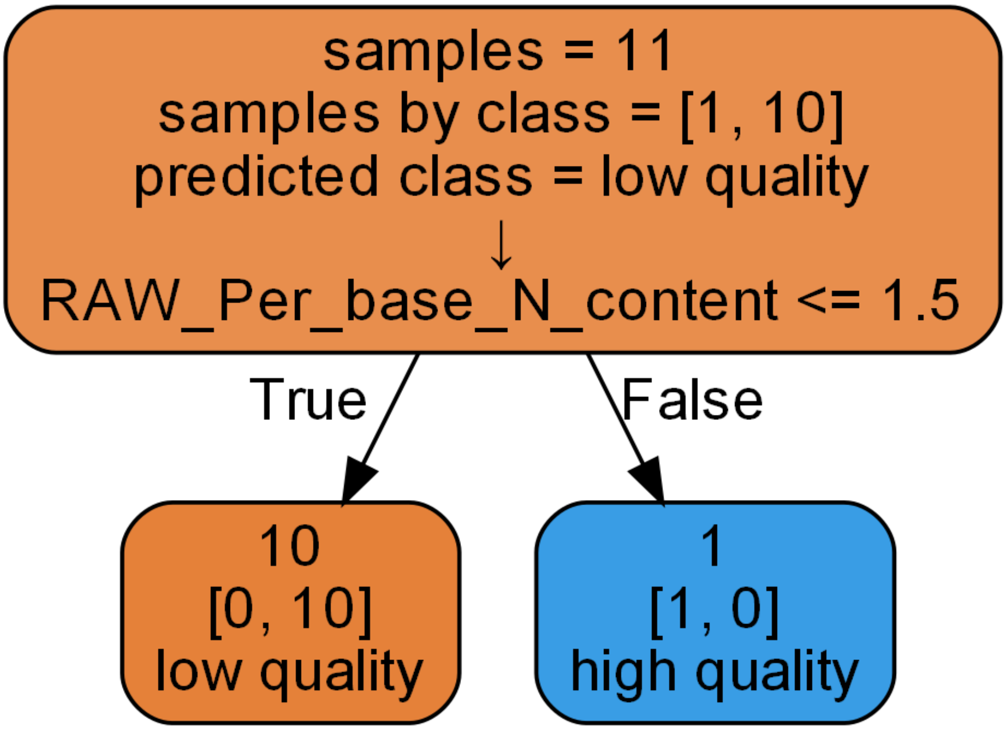

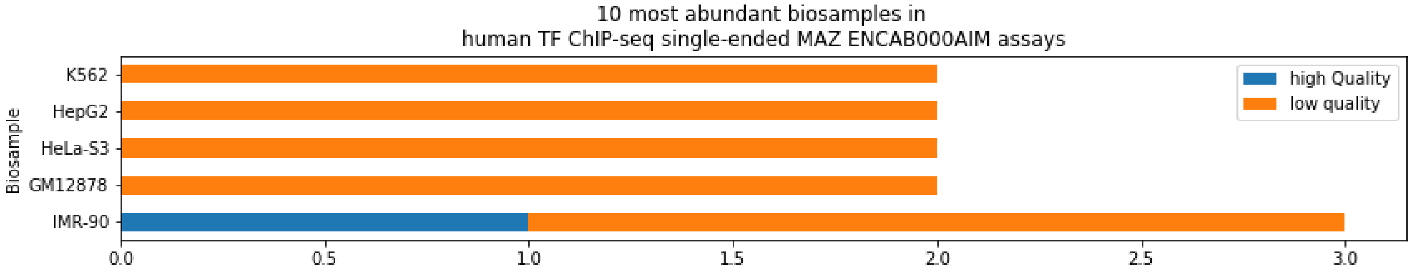

Metrics on training set:	
- Accuracy:	1.0
High-quality files:	
- Precision:	1.0
- Recall:	1.0
- F1-score:	1.0
Low-quality files	
- Precision:	1.0
- Recall:	1.0
- F1-score:	1.0

Legend:
Nodes show data state and decision criteria to apply
Samples by class: [high-quality files, low-quality files]

Group C

human single-ended TFChIP-seq for MXI1 (ENCAB000AIT)

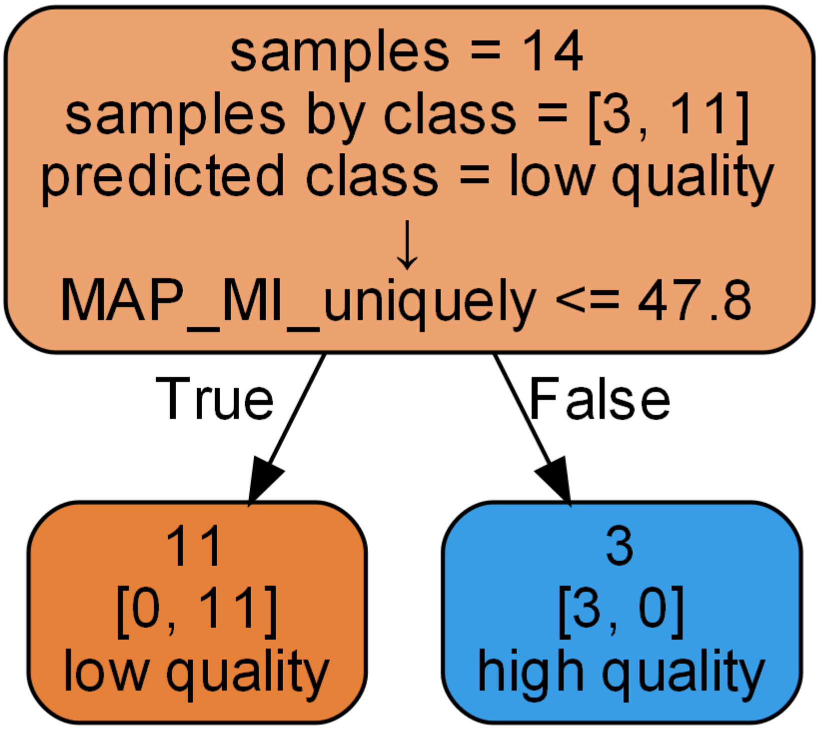

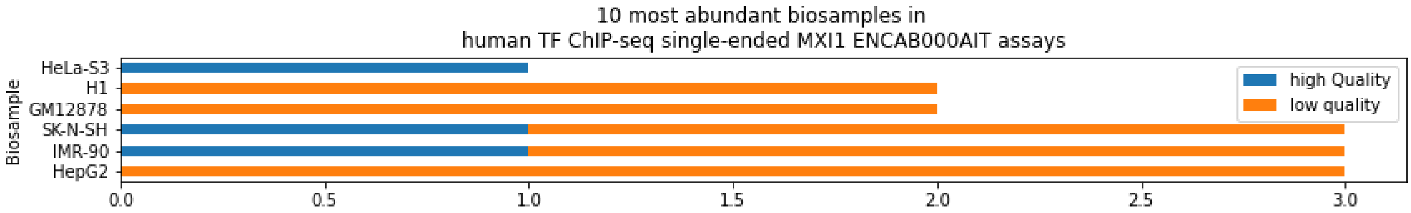

Metrics on training set:
- Accuracy: 1.0

High-quality files:
- Precision: 1.0
- Recall: 1.0
- F1-score: 1.0

Low-quality files
- Precision: 1.0
- Recall: 1.0
- F1-score: 1.0

Legend:
Nodes show data state and decision criteria to apply
Samples by class: [high-quality files, low-quality files]

Group C

human single-ended TFChIP-seq for MYC (ENCAB000AET)

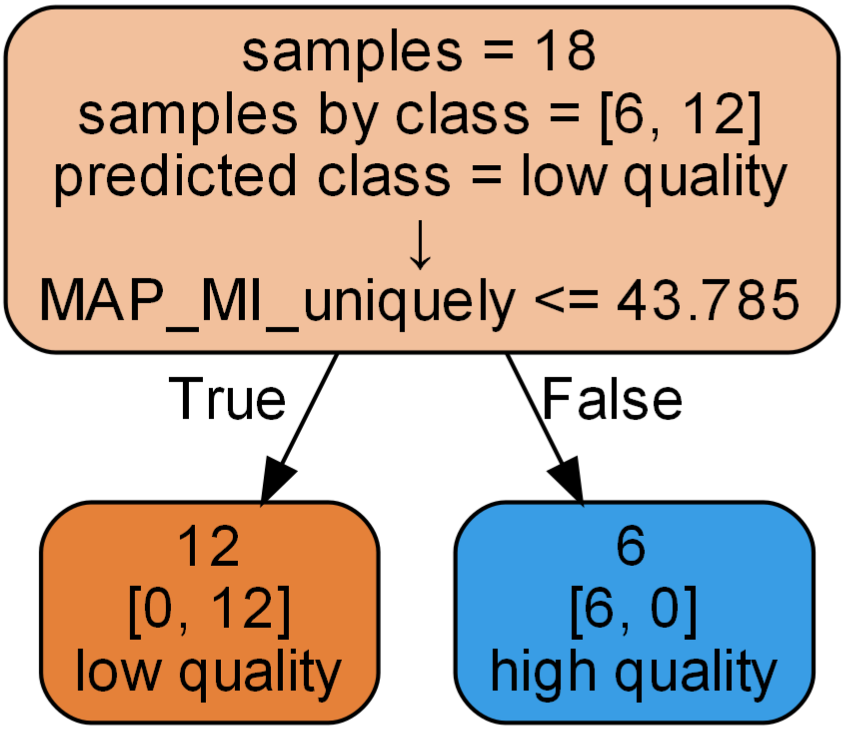

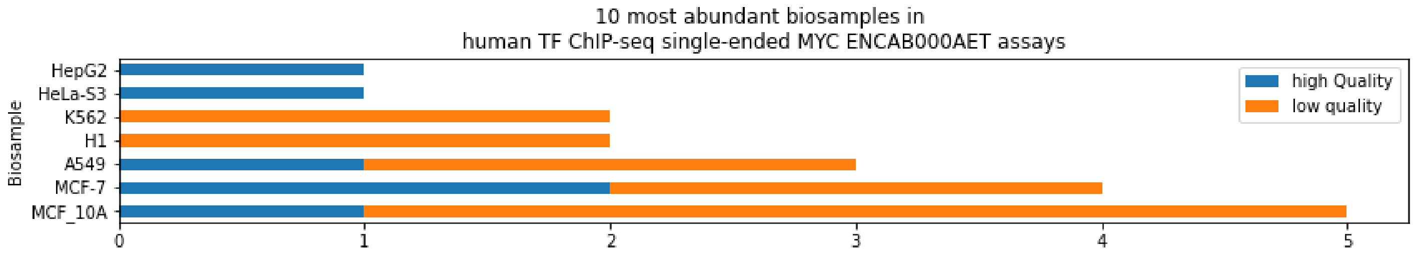

Metrics on training set:

- Accuracy: 1.0

High-quality files:

- Precision: 1.0
- Recall: 1.0
- F1-score: 1.0

Low-quality files

- Precision: 1.0
- Recall: 1.0
- F1-score: 1.0

Legend:

Nodes show data state and decision criteria to apply

Samples by class: [high-quality files, low-quality files]

Group C

human single-ended TFChIP-seq for POLR2A (ENCAB000AOC)

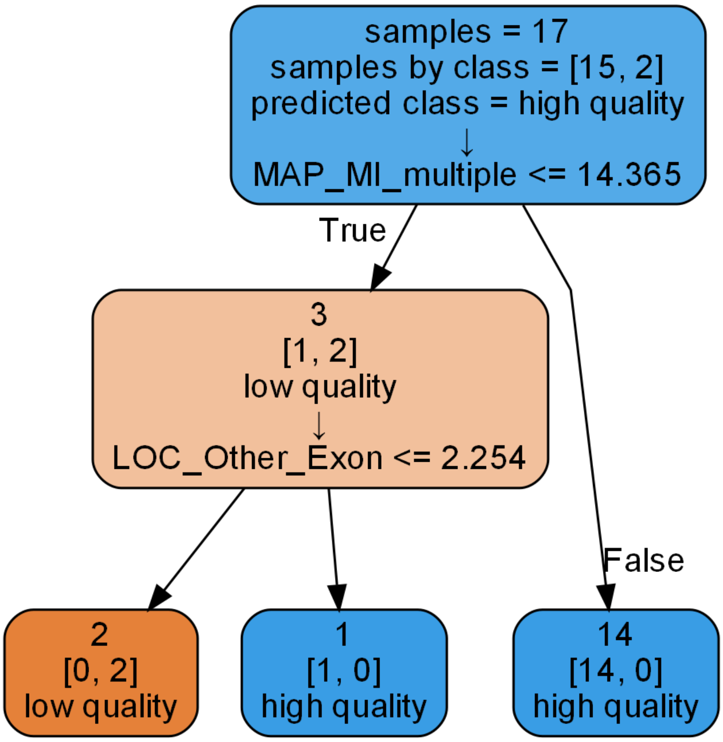

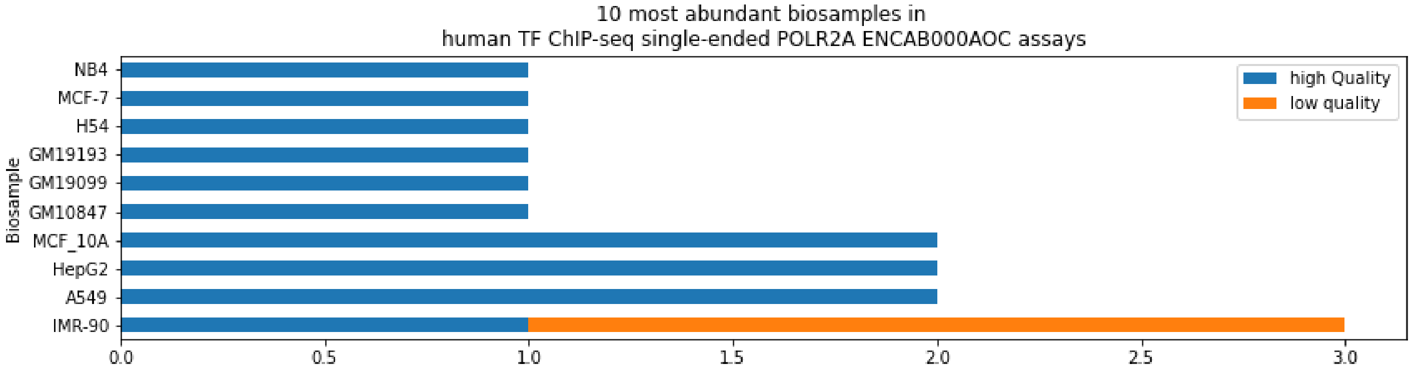

Metrics on training set:
- Accuracy: 1.0

High-quality files:
- Precision: 1.0
- Recall: 1.0
- F1-score: 1.0

Low-quality files
- Precision: 1.0
- Recall: 1.0
- F1-score: 1.0

Legend:

Nodes show data state and decision criteria to apply

Samples by class: [high-quality files, low-quality files]

Group C

human single-ended TFChIP-seq for RELA (ENCAB000AJG)

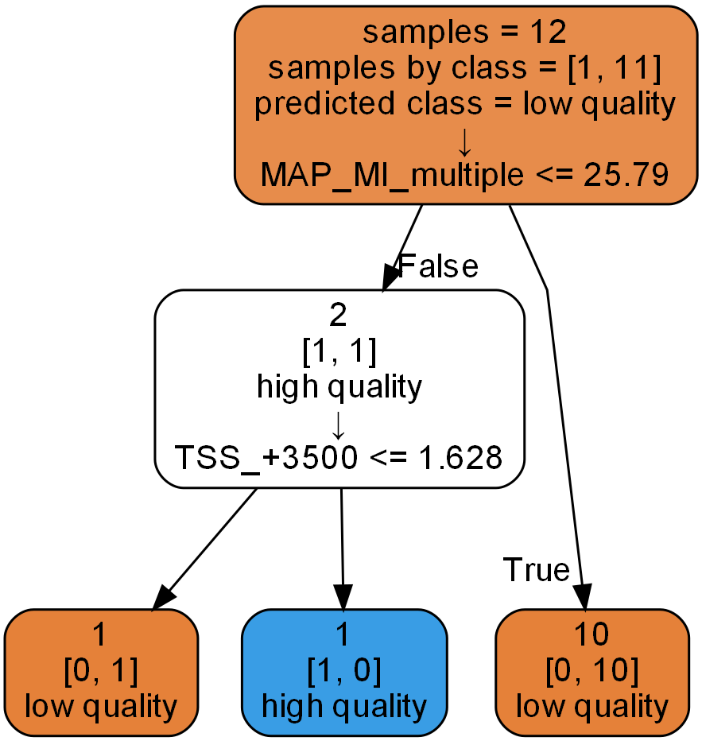

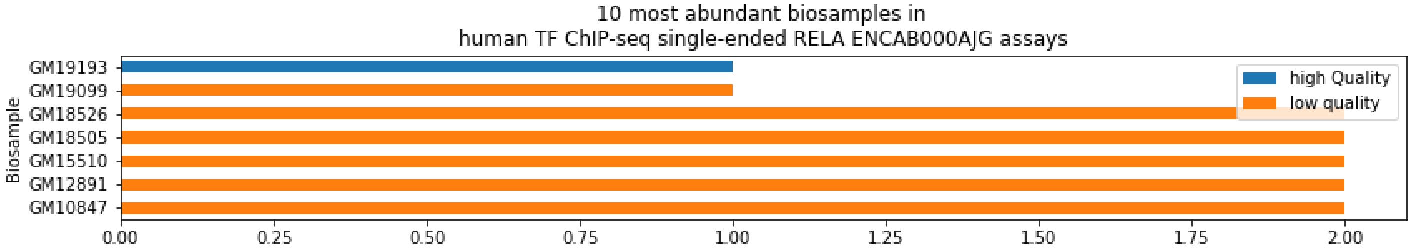

Metrics on training set:
- Accuracy: 1.0

High-quality files:
- Precision: 1.0
- Recall: 1.0
- F1-score: 1.0

Low-quality files
- Precision: 1.0
- Recall: 1.0
- F1-score: 1.0

Legend:

Nodes show data state and decision criteria to apply

Samples by class: [high-quality files, low-quality files]

Group C

human single-ended TFChIP-seq for STAT3 (ENCAB000ALH)

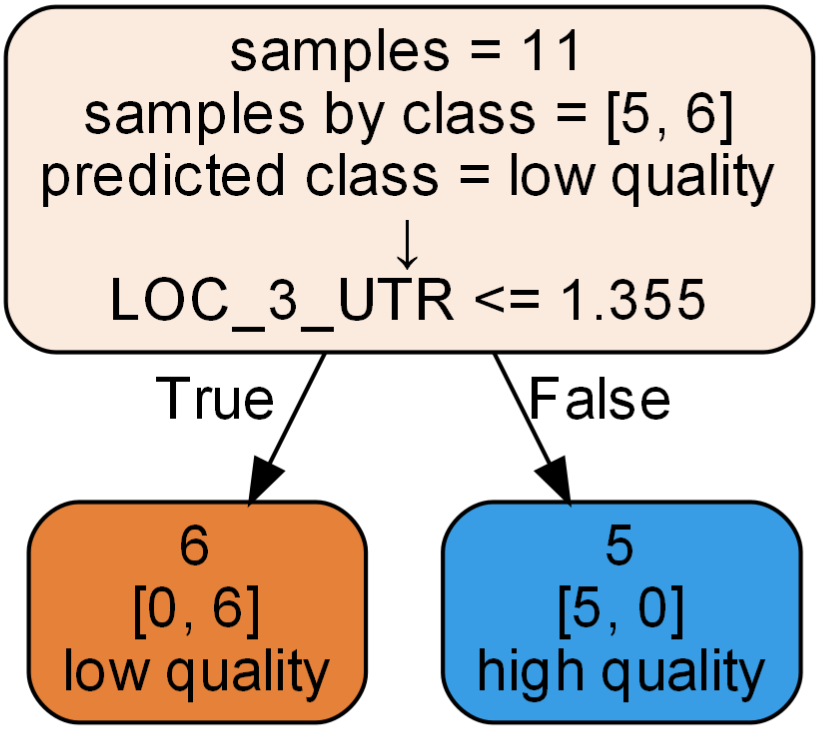

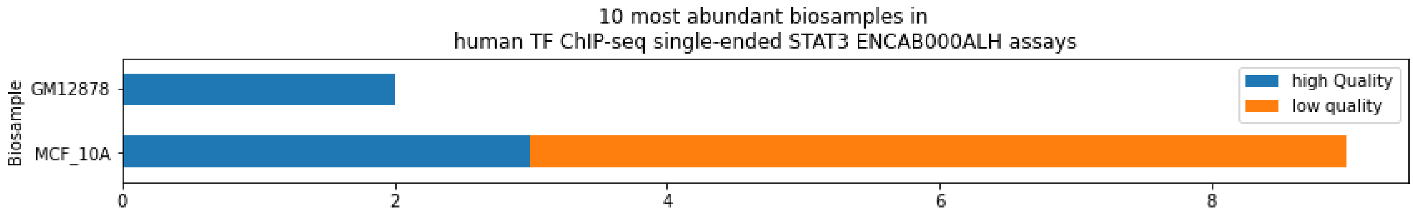

Metrics on training set:

- Accuracy:	1.0
High-quality files:	
- Precision:	1.0
- Recall:	1.0
- F1-score:	1.0
Low-quality files	
- Precision:	1.0
- Recall:	1.0
- F1-score:	1.0

Legend:
Nodes show data state and decision criteria to apply
Samples by class: [high-quality files, low-quality files]

Group C

human single-ended TFChIP-seq for TCF7L2 (ENCAB000AOA)

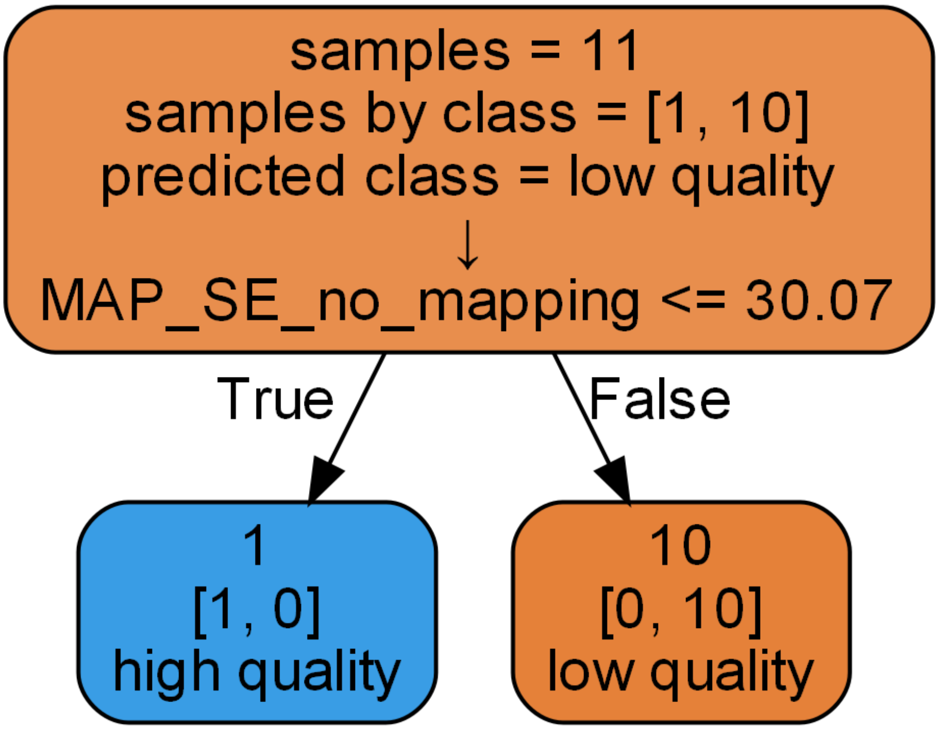

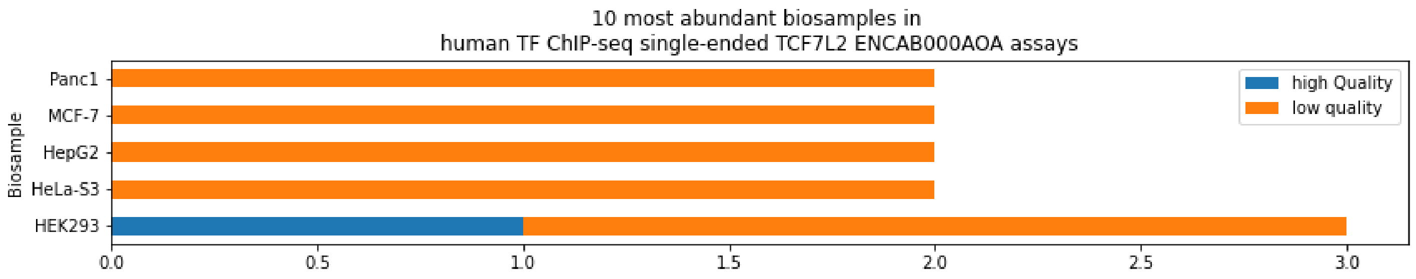

Metrics on training set:
- Accuracy: 1.0

High-quality files:
- Precision: 1.0
- Recall: 1.0
- F1-score: 1.0

Low-quality files
- Precision: 1.0
- Recall: 1.0
- F1-score: 1.0

Legend:
Nodes show data state and decision criteria to apply
Samples by class: [high-quality files, low-quality files]

Group C

mouse single-ended TFChIP-seq for CTCF (ENCAB210NHK)

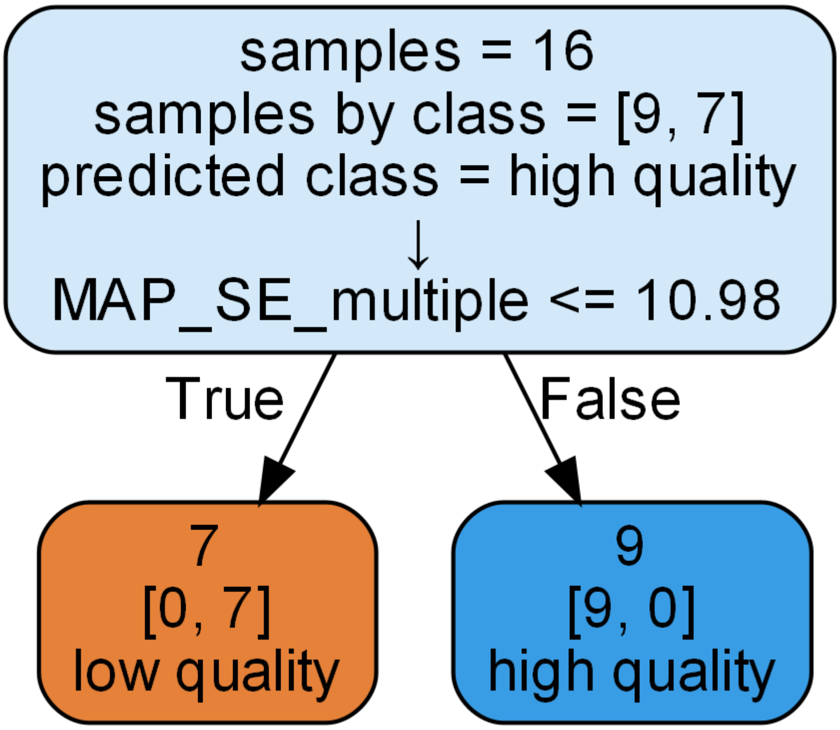

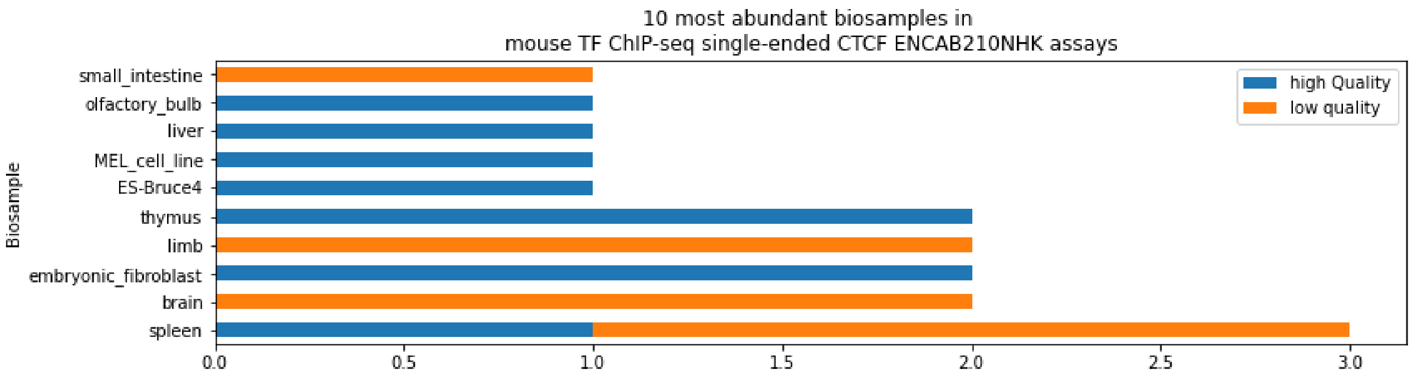

Metrics on training set:

- Accuracy: 1.0

High-quality files:

- Precision: 1.0
- Recall: 1.0
- F1-score: 1.0

Low-quality files

- Precision: 1.0
- Recall: 1.0
- F1-score: 1.0

Legend:

Nodes show data state and decision criteria to apply

Samples by class: [high-quality files, low-quality files]

Group C

mouse single-ended TFChIP-seq for POLR2A (ENCAB281FBQ)

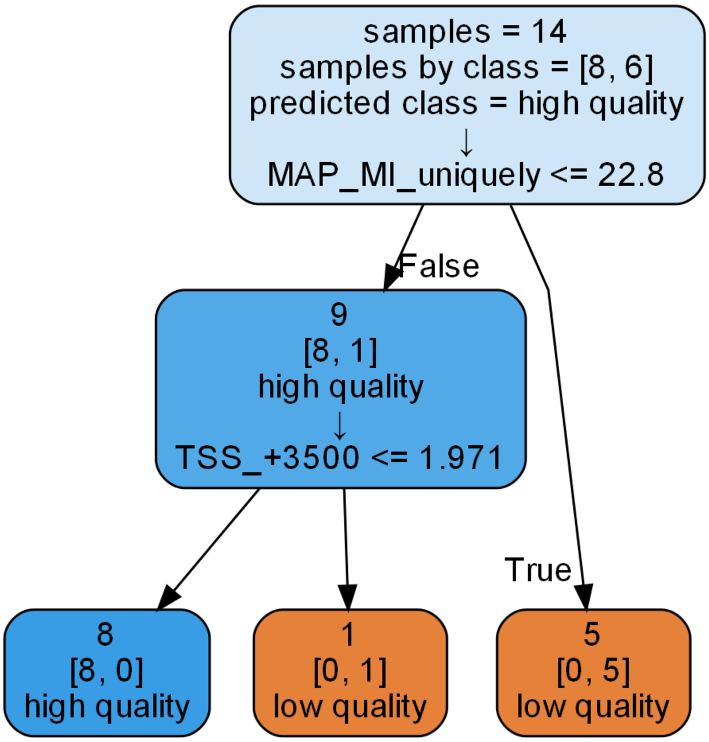

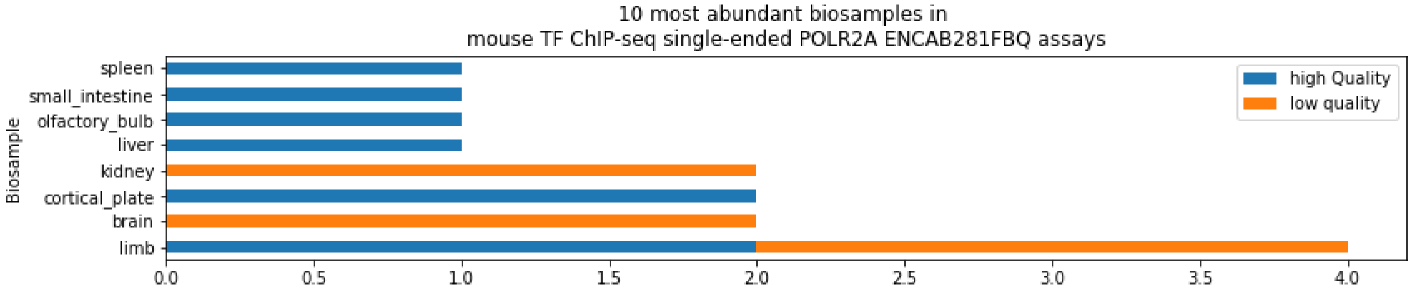

Metrics on training set:

- Accuracy: 1.0

High-quality files:

- Precision: 1.0
- Recall: 1.0
- F1-score: 1.0

Low-quality files

- Precision: 1.0
- Recall: 1.0
- F1-score: 1.0

Legend:

Nodes show data state and decision criteria to apply

Samples by class: [high-quality files, low-quality files]
