# Supplementary material for: Statistical guidelines for quality control of next-generation sequencing techniques
Source: Life Sci Alliance. 2021 Aug 30;4(11):e202101113. doi: 10.26508/lsa.202101113 (PMC8408346; doi:10.26508/lsa.202101113)
Supplement: Supplementary file 2 [file LSA-2021-01113_TableS1.docx]

## Table S1 - Quality Features

RAW was derived from FastQC, MAP are the mapping statistics using Bowtie2, ChipSeeker was used for LOC and ChIPPeakAnno for TSS.

| feature set | Feature | Definition |
| --- | --- | --- |
| RAW | Basic_Statistics | Generates some simple composition statistics, like file name and type but also the overall GC content. Never raises a warning/failure and is only kept for completeness. Values: 0 if failure, 1 if warning, 2 if pass. |
| RAW | Per_base_sequence_quality | Based on the range of phred quality scores across all bases of a read. A warning/failure is returned if any base has a lower quantile below 10/5. Values: 0 if failure, 1 if warning, 2 if pass. |
| RAW | Per_tile_sequence_quality | Averaged base quality over flow tiles of the machine generating the files (only usable with Illumina machines). Values: 0 if failure, 1 if warning, 2 if pass. |
| RAW | Per_sequence_quality_scores | Based on mean phred score for all sequences, raises warning/error when the most frequent mean quality is below 27/20. Values: 0 if failure, 1 if warning, 2 if pass. |
| RAW | Per_base_sequence_content | Based on the content of each base in given sequence, warning/failure when difference between A and T or G and C is greater than 10%/20%. Values: 0 if failure, 1 if warning, 2 if pass. |
| RAW | Per_sequence_GC_content | Based on GC content in given sequence, warning/failure if more than 15%/30% of reads deviate from normal distribution over their mean GC content. Values: 0 if failure, 1 if warning, 2 if pass. |
| RAW | Per_base_N_content | Based on the content of non-callable bases in given position, warning/failure if any position shows more than 5%/10%. Values: 0 if failure, 1 if warning, 2 if pass. |
| RAW | Sequence_Length_Distribution | Based on the distribution of fragment sizes in the file, warning if any of the sequences differ in length, failure when any sequence has a length of zero. Values: 0 if failure, 1 if warning, 2 if pass. |
| RAW | Sequence_Duplication_Levels | Based on the degree of duplication for every sequence in a library, warning/failure issued if non-unique sequences make up more than 20%/50% in total. Values: 0 if failure, 1 if warning, 2 if pass. |
| RAW | Overrepresented_sequences | Based on the count of sequences that are overrepresented in distribution of a file, warning/failure is issued if any sequence is found to represent more than 0.1%/1% of total. Values: 0 if failure, 1 if warning, 2 if pass. |
| RAW | Adapter_Content | Based on specialized Kmer content, for adapter sequences. warning/failure is issued if any sequence is present in more than 5%/10% of all reads. Values: 0 if failure, 1 if warning, 2 if pass. |
| RAW | Kmer_Content | Based on the measure of the number of each 7-mer at each position in the library and uses binomial test to for significant deviations from even coverage at all positions. Warning/failure issued if any k-mer is imbalanced with a binomial p-value < 0.01/10e-5. Values: 0 if failure, 1 if warning, 2 if pass. |
| MAP_SE | no_mapping | Percentage of reads that could not be mapped to reference genome in a single-ended experiment |
| MAP_SE | uniquely | Percentage of reads that are mapped to a unique location in a single-ended experiment |
| MAP_SE | multiple | Percentage of reads that were mapped to multiple locations in a single-ended experiment |
| MAP_SE | Overall | Percentage of reads that could be mapped to reference genome in a single-ended experiment |
| MAP_PE | con_no_mapping | Percentage of read pairs that could not be mapped concordantly to reference genome in a paired-ended experiment |
| MAP_PE | con_uniquely | Percentage of read pairs that are mapped concordantly to a unique location in a paired-ended experiment |
| MAP_PE | con_multiple | Percentage of read pairs that were mapped concordantly to multiple locations in a paired-ended experiment |
| MAP_PE | dis_uniquely | Percentage of read pairs that are mapped discordantly to a unique location in a paired-ended experiment |
| MAP_PE | cod_no_mapping | Percentage of read pairs that could not be mapped concordantly or discordantly to reference genome in a paired-ended experiment |
| MAP_PE | cod_uniquely | Percentage of read pairs that are mapped concordantly or discordantly to a unique location in a paired-ended experiment |
| MAP_PE | cod_multiple | Percentage of read pairs that were mapped concordantly or discordantly to multiple locations in a paired-ended experiment |
| MAP_PE | overall | Percentage of reads that could be mapped to reference genome in a paired-ended experiment |
| MAP_MI | no_mapping | Equals either MAP_SE_no_mapping for single-ended experiments, or MAP_PE_con_no_mapping for paired-ended experiments |
| MAP_MI | uniquely | Equals either MAP_SE_uniquely for single-ended experiments, or MAP_PE_con_uniquely for paired-ended experiments |
| MAP_MI | multiple | Equals either MAP_SE_multiple for single-ended experiments, or MAP_PE_con_multiple for paired-ended experiments |
| MAP_MI | Overall | Equals either MAP_SE_overall for single-ended experiments, or MAP_PE_con_overall for paired-ended experiments |
| LOC | Promoter | Percentage of reads in promoter regions |
| LOC | 5_UTR | Percentage of reads in 5’ UTR regions |
| LOC | 3_UTR | Percentage of reads in 3’ UTR regions |
| LOC | 1st_Exon | Percentage of reads in 1st exon regions |
| LOC | Other_Exon | Percentage of reads in non-first exon regions |
| LOC | 1st_Intron | Percentage of reads in 1st intron regions |
| LOC | Other_Intron | Percentage of reads in non-first intron regions |
| LOC | Downstream | Percentage of reads in downstream gene regions |
| LOC | Distal_Intergenic | Percentage of reads in distal intergenic regions |
| TSS | -4500 | Percentage of reads in -5k, -4k region relative to TSS |
| TSS | -3500 | Percentage of reads in [-4k, -3k] bp region relative to transcription start sites |
| TSS | -2500 | Percentage of reads in [-3k, -2k] bp region relative to transcription start sites |
| TSS | -1500 | Percentage of reads in [-2k, -1k] bp region relative to transcription start sites |
| TSS | -500 | Percentage of reads in [-1k, 0] bp region relative to transcription start sites |
| TSS | 500 | Percentage of reads in [0k, 1k] bp region relative to transcription start sites |
| TSS | 1500 | Percentage of reads in [1k, 2k] bp region relative to transcription start sites |
| TSS | 2500 | Percentage of reads in [2k, 3k] bp region relative to transcription start sites |
| TSS | 3500 | Percentage of reads in [3k, 4k] bp region relative to transcription start sites |
| TSS | 4500 | Percentage of reads in [4k, 5k] bp region relative to transcription start sites |
