# Supplementary material for: Statistical guidelines for quality control of next-generation sequencing techniques
Source: Life Sci Alliance. 2021 Aug 30;4(11):e202101113. doi: 10.26508/lsa.202101113 (PMC8408346; doi:10.26508/lsa.202101113)
Supplement: Supplementary file 3 [file LSA-2021-01113_TableS2.docx]

## Table S2 - Feature TSS_+4500 in group A subsets related to mouse paired-ended DNAse-seq

fdr: false discovery rate (Benjamini Hochberg); n_low and n_high: number of low- and high-quality files, respectively; CI_90_low and CI_90_high: 90% confidence interval of low- and high-quality files, respectively.

| Sample | fdr | n_low | n_high | CI_90_low | CI_90_high |
| --- | --- | --- | --- | --- | --- |
| midbrain | 0.02536 | 8 | 5 | 1.07-1.09 | 1.17-1.42 |
| hindbrain | 0.08578 | 9 | 11 | 1.14-1.22 | 1.17-1.38 |
| limb | 0.1886 | 13 | 7 | 1.21-1.25 | 1.2-1.31 |
