# Supplementary material for: Statistical guidelines for quality control of next-generation sequencing techniques
Source: Life Sci Alliance. 2021 Aug 30;4(11):e202101113. doi: 10.26508/lsa.202101113 (PMC8408346; doi:10.26508/lsa.202101113)
Supplement: Supplementary file 4 [file LSA-2021-01113_TableS3.docx]

## Table S3 - Feature MAP_MI_overall_mapping in group B subsets related to mouse single-ended histone ChIP-seq

fdr: false discovery rate (Benjamini Hochberg); n_low and n_high: number of low- and high-quality files, respectively; CI_90_low and CI_90_high: 90% confidence interval of low- and high-quality files, respectively.

| Protein | fdr | n_low | n_high | CI_90_low | CI_90_high |
| --- | --- | --- | --- | --- | --- |
| H3K4me1 | 0.0175 | 11 | 7 | 32.3-45.54 | 56.67-98.68 |
| H3K9me3 | 0.03684 | 6 | 6 | 34.19-43.52 | 94.81-96.37 |
| H3K27me3 | 0.03908 | 6 | 10 | 33.38-82.9 | 59.71-96.81 |
| H3K4me3 | 0.06827 | 14 | 10 | 28.67-93.41 | 78.25-97.63 |
| H3K36me3 | 0.2662 | 6 | 10 | 34.22-95.17 | 74.01-98.23 |
