# Supplementary material for: Statistical guidelines for quality control of next-generation sequencing techniques
Source: Life Sci Alliance. 2021 Aug 30;4(11):e202101113. doi: 10.26508/lsa.202101113 (PMC8408346; doi:10.26508/lsa.202101113)
Supplement: Supplementary file 5 [file LSA-2021-01113_TableS4.docx]

## Table S4 - Feature MAP_MI_multiple_mapping in group C subsets related to CTCF mouse single-ended TF ChIP-seq

fdr: false discovery rate (Benjamini Hochberg); n_low and n_high: number of low- and high-quality files, respectively; CI_90_low and CI_90_high: 90% confidence interval of low- and high-quality files, respectively.

| Antibody | fdr | n_low | n_high | CI_90_low | CI_90_high |
| --- | --- | --- | --- | --- | --- |
| ENCAB210NHK | 0.01869 | 7 | 9 | 2.39-5.36 | 17.15-28.63 |
| ENCAB000AFQ | 0.6006 | 6 | 3 | 6.92-24.05 | 21.21-22.58 |
