# Supplementary material for: Statistical guidelines for quality control of next-generation sequencing techniques
Source: Life Sci Alliance. 2021 Aug 30;4(11):e202101113. doi: 10.26508/lsa.202101113 (PMC8408346; doi:10.26508/lsa.202101113)

## Table S5 – Classification performance of individual features in group 1 subsets (area under ROC curve).

We analyzed the performance of the quality features in data subsets divided by organism, assay and run type (group 1 subsets). The Table shows the areas under Receiver Operating Characteristics curves (auROCs) of every feature for the respective subsets. Subsets are given as Organism__assay__runType. MAP features perform best overall and especially well in single-ended ChIP-Seq in mice. The RAW features also perform better for this subset than for others, the files seem to be well distributed according to their quality across these features.


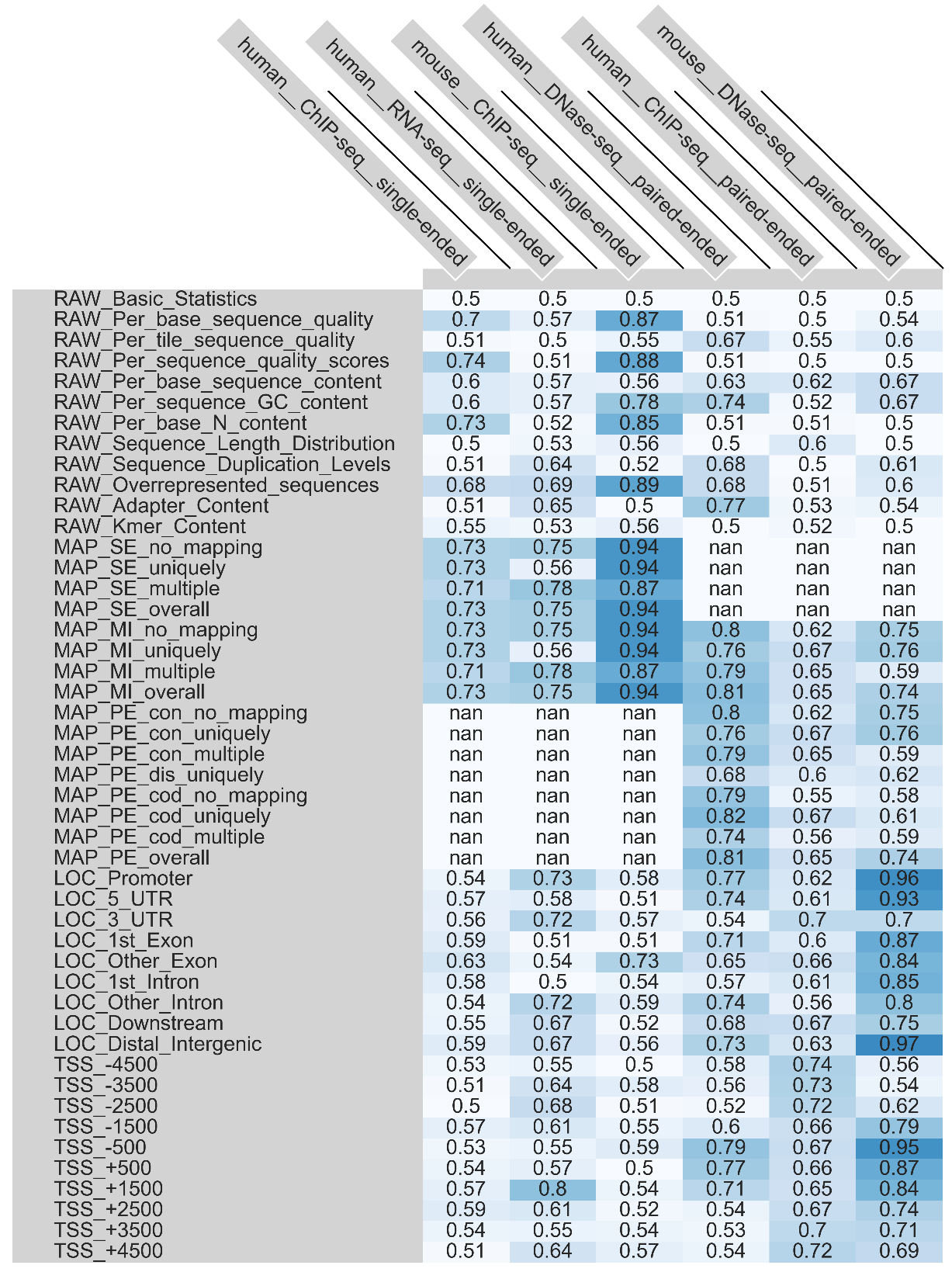

Supplement: Supplementary file 6 [file LSA-2021-01113_TableS5.docx]
