# Supplementary material for: Statistical guidelines for quality control of next-generation sequencing techniques
Source: Life Sci Alliance. 2021 Aug 30;4(11):e202101113. doi: 10.26508/lsa.202101113 (PMC8408346; doi:10.26508/lsa.202101113)

## Table S6 – Classification performance of individual features in group 2 subsets (area under ROC curve).

We analyzed the performance of the quality features in data subsets divided by organism, assay and run type (group 1 subsets). The Table shows the areas under Receiver Operating Characteristics curves (auROCs) of every feature for the respective subsets. Subsets are given as Organism__assayTitle__runType. MAP features perform best overall, especially in the single end assays. LOC and TSS features perform much better in paired end mouse DNAse-Seq and human Histone ChIP-seq, than in the rest of the subsets. The RAW features perform good for single end assays, but not so well for paired end assays.


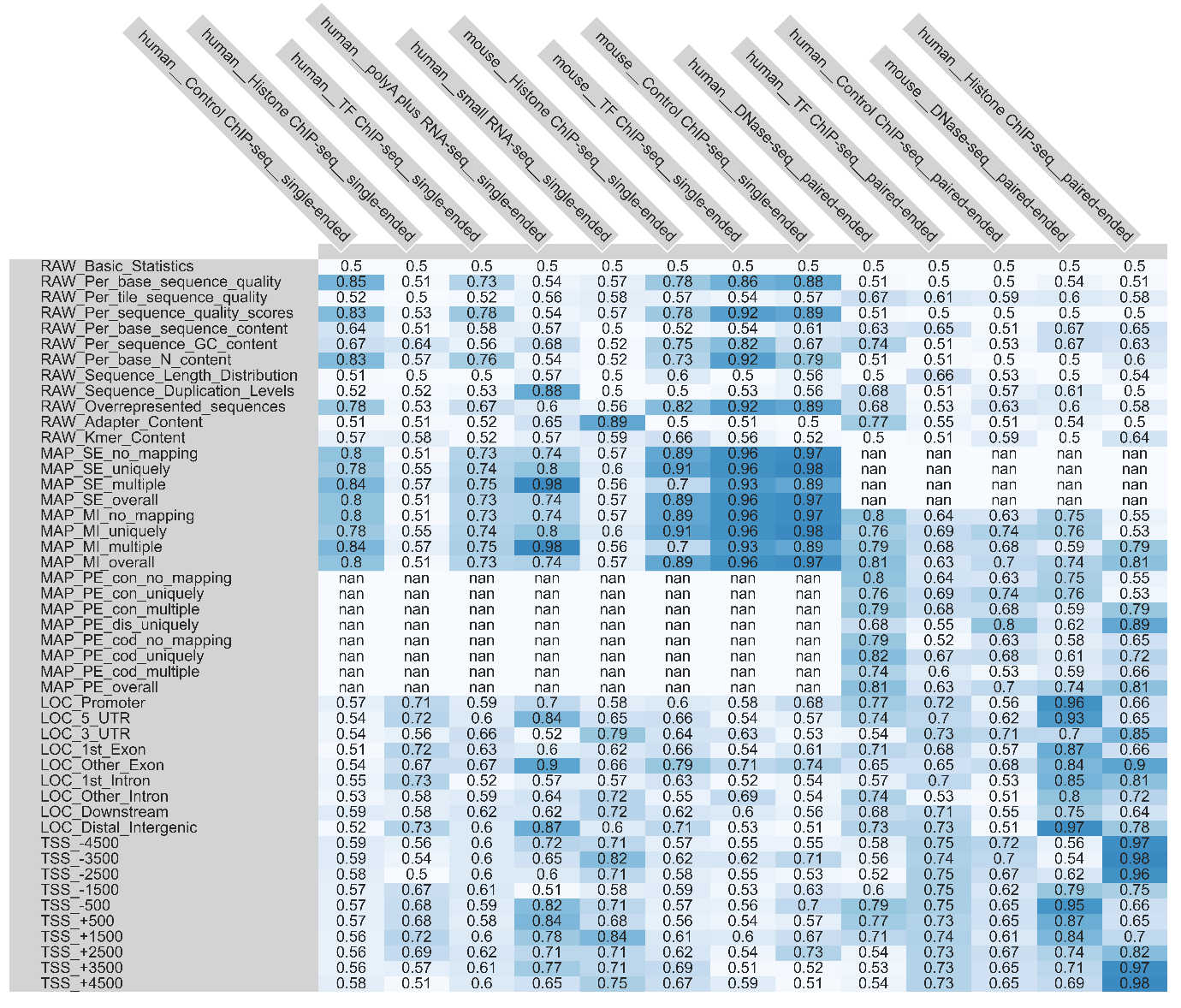

Supplement: Supplementary file 7 [file LSA-2021-01113_TableS6.docx]
