# Supplementary material for: Statistical guidelines for quality control of next-generation sequencing techniques
Source: Life Sci Alliance. 2021 Aug 30;4(11):e202101113. doi: 10.26508/lsa.202101113 (PMC8408346; doi:10.26508/lsa.202101113)
Supplement: Supplementary file 8 [file LSA-2021-01113_TableS7.docx]

## Table S7 – Classification performance of the decision trees (classification on the training set)

|  | **Subset** | **Accuracy** | **Precision low quality** | **Precision high quality** | **Recall low quality** | **Recall high quality** | **F1-Score low quality** | **F1-Score high quality** |
| --- | --- | --- | --- | --- | --- | --- | --- | --- |
| **human single-ended TF ChIP-seq in sample: H1** | **A** | **0.97** | **0.98** | **0.94** | **0.98** | **0.94** | **0.98** | **0.94** |
| **human single-ended TF ChIP-seq in sample: HeLa-S3** | **A** | **0.92** | **0.92** | **0.92** | **0.97** | **0.79** | **0.95** | **0.85** |
| **human single-ended TF ChIP-seq in sample: HepG2** | **A** | **0.80** | **1.00** | **0.63** | **0.69** | **1.00** | **0.82** | **0.77** |
| **human single-ended TF ChIP-seq in sample: K562** | **A** | **0.85** | **0.93** | **0.71** | **0.85** | **0.85** | **0.89** | **0.77** |
| **human single-ended polyA plus RNA-seq in sample: GM12878** | **A** | **1.00** | **1.00** | **1.00** | **1.00** | **1.00** | **1.00** | **1.00** |
| **human single-ended TF ChIP-seq in sample: A549** | **A** | **0.94** | **0.93** | **0.95** | **0.96** | **0.90** | **0.95** | **0.92** |
| **human single-ended TF ChIP-seq in sample: GM12878** | **A** | **0.95** | **0.94** | **1.00** | **1.00** | **0.75** | **0.97** | **0.86** |
| **human single-ended Control ChIP-seq in sample: GM12878** | **A** | **1.00** | **1.00** | **1.00** | **1.00** | **1.00** | **1.00** | **1.00** |
| **human single-ended TF ChIP-seq in sample: MCF-7** | **A** | **1.00** | **1.00** | **1.00** | **1.00** | **1.00** | **1.00** | **1.00** |
| **human single-ended TF ChIP-seq in sample: SK-N-SH** | **A** | **1.00** | **1.00** | **1.00** | **1.00** | **1.00** | **1.00** | **1.00** |
| **human single-ended Histone ChIP-seq in sample: MCF-7** | **A** | **1.00** | **1.00** | **1.00** | **1.00** | **1.00** | **1.00** | **1.00** |
| **human single-ended Control ChIP-seq in sample: HepG2** | **A** | **1.00** | **1.00** | **1.00** | **1.00** | **1.00** | **1.00** | **1.00** |
| **human single-ended TF ChIP-seq in sample: IMR-90** | **A** | **1.00** | **1.00** | **1.00** | **1.00** | **1.00** | **1.00** | **1.00** |
| **human single-ended TF ChIP-seq in sample: MCF_10A** | **A** | **1.00** | **1.00** | **1.00** | **1.00** | **1.00** | **1.00** | **1.00** |
| **mouse single-ended TF ChIP-seq in sample: MEL_cell_line** | **A** | **1.00** | **1.00** | **1.00** | **1.00** | **1.00** | **1.00** | **1.00** |
| **mouse single-ended Control ChIP-seq in sample: MEL_cell_line** | **A** | **1.00** | **1.00** | **1.00** | **1.00** | **1.00** | **1.00** | **1.00** |
| **mouse single-ended TF ChIP-seq in sample: CH12.LX** | **A** | **1.00** | **1.00** | **1.00** | **1.00** | **1.00** | **1.00** | **1.00** |
| **human paired-ended TF ChIP-seq in sample: HepG2** | **A** | **1.00** | **1.00** | **1.00** | **1.00** | **1.00** | **1.00** | **1.00** |
| **human paired-ended TF ChIP-seq in sample: K562** | **A** | **1.00** | **1.00** | **1.00** | **1.00** | **1.00** | **1.00** | **1.00** |
| **human paired-ended TF ChIP-seq in sample: MCF-7** | **A** | **1.00** | **1.00** | **1.00** | **1.00** | **1.00** | **1.00** | **1.00** |
| **human paired-ended TF ChIP-seq in sample: GM12878** | **A** | **1.00** | **1.00** | **1.00** | **1.00** | **1.00** | **1.00** | **1.00** |
| **human paired-ended DNase-seq in sample: transverse_colon** | **A** | **1.00** | **1.00** | **1.00** | **1.00** | **1.00** | **1.00** | **1.00** |
| **human paired-ended DNase-seq in sample: thyroid_gland** | **A** | **1.00** | **1.00** | **1.00** | **1.00** | **1.00** | **1.00** | **1.00** |
| **human paired-ended TF ChIP-seq in sample: HEK293** | **A** | **0.89** | **0.90** | **0.88** | **0.90** | **0.88** | **0.90** | **0.88** |
| **human single-ended Histone ChIP-seq in sample: gastrocnemius_medialis** | **A** | **1.00** | **1.00** | **1.00** | **1.00** | **1.00** | **1.00** | **1.00** |
| **human paired-ended DNase-seq in sample: body_of_pancreas** | **A** | **1.00** | **1.00** | **1.00** | **1.00** | **1.00** | **1.00** | **1.00** |
| **mouse paired-ended DNase-seq in sample: limb** | **A** | **1.00** | **1.00** | **1.00** | **1.00** | **1.00** | **1.00** | **1.00** |
| **mouse paired-ended DNase-seq in sample: hindbrain** | **A** | **1.00** | **1.00** | **1.00** | **1.00** | **1.00** | **1.00** | **1.00** |
| **mouse paired-ended DNase-seq in sample: midbrain** | **A** | **1.00** | **1.00** | **1.00** | **1.00** | **1.00** | **1.00** | **1.00** |
| **human paired-ended DNase-seq in sample: limb** | **A** | **1.00** | **1.00** | **1.00** | **1.00** | **1.00** | **1.00** | **1.00** |
| **human paired-ended Control ChIP-seq in sample: PC-9** | **A** | **1.00** | **1.00** | **1.00** | **1.00** | **1.00** | **1.00** | **1.00** |
| **human paired-ended DNase-seq in sample: neural_progenitor_cell** | **A** | **1.00** | **1.00** | **1.00** | **1.00** | **1.00** | **1.00** | **1.00** |
| **human single-ended Control ChIP-seq with target: Control** | **B** | **0.95** | **1.00** | **0.94** | **0.63** | **1.00** | **0.77** | **0.97** |
| **human single-ended Histone ChIP-seq with target: H3K4me1** | **B** | **1.00** | **1.00** | **1.00** | **1.00** | **1.00** | **1.00** | **1.00** |
| **human single-ended Histone ChIP-seq with target: H3K36me3** | **B** | **0.97** | **1.00** | **0.95** | **0.91** | **1.00** | **0.95** | **0.98** |
| **human single-ended Histone ChIP-seq with target: H3K27ac** | **B** | **1.00** | **1.00** | **1.00** | **1.00** | **1.00** | **1.00** | **1.00** |
| **human single-ended Histone ChIP-seq with target: H3K27me3** | **B** | **1.00** | **1.00** | **1.00** | **1.00** | **1.00** | **1.00** | **1.00** |
| **human single-ended Histone ChIP-seq with target: H3K4me3** | **B** | **0.98** | **1.00** | **0.98** | **0.67** | **1.00** | **0.80** | **0.99** |
| **human single-ended TF ChIP-seq with target: POLR2A** | **B** | **1.00** | **1.00** | **1.00** | **1.00** | **1.00** | **1.00** | **1.00** |
| **human single-ended Histone ChIP-seq with target: H3K9me3** | **B** | **1.00** | **1.00** | **1.00** | **1.00** | **1.00** | **1.00** | **1.00** |
| **human single-ended Histone ChIP-seq with target: H3K4me2** | **B** | **1.00** | **1.00** | **1.00** | **1.00** | **1.00** | **1.00** | **1.00** |
| **human single-ended TF ChIP-seq with target: CTCF** | **B** | **1.00** | **1.00** | **1.00** | **1.00** | **1.00** | **1.00** | **1.00** |
| **human single-ended TF ChIP-seq with target: EP300** | **B** | **1.00** | **1.00** | **1.00** | **1.00** | **1.00** | **1.00** | **1.00** |
| **human single-ended polyA plus RNA-seq with target: nan** | **B** | **1.00** | **1.00** | **1.00** | **1.00** | **1.00** | **1.00** | **1.00** |
| **human single-ended small RNA-seq with target: nan** | **B** | **1.00** | **1.00** | **1.00** | **1.00** | **1.00** | **1.00** | **1.00** |
| **human single-ended TF ChIP-seq with target: NR3C1** | **B** | **1.00** | **1.00** | **1.00** | **1.00** | **1.00** | **1.00** | **1.00** |
| **human single-ended TF ChIP-seq with target: MAX** | **B** | **1.00** | **1.00** | **1.00** | **1.00** | **1.00** | **1.00** | **1.00** |
| **human single-ended TF ChIP-seq with target: MYC** | **B** | **1.00** | **1.00** | **1.00** | **1.00** | **1.00** | **1.00** | **1.00** |
| **human single-ended Control ChIP-seq with target: rabbit-IgG-control** | **B** | **1.00** | **1.00** | **1.00** | **1.00** | **1.00** | **1.00** | **1.00** |
| **human single-ended TF ChIP-seq with target: RELA** | **B** | **1.00** | **1.00** | **1.00** | **1.00** | **1.00** | **1.00** | **1.00** |
| **human single-ended TF ChIP-seq with target: CHD1** | **B** | **1.00** | **1.00** | **1.00** | **1.00** | **1.00** | **1.00** | **1.00** |
| **human single-ended TF ChIP-seq with target: RCOR1** | **B** | **1.00** | **1.00** | **1.00** | **1.00** | **1.00** | **1.00** | **1.00** |
| **human single-ended TF ChIP-seq with target: ELK1** | **B** | **1.00** | **1.00** | **1.00** | **1.00** | **1.00** | **1.00** | **1.00** |
| **human single-ended TF ChIP-seq with target: JUND** | **B** | **1.00** | **1.00** | **1.00** | **1.00** | **1.00** | **1.00** | **1.00** |
| **human single-ended TF ChIP-seq with target: MAZ** | **B** | **1.00** | **1.00** | **1.00** | **1.00** | **1.00** | **1.00** | **1.00** |
| **human single-ended TF ChIP-seq with target: MXI1** | **B** | **1.00** | **1.00** | **1.00** | **1.00** | **1.00** | **1.00** | **1.00** |
| **human single-ended TF ChIP-seq with target: STAT3** | **B** | **1.00** | **1.00** | **1.00** | **1.00** | **1.00** | **1.00** | **1.00** |
| **human single-ended TF ChIP-seq with target: BACH1** | **B** | **1.00** | **1.00** | **1.00** | **1.00** | **1.00** | **1.00** | **1.00** |
| **human single-ended TF ChIP-seq with target: TCF7L2** | **B** | **1.00** | **1.00** | **1.00** | **1.00** | **1.00** | **1.00** | **1.00** |
| **human single-ended TF ChIP-seq with target: FOS** | **B** | **1.00** | **1.00** | **1.00** | **1.00** | **1.00** | **1.00** | **1.00** |
| **mouse single-ended Histone ChIP-seq with target: H3K27me3** | **B** | **1.00** | **1.00** | **1.00** | **1.00** | **1.00** | **1.00** | **1.00** |
| **mouse single-ended Control ChIP-seq with target: Control** | **B** | **1.00** | **1.00** | **1.00** | **1.00** | **1.00** | **1.00** | **1.00** |
| **mouse single-ended Histone ChIP-seq with target: H3K27ac** | **B** | **1.00** | **1.00** | **1.00** | **1.00** | **1.00** | **1.00** | **1.00** |
| **mouse single-ended Histone ChIP-seq with target: H3K4me3** | **B** | **0.96** | **0.93** | **1.00** | **1.00** | **0.90** | **0.97** | **0.95** |
| **mouse single-ended Histone ChIP-seq with target: H3K36me3** | **B** | **1.00** | **1.00** | **1.00** | **1.00** | **1.00** | **1.00** | **1.00** |
| **mouse single-ended Histone ChIP-seq with target: H3K4me1** | **B** | **1.00** | **1.00** | **1.00** | **1.00** | **1.00** | **1.00** | **1.00** |
| **mouse single-ended TF ChIP-seq with target: POLR2A** | **B** | **1.00** | **1.00** | **1.00** | **1.00** | **1.00** | **1.00** | **1.00** |
| **mouse single-ended TF ChIP-seq with target: CTCF** | **B** | **1.00** | **1.00** | **1.00** | **1.00** | **1.00** | **1.00** | **1.00** |
| **mouse single-ended Histone ChIP-seq with target: H3K9me3** | **B** | **1.00** | **1.00** | **1.00** | **1.00** | **1.00** | **1.00** | **1.00** |
| **human paired-ended DNase-seq with target: nan** | **B** | **0.91** | **0.90** | **0.92** | **0.92** | **0.89** | **0.91** | **0.91** |
| **human paired-ended Control ChIP-seq with target: Control** | **B** | **0.94** | **1.00** | **0.93** | **0.73** | **1.00** | **0.84** | **0.96** |
| **mouse paired-ended DNase-seq with target: nan** | **B** | **0.98** | **0.95** | **1.00** | **1.00** | **0.95** | **0.98** | **0.98** |
| **human single-ended Control ChIP-seq with target: Control and antibody: nan** | **C** | **0.95** | **1.00** | **0.94** | **0.63** | **1.00** | **0.77** | **0.97** |
| **human single-ended Histone ChIP-seq with target: H3K4me1 and antibody: ENCAB000ADW** | **C** | **1.00** | **1.00** | **1.00** | **1.00** | **1.00** | **1.00** | **1.00** |
| **human single-ended Histone ChIP-seq with target: H3K36me3 and antibody: ENCAB000ADU** | **C** | **1.00** | **1.00** | **1.00** | **1.00** | **1.00** | **1.00** | **1.00** |
| **human single-ended Histone ChIP-seq with target: H3K27me3 and antibody: ENCAB000ANB** | **C** | **1.00** | **1.00** | **1.00** | **1.00** | **1.00** | **1.00** | **1.00** |
| **human single-ended polyA plus RNA-seq with target: nan and antibody: nan** | **C** | **1.00** | **1.00** | **1.00** | **1.00** | **1.00** | **1.00** | **1.00** |
| **human single-ended small RNA-seq with target: nan and antibody: nan** | **C** | **1.00** | **1.00** | **1.00** | **1.00** | **1.00** | **1.00** | **1.00** |
| **human single-ended TF ChIP-seq with target: MAX and antibody: ENCAB000AIL** | **C** | **1.00** | **1.00** | **1.00** | **1.00** | **1.00** | **1.00** | **1.00** |
| **human single-ended TF ChIP-seq with target: POLR2A and antibody: ENCAB000AOC** | **C** | **1.00** | **1.00** | **1.00** | **1.00** | **1.00** | **1.00** | **1.00** |
| **human single-ended TF ChIP-seq with target: MYC and antibody: ENCAB000AET** | **C** | **1.00** | **1.00** | **1.00** | **1.00** | **1.00** | **1.00** | **1.00** |
| **human single-ended Control ChIP-seq with target: rabbit-IgG-control and antibody: ENCAB000AOJ** | **C** | **1.00** | **1.00** | **1.00** | **1.00** | **1.00** | **1.00** | **1.00** |
| **human single-ended TF ChIP-seq with target: RELA and antibody: ENCAB000AJG** | **C** | **1.00** | **1.00** | **1.00** | **1.00** | **1.00** | **1.00** | **1.00** |
| **human single-ended TF ChIP-seq with target: JUND and antibody: ENCAB000AID** | **C** | **1.00** | **1.00** | **1.00** | **1.00** | **1.00** | **1.00** | **1.00** |
| **human single-ended TF ChIP-seq with target: MAZ and antibody: ENCAB000AIM** | **C** | **1.00** | **1.00** | **1.00** | **1.00** | **1.00** | **1.00** | **1.00** |
| **human single-ended TF ChIP-seq with target: MXI1 and antibody: ENCAB000AIT** | **C** | **1.00** | **1.00** | **1.00** | **1.00** | **1.00** | **1.00** | **1.00** |
| **human single-ended TF ChIP-seq with target: STAT3 and antibody: ENCAB000ALH** | **C** | **1.00** | **1.00** | **1.00** | **1.00** | **1.00** | **1.00** | **1.00** |
| **human single-ended TF ChIP-seq with target: BACH1 and antibody: ENCAB000AEA** | **C** | **1.00** | **1.00** | **1.00** | **1.00** | **1.00** | **1.00** | **1.00** |
| **human single-ended TF ChIP-seq with target: TCF7L2 and antibody: ENCAB000AOA** | **C** | **1.00** | **1.00** | **1.00** | **1.00** | **1.00** | **1.00** | **1.00** |
| **human single-ended TF ChIP-seq with target: FOS and antibody: ENCAB000AEQ** | **C** | **1.00** | **1.00** | **1.00** | **1.00** | **1.00** | **1.00** | **1.00** |
| **mouse single-ended Control ChIP-seq with target: Control and antibody: nan** | **C** | **1.00** | **1.00** | **1.00** | **1.00** | **1.00** | **1.00** | **1.00** |
| **mouse single-ended TF ChIP-seq with target: POLR2A and antibody: ENCAB281FBQ** | **C** | **1.00** | **1.00** | **1.00** | **1.00** | **1.00** | **1.00** | **1.00** |
| **mouse single-ended TF ChIP-seq with target: CTCF and antibody: ENCAB210NHK** | **C** | **1.00** | **1.00** | **1.00** | **1.00** | **1.00** | **1.00** | **1.00** |
| **human single-ended Histone ChIP-seq with target: H3K27ac and antibody: ENCAB000AQN** | **C** | **1.00** | **1.00** | **1.00** | **1.00** | **1.00** | **1.00** | **1.00** |
| **human paired-ended DNase-seq with target: nan and antibody: nan** | **C** | **0.91** | **0.90** | **0.92** | **0.92** | **0.89** | **0.91** | **0.91** |
| **human paired-ended Control ChIP-seq with target: Control and antibody: nan** | **C** | **0.94** | **1.00** | **0.93** | **0.73** | **1.00** | **0.84** | **0.96** |
| **mouse paired-ended DNase-seq with target: nan and antibody: nan** | **C** | **0.98** | **0.95** | **1.00** | **1.00** | **0.95** | **0.98** | **0.98** |
